# Pd-Catalyzed Regioselective Cyclopropanation of 2-Substituted 1,3-Dienes

**DOI:** 10.1021/acsorginorgau.3c00024

**Published:** 2023-07-25

**Authors:** Agonist Kastrati, Vincent Jaquier, Michele Garbo, Céline Besnard, Clément Mazet

**Affiliations:** † Department of Organic Chemistry, University of Geneva, 30 Quai Ernest Ansermet, 1211 Geneva, Switzerland; ‡ Laboratory of Crystallography, University of Geneva, 24 Quai Ernest Ansermet, 1211 Geneva, Switzerland

**Keywords:** palladium catalysis, conjugated dienes, selective catalysis, cyclopropanation, vinylcyclopropanes

## Abstract

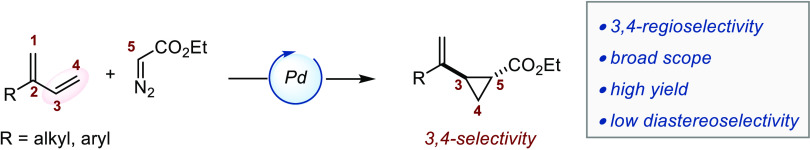

A Pd-catalyzed 3,4-regioselective cyclopropanation of 2-substituted 1,3-dienes by decomposition of diazo esters is reported. The vinylcyclopropanes generated are isolated in practical chemical yields with high levels of regioselectivity but low diastereoselectivity. The system operates under mild reaction conditions, is scalable, and tolerates various sensitive functional groups. A series of original postcatalytic derivatizations is presented to highlight the synthetic potential of the catalytic method.

Vinylcyclopropanes (VCPs) are highly prevalent structural motifs in natural and synthetic bioactive molecules.^1−5^ Owing to their propensity to ring-open in the presence of transition-metal catalysts, their reactivity has been widely studied and they are now frequently used as platforms for further transformations.^6−12^ Retrosynthetically, the transition-metal-catalyzed cyclopropanation of dienes based on diazoalkane decomposition ranks among the most direct routes for their preparation.^13,14^ In practice, while this is certainly true for symmetrical substrates where both alkenes are equivalent, the situation is more contrasted for unsymmetrical 1,3-dienes. Indeed, the highly enantio- and diastereoselective Cu-catalyzed cyclopropanation of 2,5-dimethyl-2,4-hexadiene (DMHD) for the production of pyrethroids was brought to industrial scale by Aratani and his group at Sumimoto Co., in the 1980s (Figure 1A).^2^ Today, it still constitutes one of the most significant achievements of selective homogeneous catalysis. In comparison, until recently, the cyclopropanation of 1,3-dienes featuring two distinct alkenes was considered of poor synthetic utility because of the low levels of regio- and diastereoselectivity obtained with the transition-metal catalysts typically used for simple olefins.^15−17^ Two recent studies have begun to address these shortcomings. Our group has shown that, using readily available diazo esters, the chiral Cu-bisoxazoline system catalyzes the cyclopropanation of 2-substituted 1,3-dienes with excellent levels of regio- and enantioselectivity but modest *trans*/*cis* selectivity (from 1:1 to 2:1) (Figure 1B). Because the ester stereocenter (C5) is controlled by the chiral ligand, the lack of diastereocontrol at C2 was circumvented by engaging the VCP mixtures in a subsequent Rh-catalyzed stereoconvergent intermolecular (5 + 2) cycloaddition with a variety of alkynes. Overall, this sequential approach yielded 7-membered rings with high levels of enantiopurity.^18^ Concurrently to this study, Uyeda and co-workers reported a unique dinuclear Ni catalyst for the cyclopropanation of branched dienes using silylated diazoalkanes. Cyclopropanation occurred exclusively at the most substituted double bond (*rr*_(1,2/3,4)_ > 20:1), and the corresponding racemic VCPs were isolated with moderate to excellent levels of diastereoselectivity (Figure 1C). Quite notably, an unusual diradical mechanism distinct from the classical (2 + 1) cycloaddition involving M = CR_2_ intermediates was established.^19^ Following these advances, we sought to identify a complementary catalytic system for the regioselective cyclopropanation of the terminal alkene in 2-substituted 1,3-dienes (Figure 1D). We report herein the results of our investigations in this direction.

**Figure 1:**
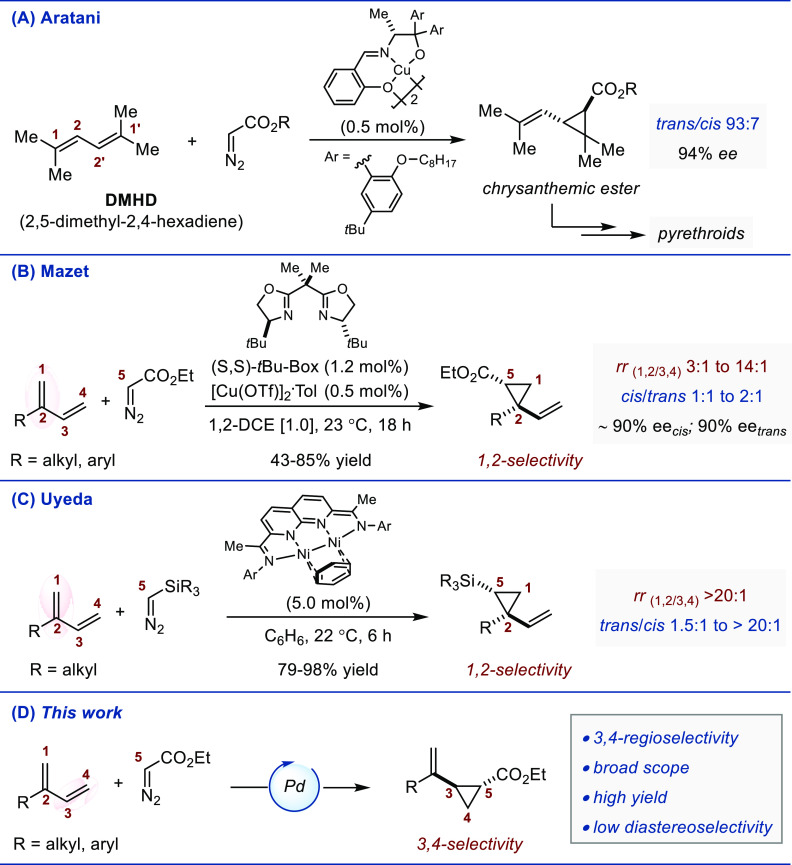
(A) Cu-catalyzed enantioselective cyclopropanation of DMHD. (B) Cu-catalyzed 1,2-regioselective and enantioselective cyclopropanation of branched dienes. (C) Ni-catalyzed 1,2-regioselective, and diastereoselective cyclopropanation of 2-substituted 1,3-dienes. (D) Pd-catalyzed 3,4-regioselective cyclopropanation of 2-substituted 1,3-dienes.

The renewed interest in Ni and Pd catalysis for the development of cyclopropanation reactions prompted us to initiate our investigations with the evaluation of group X transition-metal precatalysts using branched diene **1a** as a model substrate and ethyl diazo acetate **2a** (Table 1).^20−26^ Whereas no reaction was observed with several nickel sources, most of the Pd(II) precatalysts yielded diethyl fumarate and diethyl maleate (entries 1–3 and 5–7). Cyclopropanation occurred to a marginal extent using Pd(OAc)_2_ but with excellent 3,4-regioselectivity (12% conv., *rr*_**3**/**4**_ > 20:1; entry 4). The use of Pd_2_(dba)_3_ (>98% purity based on Ananikov’s method)^27^ led to a noticeable increase in catalytic activity, a similarly high level of regiocontrol, but essentially no diastereocontrol (entry 8). While catalytic inhibition was observed when P- or N-based ligands were employed, 64% conv. in **3a** (*rr*_**3**/**4**_ > 20:1) was achieved in tetrahydrofuran (THF) after a brief solvent survey (entries 9–12). The commercially available [(NHC)Pd(0)] complexes **C**_**2**_ and **C**_**3**_ displayed significant reactivity and selectivity but did not outcompete Pd_2_(dba)_3_ (entries 13 and 14). In the absence of Lewis acid, no polymerization of ethyl diazo acetate was observed with **C**_**2**_ and **C**_**3**_.^28,29^ Finally, we found that the optimal results were obtained with **C**_**4**_, an underused though readily available Pd(0) precursor (69% conv., *rr*_**3**/**4**_ > 20:1, *trans*/*cis* 1:1; entry 14).^30^ Under these conditions, the less reactive diazomalonate **2b** enabled the regioselective cyclopropanation of **1a** into VCP **3b** in 47% conversion (*rr*_**3**/**4**_ > 20:1), a result that could not be improved at higher temperature (entries 19 and 20).

**Table 1:**
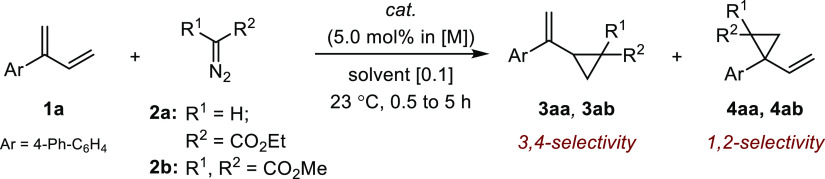
Reaction Optimization^a^

entry	catalyst	**2**	solvent	conv. (%)^b^	*rr* (_3,4/1,2_)^b^	*trans*/*cis*^b^
1	Ni(cod)_2_	**2a**	toluene	nr^c^		
2	Ni(cod)(DQ)	**2a**	toluene	nr		
3	NiCl_2_(PPh_3_)_2_	**2a**	toluene	nr		
4	Pd(OAc)_2_	**2a**	toluene	12	>20:1	1.4:1.0
5	PdCl_2_(cod)	**2a**	toluene	nr		
6	(CPhos)Pd(G3)	**2a**	toluene	nr		
7	**C**_**1**_	**2a**	toluene	nr		
8	Pd_2_(dba)_3_	**2a**	toluene	37	>20:1	1.3:1.0
9	Pd_2_(dba)_3_/**L**_**1**_	**2a**	toluene	9	>20:1	2.0:1.0
10	Pd_2_(dba)_3_/PCy_3_^d^	**2a**	toluene	<5	nd^e^	nd
11	Pd_2_(dba)_3_	**2a**	CH_2_Cl_2_	31	>20:1	1.5:1.0
12	Pd_2_(dba)_3_	**2a**	THF	64	>20:1	1.2:1.0
13	**C**_**2**_	**2a**	THF	38	>20:1	2.0:1.0
14	**C**_**3**_	**2a**	THF	41	>20:1	1.2:1.0
15	**C**_**4**_	**2a**	THF	69	>20:1	1.0:1.0
16	**C**_**4**_/**L**_**1**_	**2a**	THF	nr		
17	**C**_**4**_/PCy_3_^d^	**2a**	THF	18	>20:1	1.0:1.0
18	**C**_**3**_	**2b**	THF	nr		
19	**C**_**4**_	**2b**	THF	47	>20:1	na^g^
20^f^	**C**_**4**_	**2b**	THF	23	>20:1	na

a Reaction conditions: **1a** (0.1 mmol), **2a–b** (0.12–0.15 mmol).
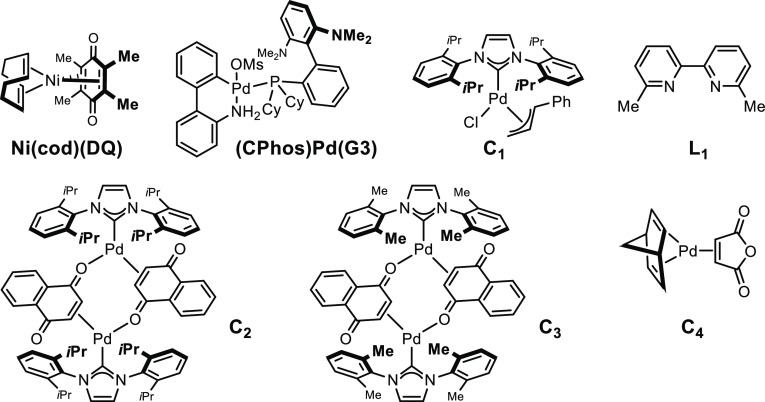

b Determined by ^1^H NMR using an internal standard.

c No reaction.

d 10 mol % of PCy_3_.

e Not determined.

f At 60 °C.

g Not applicable.

The generality of the optimized protocol was subsequently evaluated with a representative selection of 2-substituted 1,3-dienes **1a**–**n** using ethyl diazo acetate **2a** (Figure 2). Substrates containing an electron-poor, an electron-neutral, an electron-rich aromatic ring, or a heteroaromatic substituent delivered the vinylcyclopropanes in usually high yield, low *cis*/*trans* ratio but with consistently excellent 3,4-regioselectivity. Only the 3-thiophene derivative **3ia** was isolated in low yield (38% yield). *Ortho*-substitution was well-tolerated (**3da**–**3fa**). Primary, secondary, and even sterically demanding tertiary aliphatic derivatives were cyclopropanated with similar catalytic efficiency (**3ja**–**3na**). Among the diverse functional groups that were accommodated, the perfect chemo- and regioselectivity observed for substrates featuring a 1,2-(*Z*)-disubstituted alkenes (**3na**), as well as trisubstituted alkenes (**3fa**, **3ka**), is particularly noticeable.

**Figure 2:**
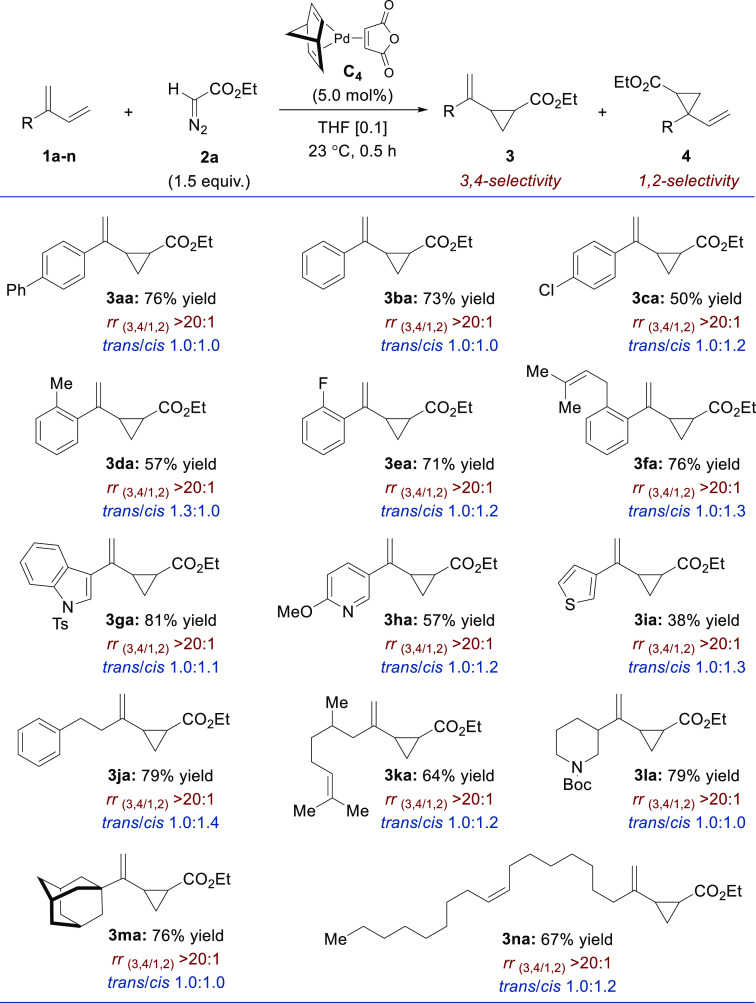
Scope of the Pd-catalyzed 3,4-regioselective cyclopropanation of 2-substituted 1,3-dienes (0.5 mmol scale). Regio- and diastereoselectivity determined by ^1^H NMR. Yield after purification.

The optimized reaction conditions using **C**_**4**_ were next applied to other classes of 1,3-dienes (Figure 3). We found that terminal diene **1o** underwent cyclopropanation with a very high level of 1,2-regioselectivity, affording **3oa** in 86% yield (*trans*/*cis* 1:1). In contrast, symmetrical dienes such as **1p** and **1q** were less reactive, delivering **3pa** and **3qa**/**3qa′** in 17% and 39% yields, respectively. The robustness of the cyclopropanation protocol was confirmed by successfully conducting the model reaction between **1a** and **2a** on a gram scale (Figure 4). Gratifyingly, the combined yield for this experiment was slightly improved, and, more importantly, we showed that both diastereoisomers could be separated by standard chromatographic purification affording 530 mg of *trans*-**3aa** and 560 mg of *cis*-**3aa**.

**Figure 3:**
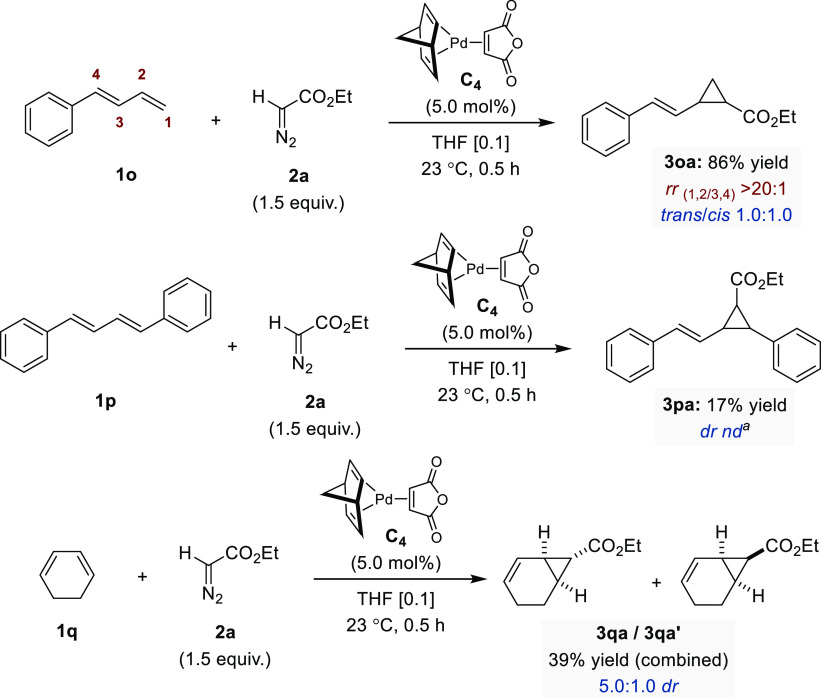
Pd-catalyzed cyclopropanation of differently substituted 1,3-dienes (0.5–1.0 mmol scale). Regio- and diastereoselectivity determined by ^1^H NMR. ^a^Not determined.

**Figure 4:**
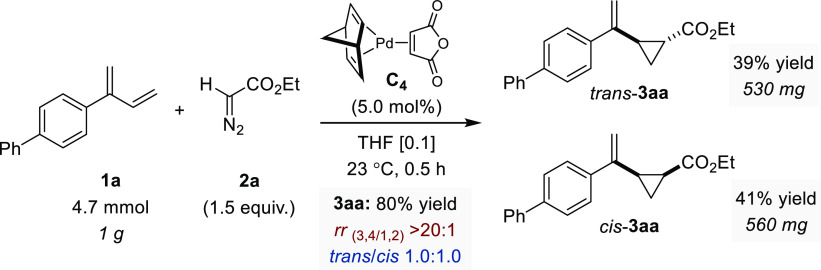
Large-scale experiment. Regio- and diastereoselectivity determined by ^1^H NMR.

The synthetic utility of the VCP obtained was demonstrated through a series of comparative postcatalytic derivatizations using *trans*-**3aa** and *cis*-**3aa** or the corresponding primary alcohols *trans*-**5aa** and *cis*-**5aa** prepared following a standard reduction procedure (see the Supporting Information for details). We initiated our investigations by evaluating two protocols recently reported by the Marek group for the cyclopropanation and epoxidation of densely substituted alkenyl cyclopropyl carbynols.^31^ Our interest stemmed from the fact that—to the best of our knowledge—the substitution pattern of the VCP generated by the Pd-catalyzed cyclopropanation of 2-substituted 1,3-dienes had not been explored in previous studies.^32−34^ While we did not observe product formation using diiodomethane for the Zn-mediated Simmons–Smith–Furukawa cyclopropanation of either *trans*-**5aa** or *cis*-**5aa**, reactivity was restored with the use of chloroiodomethane (Figure 5A).^35^ Biscyclopropyl carbinol *trans*-**6aa** was obtained in a 53% yield after purification by column chromatography starting from *trans*-**5aa**. In contrast, when *cis*-**5aa** was subjected to similar reaction conditions, cyclopropanation of the C=C bond was accompanied by competitive O–H insertion, a feature that is commonly observed with transition metals.^36,37^ The biscyclopropyl carbinol *cis*-**6aa** and the biscyclopropyl methyl ether *cis*-**7aa** thus generated could be separated by column chromatography and isolated in 28% and 48% yields, respectively. While no reaction occurred when attempting to perform a V-catalyzed cyclopropanation of *trans*-**5aa** using ^*t*^BuOOH as an oxidant, alkenyl cyclopropyl carbynol *cis*-**5aa** afforded **8aa** as a single diastereoisomer (Figure 5B).^38−40^ Unexpectedly, benzoylation of **8aa** using 3,5-dinitrobenzoyl chloride led to the diastereoselective formation of the 3-oxabicyclo[3.1.0]hexane derivative **9aa**, generated through intramolecular S_N_2 ring-opening of the epoxide by the pendant alcohol functionality and subsequent benzoylation. The relative stereochemistry of the three contiguous stereocenters in **8aa** and **9aa** was assigned by growing crystals of suitable quality for X-ray analysis of the latter. Finally, we found that Cu-catalyzed protoboration of both *trans*-**3aa** and *cis*-**3aa** led to the formation of the same ring-opened polyfunctional allyl boronate ester (*E*)-**10aa** in good yields and with excellent level of stereocontrol (*E*/*Z* > 20:1) (Figure 5C).^41−44^ Quite notably, the use of morpholine trifluoroacetic acid salt appeared crucial to avoid deborylation, a phenomenon we observed using various alcohols as proton sources. It is worth noting that the stereoconvergent nature of this unprecedented Cu-catalyzed protoboration alleviates the lack of stereocontrol of the Pd-catalyzed 3,4-regioselective cyclopropanation of branched dienes and obviates the need to separate the *cis* and *trans* VCPs.

**Figure 5:**
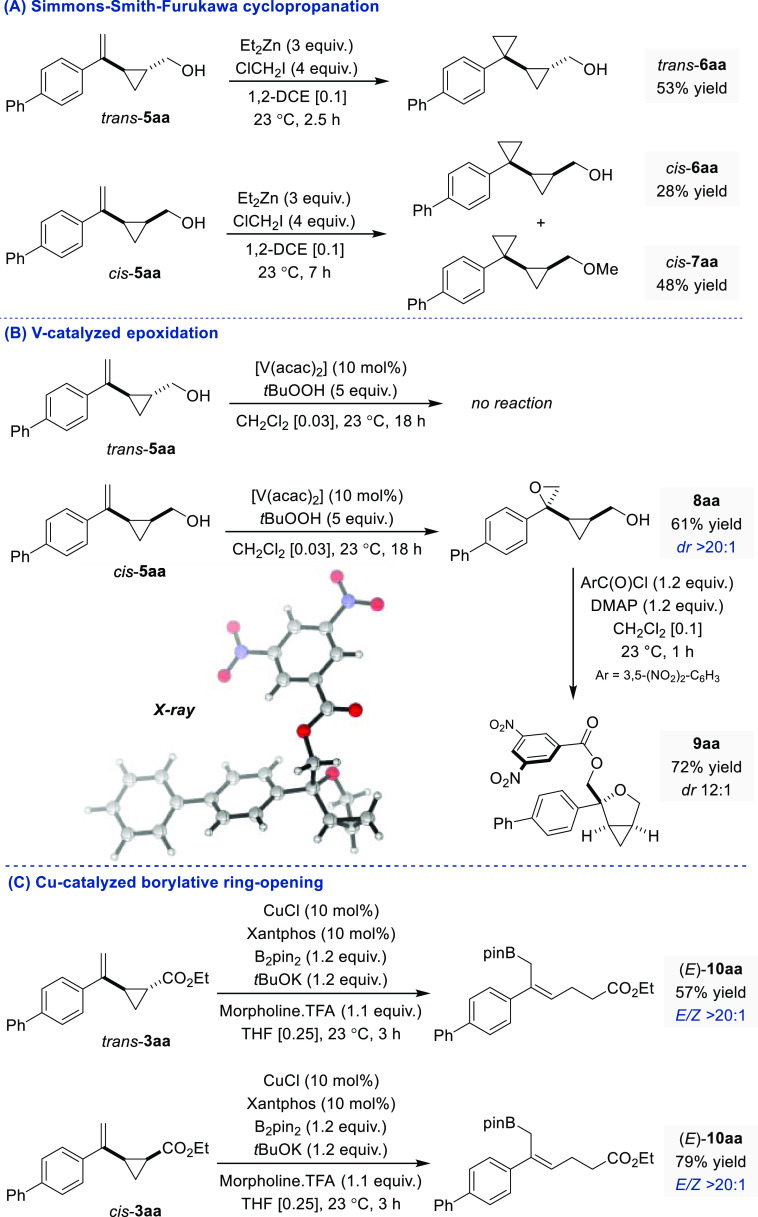
(A) Simmons–Smith–Furukawa cyclopropanations, (B) V-catalyzed epoxidations, and (C) Cu-catalyzed borylative ring-opening reactions.

In conclusion, we have developed a 3,4-regioselective Pd-catalyzed cyclopropanation of 2-substituted 1,3-dienes using readily available diazo esters. The vinylcyclopropanes generated are isolated in practical chemical yields with exquisite regioselectivity and low diastereoselectivity. The method operates under mild reaction conditions, is compatible with a wide number of potentially sensitive functional groups, and can be performed on a gram scale, thus allowing separation of the *cis* and *trans* isomers. A series of postcatalytic derivatizations served to highlight some of the intrinsic reactivity differences between these diastereoisomeric structures. Among these, an original stereoconvergent Cu-catalyzed ring-opening protoboration mitigated the modest level of stereocontrol of the catalytic cyclopropanation. Current efforts in our laboratory are directed toward understanding the origin of the high levels of regioselectivity obtained in the Pd-catalyzed 3,4-cyclopropanation of branched 1,3-dienes.

## Experimental Section

### General Cyclopropanation Procedure

In a N_2_-filled glovebox, a 15% solution of ethyl diazo acetate in toluene (**2a** or **2b**, 0.75 mmol, 1.50 equiv) was added at once to a Schlenk flask containing the appropriate diene **1a**–**n** (0.5 mmol, 1.00 equiv) and precatalyst **C**_**4**_, (5 mol %) in THF (5 mL, 0.1 M) at room temperature. The reaction mixture was stirred at 25 °C for 30 min. The Schlenk was taken out of the glovebox, and the reaction was quenched by dilution using THF (5 mL). The crude mixture was concentrated under reduced pressure and adsorbed in silica using CH_2_Cl_2_. Purification by flash chromatography using the appropriate eluent yielded the desired vinylcyclopropanes (VCPs).

## Data Availability

The data underlying this study are available in the published article and its Supporting Information.

## Supporting Information Available

Experimental procedures and characterization of all new compounds and X-ray data for compound **9aa** (PDF)
