# Supplementary material for: Pd-Catalyzed Regioselective Cyclopropanation of 2-Substituted 1,3-Dienes
Source: ACS Org Inorg Au. 2023 Jul 25;3(5):291–8. doi: 10.1021/acsorginorgau.3c00024 (PMC10557126; doi:10.1021/acsorginorgau.3c00024)

Pd-Catalyzed Regioselective Cyclopropanation of 2-Substituted 1,3-Dienes

Agonist Kastrati,[†] Vincent Jaquier,[†] Michele Garbo,[†] Céline Besnard,[‡] Clément Mazet*[,†]

[†] Department of Organic Chemistry, University of Geneva,
30 quai Ernest Ansermet, 1211 Geneva, Switzerland.

[‡] Laboratory of Crystallography, University of Geneva,
24 quai Ernest Ansermet, 1211 Geneva, Switzerland.

clement.mazet@unige.ch

Table of content

**1**	General information	3
**2**	Reaction optimization	4
**3**	Reaction scope	7
**4**	General procedure I (GPI)	8
**5**	Characterization of new compounds	9
**6**	Large scale experiment	33
**7**	X-ray analysis of **9aa**	34
**8**	References	36
**9**	NMR spectra of new compounds	38
**10**	^{1}H NMR spectra of known compounds	159

1. General Information

Unless otherwise noted, all reactions were carried out under an inert atmosphere of nitrogen using a M.Braun glove-box. Solvents were dried over activated alumina columns and further degassed by three successive "freeze-pump-thaw" cycles. All transition metal precatalysts and ligands were stored and weighted inside a M.Braun glove-box.

NMR spectrometers. NMR spectra were acquired at the University of Geneva NMR platform (https://www.unige.ch/sciences/chiorg/nmr/) using a 500 MHz Avance III Bruker NMR spectrometer equipped with a helium-cooled cryogenic 5-mm DCH ^{13}C-^{1}H/D Bruker probe, a 400 MHz Avance III HD NanoBay spectrometer equipped with a N_2 prodigy cryogenic 5 mm CPP BB(F)-H-D probe or a 300 MHz Avance III, HD NanoBay spectrometer, equipped with a 5 mm PA BBO, BB(F)-H-D probe. All ^{1}H and $^{13}C\{^{1}H\}$ experiments were internally referenced with respect to $CDCl_3$ solvent signals and acquired at 298 K or at 328 K. $^{31}P\{^{1}H\}$, $^{19}F\{^{1}H\}$ and $^{11}B\{^{1}H\}$ NMR chemical shifts are reported in ppm with reference to the lock.

Infrared spectra were obtained on a Perkin–Elmer spectrum 100 FT-IR spectrometer using neat samples on a diamond ATR Golden Gate sampler.

The mass spectrometric data were obtained at the mass spectrometry facility of the University of Geneva (http://www.unige.ch/sciences/sms/).

Thin layer chromatography (TLC) was performed on plates of silica pre-coated with 0.25 mm Kieselgel 60 F_{254} from *Merck*. Flash chromatography was performed using silica gel SiliaFlash® P60 (230-400 mesh) from *Silicycle* or aluminum oxide (neutral, Brockmann I, 40-300 mesh) from *Thermo scientific*.

All the dienes, precatalysts [Ni(cod)(DQ)], [(CPhos)Pd(G3)], **C_1**, **C_4** and diazo ester **2b** were synthesized according to literature procedures.[1-9]

2. Reaction optimization

Table S1: Catalyst screening

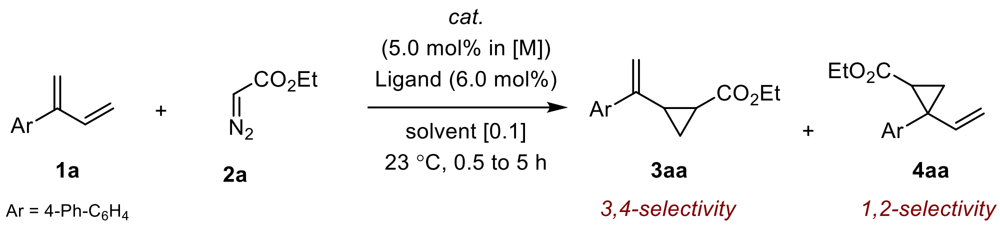

entry	catalyst	2	solvent	conv. (%)[b]	3/4[b]	*trans/cis*[b]
1	Ni(cod)$_2$	**2a**	Toluene	*nr*[c]	-	-
2	Ni(cod)(DQ)	**2a**	Toluene	*nr*	-	-
3	NiCl$_2$(PPh$_3$)$_2$	**2a**	Toluene	*nr*	-	-
4	Pd(OAc)$_2$	**2a**	Toluene	12	>20:1	1.4 : 1.0
5	PdCl$_2$(cod)	**2a**	Toluene	*nr*	-	-
6	(CPhos)Pd(G3)	**2a**	Toluene	*nr*	-	-
7	Pd$_2$(dba)$_3$/**L$_1$**	**2a**	Toluene	9	>20:1	2.0 : 1.0
8	Pd$_2$(dba)$_3$/PCy$_3$[d]	**2a**	Toluene	<5	*nd*[e]	*nd*
9	**C$_2$**	**2a**	THF	38	>20:1	2.0 : 1.0
10	**C$_3$**	**2a**	THF	41	>20:1	1.2 : 1.0
11	**C$_4$**	**2a**	THF	69	>20:1	1.0 : 1.0
12	**C$_4$**/**L$_1$**	**2a**	THF	*nr*	-	-
13	**C$_4$**/PCy$_3$[d]	**2a**	THF	18	>20:1	1.0: 1.0

Reaction optimization. [a] **1a** (0.1 mmol), **2a** (0.12-0.15 mmol). [b] Determined by ^{1}H NMR using an internal standard. [c] No reaction. [d] 10 mol% of PCy$_3$. [e] Not determined.

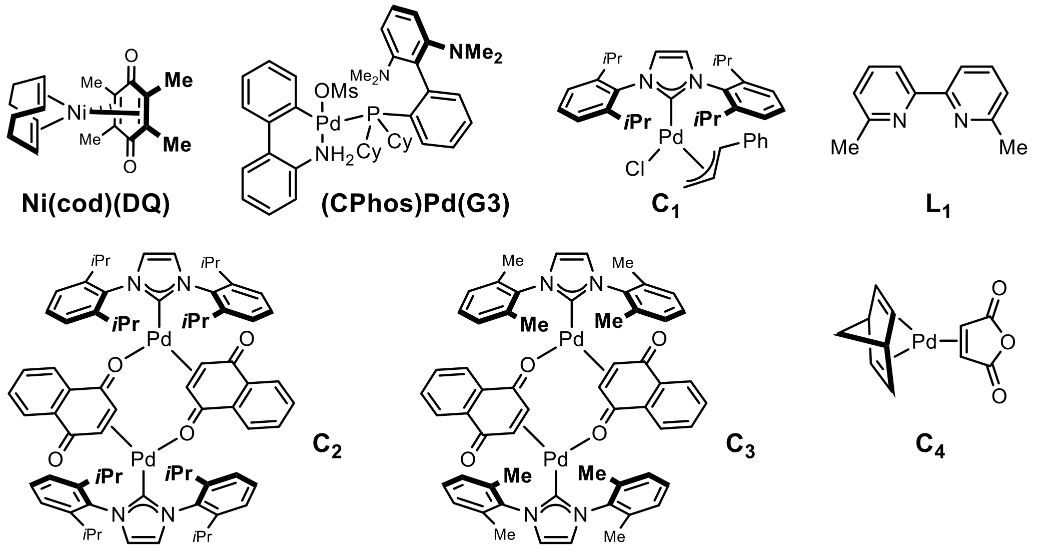

Table S2: Solvent screening

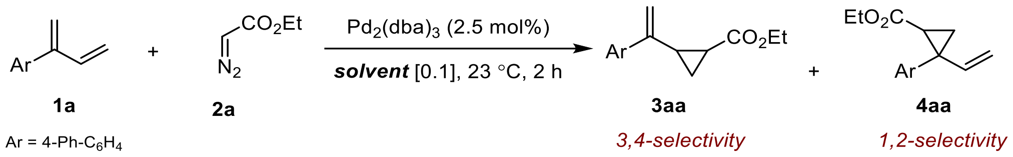

entry	catalyst	**2a** (equiv.)	solvent	conv. (%)[b]	**3**/**4**[b]	*trans/cis*[b]
1	$Pd_2(dba)_3$	1.2	Toluene	37	>20:1	1.3 : 1.0
2	$Pd_2(dba)_3$	1.2	CH_2Cl_2	31	>20:1	1.5 : 1.0
3	$Pd_2(dba)_3$	1.2	THF	64	>20:1	1.2 : 1.0
4	$Pd_2(dba)_3$	1.5	THF	73	>20:1	1.2 : 1.0

[a] **1a** (0.1 mmol), **2a** (0.12-0.15 mmol). [b] Determined by ^{1}H NMR using an internal standard.

Table S3: Cyclopropanation using dimethyl 2-diazomalonate **2b**

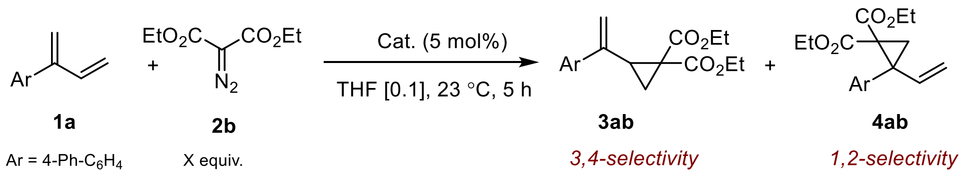

entry	catalyst	solvent	conv. (%)[b]	**3**/**4**[b]	*trans/cis*[b]
1	$\mathbf{C_3}$	THF	*nr*[c]	-	-
2	$\mathbf{C_4}$	THF	47	>20:1	*na*[e]
3[d]	$\mathbf{C_4}$	THF	23	>20:1	*na*

[a] **1a** (0.1 mmol), **2b** (0.15 mmol). [b] Determined by ^{1}H NMR using an internal standard. [c] No reaction. [d] At 60 °C. [e] Not applicable.

Table S4: Effect of the temperature

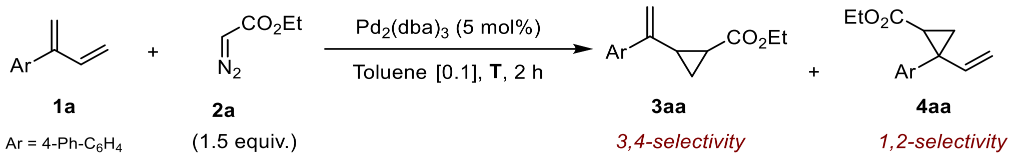

entry	catalyst	T (°C)	conv. (%)[b]	**3/4**[b]	*trans/cis*[b]
1	$Pd_2(dba)_3$	–20	21	>20:1	1.6 : 1.0
2	$Pd_2(dba)_3$	0	26	>20:1	1.4 : 1.0
3	$Pd_2(dba)_3$	40	30	>20:1	1.5 : 1.0
4	$Pd_2(dba)_3$	80	30	>20:1	1.5 : 1.0

[a] **1a** (0.1 mmol), **2a** (0.15 mmol). [b] Determined by ^{1}H NMR using an internal standard.

Table S5: Catalyst loading variation

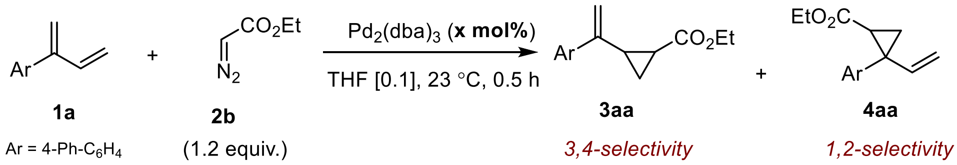

entry	catalyst	(x mol%)	conv. (%)[b]	**3/4**[b]	*trans/cis*[b]
1	$Pd_2(dba)_3$	0.5	10	>20:1	1.4 : 1.0
2	$Pd_2(dba)_3$	1.5	52	>20:1	1.2 : 1.0
3	$Pd_2(dba)_3$	2.5	64	>20:1	1.3 : 1.0

[a] **1a** (0.1 mmol), **2b** (0.12 mmol). [b] Determined by ^{1}H NMR using an internal standard.

3. Reaction scope

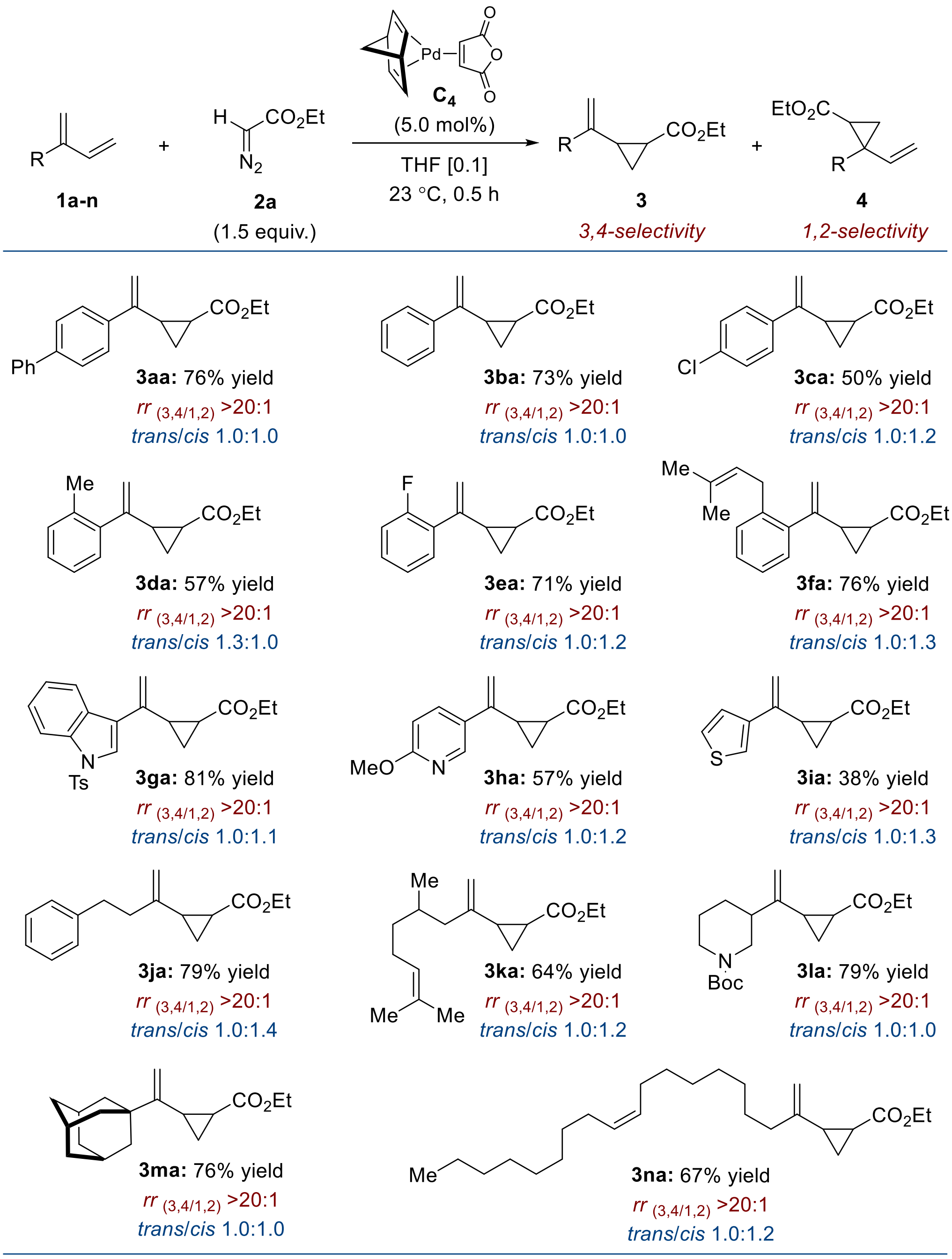

Figure S1. Scope of the Pd-catalyzed 3,4-regioselective cyclopropanation of 2-substituted 1,3-dienes (0.5 mmol scale). Regio- and diastereoselectivity determined by ^{1}H NMR. Yield after purification.

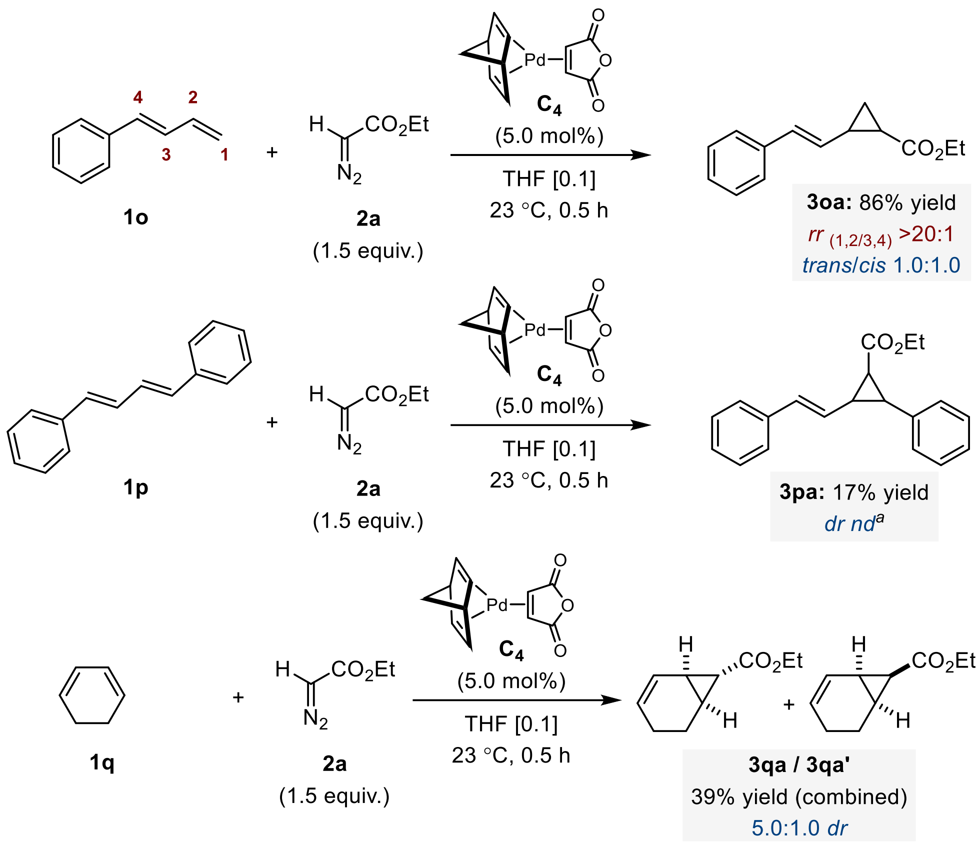

Figure S2. Pd-catalyzed cyclopropanation of differently substituted 1,3-dienes (0.5–1.0 mmol scale). Regio- and diastereoselectivity determined by ^{1}H NMR. [a] Not determined.

4. General procedure I (GPI)

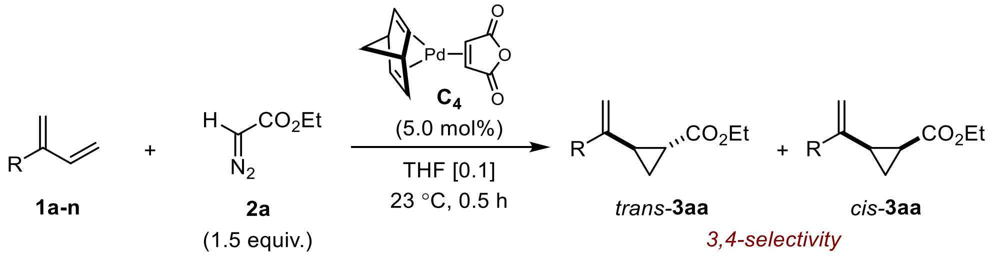

In a N_2-filled glovebox, a 15% solution of ethyl diazoacetate in toluene (**2a** or **2b**, 0.75 mmol, 1.50 equiv.) was added at once to a Schlenk flask containing the appropriate diene **1a-n** (0.5 mmol, 1.00 equiv.) and precatalyst $\mathbf{C_4}$, (5 mol%) in THF (5 mL, 0.1 M) at room temperature. The reaction mixture was stirred at 25 °C for 30 min. The Schlenk was taken out of the glovebox and the reaction was quenched by dilution using THF (5 mL). The crude mixture was concentrated under reduced pressure and adsorbed in silica using CH_2Cl_2. Purification by flash chromatography using the appropriate eluent yielded the desired vinylcyclopropanes (VCPs).

5. Characterization of new compounds

<u>Note</u>*: The majority of diastereoisomeric mixtures of the VCP reported hereafter could not be separated by column chromatography. The yields reported reflect the sum of both diastereoisomers and therefore the net efficiency of the catalytic process. In some cases, we have been able to separate small amounts of each isomer (cis and trans) and could provide both a detailed description and individual NMR spectra.*

Ethyl-2-(1-([1,1'-biphenyl]-4-yl)vinyl)cyclopropane-1-carboxylate (*trans*-**3aa**)

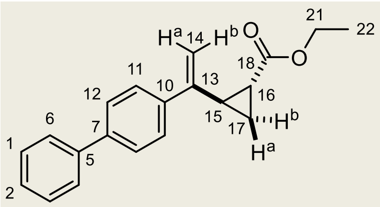

Trans-**3aa** was synthesized according to **GPI** on a 0.5 mmol scale using **1a** and **C4** (5 mol%, 7.42 mg) at 25 °C for 30 min. Purification by flash chromatography gave the desired compound as a white solid (54.2 mg, 0.18 mmol, 37%) (*trans*-**3aa** + *cis*-**3aa**: 76% yield, $dr_{(trans\text{-}\mathbf{3aa}/cis\text{-}\mathbf{3aa})}$ = 1.0 : 1.0). All spectroscopic data are in full agreement with those reported in the literature.[10]

TLC: Rf = 0.54 (pentane/Et_2O = 98:2).

^{1}H NMR (400 MHz, $CDCl_3$) δ 7.63 – 7.58 (m, 6H, H-6+H-12+H-11), 7.49 – 7.41 (m, 2H, H-1), 7.39 – 7.31 (m, 1H, H-2), 5.45 (s, 1H, H-17a), 5.06 (t, *J* = 0.9 Hz, 1H, H-17b), 4.21 (qd, *J* = 7.2, 0.8 Hz, 2H, H-21), 2.37 (dddd, *J* = 10.6, 6.0, 4.5, 1.2 Hz, 1H, H-15), 1.81 (dt, *J* = 8.4, 4.8 Hz, 1H, H-16), 1.51 (ddd, *J* = 9.1, 5.1, 4.2 Hz, 1H, H-17b), 1.31 (t, *J* = 7.1 Hz, 3H, H-22), 1.23 (ddd, *J* = 8.4, 6.7, 4.2 Hz, 1H, H-17a).

^{13}C{^{1}H} NMR (101 MHz, $CDCl_3$) δ = 173.7 (C-18), 145.8 (C-13), 140.8 (C-5+C-7), 139.3 (C-10), 128.9 (C-1), 127.5 (C-2), 127.2 (C-6), 127.1 (C-12), 126.5 (C-11), 111.3 (C-14), 60.9 (C-21), 26.2 (C-15), 22.4 (C-16), 15.5 (C-17), 14.5 (C-22).

IR (neat): ν (cm^{-1}): 2981, 1723, 1486, 1403, 1336, 1180, 1034, 901, 844, 774, 735, 690.

HRMS (ESI-TOF) m/z: $[M + H]^+$ Calcd for $C_{20}H_{21}O_2$ 293.1541; Found 293.1555.

mp: 53.4 – 55.4 °C.

Ethyl-2-(1-([1,1'-biphenyl]-4-yl)vinyl)cyclopropane-1-carboxylate (*cis*-**3aa**)

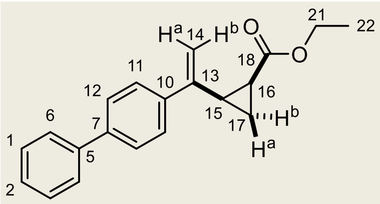

Cis-**3aa** was synthesized according to **GPI** on a 0.5 mmol scale using **1a** and **C4** (5 mol%, 7.42 mg) at 25 °C for 30 min. Purification by flash chromatography gave the desired compound as a colorless oil (56.5 mg, 0.19 mmol, 39%) (*cis*-**3aa** + *trans*-**3aa**: 76%, $dr_{(cis\text{-}\mathbf{3aa}/trans\text{-}\mathbf{3aa})}$ = 1.0 : 1.0). All spectroscopic data are in agreement with those reported in the literature.[10]

TLC: Rf = 0.42 (pentane/Et_2O = 98:2).

^{1}H NMR (400 MHz, $CDCl_3$) δ 7.65 – 7.53 (m, 6H, H-11+H-12+H-6), 7.47 – 7.40 (m, 2H, H-1), 7.38 – 7.32 (m, 1H, H-2), 5.70 (q, *J* = 0.9 Hz, 1H, H-14a), 5.26 (dd, *J* = 1.7, 0.9 Hz, 1H, H-14b), 3.91 (tq, *J* = 7.1, 3.4 Hz, 2H, H-21), 2.37 – 2.27 (m, 1H, H-15), 2.15 (ddd, *J* = 9.3, 7.8, 5.5 Hz, 1H, H-16), 1.60 (dt, *J* = 7.4, 5.2 Hz, 1H, H-17a), 1.30 (td, *J* = 8.1, 4.9 Hz, 1H, H-17b), 1.01 (t, *J* = 7.1 Hz, 3H, H-22).

^{13}C{^{1}H} NMR (101 MHz, $CDCl_3$) δ 171.0 (C-18), 141.8 (C-13), 140.9 (C-5), 140.4 (C-7), 139.5 (C-10), 128.9 (C-1), 127.4 (C-2), 127.1 (C-6), 127.0 (C-12), 126.4 (C-11), 115.1 (C-14), 60.4 (C-21), 25.8 (C-15), 21.5 (C-16), 14.3 (C-22), 11.4 (C-17).

IR (neat): ν (cm^{-1}): 2986, 1718, 1487, 1400, 1381, 1173, 1121, 1035, 906, 842, 772, 738, 695.

HRMS (ESI-TOF) m/z: [M + H]$^+$ Calcd for $C_{20}H_{21}O_2$ 293.1541; Found 293.1555.

mp: 50.0 – 51.0 °C.

Ethyl-2-(1-phenylvinyl)cyclopropane-1-carboxylate (*trans*-**3ba**)

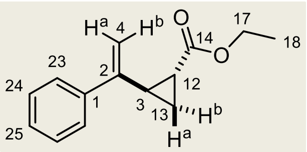

Trans-**3ba** was synthesized according to **GPI** on a 0.5 mmol scale using **1b** and **C$_4$** (5 mol%, 7.42 mg) at 25 °C for 30 min. Purification by flash chromatography gave a mixture of *trans* and *cis* diastereoisomers as a colorless oil (*trans*-**3ba** + *cis*-**3ba**: 79.4 mg, 0.36 mmol, 73%, *dr*$_{(trans\text{-}\mathbf{3ba}/cis\text{-}\mathbf{3ba})}$ = 1.0 : 1.0).

TLC: Rf = 0.38 (pentane/Et_2O = 95:5).

^{1}H NMR (300 MHz, $CDCl_3$) δ 7.55 – 7.45 (m, 2H, H-23+H-27), 7.39 – 7.28 (m, 3H, H2-4+H-25+H-26), 5.37 (s, 1H, H-4a), 5.02 (dd, *J* = 1.3, 0.7 Hz, 1H, H-4b), 4.19 (q, *J* = 7.1 Hz, 2H, H-17), 2.34 (m, 1H, H-3), 1.78 (ddd, *J* = 8.4, 5.1, 4.4 Hz, 1H, H-12), 1.48 (ddd, *J* = 9.2, 5.1, 4.2 Hz, 1H, H-13b), 1.29 (t, *J* = 7.1 Hz, 3H, H-18), 1.26 – 1.13 (m, 1H, H-13a).

^{13}C{^{1}H} NMR (75 MHz, $CDCl_3$) δ 173.7 (C-14), 146.4 (C-1), 140.4 (C-2), 128.5 (C-23+C-27), 128.0 (C-25), 126.2 (C-24+C-26), 111.3 (C-4), 60.8 (C-17), 26.2 (C-3), 22.4 (C-12), 15.4 (C-13), 14.5 (C-18).

IR (neat): ν (cm^{-1}): 2981, 1722, 1408, 1331, 1177, 1029, 897, 777, 703.

HRMS (ESI-TOF) m/z: [M + H]$^+$ Calcd for $C_{14}H_{17}O_2$ 217.1228; Found 217.1231.

Ethyl-2-(1-phenylvinyl)cyclopropane-1-carboxylate (*cis*-**3ba**)

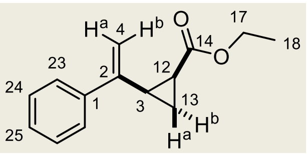

Cis-**3ba** was synthesized according to **GPI** on a 0.5 mmol scale using **1b** and **C$_4$** (5 mol%, 7.42 mg) at 25 °C for 30 min. Purification by flash chromatography gave a mixture of *trans* and *cis* diastereoisomers as a colorless oil (*cis*-**3ba** + *trans*-**3ba**: 79.4 mg, 0.36 mmol, 73%, *dr*$_{(cis\text{-}\mathbf{3ba}/trans\text{-}\mathbf{3ba})}$ = 1.0 : 1.0).

TLC: Rf = 0.35 (pentane/Et_2O = 95:5).

^{1}H NMR (400 MHz, CD_2Cl_2) δ 7.56 – 7.49 (m, 2H, H-23+H-27), 7.36 – 7.28 (m, 2H, H-24+H-26), 7.28 – 7.21 (m, 1H, H-25), 5.61 (q, *J* = 0.9 Hz, 1H, H-4a), 5.20 (t, *J* = 1.3 Hz, 1H, H-4b), 3.85 (qd, *J* = 7.1, 0.9 Hz, 2H, H-17), 2.34 – 2.22 (m, 1H, H-3), 2.09 (ddd, *J* = 9.3, 7.8, 5.5 Hz, 1H, H-12), 1.52 – 1.47 (m, 1H, H-13a), 1.26 (td, *J* = 8.1, 4.8 Hz, 2H, H-13b), 0.98 (t, *J* = 7.1 Hz, 3H, H-18).

^{13}C{^{1}H} NMR (101 MHz, CD_2Cl_2) δ 170.9 (C-14), 142.7 (C-1), 140.9 (C-2), 128.5 (C-23+C-27), 127.9 (C-25), 126.2 (C-24+C-26), 114.9 (C-4), 60.5 (C-17), 25.9 (C-3), 21.7 (C-12), 14.3 (C-18), 11.4 (C-13).

IR (neat): ν (cm^{-1}): 2979, 2930, 1728, 1445, 1399, 1380, 1261, 1174, 1097, 1033, 900, 777.

HRMS (ESI-TOF) m/z: [M + H]$^+$ Calcd for $C_{14}H_{17}O_2$ 217.1228; Found 217.1231.

Ethyl-2-(1-(4-chlorophenyl)vinyl)cyclopropane-1-carboxylate (*trans*-**3ca**)

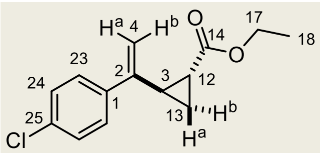

Trans-**3ca** was synthesized according to **GPI** on a 0.5 mmol scale using **1c** and **C4** (5 mol%, 7.42 mg) at 25 °C for 30 min. Purification by flash chromatography gave a mixture of *trans* and *cis* diastereoisomers as a colorless oil (*trans*-**3ca** + *cis*-**3cb**: 63.1 mg, 0.25 mmol, 50%, $dr_{(trans\text{-}\mathbf{3ca}/cis\text{-}\mathbf{3ca})}$ = 1.0 : 1.2).

TLC: Rf = 0.49 (pentane/CH_2Cl_2 = 80:20).

^{1}H NMR (400 MHz, $CDCl_3$) δ 7.43 (d, *J* = 8.6 Hz, 2H, H-23), 7.31 (d, *J* = 8.6 Hz, 2H, H-24), 5.36 (s, 1H, H-4a), 5.04 (d, *J* = 1.3 Hz, 1H, H-4b), 4.19 (q, *J* = 7.1 Hz, 2H, H-17), 2.32 – 2.22 (m, 1H, H-3), 1.80 – 1.71 (m, 1H, H-12), 1.47 (ddd, *J* = 9.2, 5.1, 4.2 Hz, 1H, H-13b), 1.29 (t, *J* = 7.1 Hz, 3H, H-18), 1.18 (ddd, *J* = 8.4, 6.6, 4.3 Hz, 1H, H-13a).

^{13}C{^{1}H} NMR (101 MHz, $CDCl_3$) δ = 173.6 (C-14), 145.3 (C-2), 138.8 (C-1), 133.8 (C-25), 128.6 (C-24), 127.4 (C-23), 111.8 (C-4), 60.9 (C-17), 26.0 (C-3), 22.3 (C-12), 15.4, (C-13), 14.5 (C-18).

IR (neat): ν (cm^{-1}): 2981, 1722, 1492, 1396, 1331, 1265, 1203, 1176, 1097, 1034, 1012, 899.

HRMS (ESI-TOF) m/z: [M + H]$^+$ Calcd for $C_{14}H_{16}ClO_2$ 251.0839; Found 251.0854.

Ethyl-2-(1-(4-chlorophenyl)vinyl)cyclopropane-1-carboxylate (*cis*-**3ca**)

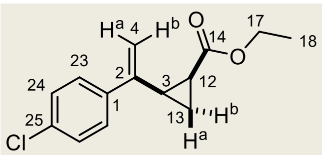

Cis-**3ca** was synthesized according to **GPI** on a 0.5 mmol scale using **1c** and **C4** (5 mol%, 7.42 mg) at 25 °C for 30 min. Purification by flash chromatography gave a mixture of *trans* and *cis* diastereoisomers as a colorless oil (*cis*-**3ca** + *trans*-**3ca**: 63.1 mg, 0.25 mmol, 50%, $dr_{(cis\text{-}\mathbf{3ca}/trans\text{-}\mathbf{3ca})}$ = 1.2 : 1.0).

TLC: Rf = 0.38 (pentane/CH_2Cl_2 = 80:20).

^{1}H NMR (400 MHz, $CDCl_3$) δ 7.46 (d, *J* = 8.6 Hz, 2H, H-23), 7.32 – 7.24 (m, 2H, H-24), 5.62 (q, *J* = 0.8 Hz, 1H, H-13a), 5.24 (dd, *J* = 1.7, 0.7 Hz, 1H, H-13b), 3.90 (dd, *J* = 7.1, 0.7 Hz, 2H, H-17), 2.23 (dq, *J* = 8.4, 0.8 Hz, 1H, H-3), 2.16 – 2.06 (m, 1H, H-12), 1.60 – 1.51 (m, 1H, H-13a), 1.33 – 1.19 (m, 1H, H-13b), 1.02 (t, *J* = 7.1 Hz, 3H, H-18).

^{13}C{^{1}H} NMR (101 MHz, $CDCl_3$) δ = 170.8 (C-14), 141.2 (C-2), 139.0 (C-1), 133.5 (C-25), 128.4 (C-24), 127.3 (C-23), 115.6 (C-4), 60.5 (C-17), 25.6 (C-3), 21.4 (C-12), 14.3 (C-18), 11.4 (C-13).

IR (neat): ν (cm^{-1}): 2981, 1727, 1492, 1396, 1380, 1174, 1095, 1012, 1036, 904, 834.

HRMS (ESI-TOF) m/z: [M + H]$^+$ Calcd for $C_{14}H_{16}ClO_2$ 251.0839; Found 251.0854.

Ethyl-2-(1-(o-tolyl)vinyl)cyclopropane-1-carboxylate (*trans*-**3da**)

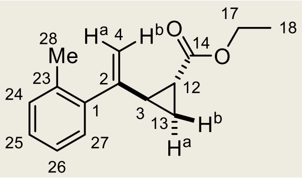

Trans-**3da** was synthesized according to **GPI** on a 0.5 mmol scale using **1d** and **C$_4$** (5 mol%, 7.42 mg) at 25 °C for 30 min. Purification by flash chromatography gave a mixture of *trans* and *cis* diastereoisomers as a colorless oil (*trans*-**3da** + *cis*-**3da**: 65.7 mg, 0.28 mmol, 57%, *dr*$_{(trans\text{-}\mathbf{3da}/cis\text{-}\mathbf{3da})}$ = 1.1 : 1.0).

TLC: Rf = 0.56 (pentane/CH_2Cl_2 = 50:50).

^{1}H NMR (400 MHz, $CDCl_3$) δ 7.21 – 7.10 (m, 3H, H-24+H-25+H-26), 7.09 – 7.05 (m, 1H, H-27), 5.33 (t, *J* = 1.5 Hz, 1H, H-4b), 5.15 (t, *J* = 1.2 Hz, 1H, H-4a), 4.07 (q, *J* = 7.1 Hz, 2H, H-17), 2.35 (s, 3H, H-28), 2.28 – 2.24 (m, 1H, H-3), 1.97 (ddd, *J* = 9.4, 7.8, 5.7 Hz, 1H, H-12), 1.49 (ddd, *J* = 7.7, 5.7, 5.0 Hz, 1H, H-13a), 1.20 (t, *J* = 7.2 Hz, 4H, 3-13b+H-18).

^{13}C{^{1}H} NMR (101 MHz, $CDCl_3$) δ 171.1 (C-14), 143.3 (C-2), 142.8 (C-1), 135.0 (C-23), 130.3 (C-24), 128.7 (C-27), 127.1 (C-26), 125.5 (C-25), 117.5 (C-4), 60.7 (C-17), 27.3 (C-3), 22.2 (C-12), 20.3 (C-28), 14.9 (C-13), 14.4 (C-18).

IR (neat): ν (cm^{-1}): 2981, 1724, 1446, 1401, 1381, 1263, 1175, 1095, 1035, 903, 765, 731.

HRMS (ESI-TOF) m/z: [M + H]$^+$ Calcd for $C_{15}H_{19}O_2$ 231.1385; Found 231.1386.

Ethyl-2-(1-(o-tolyl)vinyl)cyclopropane-1-carboxylate (*cis*-**3da**)

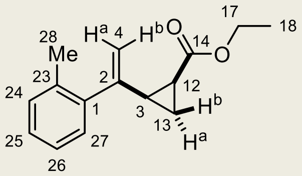

Cis-**3da** was synthesized according to **GPI** on a 0.5 mmol scale using **1d** and **C$_4$** (5 mol%, 7.42 mg) at 25 °C for 30 min. Purification by flash chromatography gave a mixture of *trans* and *cis* diastereoisomers as a colorless oil (*cis*-**3da** + *trans*-**3da**: 65.7 mg, 0.28 mmol, 57%, *dr*$_{(cis\text{-}\mathbf{3da}/trans\text{-}\mathbf{3da})}$ = 1.0 : 1.1).

TLC: Rf = 0.50 (pentane/CH_2Cl_2 = 50:50).

^{1}H NMR (400 MHz, $CDCl_3$) δ 7.21 – 7.11 (m, 3H, H-24+H-25+H-26), 7.02 (dt, *J* = 7.5, 1.2 Hz, 1H, H-27), 5.26 (dd, *J* = 1.6, 0.7 Hz, 1H, H-4a), 4.91 (d, *J* = 1.5 Hz, 1H, H-4b), 4.14 (qd, *J* =

7.1, 2.1 Hz, 2H, H-17), 2.29 (s, 3H, H-28), 2.24 – 2.16 (m, 1H, H-3), 1.61 (ddd, J = 8.5, 5.2, 4.2 Hz, 1H, H-12), 1.35 (ddd, J = 9.3, 5.2, 4.3 Hz, 1H, H-13a), 1.26 (t, J = 7.1 Hz, 3H, H-18), 0.92 (ddd, J = 8.5, 6.6, 4.3 Hz, 1H, H-13b).

^{13}C{^{1}H} NMR (101 MHz, $CDCl_3$) δ 173.7 (C-14), 147.8 (C-2), 140.1 (C-1), 135.7 (C-23), 130.1 (C-24), 129.1 (C-27), 127.5 (C-26), 125.6 (C-25), 114.0 (C-4), 60.5 (C-17), 28.0 (C-3), 21.6 (C-12), 20.0 (C-28), 14.4 (C-18), 11.7 (C-13).

IR (neat): ν (cm^{-1}): 2981, 1724, 1446, 1401, 1381, 1263, 1175, 1095, 1035, 903, 765, 731.

HRMS (ESI-TOF) m/z: [M + H]$^+$ Calcd for $C_{15}H_{19}O_2$ 231.1385; Found 231.1363.

Ethyl-2-(1-(2-fluorophenyl)vinyl)cyclopropane-1-carboxylate (*trans*-**3ea**)

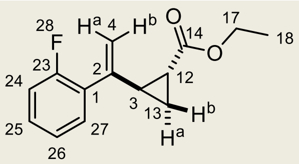

Trans-**3ea** was synthesized according to **GPI** on a 0.5 mmol scale using **1e** and **C_4** (5 mol%, 7.42 mg) at 25 °C for 30 min. Purification by flash chromatography gave *trans*-**3ea** as a colorless oil (38.6 mg, 0.16 mmol, 33%). The diastereomeric ratio was measured by 1H NMR of the crude mixture before purification: $dr_{(trans\text{-}\mathbf{3ea}/cis\text{-}\mathbf{3ea})}$ = 1.0 : 1.2.

TLC: Rf = 0.54 (pentane/ether = 98:2).

^{1}H NMR (500 MHz, $CDCl_3$) δ 7.30 – 7.23 (m, 2H, H-27+H-24), 7.10 (td, J = 7.6, 1.2 Hz, 1H, H-25), 7.05 (ddd, J = 10.7, 8.2, 1.2 Hz, 1H, H-24), 5.25 (d, J = 1.0 Hz, 1H, H-4a), 5.24 (q, J = 0.8 Hz, 1H, H-4b), 4.15 (q, J = 7.1 Hz, 2H, H-17), 2.37 – 2.29 (m, 1H, H-3), 1.76 – 1.69 (m, 1H, H-12), 1.41 (ddd, J = 9.1, 5.2, 4.4 Hz, 1H, H-13a), 1.27 (t, J = 7.1 Hz, 3H, H-18), 1.12 (dddd, J = 8.4, 6.6, 4.4, 0.7 Hz, 1H, H-13b).

^{13}C{^{1}H} NMR (126 MHz, $CDCl_3$) δ 173.9 (C-14), 160.2 (d, $^1J_{CF}$ = 248.1 Hz, C-23), 142.9 (C-2), 130.4 (d, $^3J_{CF}$ = 3.8 Hz, C-27), 129.6 (d, $^3J_{CF}$ = 8.1 Hz, C-25), 128.8 (d, $^2J_{CF}$ = 14.3 Hz, C-1), 124.3 (d, ^{4}JCF = 3.7 Hz, C-26), 116.3 (d, ^{1}JCF = 22.9 Hz, C-24), 115.7 (d, ^{4}JCF = 3.7 Hz, C-4), 61.0 (C-17), 27.4 (d, ^{4}JCF = 2.3 Hz, C-3), 22.6 (C-12), 15.7 (C-13), 14.7 (C-18).

^{19}F{^{1}H} NMR (282 MHz, $CDCl_3$) δ –114.3.

IR (neat): ν (cm^{-1}): 2983, 1724, 1488, 1450, 1381, 1176, 1035, 912, 843, 761.

HRMS (ESI-TOF) m/z: [M + H]$^+$ Calcd for $C_{14}H_{16}FO_2$ 235.1134; Found 235.1134.

Ethyl-2-(1-(2-fluorophenyl)vinyl)cyclopropane-1-carboxylate (*cis*-**3ea**)

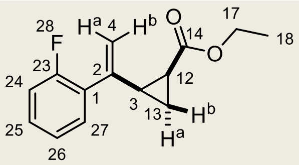

Cis-**3eb** was synthesized according to **GPI** on a 0.5 mmol scale using **1e** and **C_4** (5 mol%, 7.42 mg) at 25 °C for 30 min. Purification by flash chromatography gave *cis*-**3ea** as a colorless oil (44.8 mg, 0.19 mmol, 38%).

TLC: Rf = 0.45 (pentane/ether = 98:2).

^{1}H NMR (400 MHz, CD_2Cl_2) δ 7.31 (td, *J* = 7.8, 1.9 Hz, 1H, H-27), 7.24 (dddd, *J* = 8.1, 7.1, 5.0, 1.8 Hz, 1H, H-25), 7.10 (td, *J* = 7.5, 1.2 Hz, 1H, H-26), 7.04 (ddd, *J* = 11.3, 8.2, 1.3 Hz, 1H, H-24), 5.51 (dd, *J* = 1.7, 0.9 Hz, 1H, H-4a), 5.40 (dt, *J* = 2.1, 1.1 Hz, 1H, H-4b), 3.89 (q, *J* = 7.1 Hz, 2H, H-17), 2.44 – 2.32 (m, 1H, H-3), 1.96 (ddd, *J* = 9.3, 7.7, 5.5 Hz, 1H, H-12), 1.50 – 1.40 (m, 1H, H-13b), 1.29 – 1.17 (m, 1H, H-13a), 1.04 (t, *J* = 7.1 Hz, 3H, H-18).

^{13}C{^{1}H} NMR (101 MHz, CD_2Cl_2) δ 171.1(C-14), 160.3 (d, $^1J_{CF}$ = 247.7, Hz, C-23), 139.3 (d, $^3J_{CF}$ = 1.8 Hz, C-2), 130.2 (d, $^3J_{CF}$ = 4.2 Hz, C-27), 129.7 (d, $^2J_{CF}$ = 13.0 Hz, C-1), 129.2 (d, $^3J_{CF}$ = 8.4 Hz, C-25), 124.2 (d, $^4J_{CF}$ = 3.5 Hz, C-26), 119.5 (d, $^4J_{CF}$ = 4.5 Hz, C-4), 116.0 (d, $^2J_{CF}$ = 22.8 Hz, C-24), 60.5 (C-17), 26.5 (d, $^4J_{CF}$ = 3.4 Hz, C-3), 21.5 (C-3), 14.3 (C-18), 11.9 (C-13).

^{19}F{^{1}H} NMR (282 MHz, CD_2Cl_2) δ –115.0.

IR (neat): ν (cm^{-1}): 2987, 1722, 1488, 1450, 1381, 1178, 1033, 912, 843, 761.

HRMS (ESI-TOF) m/z: $[M + H]^+$ Calcd for $C_{14}H_{16}FO_2$ 235.1134; Found 235.1134.

Ethyl-2-(1-(2-(3-methylbut-2-en-1-yl)phenyl)vinyl)cyclopropane-1-carboxylate (*trans*-**3fa**)

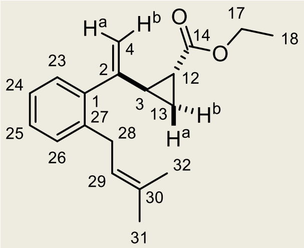

Trans-**3fa** was synthesized according to **GPI** on a 0.5 mmol scale using **1f** and **C$_4$** (5 mol%, 7.42 mg) at 25 °C for 30 min. Purification by flash chromatography gave a mixture of *trans* and *cis* diastereoisomers as a colorless oil (*trans*-**3fa** + *cis*-**3fa**: 109 mg, 0.38 mmol, 76%, $dr_{(trans\text{-}\mathbf{3fa}/cis\text{-}\mathbf{3fa})}$ = 1.0 : 1.3).

TLC: Rf = 0.37 (pentane/ CH_2Cl_2 = 80:20).

^{1}H NMR (400 MHz, $CDCl_3$) δ 7.25 – 7.17 (m, 2H, H-25+H-26), 7.13 (td, *J* = 7.0, 2.2 Hz, 1H, H-24), 7.00 (dd, *J* = 7.5, 1.3 Hz, 1H, H-23), 5.27 (d, *J* = 1.5 Hz, 1H, H-4a), 5.24 (dtt, *J* = 7.3, 2.9, 1.7 Hz, 1H, H-29), 4.92 (d, *J* = 1.6 Hz, 1H, H-4b), 4.12 (qd, *J* = 7.1, 2.4 Hz, 2H, H-17), 3.31 (d, *J* = 7.2 Hz, 2H, H-28), 2.26 (ddd, *J* = 9.1, 6.6, 4.3 Hz, 1H, H-3), 1.73 (d, *J* = 1.4 Hz, 3H, H-30), 1.70 (d, *J* = 1.4 Hz, 3H, H-32), 1.61 (ddd, *J* = 8.5, 5.2, 4.2 Hz, 1H, H-12), 1.33 (ddd, *J* = 9.3, 5.3, 4.3 Hz, 1HH-13b), 1.25 (t, *J* = 7.1 Hz, 3H, H-18), 0.92 (ddd, *J* = 8.5, 6.5, 4.3 Hz, 1H, H-13a).

^{13}C{^{1}H} NMR (101 MHz, $CDCl_3$) δ 173.7 (C-14), 147.4 (C-1), 139.5 (C-2), 132.4 (C-27), 129.2 (C-23), 129.1 (C-26), 127.7 (C-24), 125.5 (C-25), 123.5 (C-28), 114.4 (C-4), 60.7 (C-17), 31.8 (C-28), 28.3 (C-3), 25.9 (C-31), 21.4 (C-12), 18.1 (C-32), 14.8 (C-13), 14.4 (C-18).

IR (neat): ν (cm^{-1}): 2981, 2916, 1724, 1446, 1407, 1381, 1315, 1176, 1096, 1046, 897, 852, 766.

HRMS (ESI-TOF) m/z: $[M + Na]^+$ Calcd for $C_{19}H_{24}O_2Na$ 307.1674; Found 307.1647.

Ethyl-2-(1-(2-(3-methylbut-2-en-1-yl)phenyl)vinyl)cyclopropane-1-carboxylate (*cis*-**3fa**)

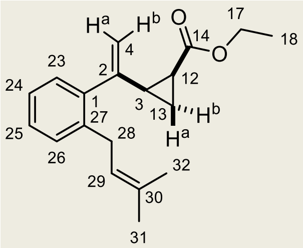

Cis-**3fa** was synthesized according to **GPI** on a 0.5 mmol scale using **1f** and **C4** (5 mol%, 7.42 mg) at 25 °C for 30 min. Purification by flash chromatography gave a mixture of *trans* and *cis* diastereoisomers as a colorless oil (*cis*-**3fa** + *trans*-**3fa**: 109 mg, 0.38 mmol, 76%, $dr_{(cis\text{-}\mathbf{3fa}/trans\text{-}\mathbf{3fa})}$ = 1.3 : 1.0).

TLC: Rf = 0.34 (pentane/ CH_2Cl_2 = 80:20).

^{1}H NMR (400 MHz, $CDCl_3$) δ 7.23 – 7.16 (m, 2H, H-25+H26), 7.12 (ddd, *J* = 7.6, 5.5, 3.2 Hz, 1H, H-24), 7.02 (dt, *J* = 7.7, 1.1 Hz, 1H, H23), 5.31 (t, *J* = 1.5 Hz, 1H, H-4b), 5.25 (ddq, *J* = 7.1, 5.7, 1.5 Hz, 1H, H-29), 5.17 – 5.10 (m, 1H, H-4a), 4.09 (q, *J* = 7.1 Hz, 2H, H-17), 3.37 (qt, *J* = 11.1, 5.8 Hz, 2H, H-28), 2.21 – 2.09 (m, 1H, H-3), 1.96 (ddd, *J* = 9.3, 7.7, 5.7 Hz, 1H, H-12), 1.73 (q, *J* = 1.4 Hz, 3H, H-31), 1.70 (d, *J* = 1.4 Hz, 3H, H-32), 1.51 (ddd, *J* = 7.7, 5.7, 5.0 Hz, 1H, H-13a), 1.22 (t, *J* = 7.1 Hz, 3H, H-18), 1.19 – 1.14 (m, 1H, H13b).

^{13}C{^{1}H} NMR (101 MHz, $CDCl_3$) δ 171.1 (C-14), 143.0 (C-2), 142.6 (C-27), 138.9 (C-1), 132.2 (C-30), 129.2 (C-26), 128.8 (C-23), 127.3 (C-25), 125.6 (C-24), 124.0 (C-29), 117.4 (C-4), 60.5 (C-17), 31.9 (C-28), 27.8 (C-3), 25.9 (C-31), 22.4 (C-12), 18.1 (C-32), 14.5 (C-18), 11.6 (C-13).

IR (neat): ν (cm^{-1}): 2979, 2912, 1728, 1445, 1380, 1175, 1097, 1041, 907, 764, 747.

HRMS (ESI-TOF) m/z: $[M + Na]^+$ Calcd for $C_{19}H_{24}O_2Na$ 307.1674; Found 307.1683.

Ethyl-2-(1-(1-tosyl-1H-indol-2-yl)vinyl)cyclopropane-1-carboxylate (*trans*-**3ga**)

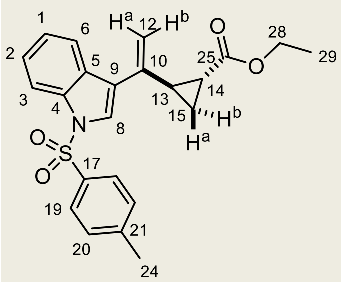

Trans-**3ga** was synthesized according to **GPI** on a 0.5 mmol scale using **1g** and **C4** (5 mol%, 7.42 mg) at 25 °C for 30 min. Purification by flash chromatography gave a mixture of *trans* and *cis* diastereoisomers. (*trans*-**3ga** + *cis*-**3ga**: 166 mg, 0.45 mmol, 81%, $dr_{(trans\text{-}\mathbf{3ga}/cis\text{-}\mathbf{3ga})}$ = 1.1 : 1.0).

TLC: Rf = 0.46 (pentane/ether = 90:10).

^{1}H NMR (400 MHz, None) δ 7.99 (d, *J* = 8.2 Hz, 1H, H-3), 7.82 – 7.72 (m, 3H, H-19+H-6), 7.69 (d, *J* = 1.3 Hz, 1H, H-8), 7.34 (ddd, *J* = 8.3, 7.2, 1.4 Hz, 1H, H-1), 7.27 (dd, *J* = 8.0, 4.0 Hz, 3H, H-20+H-2), 5.50 (s, 1H, H-12a), 5.22 (s, 1H, H12b), 4.20 (qdd, *J* = 7.1, 5.0, 1.2 Hz, 2H, H-28), 2.34 (s, 3H, H-24), 2.33 – 2.26 (m, 1H, H-13), 1.92 – 1.75 (m, 1H, H-14), 1.47 (dt, *J* = 8.0, 4.7 Hz, 1H, H-15b), 1.32 (td, *J* = 7.2, 1.2 Hz, 3H, H-29), 1.27 – 1.18 (m, 1H, H-15a).

^{13}C{^{1}H} NMR (101 MHz, CD_2Cl_2) δ 173.5 (C-25), 145.9 (C-17), 139.4 (C-10), 135.8 (C-4), 135.3 (C-21), 130.4 (C-20), 129.4 (C-5), 127.2 (C19), 125.2 (C-1), 124.4 (C-2), 124.0 (C-8), 123.4 (C-9), 121.4 (C-6), 114.1 (C-3), 112.7 (C-12), 61.2 (C-28), 27.2 (C-24), 22.2 (C-13), 21.7 (C-14), 15.0 (C-15), 14.5 (C-29).

IR (neat): ν (cm^{-1}): 3137, 2982, 1717, 1447, 1370, 1169, 1136, 1090, 1051, 963, 813, 718.
HRMS (ESI-TOF) m/z: $[M + H]^+$ Calcd for $C_{23}H_{24}NO_4S$ 410.1426; Found 410.1461.
mp: 93.9 – 95.4 °C.

Ethyl-2-(1-(1-tosyl-1H-indol-2-yl)vinyl)cyclopropane-1-carboxylate (*cis*-**3ga**)

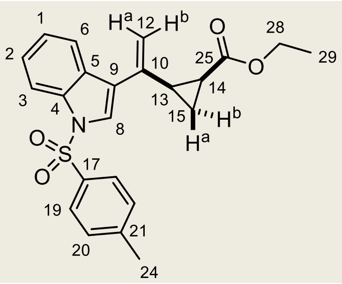

Cis-**3ga** was synthesized according to **GPI** on a 0.5 mmol scale using **1g** using **C**$_4$ (5 mol%, 7.42 mg) at 25 °C for 30 min. Purification by flash chromatography gave a mixture of *trans* and *cis* diastereoisomers. (*cis*-**3ga** + *trans*-**3ga**: 166 mg, 0.45 mmol, 81%, $dr_{(cis\text{-}\mathbf{3ga}/trans\text{-}\mathbf{3ga})}$ = 1.0 : 1.1).
TLC: Rf = 0.42 (pentane/ether = 90:10).
^{1}H NMR (400 MHz, CD_2Cl_2) δ 7.96 (dt, *J* = 8.3, 1.0 Hz, 1H, H-3), 7.84 – 7.74 (m, 3H, H-19+H-6), 7.70 (s, 1H, H-8), 7.32 (ddd, *J* = 8.3, 7.2, 1.4 Hz, 1H, H-1), 7.29 – 7.24 (m, 3H, H-20+H-2), 5.72 (d, *J* = 1.2 Hz, 1H, H-15a), 5.38 (t, *J* = 1.3 Hz, 1H, H-12b), 3.83 (qd, *J* = 7.1, 5.8 Hz, 2H, H-28), 2.36 – 2.28 (m, 4H, H-24+H-13), 2.14 (ddd, *J* = 9.3, 7.8, 5.6 Hz, 1H, H-14), 1.58 (ddd, *J* = 7.4, 5.6, 5.0 Hz, 1H, H-15a), 1.26 (td, *J* = 8.1, 4.9 Hz, 1H, H-15b), 0.93 (t, *J* = 7.1 Hz, 3H, H-29).
^{13}C{^{1}H} NMR (101 MHz, CD_2Cl_2) δ 171.0 (C-25), 145.8 (C-17), 135.8 (C-4), 135.6 (C-10), 135.4 (C-21), 130.3 (C-20), 129.2 (C-5), 127.2 (C-19), 125.1 (C-1), 124.5 (C-2), 123.9 (C-8), 123.7 (C-9), 121.6 (C-6), 116.0 (C-12), 113.9 (C-3), 60.6 (C-28), 26.8 (C-13), 21.7 (C-24), 21.4 (C-14), 14.3 (C-29), 11.1 (C-15).
IR (neat) ν (cm^{-1}) = 3135, 2983, 17171447, 1370, 1169, 1136, 1116, 1091, 1051, 813, 765, 748, 736, 667.
HRMS (ESI-TOF) m/z: $[M + H]^+$ Calcd for $C_{23}H_{24}NO_4S$ 410.1426; Found 410.1461.

Ethyl-2-(1-(6-methoxypyridin-3-yl)vinyl)cyclopropane-1-carboxylate (*trans*-**3ha**)

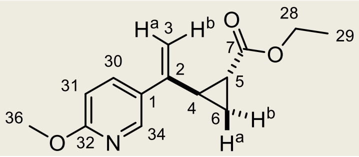

Trans-**3ha** was synthesized according to **GPI** on a 0.5 mmol scale using **1h** and **C**$_4$ (5 mol%, 7.42 mg) at 25 °C for 30 min. Purification by flash chromatography gave a mixture of *trans* and *cis* diastereoisomers as a colorless gel (*trans*-**3ha** + *cis*-**3ha**: 71 mg, 0.28 mmol, 57% $dr_{(trans\text{-}\mathbf{3ha}/cis\text{-}\mathbf{3ha})}$ = 1.0 : 1.2).
TLC: Rf = 0.53 (pentane/ether = 80:20).
^{1}H NMR (400 MHz, $CDCl_3$) δ 8.31 (dd, *J* = 2.5, 0.8 Hz, 1H, H-34), 7.70 (dd, *J* = 8.7, 2.6 Hz, 1H, H--30), 6.72 (dd, *J* = 8.6, 0.8 Hz, 1H, H-31), 5.30 (s, 1H, H-3a), 5.00 (d, *J* = 1.3 Hz, 1H, H-3b), 4.18 (dd, *J* = 7.2, 0.8 Hz, 2H, H-28), 3.94 (s, 3H, H-36), 2.32 – 2.22 (m, 1H, H-4), 1.76

(ddd, *J* = 8.4, 5.1, 4.4 Hz, 1H, H-5), 1.47 (d, *J* = 4.9 Hz, 1H, H-6b), 1.29 (t, *J* = 7.1 Hz, 3H, H-29), 1.22 – 1.15 (m, 1H, H-6a).

^{13}C{^{1}H} NMR (101 MHz, $CDCl_3$) δ 173.5 (C-7), 164.0 (C-32), 144.4 (C-34), 143.2 (C-2), 136.5 (C-30), 129.3 (C-1), 110.9 (C-3), 110.6 (C-31), 60.9 (C-28), 53.6 (C-36), 25.9 8 (C-4), 22.1 (C-5), 15.2 (C-6), 14.4 (C-29).

IR (neat): ν (cm^{-1}): 2983, 1722, 1600, 1493, 1367, 1286, 1176, 1023, 894, 831.

HRMS (ESI-TOF) m/z: [M + H]$^+$ Calcd for $C_{14}H_{18}NO_3$ 248.1287; Found 248.1283.

Ethyl-2-(1-(6-methoxypyridin-3-yl)vinyl)cyclopropane-1-carboxylate (*cis*-**3ha**)

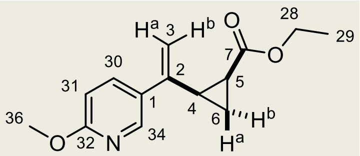

Cis-**3ha** was synthesized according to **GPI** on a 0.5 mmol scale using **1h** and **C$_4$** (5 mol%, 7.42 mg) at 25 °C for 30 min. Purification by flash chromatography gave a mixture of *trans* and *cis* diastereoisomers as a colorless gel (*cis*-**3ha** + *trans*-**3ha**: 71 mg, 0.28 mmol, 57% *dr*$_{(cis\text{-}\mathbf{3ha}/trans\text{-}\mathbf{3ha})}$ = 1.2 : 1.0).

TLC: Rf = 0.46 (pentane/ether = 80:20).

^{1}H NMR (400 MHz, $CDCl_3$) δ 8.35 (dd, *J* = 2.6, 0.7 Hz, 1H, H-34), 7.73 (dd, *J* = 8.7, 2.6 Hz, 1H, H-30), 6.69 (dd, *J* = 8.7, 0.7 Hz, 1H, H-31), 5.56 (q, *J* = 0.8 Hz, 1H, H-3a), 5.30 – 5.06 (m, 1H, H-3b), 3.93 (s, 5H, H-36+H-28), 2.28 – 2.17 (m, 1H, H-4), 2.10 (ddd, *J* = 9.3, 7.8, 5.6 Hz, 1H, H-5), 1.61 – 1.51 (m, 1H, H-6a), 1.32 – 1.19 (m, 1H, H-6b), 1.03 (t, *J* = 7.1 Hz, 3H, H-29).

^{13}C{^{1}H} NMR (101 MHz, $CDCl_3$) δ 170.8 (C-7), 163.8 (C-32), 144.3 (C-34), 139.1 (C-2), 136.3 (C-30), 129.5 (C-1), 114.3 (C-3), 110.4 (C-31), 60.5 (C-28), 53.6 (C-36), 25.4 (C-4), 21.3 (C-5), 14.3 (C-29), 11.3 (C-6).

IR (neat): ν (cm^{-1}): 2981, 1726, 1600, 1493, 1365, 1287, 1176, 1024, 900, 832.

HRMS (ESI-TOF) m/z: [M + H]$^+$ Calcd for $C_{14}H_{18}NO_3$ 248.1287; Found 248.1283.

Ethyl-2-(1-(thiophen-3-yl)vinyl)cyclopropane-1-carboxylate (*trans*-**3ia**)

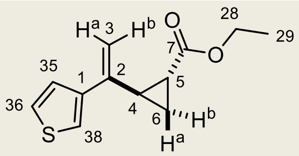

Trans-**3ia** was synthesized according to **GPI** on a 0.5 mmol scale using **1i** and **C$_4$** (5 mol%, 7.42 mg) at 25 °C for 30 min. Purification by flash chromatography gave a mixture of *trans* and *cis* diastereoisomers as a pale yellow oil (*trans*-**3ia** + *cis*-**3ib**: 38.7 mg, 0.17 mmol, 38%, *dr*$_{(trans\text{-}\mathbf{3ia}/cis\text{-}\mathbf{3ia})}$ = 1.0 : 1.3).

TLC: Rf = 0.38 (pentane/ CH_2Cl_2 = 80:20).

^{1}H NMR (400 MHz, $CDCl_3$) δ 7.34 (dd, *J* = 2.9, 1.4 Hz, 1H, H-38), 7.29 (dd, *J* = 5.0, 2.9 Hz, 1H, H-36), 7.27 – 7.24 (m, 1H, H-35), 5.37 (s, 1H, H-3a), 5.05 – 4.85 (m, 1H, H-3b), 4.19 (q, *J* = 7.1 Hz, 2H, H-28), 2.34 (ddt, *J* = 9.7, 5.2, 2.5 Hz, 1H, H-4), 1.76 (dt, *J* = 8.4, 4.8 Hz, 1H,

H-5), 1.44 (ddd, J = 9.2, 5.1, 4.2 Hz, 1H, H-6b), 1.30 (t, J = 7.1 Hz, 3H, H-29), 1.20 (ddd, J = 8.4, 6.7, 4.2 Hz, 1H, H-6a).

^{13}C{^{1}H} NMR (101 MHz, $CDCl_3$) δ 173.8 (C-7), 142.1 (C-1), 141.1 (C-2), 125.8 (C-35), 125.6 (C-36), 121.2 (C-38), 110.3 (C-3), 60.9 (C-28), 26.1 (C-4), 21.8 (C-5), 14.9 (C-6), 14.5 (C-29).

IR (neat): ν(cm^{-1}): 3110, 2925, 1720, 1624, 1446, 1383, 1324, 1264, 1176, 1094, 1054, 1033, 866, 789, 733.

HRMS (ESI-TOF) m/z: $[M + H]^+$ Calcd for $C_{12}H_{15}O_2S$ 223.0793; Found 223.0607.

Ethyl-2-(1-(thiophen-3-yl)vinyl)cyclopropane-1-carboxylate (*cis*-**3ia**)

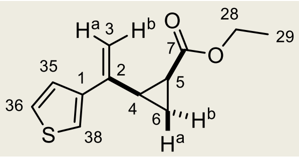

Cis-**3ia** was synthesized according to **GPI** on a 0.5 mmol scale using **1i** and **C₄** (5 mol%, 7.42 mg) as a catalyst at 25 °C for 30 min. Purification by flash chromatography gave a mixture of *trans* and *cis* diastereoisomers as a pale yellow oil (*cis*-**3ia** + *trans*-**3ia**: 38.7 mg, 0.17 mmol, 38%, $dr_{(cis\text{-}\mathbf{3ia}/trans\text{-}\mathbf{3ia})}$ = 1.3 : 1.0).

TLC: Rf = 0.35 (pentane/ CH_2Cl_2 = 80:20).

^{1}H NMR (400 MHz, $CDCl_3$) δ 7.34 (dd, J = 2.9, 1.4 Hz, 1H, H-38), 7.28 – 7.25 (m, 1H, H-36), 7.23 (dd, J = 5.1, 2.9 Hz, 1H, H-35), 5.60 (q, J = 0.9 Hz, 1H, H-3a), 5.33 – 5.09 (m, 1H, H-3b), 3.92 (qq, J = 7.0, 3.7 Hz, 2H, H-28), 2.34 – 2.20 (m, 1H, H-4), 2.08 (ddd, J = 9.4, 7.8, 5.5 Hz, 1H, H-5), 1.62 – 1.50 (m, 1H, H-6a), 1.25 (td, J = 8.1, 4.9 Hz, 1H, H-6b), 1.01 (t, J = 7.1 Hz, 3H, H-29).

^{13}C{^{1}H} NMR (101 MHz, $CDCl_3$) δ 171.0 (C-7), 142.4 (C-1), 137.5 (C-2), 125.6 (C-35), 125.4 (C-36), 121.0 (C-38), 114.0 (C-3), 60.4 (C-28), 25.9 (C-4), 20.8 (C-5), 14.2 (C-29), 11.2 (C-6).

IR (neat): ν(cm^{-1}): 3106, 2980, 2925, 1725, 1626, 1399, 1380, 1263, 1179, 1162, 1095, 1035, 895, 867, 836, 789, 713.

HRMS (ESI-TOF) m/z: $[M + H]^+$ Calcd for $C_{12}H_{15}O_2S$ 223.0793; Found 223.0790.

Ethyl-2-(4-phenylbut-1-en-2-yl)cyclopropane-1-carboxylate (*trans*-**3ja**)

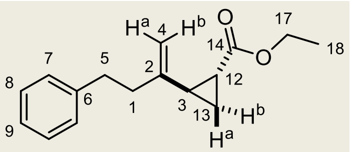

Trans-**3ja** was synthesized according to **GPI** on a 0.5 mmol scale using **1j** and **C₄** (5 mol%, 7.42 mg) at 25 °C for 30 min. Purification by flash chromatography gave *trans*-**3ja** as a colorless oil (42.8 mg, 0.17 mmol, 36%). The diastereomeric ratio was measured by ^{1}H NMR of the crude mixture before purification: $dr_{(trans\text{-}\mathbf{3ja}/cis\text{-}\mathbf{3ja})}$ = 1.0 : 1.4.

TLC: Rf = 0.60 (pentane/ CH_2Cl_2 = 50:50).

^{1}H NMR (400 MHz, CD_2Cl_2) δ 7.28 (t, J = 7.5 Hz, 2H, H-8), 7.23 – 7.14 (m, 3H, H-7+H-9), 4.77 (d, J = 1.4 Hz, 1H, H-4a), 4.74 (s, 1H, H-4b), 4.11 (q, J = 7.1 Hz, 2H, H-17), 2.89 – 2.67 (m, 2H, H-5), 2.36 (dd, J = 9.0, 7.3 Hz, 2H, H-1), 1.94 (ddd, J = 9.9, 6.6, 4.3 Hz, 1H, H-3), 1.64

(dt, J = 8.5, 4.8 Hz, 1H, H-12), 1.41 – 1.16 (m, 4H, H-18+H-13b), 1.07 (ddd, J = 8.4, 6.7, 4.2 Hz, 1H, H-13a).

^{13}C{^{1}H} NMR (101 MHz, CD_2Cl_2) δ 173.8 (C-14), 147.7 (C-2), 142.4 (C-6), 128.7 (C-8), 128.7 (C-7), 126.2 (C-9), 109.1 (C-4), 60.9 (C-17), 38.0 (C-1), 34.7 (C-5), 27.2 (C-3), 21.9 (C-12), 14.9 (C-13), 14.5 (C-18).

IR (neat): ν (cm^{-1}): 2981, 1722, 1454, 1407, 1327, 1264, 1176, 1055, 1033, 889, 859, 748, 699.

HRMS (ESI-TOF) m/z: $[M + Na]^+$ Calcd for $C_{16}H_{20}O_2Na$ 267.1361; Found 267.1339.

Ethyl-2-(4-phenylbut-1-en-2-yl)cyclopropane-1-carboxylate (*cis*-**3ja**)

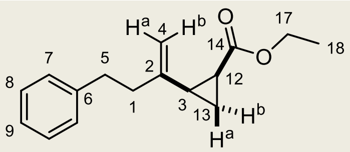

Cis-**3ja** was synthesized according to **GPI** on a 0.5 mmol scale using **1j** and **C$_4$** (5 mol%, 7.42 mg) as a catalyst at 25 °C for 30 min. Purification by flash chromatography gave *cis*-**3ja** as a colorless oil (52.5 mg, 0.21 mmol, 43).

TLC: Rf = 0.53 (pentane/ CH_2Cl_2 = 50:50).

^{1}H NMR (400 MHz, CD_2Cl_2) δ 7.31 – 7.24 (m, 2H, H-8), 7.23 – 7.14 (m, 3H, H-7+H-9), 4.97 (p, J = 1.4 Hz, 1H, H-4a), 4.91 – 4.86 (m, 1H, H-4b), 4.05 (q, J = 7.1 Hz, 2H, H-17), 2.77 (td, J = 7.8, 4.9 Hz, 2H, H-5), 2.50 – 2.15 (m, 2H, H-1), 2.09 – 1.79 (m, 2H, H-3+H-12), 1.38 (ddd, J = 7.2, 5.9, 4.7 Hz, 1H, H-13a), 1.20 (t, J = 7.1 Hz, 3H, H-18), 1.10 (td, J = 8.1, 4.7 Hz, 1H, H-13b).

^{13}C{^{1}H} NMR (101 MHz, CD_2Cl_2) δ 171.4 (C-14), 143.9 (C-2), 142.7 (C-6), 128.7 (C-8), 128.7), 126.1 (C-9), 112.9 (C-4), 60.6 (C-17), 39.2 (C-1), 34.6 (C-5), 26.6 (C-3), 20.9 (C-12), 14.6 (C-18), 11.0 (C-13).

IR (neat): ν (cm^{-1}): 2981, 1728, 1454, 1396, 1380, 1162, 1113, 1097, 1040, 897, 748, 699.

HRMS (ESI-TOF) m/z: $[M + Na]^+$ Calcd for $C_{16}H_{20}O_2Na$ 267.1361; Found 267.1339.

Ethyl-2-(4,8-dimethylnona-1,7-dien-2-yl)cyclopropane-1-carboxylate (*trans*-**3ka**)

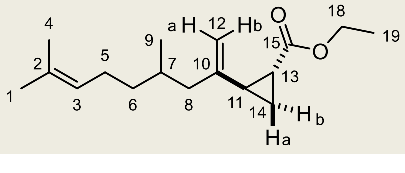

Trans-**3ka** was synthesized according to **GPI** on a 0.5 mmol scale using **1k** and **C$_4$** (5 mol%, 7.42 mg) at 25 °C for 30 min. Purification by flash chromatography gave a mixture of *trans* and *cis* diastereoisomers as a colorless oil (*trans*-**3ka** + *cis*-**3ka**: 84.5 mg, 0.32 mmol, 64%, $dr_{(trans\text{-}\mathbf{3ka}/cis\text{-}\mathbf{3ka})}$ = 1.0 : 1.2).

TLC: Rf = 0.67 (pentane/ ether = 98:2).

^{1}H NMR (400 MHz, $CDCl_3$) δ 5.16 – 5.05 (m, 1H, H-3), 4.69 (d, J = 1.2 Hz, 2H, H-12a+H-12b), 4.14 (d, J = 7.1 Hz, 2H, , H-18), 2.14 – 2.05 (m, 1H, H-8), 2.04 – 1.91 (m, 2H, H-5+H6), 1.89 – 1.81 (m, 2H, H-11+H-8), 1.68 (t, J = 1.3 Hz, 3H, H-1), 1.67 – 1.61 (m, 2H, H-13+H-7), 1.61

(d, *J* = 1.4 Hz, 3H, H-4), 1.41 – 1.31 (m, 1H, H-5+H-6), 1.27 (t, *J* = 7.1 Hz, 4H, H-14b+H-19), 1.19 – 1.08 (m, 1H, H-5), 1.04 (dtd, *J* = 8.4, 6.9, 4.1 Hz, 1H, H-14a), 0.87 (dd, *J* = 6.6, 1.6 Hz, 3H, H-9).

^{13}C{^{1}H} NMR (101 MHz, $CDCl_3$) δ 174.0 (C-15), 174.0 (C-15), 146.7 (C-10), 146.7 (C-10), 131.4 (C-2), 131.4 (C-2), 124.9 (C3), 124.9 (C-3), 109.7 (C-12), 109.7 (C-12), 60.7 (C-18), 44.8 (C-8), 44.7 (C-8), 37.0 (C-5), 30.8 (C-7), 30.7 (C-7), 26.9 (C-11), 26.8 (C-11), 25.9 (C-1), 25.7 (C-6), 25.7 (C-6), 21.9 (C-13), 21.8 (C-13), 19.6 (C-9), 19.6 (C-9), 17.8 (C-4), 15.4 (C-14), 15.1(C-14), 14.4 (C-19).

IR (neat): ν (cm^{-1}): 2966, 2915, 1727, 1644, 1448, 1331, 1263, 1175, 1036, 935, 888, 789.

HRMS (ESI-TOF) m/z: $[M + Na]^+$ Calcd for $C_{17}H_{28}O_2Na$ 287.1987; Found 287.1971.

Ethyl-2-(4,8-dimethylnona-1,7-dien-2-yl)cyclopropane-1-carboxylate (*cis*-**3ka**)

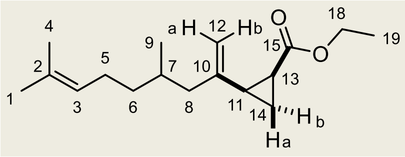

Cis-**3ka** was synthesized according to **GPI** on a 0.5 mmol scale using **1k** and **C$_4$** (5 mol%, 7.42 mg) at 25 °C for 30 min. Purification by flash chromatography gave a mixture of *trans* and *cis* diastereoisomers as a colorless oil (*cis*-**3ka** + *trans*-**3ka**: 84.5 mg, 0.32 mmol, 64%, *dr*$_{(cis\text{-}\mathbf{3ka}/trans\text{-}\mathbf{3ka})}$ = 1.2 : 1.0).

TLC: Rf = 0.60 (pentane/ ether = 98:2).

^{1}H NMR (400 MHz, $CDCl_3$) δ 5.10 (ddq, *J* = 7.1, 4.6, 1.5 Hz, 1H, H-3), 4.93 – 4.87 (m, 2H, H-12a+12b), 4.15 – 4.00 (m, 2H, H-18), 2.11 – 1.92 (m, 3H, H-5+H-8), 1.90 – 1.77 (m, 3H, H-8+H,+H-11+H-13), 1.68 (t, *J* = 1.3 Hz, 4H, H-1+H-7), 1.60 (d, *J* = 1.9 Hz, 3H, H-4), 1.44 – 1.30 (m, 2H, H+14a+H-6), 1.22 (td, *J* = 7.1, 1.2 Hz, 3H, H-19), 1.18 – 1.03 (m, 2H, H+14b+H-6), 0.85 (t, *J* = 6.1 Hz, 3H, H-9).

^{13}C{^{1}H} NMR (101 MHz, $CDCl_3$) δ 171.4 (C-15), 142.6 (C-10), 142.3 (C-10), 131.3 (C-2), 125.0 (C3), 124.9 (C-3), 114.1 (C-12), 114.0 (C-12), 60.4 (C-18), 60.4 (C-18), 45.4 (C-8), 45.0 (C-8), 37.5 (C-6), 36.9 (C-6), 30.6 (C-7), 30.5 (C-7), 26.6 (C-11), 26.3 (C-11), 25.9 (C-1), 25.7 (C-5), 25.7 (C-5), 21.0 (C-13), 20.5 (C-13), 19.8 (C-9), 19.5 (C-9), 17.8 (C-4), 14.5 (C-19), 11.3 (C-14), 11.1 (C-14).

IR (neat): ν (cm^{-1}): 2965, 2916, 1732, 1445, 1379, 1162, 1114, 1098, 984, 896, 829.

HRMS (ESI-TOF) m/z: $[M + Na]^+$ Calcd for $C_{17}H_{28}O_2$ Na 287.1987; Found 287.1971.

***t*-Butyl-3-(1-(2-(ethoxycarbonyl)cyclopropyl)vinyl)piperidine-1-carboxylate** (*trans*-**3la**)

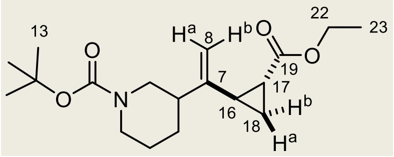

Trans-**3la** was synthesized according to **GPI** on a 0.5 mmol scale using **1l** using **C$_4$** (5 mol%, 7.42 mg) at 25 °C for 30 min. Purification by flash chromatography gave a mixture of *trans*

and *cis* diastereoisomers as a colorless oil (*trans*-**3la** + *cis*-**3la**: 128 mg, 0.395 mmol, 79%, $dr_{(trans\text{-}\mathbf{3la}/cis\text{-}\mathbf{3la})}$ = 1.0 : 1.0).

TLC: Rf = 0.68 (CH_2Cl_2/EtOAc = 96:4).

^{1}H NMR (500 MHz, $CDCl_3$) δ 4.76 (d, *J* = 4.3 Hz, 1H, H-8a), 4.72 (d, *J* = 7.7 Hz, 1H, H-8b), 4.14 (d, *J* = 7.2 Hz, 3H, H-22+*Alkyl-H*), 4.04 (s, 1H, *Alkyl-H*), 2.60 (s, 2H, *Alkyl-H*), 2.09 (d, *J* = 11.9 Hz, 1H, *Alkyl-H*), 1.92 (d, *J* = 2.1 Hz, 2H, *Alkyl-H*), 1.66 (ddt, *J* = 19.4, 9.5, 4.0 Hz, 2H, *Alkyl-H*), 1.46 (d, *J* = 1.8 Hz, 9H, H-13), 1.34 – 1.29 (m, 1H, H-18b), 1.26 (s, 3H, H-23), 1.08 – 0.99 (m, 1H, H-18a).

^{13}C{^{1}H} NMR (126 MHz, $CDCl_3$) δ 173.5, 173.5, 155.0, 149.7, 149.6, 108.6, 108.3, 79.5, 60.7, 49.5, 44.6, 42.9, 42.7, 30.6, 30.6, 28.7, 25.9, 25.9, 25.5, 22.0, 22.0, 15.1, 15.1, 14.4.

IR (neat): ν (cm^{-1}): 2979, 2932, 2862, 1724, 1691, 1417, 1365, 1331, 1263, 1240, 1167, 1148, 1033, 885, 885, 768.

HRMS (ESI-TOF) m/z: $[M + Na]^+$ Calcd for $C_{18}H_{29}NO_4Na$ 346.1994; Found 346.1984.

***t*-Butyl 3-(1-(2-(ethoxycarbonyl)cyclopropyl)vinyl)piperidine-1-carboxylate** (*cis*-**3la**)

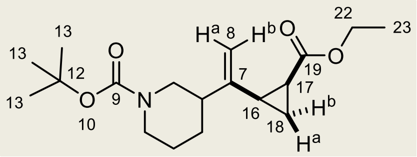

Cis-**3la** was synthesized according to **GPI** on a 0.5 mmol scale using **1l** and **C$_4$** (5 mol%, 7.42 mg) at 25 °C for 30 min. Purification by flash chromatography gave a mixture of *trans* and *cis* diastereoisomers as a colorless oil (*cis*-**3la** + *trans*-**3la**: 128 mg, 0.395 mmol, 79%, $dr_{(cis\text{-}\mathbf{3la}/trans\text{-}\mathbf{3la})}$ = 1.0 : 1.0).

TLC: Rf = 0.62 (CH_2Cl_2/EtOAc = 96:4).

^{1}H NMR (500 MHz, $CDCl_3$) δ 4.98 (d, *J* = 1.2 Hz, 1H, H-8a), 4.93 (s, 1H, H-8b), 4.24 – 4.12 (m, 1H, *Alkyl-H*), 4.08 (d, *J* = 7.1 Hz, 3H, H-22- *Alkyl-H*), 2.65 (d, *J* = 12.7 Hz, 1H, *Alkyl-H*), 2.53 – 2.43 (m, 1H, *Alkyl-H*), 2.08 (m, 1H, H- *Alkyl-H*), 1.98 – 1.84 (m, 3H, H—16+H-17+ *Alkyl-H*), 1.67 (tq, *J* = 9.3, 3.2 Hz, 2H, *Alkyl-H*), 1.47 (s, 9H, H-13+H-18a), 1.41 – 1.33 (m, 1H, *Alkyl-H*), 1.22 (s, 3H, H-23), 1.10 (d, *J* = 5.0 Hz, 1H, H-18b).

^{13}C{^{1}H} NMR (101 MHz, $CDCl_3$) δ 171.2, 154.9, 154.9, 144.7, 144.5, 112.5, 79.5, 79.4, 60.5, 60.4, 43.0, 30.1, 29.9, 28.6, 25.8, 25.5, 21.6, 21.2, 14.5, 10.7, 10.5.

IR (neat): ν (cm^{-1}): 2977, 2934, 2855, 1727, 1689, 1418, 1365, 1263, 1240, 1164, 1149, 1035, 857, 767.

HRMS (ESI-TOF) m/z: $[M + Na]^+$ Calcd for $C_{18}H_{29}NO_4Na$ 346.1994; Found 346.1984.

Ethyl 2-(1-((3r,5r,7r)-adamantan-1-yl)vinyl)cyclopropane-1-carboxylate (*trans*-**3ma**)

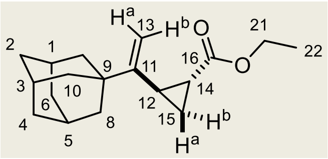

Trans-**3ma** was synthesized according to **GPI** on a 0.5 mmol scale using **1m** and **C$_4$** (5 mol%, 7.42 mg) at 25 °C for 30 min. Purification by flash chromatography gave *trans*-**3ma** as a

colorless oil (58.0 mg, 0.211 mmol, 42%). The diastereomeric ratio was measured by ^{1}H NMR of the crude mixture before purification: $dr_{(trans\text{-}\mathbf{3ma}/cis\text{-}\mathbf{3ma})}$ = 1.0 : 1.0.

TLC: Rf = 0.68 (pentane/ ether = 98:2).

^{1}H NMR (400 MHz, $CDCl_3$) δ 4.70 (s, 1H,H-13a), 4.55 (d, *J* = 1.0 Hz, 1H, H-13b), 4.16 (dd, *J* = 7.1, 4.0 Hz, 2H, H-21), 2.05 – 1.99 (m, 4H, H-1+H-3+H-5+H-12), 1.81 – 1.60 (m, 12H, H-2+H-4+H-6+H-7+H-8+H-10), 1.56 (dt, *J* = 8.2, 4.8 Hz, 1H, H-14), 1.27 (t, *J* = 7.1 Hz, 4H, H-15b+H-22), 1.09 – 0.99 (m, 1H, H-15a).

^{13}C{^{1}H} NMR (101 MHz, $CDCl_3$) δ 174.1 (C-16), 157.0 (C-11), 104.9 (C-13), 60.6 (C21), 41.6 (C-7+C-8+C-10), 38.4 (C-9), 37.1 (C-2+C-4+C-6), 28.8 (C-1+C-3+C-5), 23.5 8 (C-12), 23.0 (C14), 15.4 (C-15), 14.5 (C-22).

IR (neat): ν (cm^{-1}): 2902, 2849, 1725, 1631, 1449, 1406, 1331, 1177.

HRMS (ESI-TOF) m/z: [M + H]$^+$ Calcd for $C_{18}H_{27}O_2$ 275.2011; Found 275.2002.

Ethyl 2-(1-((3r,5r,7r)-adamantan-1-yl)vinyl)cyclopropane-1-carboxylate (*cis*-**3ma**)

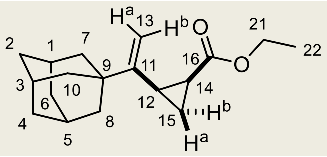

Cis-**3ma** was synthesized according to **GPI** on a 0.5 mmol scale using **1m** and **C4** (5 mol%, 7.42 mg) at 25 °C for 30 min. Purification by flash chromatography gave *cis*-**3ma** as a colorless oil (46.9 mg, 0.17 mmol, 34%).

TLC: Rf = 0.60 (pentane/ ether = 98:2).

^{1}H NMR (400 MHz, $CDCl_3$) δ 4.99 (s, 1H, H-13a), 4.82 (s, 1H, H-13b), 4.05 (q, *J* = 7.1 Hz, 2H, H-21), 2.05 – 1.95 (m, 4H, H-1+H-3+H-5+H-12), 1.91 (ddd, *J* = 9.3, 7.8, 5.5 Hz, 1H, H-14), 1.69 (dd, *J* = 13.3, 2.8 Hz, 12H, H-2+H-4+H-6+H-7+H-8+H-10), 1.46 (dt, *J* = 7.9, 5.2 Hz, 1H, H-15a), 1.21 (t, *J* = 7.1 Hz, 3H, H-22), 1.07 (td, *J* = 8.1, 4.9 Hz, 1H, H-15b).

^{13}C{^{1}H} NMR (101 MHz, $CDCl_3$) δ 171.3 (C-16), 150.9 (C-11), 109.8 (C-13), 60.2 (C21), 41.7 (C-7+C-8+C-10), 38.0 (C-9), 37.1 (C-2+C-4+C-6), 28.9 (C-1+C-3+C-5), 23.6 (C-12), 22.6 (14), 14.5 (C-22), 10.0 (C-15).

IR (neat): ν (cm^{-1}): 2981, 2905, 2847, 1726, 1631, 1444, 1401, 1383, 1173, 1112, 1037, 897.

HRMS (ESI-TOF) m/z: [M + H]$^+$ Calcd for $C_{18}H_{27}O_2$ 275.2011; Found 275.2036.

Ethyl (*Z*)-2-(nonadeca-1,10-dien-2-yl)cyclopropane-1-carboxylate (*trans*-**3na**)

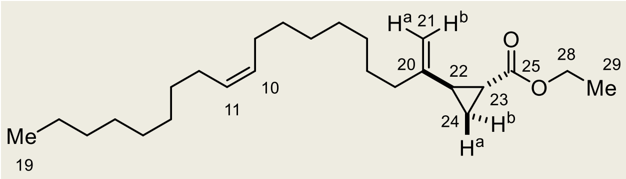

Trans-**3na** was synthesized according to **GPI** on a 0.5 mmol scale using **1n** and **C4** (5 mol%, 7.42 mg) at 25 °C for 30 min. Purification by flash chromatography gave a mixture of *trans* and *cis* diastereoisomers as a colorless oil (*trans*-**3na** + *cis*-**3nb**: 126 mg, 0.33 mmol, 67%, $dr_{(trans\text{-}\mathbf{3na}/cis\text{-}\mathbf{3na})}$ = 1.0 : 1.2).

TLC: Rf = 0.60 (pentane/ ether = 95:5).

^{1}H NMR (400 MHz, $CDCl_3$) δ 5.35 (s, 2H, H-10+H11), 4.71 (d, *J* = 1.4 Hz, 1H, H-21a), 4.68 (s, 1H, H-21b), 4.14 (d, *J* = 7.1 Hz, 2H, H-28), 2.01 (d, *J* = 6.9 Hz, 6H, *alkyl*-H), 1.95 – 1.87 (m, 1H, H-23), 1.63 (d, *J* = 8.3 Hz, 1H, H-22), 1.46 (d, *J* = 1.8 Hz, 3H, *alkyl*-H), 1.27 (s, 25H, *alkyl*-H), 1.05 (s, 1H, H-24a), 0.88 (s, 3H, H-19).

^{13}C{^{1}H} NMR (101 MHz, $CDCl_3$) δ 174.0 (C-25), 148.0 (C-19), 130.1 (C-11), 130.0 (C-10), 108.4 (C-21), 60.7 (C-28), 36.0 (*alkyl-C*), 32.1 (*alkyl-C*), 29.9 (*alkyl-C*), 29.9 (*alkyl-C*), 29.7 (*alkyl-C*), 29.6 (*alkyl-C*), 29.5 (*alkyl-C*), 29.4 (*alkyl-C*), 28.0 (*alkyl-C*), 27.4 (*alkyl-C*), 27.3 (*alkyl-C*), 27.1 (C-23), 22.8, 21.7 (C-22), 14.9 (C-24), 14.4 (*alkyl-C*), 14.3 (*alkyl-C*).

IR (neat): ν (cm^{-1}): 2924, 2854, 1728, 1644, 1464, 1331, 1262, 1176, 1038, 888, 859, 725.

HRMS (ESI-TOF) m/z: $[M + Na]^+$ Calcd for $C_{25}H_{44}O_2Na$ 399.3239; Found 399.3198.

Ethyl (*Z*)-2-(nonadeca-1,10-dien-2-yl)cyclopropane-1-carboxylate (*cis*-**3na**)

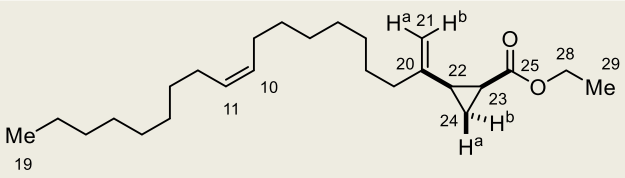

Cis-**3na** was synthesized according to **GPI** on a 0.5 mmol scale using **1n** and **C$_4$** (5 mol%, 7.42 mg) at 25 °C for 30 min. Purification by flash chromatography gave a mixture of *trans* and *cis* diastereoisomers as a colorless oil (*cis*-**3na** + *trans*-**3na**: 126 mg, 0.33 mmol, 67%, *dr*$_{(cis\text{-}\mathbf{3na}/trans\text{-}\mathbf{3na})}$ = 1.2 : 1.0).

TLC: Rf = 0.53 (pentane/ ether = 95:5).

^{1}H NMR (400 MHz, $CDCl_3$) δ 5.42 – 5.24 (m, 2H, H-10+H-11), 4.93 (q, *J* = 1.5 Hz, 1H, H-21a), 4.85 (d, *J* = 1.6 Hz, 1H, H-21b), 4.07 (q, *J* = 7.1 Hz, 2H, H-28), 2.04 – 1.97 (m, 6H, *Alkyl-H*), 1.87 (dd, *J* = 8.0, 6.5 Hz, 2H, H-22+H-23), 1.50 – 1.38 (m, 3H, *Alkyl-H*, H-24a), 1.37 – 1.24 (m, 21H, *Alkyl-H*), 1.22 (t, *J* = 7.1 Hz, 3H, H-29), 1.08 (td, *J* = 8.1, 4.9 Hz, 1H, H-24b), 0.91 – 0.85 (m, 3H, H-19).

^{13}C{^{1}H} NMR (101 MHz, $CDCl_3$) δ 171.4 (C-25), 143.9 (C-20), 130.1 (*Alkyl-C)*, 130.0, (C10+C11) 112.3 (C-21), 60.4 (C-28), 37.3 (*Alkyl-C)*, 32.1 (*Alkyl-C)*, 29.9 (*Alkyl-C)*, 29.7 (*Alkyl-C)*, 29.6 (*Alkyl-C)*, 29.5 (*Alkyl-C)*, 29.4 (*Alkyl-C)*, 27.9 (*Alkyl-C)*, 27.4 (*Alkyl-C)*, 26.6 (*Alkyl-C)*, 22.8 (*Alkyl-C)*, 20.7 (*Alkyl-C*), 14.5 (C-29), 14.3 (C-19), 10.9 (C-24).

IR (neat): ν (cm^{-1}): 2924, 2854, 1734, 1647, 1464, 1380, 1275, 1161, 1113, 1097, 1041, 894.

HRMS (ESI-TOF) m/z: $[M + Na]^+$ Calcd for $C_{25}H_{44}O_2Na$ 399.3239; Found 399.3239.

Ethyl (*E*)-2-styrylcyclopropane-1-carboxylate (*trans*-**3oa**)

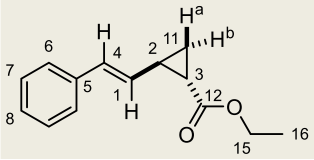

Trans-**3oa** was synthesized according to **GPI** on a 0.5 mmol scale using **1o** and **C$_4$** (5 mol%, 7.42 mg) at 25 °C for 30 min. Purification by flash chromatography gave a mixture of *trans* and *cis*

diastereoisomers as a colorless oil (*trans*-**3oa** + *cis*-**3oa**: 93 mg, 0.43 mmol, 86%, *dr*(*trans*-**3oa**/*cis*-**3oa**) = 1.0 : 1.0).

TLC: Rf = 0.60 (pentane/ ether = 98:2).

^{1}H NMR (400 MHz, $CDCl_3$) δ 7.37 – 7.31 (m, 2H, H-6), 7.31 – 7.25 (m, 2H, H-7), 7.22 – 7.15 (m, 1H, H-8), 6.58 (d, *J* = 15.9 Hz, 1H, H-4), 6.23 (dd, *J* = 15.9, 9.3 Hz, 1H, H-1), 4.15 (dddd, *J* = 17.9, 10.8, 7.1, 3.7 Hz, 2H, H-15), 2.09 (dd, *J* = 8.9, 6.9 Hz, 1H, H-2), 2.00 (td, *J* = 8.3, 6.0 Hz, 1H, H-3), 1.41 – 1.35 (m, 1H, H-11a), 1.35 – 1.30 (m, 1H, H-11b), 1.26 (t, *J* = 7.1 Hz, 3H, H-16).

^{13}C{^{1}H} NMR (101 MHz, $CDCl_3$) δ 172.2 (C-12), 137.5 (C-5), 131.5 (C-4), 128.6 (C-7), 127.7 (C-1), 127.2 (C-8), 126.1 (C-6), 60.7 (C15), 24.8 (C-2), 21.6 (C-3), 14.9 (C-11), 14.5 (C-16).

IR (neat): ν (cm^{-1}): 2983, 1720, 1448, 1404, 1380, 1178, 1148, 966, 815, 766, 693.

HRMS (ESI-TOF) m/z: $[M + H]^+$ Calcd for $C_{14}H_{17}O_2$ 217.1228; Found 217.1231.

Ethyl (*E*)-2-styrylcyclopropane-1-carboxylate (*cis*-**3oa**)

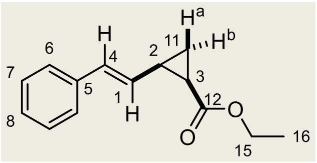

Cis-**3oa** was synthesized according to **GPI** on a 0.5 mmol scale using **1o** and **C4** (5 mol%, 7.42 mg) at 25 °C for 30 min. Purification by flash chromatography gave a mixture of *trans* and *cis* diastereoisomers as a colorless oil (*cis*-**3oa** + *trans*-**3oa**: 93 mg, 0.43 mmol, 86%, *dr*(*cis*-**3oa**/*trans*-**3oa**) = 1.0 : 1.0).

TLC: Rf = 0.51 (pentane/ ether = 98:2).

^{1}H NMR (400 MHz, $CDCl_3$) δ 7.32 – 7.27 (m, 4H, H-6+H-7), 7.21 (dt, *J* = 6.4, 2.9 Hz, 1H, H-8), 6.54 (d, *J* = 15.8 Hz, 1H, H-4), 5.75 (dd, *J* = 15.8, 8.7 Hz, 1H, H-1), 4.16 (q, *J* = 7.1 Hz, 2H, H-15), 2.17 (dd, *J* = 6.0, 3.7 Hz, 1H, H-2), 1.75 (ddd, *J* = 8.3, 5.3, 3.9 Hz, 1H, H-3), 1.48 (ddd, *J* = 8.7, 5.3, 4.4 Hz, 1H), 1.28 (t, *J* = 7.2 Hz, 3H, H-16, H-11b), 1.09 (ddd, *J* = 8.3, 6.2, 4.4 Hz, 1H,H-11a).

^{13}C{^{1}H} NMR (101 MHz, $CDCl_3$) δ 173.5 (C-12), 137.1 (C-5), 130.4 (C-4), 130.2 (C-1), 128.7 (C-7), 127.4 (C-8), 126.0 (C-6), 60.8 (C-15) 25.7 (C-2), 22.5 (C-3), 16.1 (C-11), 14.4 (C-16).

IR (neat): ν (cm^{-1}): 2983, 1717, 1407, 1342, 1175, 1037, 994, 964, 942, 819, 754, 698.

HRMS (ESI-TOF) m/z: $[M + H]^+$ Calcd for $C_{14}H_{17}O_2$ 217.1228; Found 217.1231.

mp: 40.3 – 41.4 °C.

Ethyl (*E*)-2-phenyl-3-styrylcyclopropane-1-carboxylate (*trans*-**3pa**)

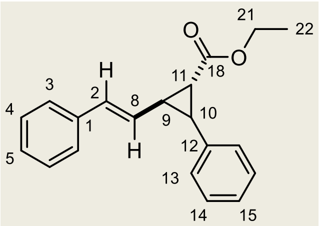

Trans-**3pa** was synthesized according to **GPI** on a 0.5 mmol scale using **1p** and $\mathbf{C_4}$ (5 mol%, 7.42 mg) at 25 °C for 30 min. Purification by flash chromatography gave a mixture of diastereoisomers as a colorless oil (24.7 mg, 0.084 mmol, 17%, *dr could not be determined due to signal overlap in ^{1}H NMR*).

TLC: Rf = 0.63 (pentane/ EtOAc = 98:2).

^{1}H NMR (400 MHz, CD_2Cl_2) δ 7.39 – 7.33 (m, 2H, H-3), 7.33 – 7.25 (m, 4H, H-4+H-14), 7.25 – 7.19 (m, 2H, H-5+H-15), 7.17 (tt, *J* = 6.0, 1.3 Hz, 2H, H-13), 6.63 (d, *J* = 15.9 Hz, 1H, H-2), 6.41 (ddd, *J* = 15.9, 8.3, 0.9 Hz, 1H, H-8), 4.16 (dddd, *J* = 18.0, 10.8, 7.1, 3.7 Hz, 2H, H-21), 2.83 (t, *J* = 5.9 Hz, 1H, H-11), 2.46 – 2.34 (m, 2H, H-9+H-10), 1.26 (t, *J* = 7.1 Hz, 3H, H-22).

^{13}C{^{1}H} NMR (101 MHz, CD_2Cl_2) δ 171.1, 139.8 (C-12), 137.7 (C-1), 131.9 (C-2), 128.9 (C-4+C-14), 127.6 (C-5), 127.0 (C-15), 126.9 (C-13), 126.6 (C-3), 126.3 (C-8), 61.2 (C-21), 34.9 (C-9), 32.8 (C-10), 31.1 (C-11), 14.5 (C-22).

IR (neat): ν (cm^{-1}): 2981, 1719, 1603, 1495, 1427, 1375, 1335, 1176, 1028, 964, 825, 752, 737, 692.

HRMS (ESI-TOF) m/z: [M + Na]$^+$ Calcd for $C_{20}H_{20}O_2Na$ 315.1361; Found 315.1341.

Ethyl (*E*)-2-phenyl-3-styrylcyclopropane-1-carboxylate (**3pa**)

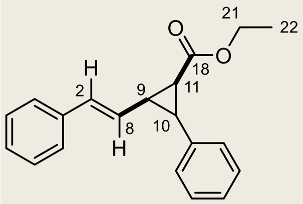

Compound **3pa** was synthesized according to **GPI** on a 0.5 mmol scale using **1p** and $\mathbf{C_4}$ (5 mol%, 7.42 mg) at 25 °C for 30 min. Purification by flash chromatography gave a mixture of diastereoisomers as a colorless oil (24.7 mg, 0.084 mmol, 17%, *dr could not be determined due to signal overlap in ^{1}H NMR*).

TLC: Rf = 0.56 (pentane/ EtOAc = 98:2).

^{1}H NMR (500 MHz, CD_2Cl_2) δ 7.39 – 7.35 (m, 2H, *Ar-H*), 7.33 – 7.26 (m, 6H, *Ar-H*), 7.24 – 7.20 (m, 2H, *Ar-H*), 6.67 (d, *J* = 15.8 Hz, 1H, H-2), 6.01 (dd, *J* = 15.8, 8.1 Hz, 1H, H-8), 3.92 (qd, *J* = 7.1, 1.4 Hz, 2H, H-21), 2.84 – 2.78 (m, 1H, H-9), 2.75 (dd, *J* = 9.5, 6.8 Hz, 1H, H-11), 2.23 (dd, *J* = 9.6, 5.0 Hz, 1H, H-10), 1.04 (t, *J* = 7.1 Hz, 3H, H-22).

^{13}C{^{1}H} NMR (126 MHz, CD_2Cl_2) δ 170.1 (C-18), 137.5 (C-1), 136.6 (C-12), 130.8 (C-2), 129.9 (C-8), 129.6 (*Ar-C*), 129.0 (*Ar-C*), 128.3 (*Ar-C*), 127.7 (*Ar-C*), 127.1 (*Ar-C*), 126.3 (Ar-C), 60.8 (C-21), 33.7 (C-11), 30.3 (C-10), 28.8 (C-9), 14.3 (C-22).

IR (neat): ν (cm^{-1}): 2928, 1724, 1498, 1448, 1373, 1271, 1182, 1030, 960, 733, 695.

HRMS (ESI-TOF) m/z: [M + H]$^+$ Calcd for $C_{20}H_{21}O_2$ 293.1536; Found 293.1541.

Ethyl bicyclo[4.1.0]hept-2-ene-7-carboxylate (3qa)

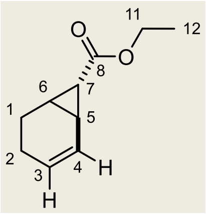

Compound **3qa** was synthesized according to **GPI** on a 1 mmol scale using **1q** and **C_4** (5 mol%, 7.42 mg) at 25 °C for 30 min. Purification by flash chromatography gave an inseparable mixture of **3qa** and **3qa’** as a colorless liquid (**3qa** + **3qa’**: 64.7 mg, 0.38 mmol, 39%, **3qa**/**3qa’** = 5.0 : 1.0).

TLC: Rf = 0.60 (pentane/ ether = 90:10).

^{1}H NMR (400 MHz, $CDCl_3$) δ 5.98 (d, *J* = 3.0 Hz, 1H), 5.56 (d, *J* = 7.1 Hz, 1H), 4.12 (d, *J* = 7.1 Hz, 2H), 2.11 – 1.59 (m, 7H), 1.26 (s, 3H).

^{13}C{^{1}H} NMR (101 MHz, $CDCl_3$) δ 173.5, 125.6, 125.3, 60.5, 25.2, 24.2, 21.1, 20.7, 17.7, 14.4.

IR (neat): ν (cm^{-1}): 2930, 1720, 1429, 1367, 1337, 1294, 1178, 1157, 1047, 1019, 799, 739.

HRMS (ESI-TOF) m/z: [M + H]$^+$ Calcd for $C_{10}H_{15}O_2$ 167.1067; Found 167.1072.

Ethyl bicyclo[4.1.0]hept-2-ene-7-carboxylate (3qa’)

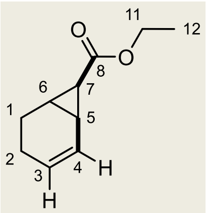

Compound **3qa’** was synthesized according to **GPI** on a 1 mmol scale using **1q** and **C_4** (5 mol%, 7.42 mg) at 25 °C for 30 min. Purification by flash chromatography gave an inseparable mixture of **3qa’** and **3qa** as a colorless liquid (**3qa’** + **3qa**: 64.7 mg, 0.38 mmol, 39%, **3qa’**/**3qa** = 1.0 : 5.0).

TLC: Rf = 0.60 (pentane/ ether = 90:10).

^{1}H NMR (400 MHz, $CDCl_3$) δ 5.87 – 5.80 (m, 1H), 5.80 – 5.73 (m, 4H), 4.12 (d, *J* = 7.1 Hz, 2H), 2.11 – 1.58 (m, 7H), 1.26 (s, 3H).

^{13}C{^{1}H} NMR (101 MHz, $CDCl_3$) δ 170.9, 129.4, 121.4, 60.1, 27.0, 21.8, 16.9, 16.1.

IR (neat): ν (cm^{-1}): 2930, 1720, 1429, 1367, 1337, 1294, 1178, 1157, 1047, 1019, 799, 739.

HRMS (ESI-TOF) m/z: [M + H]$^+$ Calcd for $C_{10}H_{15}O_2$ 167.1067; Found 167.1072.

Bicyclo[4.1.0]hept-2-en-7-ylmethanol (5qa/5qa’)

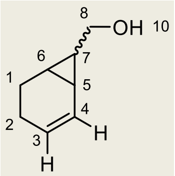

A THF solution of $LiAlH_4$ (2.4 M, 0.3 mL, 0.742 mmol, 3 equiv.) was added dropwise to a solution of **3qa’** and **3qa** at 0 °C. After 5 min., the ice bath was removed and the reaction stirred at 25 °C for 30 min. Purification by flash chromatography gave an inseparable mixture of diastereoisomers **5qa/5qa’** as a colorless oil (26.0 mg, 0.21 mmol, 85%, **5qa**/**5qa’** = 1 : 7.1).

TLC: Rf = 0.46 (pentane/ EtOAc = 80:20).

^{1}H NMR (400 MHz, $CDCl_3$) δ 6.04 (ddd, *J* = 9.9, 5.4, 3.0 Hz, 1H, H-4), 5.46 (ddd, *J* = 9.5, 6.8, 2.4 Hz, 1H, H-3), 3.49 (qd, *J* = 11.2, 7.0 Hz, 2H, H-8), 2.03 – 1.92 (m, 2H, H-2+H1), 1.86 – 1.70 (m, 1H, H-1), 1.56 (tdd, *J* = 13.0, 6.2, 3.5 Hz, 1H, H-2+H-10), 1.40 – 1.31 (m, 1H, H-7), 1.20 (dddt, *J* = 6.9, 5.2, 3.5, 1.8 Hz, 1H, H-6), 1.11 (ddd, *J* = 8.7, 5.3, 3.6 Hz, 1H, H-5).

$^{13}C\{^{1}H\}$ NMR (101 MHz, $CDCl_3$) δ 127.6 (C-4), 123.6 (C-3), 66.4 (C-8), 26.2 (C-7), 21.3 (C-1), 19.5 (C-6), 18.2 (C-2), 15.5 (C-5).

IR (neat): *ν* (cm^{-1}): 3309, 3028, 2918, 1639, 1442, 1074, 1048, 1020, 975, 769, 720, 679, 585.

HRMS (ESI-TOF) m/z: $[M + H]^+$ Calcd for $C_8H_{13}O$ 125.0966; Found 125.0962.

(2-(1-([1,1'-biphenyl]-4-yl)vinyl)cyclopropyl)methanol (*trans*-**5aa**)

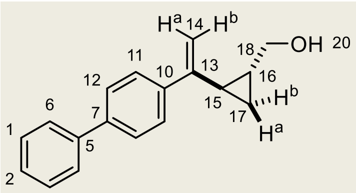

A THF solution of $LiAlH_4$ (0.85 mL, 2.05 mmol, 3 equiv.) was added drop wise at 0 °C to a solution of *trans*-**1aa** (205 mg, 0.7 mmol, 1 equiv.) in THF (9 mL). The ice bath was removed after 10 min. and the reaction continued stirring at room temperature for 30 min. The reaction was quenched with a saturated aqueous solution of NH_4Cl and extracted with EtOAc (3×20 mL). The collected organic layers were washed with water, brine and dried over Na_2SO_4 and concentarted under vacuo. The resulting white solid obtained after extraction was triturated with pentane to afford *trans*-**5aa** as a white solid (152 mg, 0.6 mmol, 87%).

^{1}H NMR (400 MHz, $CDCl_3$) δ 7.70 – 7.64 (m, 2H, H-11), 7.64 – 7.54 (m, 4H, H-6+H-12), 7.50 – 7.40 (m, 2H, H-1), 7.39 – 7.30 (m, 1H, H-2), 5.42 – 5.33 (m, 1H, H-14a), 5.00 (t, *J* = 1.1 Hz, 1H, H-14b), 3.68 (d, *J* = 6.8 Hz, 2H, H-18), 1.70 – 1.61 (m, 1H, H-15), 1.43 – 1.30 (m, 2H, H-16+H-20), 0.93 (ddd, *J* = 8.5, 5.5, 4.6 Hz, 1H, H-17a), 0.83 (dt, J = 8.6, 5.0 Hz, 1H, H-17b).

$^{13}C\{^{1}H\}$ NMR (101 MHz, $CDCl_3$) δ 147.7 (C-13), 140.9 (C-7), 140.5 (C-5), 140.4 (C-10), 128.9 (C-1), 127.4 (C-2), 127.1 (C-12), 127.1 (C-6), 126.6 (C-11), 110.1 (C-14), 66.8 (C-18), 23.4 (C-16), 21.7 (C-15), 11.5 (C-17).

IR (neat): *ν* (cm^{-1}): 3320, 3033, 2864, 1622, 1485, 1402, 1356, 1269, 1101, 1023, 1005, 895, 842, 768, 734, 687.

HRMS (ESI-TOF) m/z: $[M + Na]^+$ Calcd for $C_{18}H_{18}ONa$ 273.1255; Found 273.1260.

mp: 69.6 – 70.5 °C.

(2-(1-([1,1'-biphenyl]-4-yl)vinyl)cyclopropyl)methanol (*cis*-**5aa**)

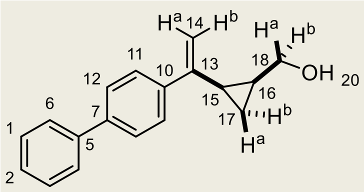

A THF solution of $LiAlH_4$ (0.85 mL, 2.05 mmol, 3 equiv.) was added dropwise at 0 °C to a solution of *cis*-**1aa** (205 mg, 0.7 mmol, 1 equiv.) in THF (8.7 mL). The ice bath was removed after 10 min and stirring continued at room temperature for 30 min. The reaction was quenched with a saturated aqueous solution of NH_4Cl and extracted with EtOAc (3×20 mL). The collected organic layers were washed with water, brine, dried over Na_2SO_4 and concentarted under vacuo. The resulting white solid obtained after extraction was triturated with pentane to afford *cis*-**5aa** as a white solid (162 mg, 0.64 mmol, 95%).

^{1}H NMR (400 MHz, $CDCl_3$) δ 7.72 (s, 2H, H-11), 7.61 (s, 4H, H-6+H-12), 7.45 (s, 2H, H-1), 7.35 (s, 1H, H-2), 3.57 (d, J = 6.0 Hz, 1H, H-18a), 3.41 (d, J = 8.5 Hz, 1H, H-18b), 2.11 – 1.99 (m, 1H, H-15), 1.60 (d, J = 8.6 Hz, 1H, H-16), 1.01 (d, J = 5.2 Hz, 1H, H-17b), 0.72 (d, J = 5.6 Hz, 1H, H-17a).

^{13}C{^{1}H} NMR (101 MHz, $CDCl_3$) δ 143.7 (C-13), 140.8 (C-5), 140.7 (C-7), 140.0 (C-10), 129.0 (C-1), 127.5 (C-2), 127.3 (C-6), 127.1 (C-12), 126.3 (C-1), 113.2 (C-14), 62.6 (C-18), 21.3 (C-15), 20.7 (C-18), 7.6 (C-17).

IR (neat): ν (cm^{-1}): 3389, 3063, 2880, 1818, 1620, 1486, 1433, 1402, 1253, 1052, 1018, 901, 842, 770, 735, 687.

HRMS (ESI-TOF) m/z: $[M + Na]^+$ Calcd for $C_{18}H_{18}ONa$ 273.1255; Found 273.1260.

mp: 90.2 – 91.0 °C.

(1'-([1,1'-biphenyl]-4-yl)-[1,1'-bi(cyclopropan)]-2-yl)methanol (*trans*-**6aa**)[8]

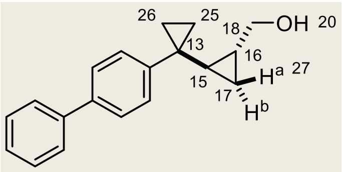

Chloromethyliodide ($ClCH_2I$, 141 mg, 0.8 mmol, 4 equiv.) was added dropwise to a hexane solution of diethyl zinc (0.67 mL, 0.9 M, 0.6 mmol, 3 equiv.) in 1,2-dichloroethane (1 mL) at 0 °C and stirred for 5 minutes. A solution of *trans*-**5aa** (50.1 mg, 0.2 mmol, 1 equiv.) in 1,2-dichloroethane (1 mL) was added dropwise and stirred for 10 minutes at the same temperature. The ice bath was removed and the reaction mixture was stirred at room temperature for 2.5 h. The reaction was quenched with water (10 mL), a saturated NH_4Cl solution (10 mL) and extracted with CH_2Cl_2 (3×20 mL). The organic layers were combined and washed with water (20 mL), brine (20 mL) and dried over $MgSO_4$. Purification by flash chromatography afforded *trans*-**6aa** as a pale yellow oil (28.2 mg, 0.1 mmol, 53%).

TLC: Rf = 0.39 (pentane/ EtOAc = 80:20).

^{1}H NMR (400 MHz, $CDCl_3$) δ 7.46 – 7.40 (m, 2H, *Ar-H*), 7.40 – 7.35 (m, 2H, *Ar-H*), 7.31 – 7.23 (m, 4H, *Ar-H*), 7.21 – 7.14 (m, 1H, *Ar-H*), 3.36 – 3.26 (m, 2H, H-18), 1.22 (s, 1H, H-20), 1.09 (ddd, J = 7.5, 6.1, 4.5 Hz, 1H, H-15), 0.90 – 0.74 (m, 1H, H-16), 0.70 – 0.60 (m, 2H, H-26), 0.58 – 0.48 (m, 2H, H-25), 0.33 – 0.23 (m, 2H, H-17).

^{13}C{^{1}H} NMR (101 MHz, CDCl3) δ 145.6 (*Ar-C*), 141.1 (*Ar-C*), 139.0 (*Ar-C*), 128.9 (*Ar-C*), 128.3 (*Ar-C*), 127.2 (*Ar-C*), 127.1 (*Ar-C*), 127.1 (*Ar-C*), 66.9 (C-18), 24.6 (C-13), 23.8 (C-15), 19.6 (C-16), 12.2 (C-26), 11.9 (C-25), 8.2 (C-16).

IR (neat): ν (cm^{-1}): 3335, 3077, 3029, 3001, 2869, 1601, 1486, 1099, 1033, 824, 763, 729, 696, 657, 578.

HRMS (ESI-TOF) m/z: $[M + Na]^+$ Calcd for $C_{19}H_{20}ONa$ 287.1412; Found 287.1416.

(1'-([1,1'-biphenyl]-4-yl)-[1,1'-bi(cyclopropan)]-2-yl)methanol (*cis*-**6aa**)[11]

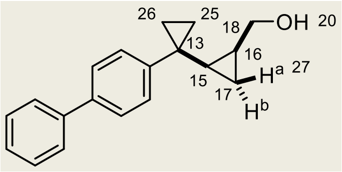

Chloromethyliodide ($ClCH_2I$, 141 mg, 0.8 mmol, 4 equiv.) was added dropwise to a hexane solution of diethyl zinc (0.67 mL, 0.9M, 0.6 mmol, 3 equiv.) in 1,2-dichloroethane (1 mL) at 0 °C and stirred for 5 minutes. A solution of *trans*-**5aa** (50.1 mg, 0.2 mmol, 1 equiv.) in 1,2-dichloroethane (1 mL) was added dropwise and stirred for 10 minutes at the same temperature. Next, the ice bath was removed and the reaction mixture was stirred at room temperature for 7 h. The reaction was quenched with water (10 mL) and sat. NH_4Cl sol. (10 mL) and extracted with CH_2Cl_2 (3×20 mL). The organic layers were combined and washed with water (20 mL), brine (20 mL) and dried over $MgSO_4$. Purification by flash chromatography afforded *cis*-**6aa** as a colorless oil (14.8 mg, 0.056 mmol, 28%).

TLC: Rf = 0.36 (pentane/ EtOAc = 80:20).

^{1}H NMR (400 MHz, $CDCl_3$) δ 7.61 – 7.52 (m, 4H, *Ar-H*), 7.43 (dt, *J* = 7.8, 3.4 Hz, 4H, *Ar-H*), 7.37 – 7.30 (m, 1H, *Ar-H*), 3.70 – 3.57 (m, 2H, H-18), 1.79 (td, *J* = 8.3, 5.8 Hz, 1H, H-15), 1.37 (td, *J* = 7.9, 5.3 Hz, 1H, H-16), 1.10 – 0.99 (m, 1H, H-25), 0.93 – 0.82 (m, 3H, H-25+H-26), 0.69 (td, *J* = 8.7, 5.2 Hz, 1H, H-17b), 0.14 (q, *J* = 5.5 Hz, 1H, H-17a).

^{13}C{^{1}H} NMR (101 MHz, $CDCl_3$) δ 146.0 (Ar-C), 140.9 (Ar-C), 138.8 (Ar-C), 128.9 (Ar-C), 127.3 (Ar-C), 127.3 (Ar-C), 127.1 (Ar-C), 126.8 (Ar-C), 63.1 (C-18), 22.5 (C-15), 21.1 (C-13), 20.6 (C-16), 15.9 (C-26), 13.2 (C-25), 6.6 (C-17).

IR (neat): ν (cm^{-1}): 3351, 3076, 3002, 2883, 1600, 1486, 1447, 1403, 1018, 910, 824, 762, 730, 696, 657, 561.

HRMS (ESI-TOF) m/z: $[M + Na]^+$ Calcd for $C_{19}H_{20}ONa$ 287.1412; Found 287.1416.

4-(2'-(methoxymethyl)-[1,1'-bi(cyclopropan)]-1-yl)-1,1'-biphenyl (*cis*-**7aa**)

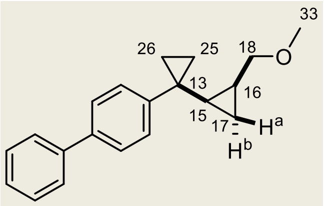

Chloromethyliodide ($ClCH_2I$, 141 mg, 0.8 mmol, 4 equiv.) was added dropwise to a hexane solution of diethyl zinc (0.67 mL, 0.9M, 0.6 mmol, 3 equiv.) in 1,2-dichloroethane (1 mL) at 0 °C and stirred for 5 minutes. A solution of *trans*-**5aa** (50.1 mg, 0.2 mmol, 1 equiv.) in 1,2-dichloroethane (1 mL) was added dropwise and stirred for 10 minutes at the same temperature. Next, the ice bath was removed and the reaction mixture was stirred at room temperature for 7 h. The reaction was quenched with water (10 mL) and sat. NH_4Cl sol. (10 mL) and extracted with CH_2Cl_2 (3×20 mL). The organic layers were combined and washed with water (20 mL), brine (20 mL) and dried over $MgSO_4$. Purification by flash chromatography afforded *cis*-**7aa** (side product) as a colorless oil (26.8 mg, 0.09 mmol, 48%).

TLC: Rf = 0.37 (pentane/ EtOAc = 95:5).

^{1}H NMR (400 MHz, $CDCl_3$) δ 7.62 – 7.57 (m, 2H, *Ar-H*), 7.56 – 7.51 (m, 2H, *Ar-H*), 7.47 – 7.37 (m, 4H, *Ar-H*), 7.36 – 7.31 (m, 1H, *Ar-H*), 3.65 – 3.58 (m, 1H, H-18), 3.31 (s, 3H, H-33), 3.27 – 3.19 (m, 1H, H-18), 1.72 (td, *J* = 8.6, 5.9 Hz, 1H, H-15), 1.36 (tdd, *J* = 8.7, 5.3, 3.4 Hz, 1H, H-16), 0.99 – 0.85 (m, 4H, H25+H-26), 0.73 (tdd, *J* = 8.6, 5.2, 0.9 Hz, 1H, H-17b), 0.22 (q, *J* = 5.5 Hz, 1H, H17a).

^{13}C{^{1}H} NMR (101 MHz, $CDCl_3$) δ 146.0 (*Ar-C*), 141.1 (*Ar-C*), 138.5 (*Ar-C*), 128.9 (*Ar-C*), 127.1 (*Ar-C*), 127.1 (*Ar-C*), 127.0 (*Ar-C*), 127.0 (*Ar-C*), 126.9 (*Ar-C*), 72.5 (C-18), 58.6 (C-33), 21.7 (C-15), 21.3 (C-13), 17.8 (C-16), 15.9 (C-26), 14.1 (C-25), 7.3 (C-17).

IR (neat): ν (cm^{-1}): 3002, 2923, 2811, 1486, 1097, 824, 763, 730, 696, 560, 513.

HRMS (ESI-TOF) m/z: $[M + Na]^+$ Calcd for $C_{20}H_{22}ONa$ 301.1568; Found 301.1563.

(2-(2-([1,1'-biphenyl]-4-yl)oxiran-2-yl)cyclopropyl)methanol (**8aa**)[12]

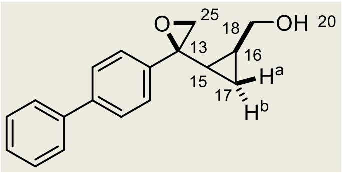

A decane solution of *t*-BuOOH (0.27 mL, 5-6 M, 1.5 mmol, 5 equiv.) was added dropwise at 0 °C to a solution of [VO(acac)$_2$] (8.12 mg, 5 mol%) in CH_2Cl_2 (7 mL) resulting in the formation of a reddish solution. After 5 minutes, a solution of *cis*-**5aa** (75.1 mg, 0.3 mmol, 1 equiv.) in CH_2Cl_2 (2 mL) was added dropwise and stirred for 5 minutes. Then, the ice bath was removed and stirring continued at room temperature for 18 h. The reaction was quenched with a saturated solution of Na_2SO_3 and extracted with CH_2Cl_2 (3×20 mL). The organic layers were collected and washed with water, brine and dried over $MgSO_4$. Purification by flash chromatography afforded **8aa** as a colorless oil (48.5 mg, 0.18 mmol, 61%).

TLC: Rf = 0.46 (pentane/EtOAc = 80:20).

^{1}H NMR (400 MHz, $CDCl_3$) δ 7.65 – 7.57 (m, 4H, *Ar-H*), 7.56 – 7.50 (m, 2H, *Ar-H*), 7.47 – 7.41 (m, 2H, *Ar-H*), 7.40 – 7.31 (m, 1H, *Ar-H*), 3.93 (d, *J* = 8.2 Hz, 1H, H-25), 3.86 (d, *J* = 11.3 Hz, 3H, H-25+H-18), 1.95 (td, *J* = 7.2, 4.2 Hz, 1H, H-15), 1.68 (s, 1H, H-20), 1.63 – 1.53 (m, 1H, H-16), 0.76 (q, *J* = 4.4 Hz, 1H, 17a), 0.69 (td, *J* = 7.7, 5.0 Hz, 1H, H-17b).

^{13}C{^{1}H} NMR (101 MHz, $CDCl_3$) δ 141.6 (*Ar-C*), 140.9 (*Ar-C*), 140.4 (*Ar-C*), 128.9 (*Ar-C*), 127.4 (*Ar-C*), 127.4 (*Ar-C*), 127.3 (*Ar-C*), 126.2 (*Ar-C*), 86.4 (C-13), 69.0 (C-25, 69.0 (C-18), 23.5 (C-15), 16.7 (C-16), 6.7 (C-17).

IR (neat): ν (cm^{-1}): 3432, 3029, 2927, 2867, 1735, 1600, 1486, 1245, 1075, 1046, 1007, 957, 839, 766, 734, 696, 655, 599, 552.

HRMS (ESI-TOF) m/z: $[M + Na]^+$ Calcd for $C_{18}H_{18}O_2Na$ 289.1205; Found 289.1178.

(2-([1,1'-biphenyl]-4-yl)-3-oxabicyclo[3.1.0]hexan-2-yl)methyl 3,5-dinitrobenzoate (**9aa**)

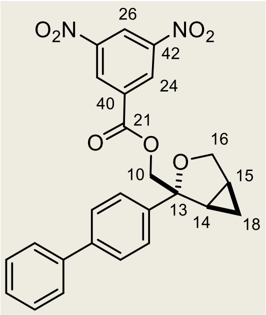

Compound **9aa** was synthesized according to a modified literature procedure.[13] 4-(dimethylamino)pyridine (26.6 mg, 0.28 mmol, 1.2 equiv.) was added at once at 0 °C to a solution of alcohol **8aa** (1 equiv., 44.5 mg, 0.178 mmol) and 3,5-dinitrobenzoyl chloride (1.28 equiv., 50.2 mg, 0.21 mmol). After 5 min the ice bath was removed and the stirring continued at room temperature for 1 h. The crude mixture was concentrated, adsorbed in silica using CH_2Cl_2 and purified by flash chromatography to give **9aa** as a light yellow solid (55.7 mg, 0.12 mmol, 72%). The resulting solid contained both diastereoisomers in a 12:1 ratio based on analysis of the crude reaction mixture by ^{1}H NMR. Crystals of suitable quality for an X-ray analysis were grown by slow evaporation of ethyl acetate at room temperature.

TLC: Rf = 0.50 (pentane/ EtOAc = 90:10).

^{1}H NMR (400 MHz, $CDCl_3$) δ 9.17 (t, *J* = 2.2 Hz, 1H, H-26), 9.01 (d, *J* = 2.2 Hz, 2H, H-24), 7.71 – 7.66 (m, 2H, *Ar-H*), 7.65 – 7.57 (m, 4H, *Ar-H*), 7.48 – 7.39 (m, 2H, *Ar-H*), 7.38 – 7.32 (m, 1H, Ar-H), 4.72 – 4.58 (m, 2H, H-10), 3.98 (d, *J* = 8.4 Hz, 1H, H-16), 3.90 (dd, *J* = 8.4, 3.0 Hz, 1H, H-16), 2.09 (td, *J* = 6.7, 4.9 Hz, 1H, H-14), 1.66 (dtd, *J* = 7.4, 5.4, 4.5, 2.9 Hz, 1H, H-15), 0.81 – 0.71 (m, 2H, H-18).

^{13}C{^{1}H} NMR (101 MHz, $CDCl_3$) δ 162.3 (C-9), 148.7 (C-42), 140.9 (Ar-C), 140.6 (Ar-C), 140.4 (Ar-C), 133.9 (C-40), 129.6 (*Ar-C*), 129.0 (C-24), 127.6 (*Ar-C*), 127.6 (*Ar-C*), 127.2 (*Ar-C*), 126.2 (*Ar-C*), 122.5 (C-26), 84.8 (C-13), 71.4 (C-10), 69.2 (C-16), 23.5 (C-14), 16.8 (C-15), 6.8 (C-18).

IR (neat): ν (cm^{-1}): 3102, 1734, 1539, 1483, 1341, 1263, 1161, 1071, 982, 918, 730, 717, 700, 602, 460.

HRMS (ESI-TOF) m/z: $[M + H]^+$ Calcd for $C_{25}H_{20}N_2O_7Na$ 483,1168; Found 483,1168.

mp: 185.7 – 187.0 °C.

Ethyl (E)-5-([1,1'-biphenyl]-4-yl)-6-(4,4,5,5-tetramethyl-1,3,2-dioxaborolan-2-yl)hex-4-enoate ((*E*)-**10aa**)

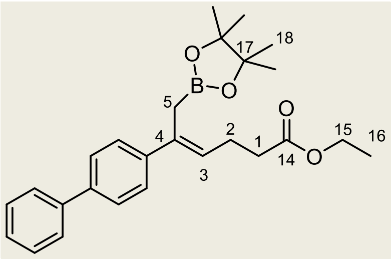

Following a procedure adapted from the literature.[14] Inside the glovebox, a microwave tube equipped with a stir bar was charged with copper(I) chloride (0.9 mg, 10 mol%), 4,4-bis(diphenylphosphino)-9,9-dimethylxanthene (6.36 mg, 11 mol%), potassium *tert*-butoxide (13.5 mg, 0.12 mmol), bis(pinacolato)diboron (30.5 mg, 0.12 mmol), and THF (0.4 mL). The reaction mixture was stirred for 5 minutes, followed by addition of the *trans*-**3aa** (29.2

mg, 0.1 mmol). After 5 min., morpholine•TFA (20.1 mg, 0.1 mmol) was added and the reaction stirred for three hours. The reaction was concentrated and purified by flash chromatography to give (*E*)-**10aa** as a colorless oil (24.1 mg, 0.057 mmol, 57%).

Following the same reaction protocol, *cis*-**3aa** afforded (*E*)-**10aa** in 79% yield (33.3 mg, 0.079 mmol, 79%).

TLC: Rf = 0.45 (pentane/ EtOAc = 95:5).

^{1}H NMR ($CDCl_3$, 300 MHz) δ 7.60 (m, 2H, *Ar-H*), 7.55 (m, 2H, *Ar-H*), 7.47-7.43 (m, 4H, *Ar-H*), 7.36-7.30 (m, 1H, *Ar-H*), 5.80-5.78 (t, 1H, H-3), 4.20-4.13 (q, 2H, H-15), 2.58-2.46 (m, 4H, H-1+H-2), 2.16 (s, 2H, H-5), 1.30-1.25 (t, 3H, H-16), 1.2 (s, 12H, H-18).

^{13}C{^{1}H} NMR ($CDCl_3$, 101 MHz) δ 173.4 (C-14), 143.0 (C-4), 141.1 (*Ar-C*), 139.4 (*Ar-C*), 136.8 (*Ar-C*), 128.7 (*Ar-C*), 127.1 (*Ar-C*), 126.9 (*Ar-C*), 126.8 (*Ar-C*), 126.4 (*Ar-C*), 125.4 (C-3), 83.6 (17), 60.3 (C-15), 34.2 (C-1), 24.8 (C-2), 24.7 (C-18), 14.3 (C-16).

^{11}B NMR (128 MHz, $CDCl_3$) δ 33.84.

IR (neat): ν (cm^{-1}): 2978, 2928, 1732, 1486, 1371, 1330, 1143, 965, 847, 765, 698.

HRMS (ESI-TOF) m/z: [M + Na]$^{+}$ Calcd for $C_{26}H_{33}BO_4Na$ 443.2374; Found 443.2351.

6. Large scale experiment

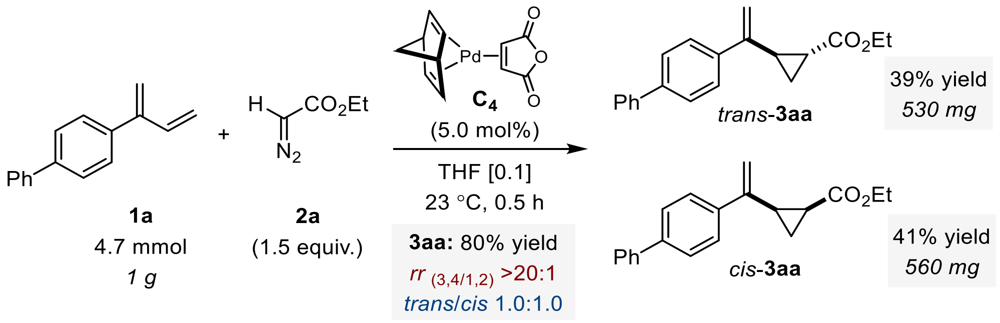

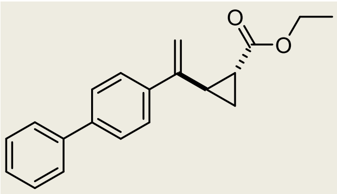

Compound *trans*-**3aa** was synthesized according to **GPI** on a 4.7 mmol scale using **1a** and **C**$_4$ (5 mol%, 69.7 mg,) at 25 °C in THF (47 mL) for 30 min. The reaction mixture was quenched by dilution using THF (45 mL), concentrated under reduced pressure, adsorbed in silica using CH_2Cl_2 and purified by flash chromatography to give the desired compound as a white solid (537 mg, 1.9 mmol, 39%), (*trans*-**3aa** + *cis*-**3aa**: 80% yield, *dr*$_{(trans\text{-}\mathbf{3aa}/cis\text{-}\mathbf{3aa})}$ = 1.0 : 1.0).

TLC: Rf = 0.54 (pentane/Et_2O = 98:2).

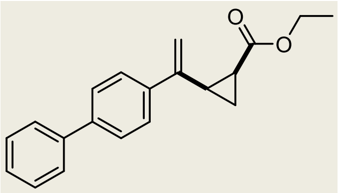

Compound *cis*-**3aa** was synthesized according to **GPI** on a 4.7 mmol scale using **1a** and **C**$_4$ (5 mol%, 69.7 mg,) at 25 °C in THF (47 mL) for 30 min. The reaction mixture was quenched by dilution using THF (45 mL), concentrated under reduced pressure, adsorbed in the silica using CH_2Cl_2 and purified by flash chromatography to give the desired compound as a colorless oil (566 mg, 1.9 mmol, 41%), (*cis*-**3aa** + *trans*-**3aa**: 80%, *dr*$_{(cis\text{-}\mathbf{3aa}/trans\text{-}\mathbf{3aa})}$ = 1.0 : 1.0).

TLC: Rf = 0.42 (pentane/Et_2O = 98:2).

7. X-ray analysis of 9aa

All data were collected on an Agilent Supernova diffractometer equipped with an ATLAS CCD detector using Cu radiation. The crystal was kept at 180.01(10) K during data collection. Using Olex2, the structure was solved with the ShelXT structure solution program using Intrinsic Phasing and refined with the ShelXL refinement package using Least Squares minimization.[15-17]

Empirical formula	C25 H20 N2 O7	
Formula weight	460.43	
Temperature	100.00(13) K	
Wavelength	1.54184 Å	
Crystal system	Monoclinic	
Space group	P 1 21 1	
Unit cell dimensions	a = 7.07831(5) Å	α = 90°
	b = 11.40109(8) Å	β = 101.1451(8)°
	c = 13.26715(11) Å	γ = 90°
Volume	1050.472(13) $Å^3$	
Z	2	
Density (calculated)	1.456 Mg/m^3	
Absorption coefficient	0.901 mm^{-1}	
F(000)	480	
Crystal size	0.292 x 0.103 x 0.067 mm^3	
Theta range for data collection	3.395 to 75.867°.	
Index ranges	-8<=h<=8, -14<=k<=14, -16<=l<=16	
Reflections collected	62905	
Independent reflections	4280 [R(int) = 0.0365]	
Completeness to theta = 67.684°	100.0 %	
Absorption correction	Semi-empirical from equivalents	
Max. and min. transmission	1.00000 and 0.81757	
Refinement method	Full-matrix least-squares on F^2	
Data / restraints / parameters	4280 / 1 / 307	
Goodness-of-fit on F^2	1.056	
Final R indices [I>2sigma(I)]	R1 = 0.0306, wR2 = 0.0832	
R indices (all data)	R1 = 0.0309, wR2 = 0.0834	
Absolute structure parameter	0.01(5)	
Extinction coefficient	n/a	
Largest diff. peak and hole	0.263 and -0.161 e.$Å^{-3}$	

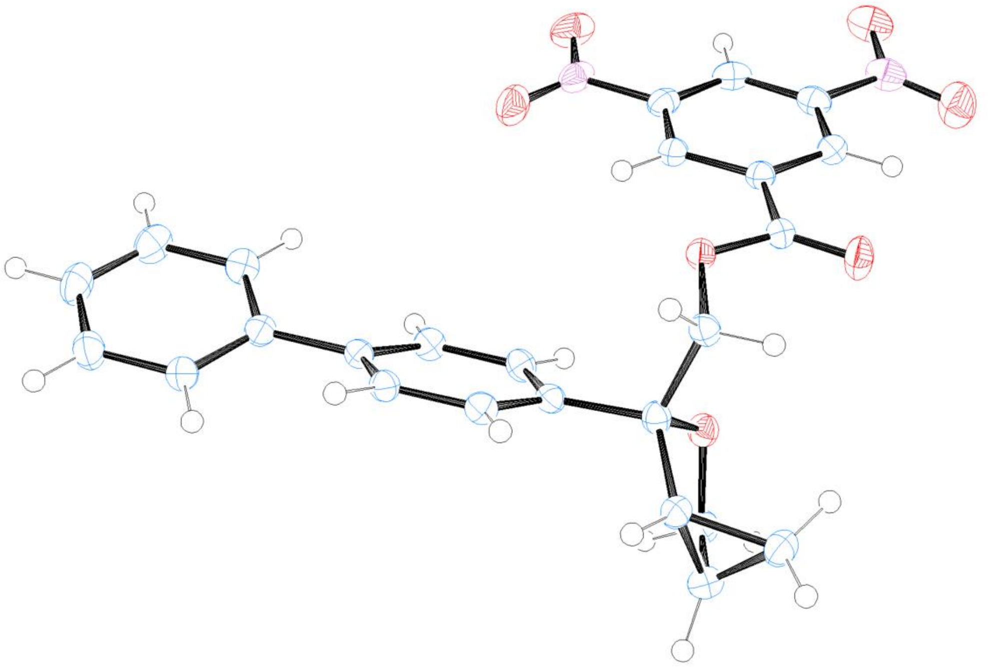

Figure S3. Crystal structure for **9aa**. Ellipsoids at 50% probability level.

8. References

(1) (a) Fiorito, D.; Folliet, S.; Liu, Y.; Mazet, C. A General Nickel-Catalyzed Kumada Vinylation for the Preparation of 2-Substituted 1,3-Dienes. *ACS Catal.* **2018**, *8*, 1392−1398. (b) Wang, Z.; Wang, Y.; Zhang, L. Soft Propargylic Deprotonation: Designed Ligand Enables Au-Catalyzed Isomerization of Alkynes to 1,3-Dienes. *J. Am. Chem. Soc.* **2014**, *136*, 8887–8890. (c) Imoto, J.; Hayashi, S.; Hirano, K.; Yorimitsu, H.; Oshima, K. Palladium-Catalyzed 1-Methylene-2-propenylation Reactions of Aryl Bromides with 3,4-Alkadien-1-ols via Carbon–Carbon Bond Cleavage for the Synthesis of 2-Aryl-1,3-butadiene Derivatives. *Bull. Chem. Soc. Jpn*. **2009**, *82*, 393−400. (d) Duguid, R. J.; Morrison, H. Photochemistry of 3-Methyl- and 4-Methyl-1,2-dihydronaphthalene in Solution. *J. Am. Chem. Soc.* **1991**, *113*, 1265–1271. (e) Shang, H.; Wang, Y.; Tian, Y.; Feng, J.; Tang, Y. The Divergent Synthesis of Nitrogen Heterocycles by Rhodium(II)-Catalyzed Cycloadditions of 1-Sulfonyl 1,2,3-Triazoles with 1,3-Dienes. *Angew. Chem., Int. Ed.* **2014**, *53*, 5662−5666. (f) Kotha, S.; Chavan, A. S.; Goyal, D. Diversity-Oriented Approaches to Polycycles and Heterocycles via Enyne Metathesis and Diels–Alder Reaction as Key Steps. *ACS Omega* **2019**, *4*, 22261–22273. (g) Li, Y.; Han, Y.; Xiong, H.; Zhu, N.; Qian, B.; Ye, C.; Kantchev, E. A. B.; Bao, H. Copper-Catalyzed Regioselective 1,2-Alkylesterification of Dienes to Allylic Esters. *Org. Lett.* **2016**, *18*, 392–395. (h) Kobayashi, S.; Kataoka, H.; Ishizone, T. Synthesis of Well-Defined Poly(ethylene-alt-1-vinyladamantane) via Living Anionic Polymerization of 2-(1-Adamantyl)-1,3-butadiene, Followed by Hydrogenation. *Macromolecules* **2009**, *42*, 5017–5026. (i) Li, H.; Fiorito, D.; Mazet, C. Exploring Site Selectivity of Iridium Hydride Insertion into Allylic Alcohols: Serendipitous Discovery and Comparative Study of Organic and Organometallic Catalysts for the Vinylogous Peterson Elimination. *ACS Catal.* **2017**, *7*, 1554–1562. (j) Poisson, P.-A.; Tran, G.; Besnard, C.; Mazet, C. Nickel-Catalyzed Kumada Vinylation of Enol Phosphates: A Comparative Mechanistic Study. *ACS Catal*. **2021**, *11*, 15041–15050.

(2) Krysan, D. J.; Mackenzie, P. B. A new, convenient preparation of bis(1,5-cyclooctadiene) nickel (0). *J. Org. Chem*. **1990**, *55*, 4229–4230.

(3) Bruno, N. C.; Tudge, M. T.; Buchwald, S. L. Design and preparation of new palladium precatalysts for C–C and C–N cross-coupling reactions. *Chem. Sci*. **2013**, *4*, 916–920.

(4) Viciu, M. S.; Navarro, O.; Germaneau, R. F.; Kelly, R. A.; Sommer, W.; Marion, N.; Stevens, E. D.; Cavallo, L.; Nolan, S. P. Synthetic and Structural Studies of (NHC)Pd(allyl)Cl Complexes (NHC = N-heterocyclic carbene). *Organometallics* **2004**, *23*, 1629–1635.

(5) Itoh, K.; Ueda, F.; Hirai, K.; Ishii, Y. New mixed olefin complexes of palladium (0). A stabilization of the Pd(0) state with a combination of electron-donating and electron-withdrawing olefins. *Chem. Lett.* **1977**, *6*, 877–880.

(6) Davies, M. L. H.; Cantrell, R. W.; Jr.; Romines, R. K.; Baum, S. Synthesis of Furanes via Rhodium(II) Acetate-Catalysed Reaction of Acetylenes with α-diazocarbonyls: Ethyl 2-methyl-5-Phenyl-3-furancarboxylate. *Org. Synth*. **1992**, *70*, 92–97.

(7) Tran, V. T.; Li, Z.; Apolinar, O.; Derosa, J.; Joannou, M. V.; Wisniewski, S. R.; Eastgate, M. D.; Engle, K. M. Ni(COD)(DQ): An Air-Stable 18-Electron Nickel(0)–Olefin Precatalyst. *Angew. Chem., Int. Ed*. **2020**, *59*, 7409–7413.

(8) DeBergh, J. R.; Niljianskul, N.; Buchwald, S. L. Synthesis of Aryl Sulfonamides via Palladium-Catalyzed Chlorosulfonylation of Arylboronic Acids. *J. Am. Chem. Soc*. **2013**, *135*, 10638–10641.

(9) Viciu, M. S.; Navarro, O.; Germaneau, R. F.; Kelly, R. A.; Sommer, W.; Marion, N.; Stevens, E. D.; Cavallo, L.; Nolan, S. P. Synthetic and Structural Studies of (NHC)Pd(allyl)Cl Complexes (NHC = N-heterocyclic carbene). *Organometallics* **2004**, *23*, 1629–1635.

(10) Garbo, M.; Besnard, C.; Guénée, L.; Mazet, C. Access to Optically Active 7-Membered Rings by a 2-Step Synthetic Sequence: Cu-Catalyzed Stereoselective Cyclopropanation of Branched 1,3-Dienes/Rh-Catalyzed Stereoconvergent [5 + 2] Cycloaddition. *ACS Catal*. **2020**, *10*, 9604–9611.

(11) Denmark, S. E.; Edwards, J. P. A Comparison of (Chloromethyl)zinc and (Iodomethyl)zinc Cyclopropanation Reagents. *J. Org. Chem.* **1991**, *56*, 6974–6981.

(12) Cohen, A.; Siddaraju, Y.; Marek, I. Directed Diastereoselective Cyclopropanation and Epoxidation of Alkenyl Cyclopropyl Carbinol Derivatives. *Org. Lett*. **2022**, *24*, 8322–8325.

(13) Navuluri, C.; Charette, A. B. Diastereoselective Fluorocyclopropanation of Chiral Allylic Alcohols Using an α-Fluoroiodomethylzinc Carbenoid. *Org. Lett.* **2015**, *17*, 4288–4291.

(14) Lippincott, D. J.; Linstadt, R. T. H.; Maser, M. R.; Lipshutz, B. H. Synthesis of Functionalized [3], [4], [5] and [6] Dendralenes through Palladium-Catalyzed Cross-Couplings of Substituted Allenoates. *Angew. Chem., Int. Ed.* **2017**, *56*, 847−850.

(15) Dolomanov, O.V.; Bourhis, L.J.; Gildea, R.J; Howard, J.A.K.; Puschmann, H. OLEX2: A Complete Structure Solution, Refinement and Analysis Program. *J. Appl. Cryst.* **2009**, *42*, 339−341.

(16) Sheldrick, G.M. SHELXT - Integrated Space-Group and Crystal-Structure Determination. *Acta Cryst.* **2015**, A71, 3−8.

(17) Sheldrick, G.M. Crystal Structure Refinement with SHELXL. *Acta Cryst.* **2015**, C71, 3−8.

9. NMR spectra of new compounds

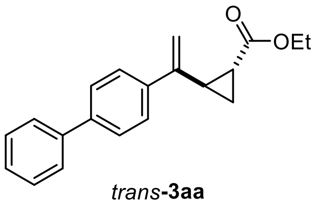

trans-**3aa**

^{1}H NMR ($CDCl_3$, 400 MHz)

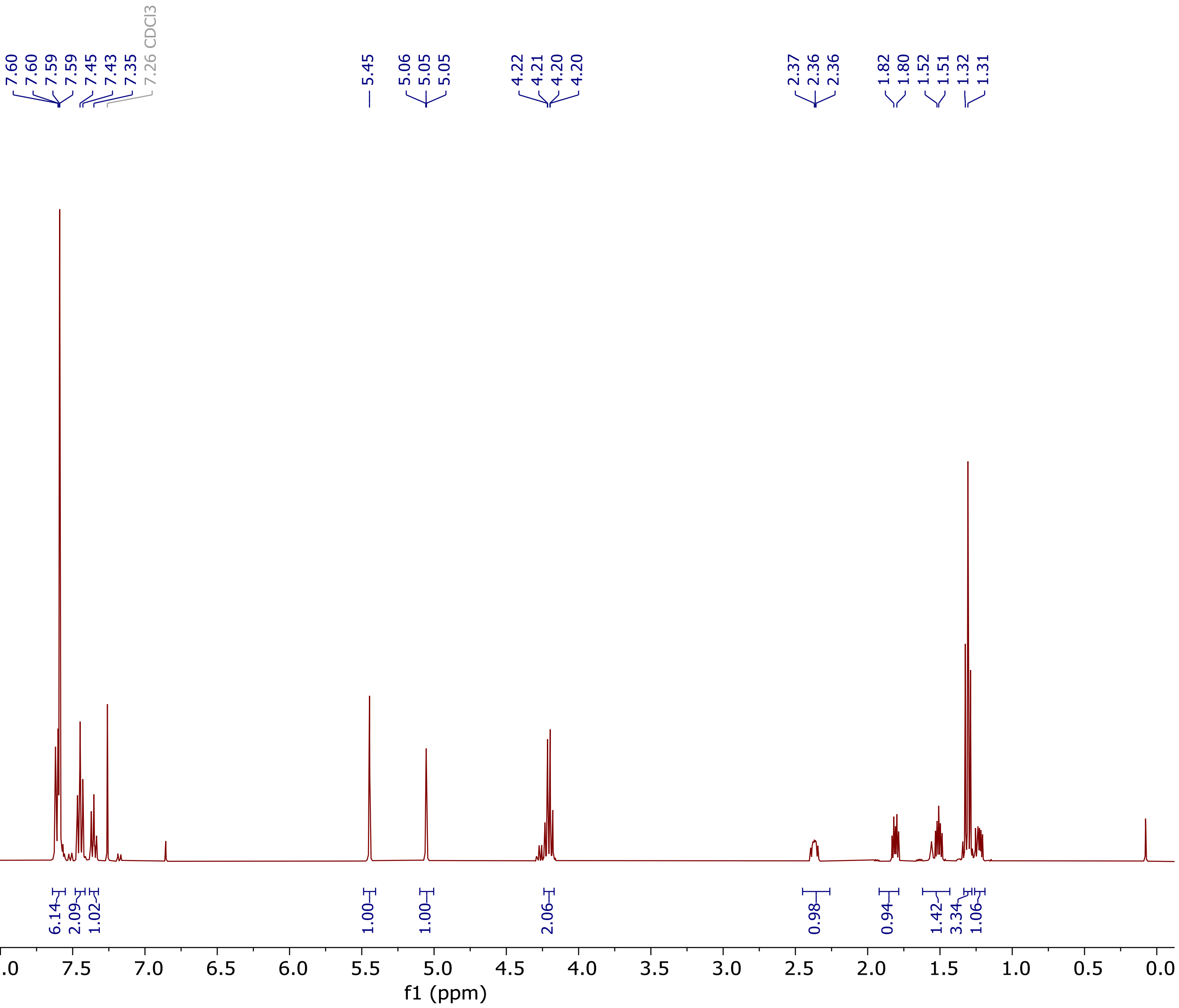

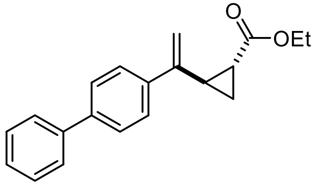

trans-**3aa**

$^{13}C\{^1H\}$ NMR ($CDCl_3$, 101 MHz)

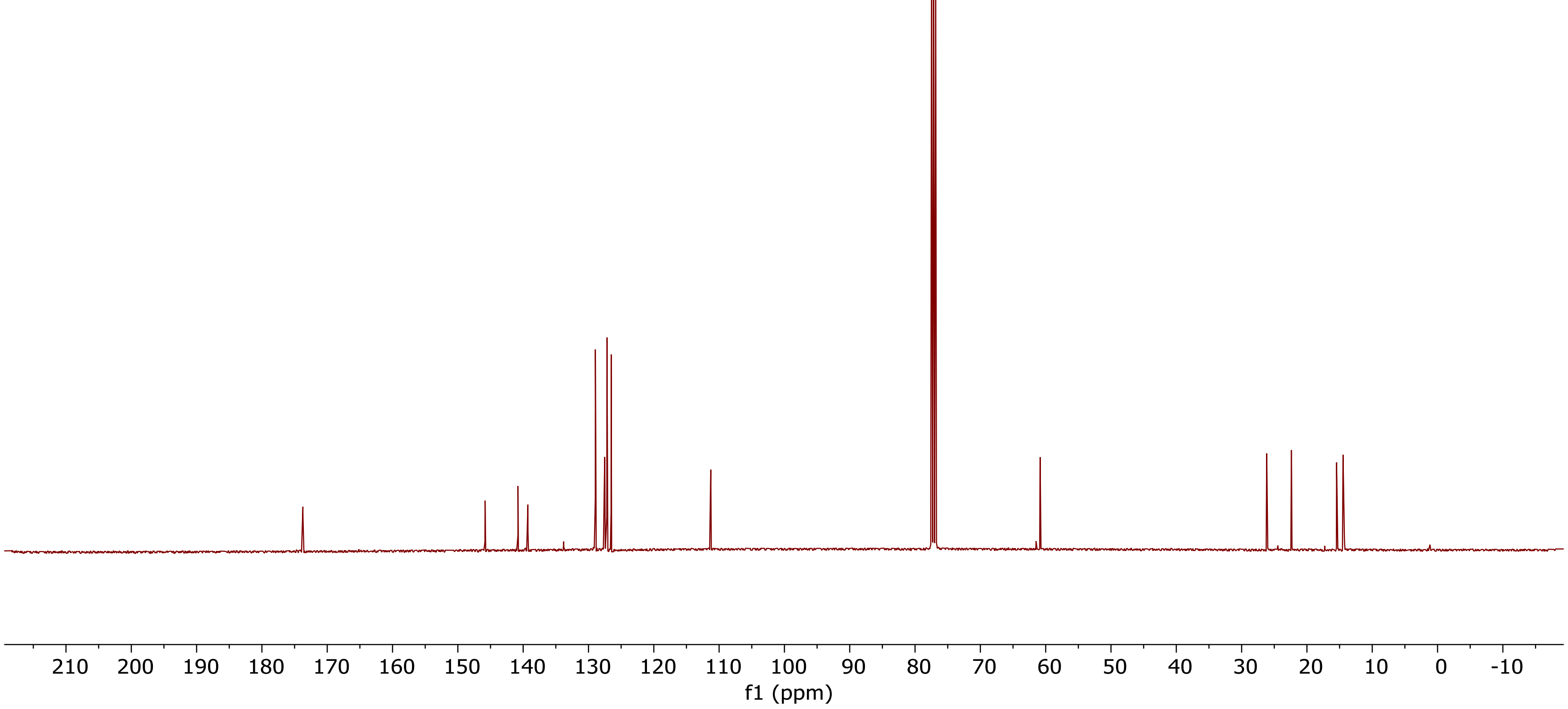

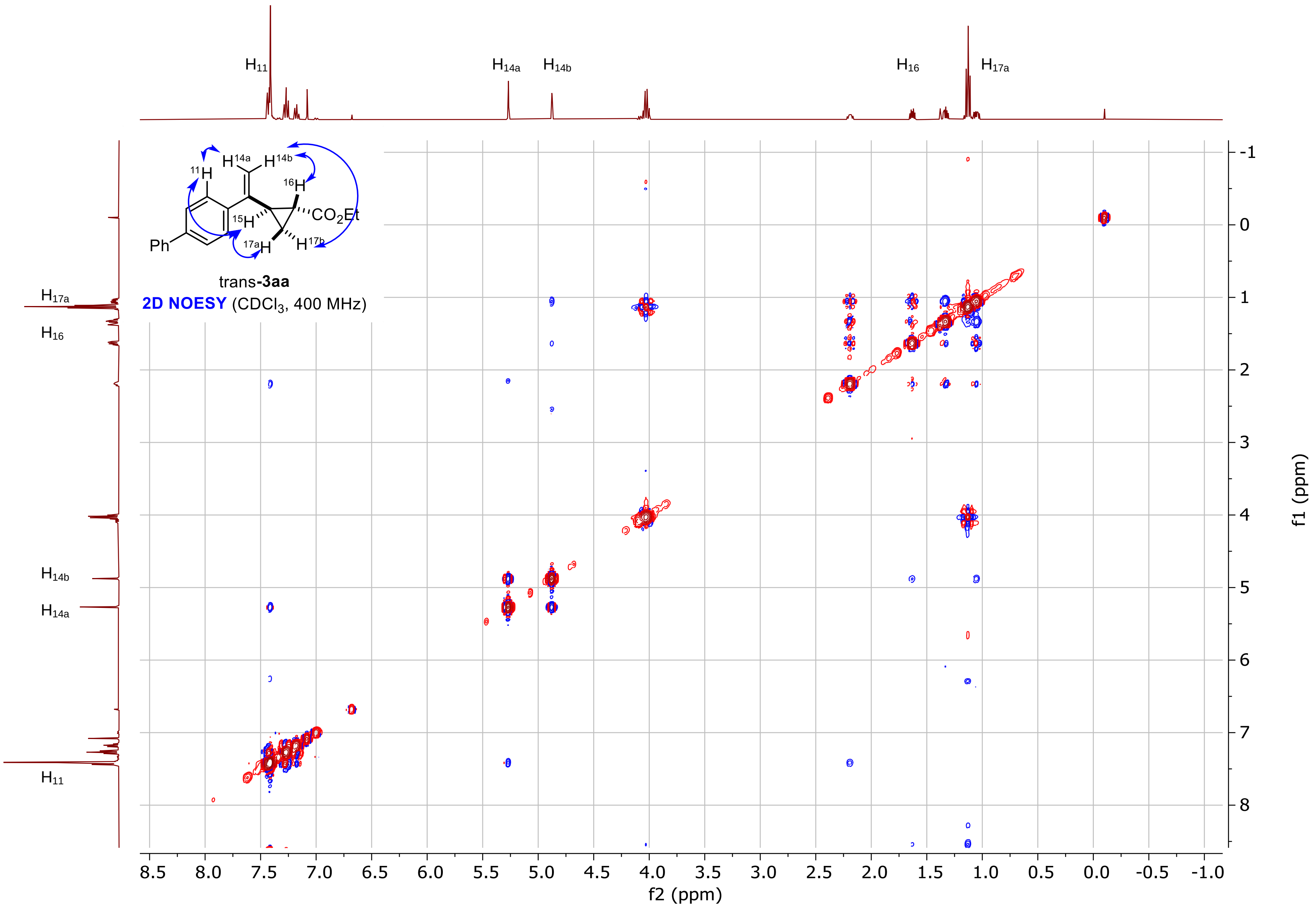
H11
H14a
H14b
H16
H17a
15H
17aH
H17b
CO2Et
Ph
trans-3aa
2D NOESY (CDCl3, 400 MHz)
f2 (ppm)
f1 (ppm)

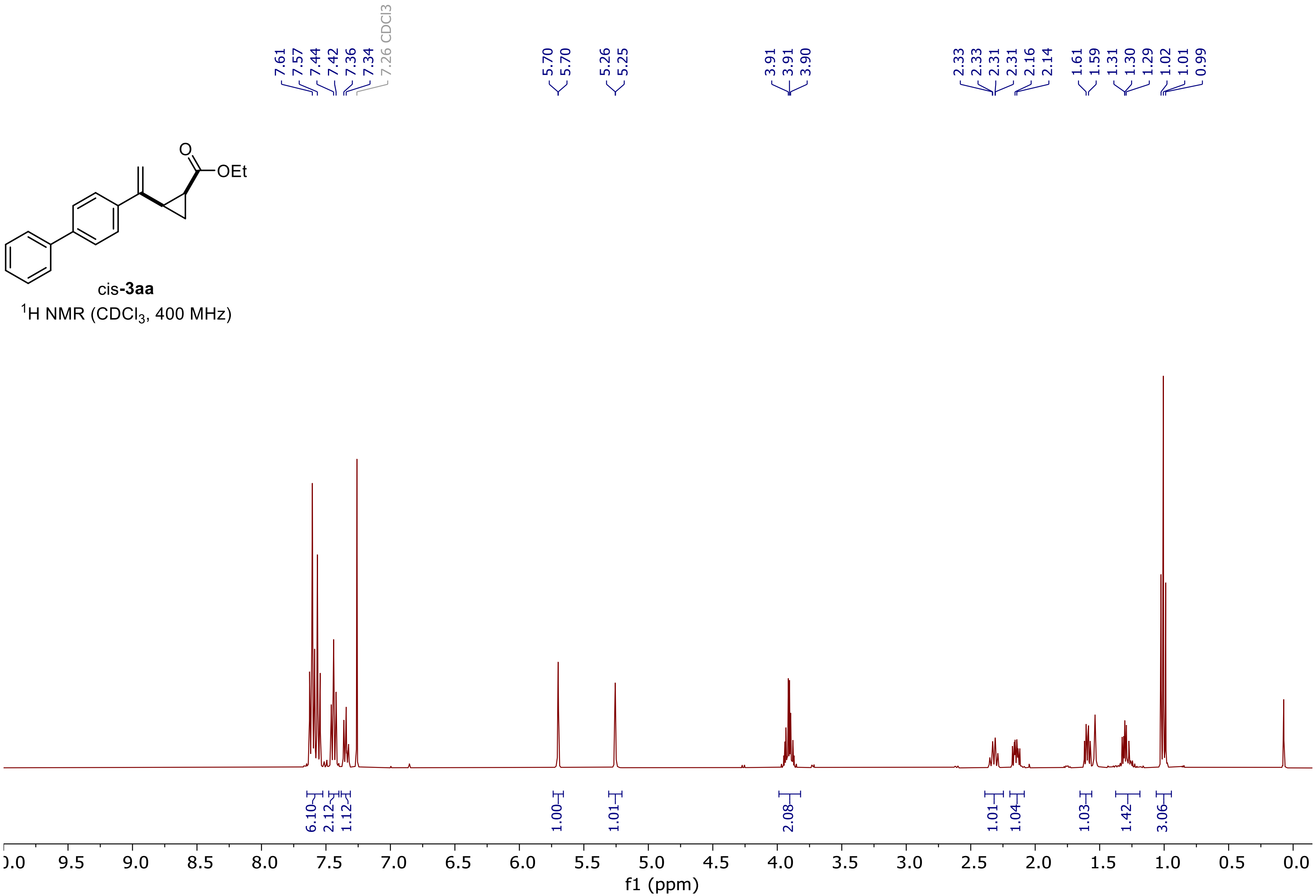

cis-**3aa**

^{1}H NMR ($CDCl_3$, 400 MHz)

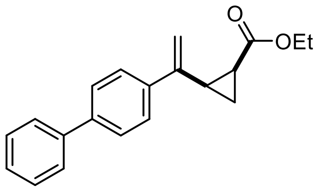

cis-**3aa**

$^{13}C\{^{1}H\}$ NMR ($CDCl_3$, 101 MHz)

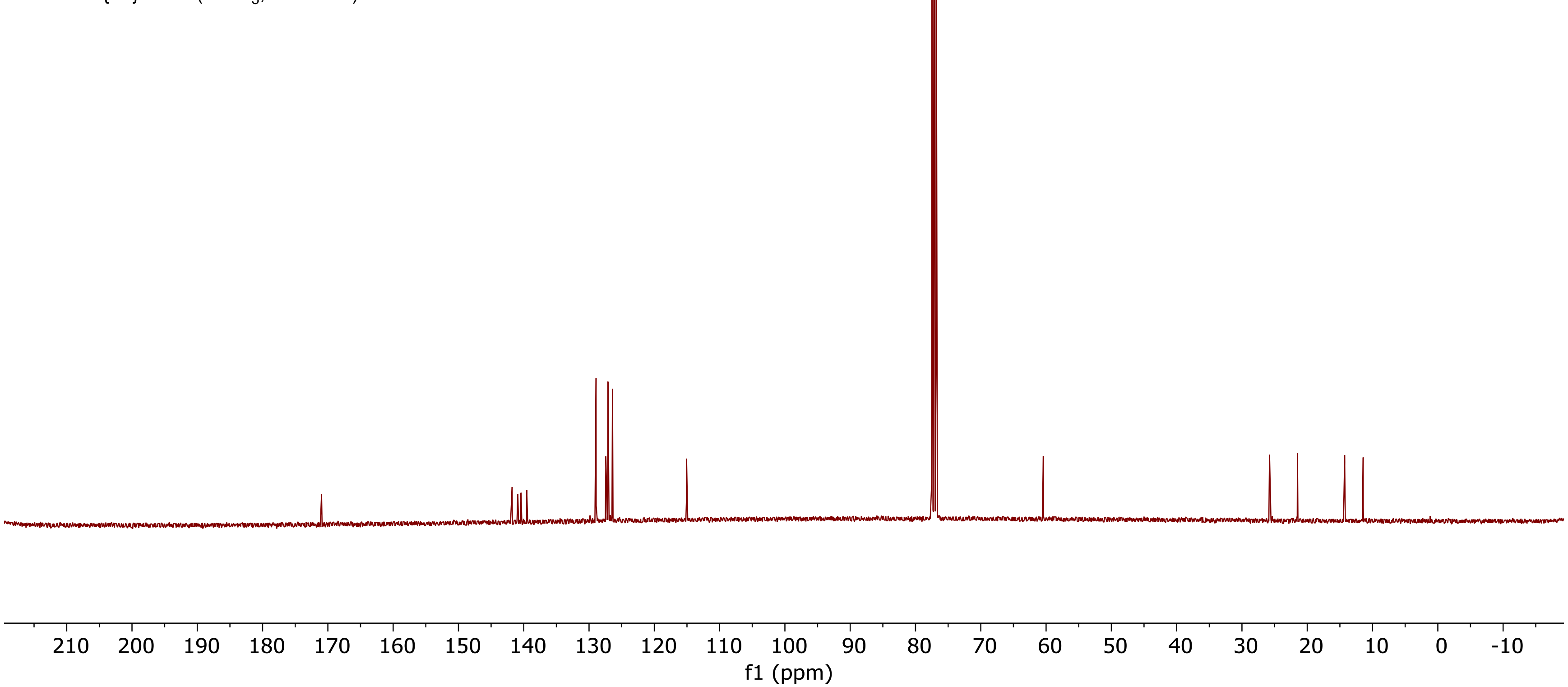

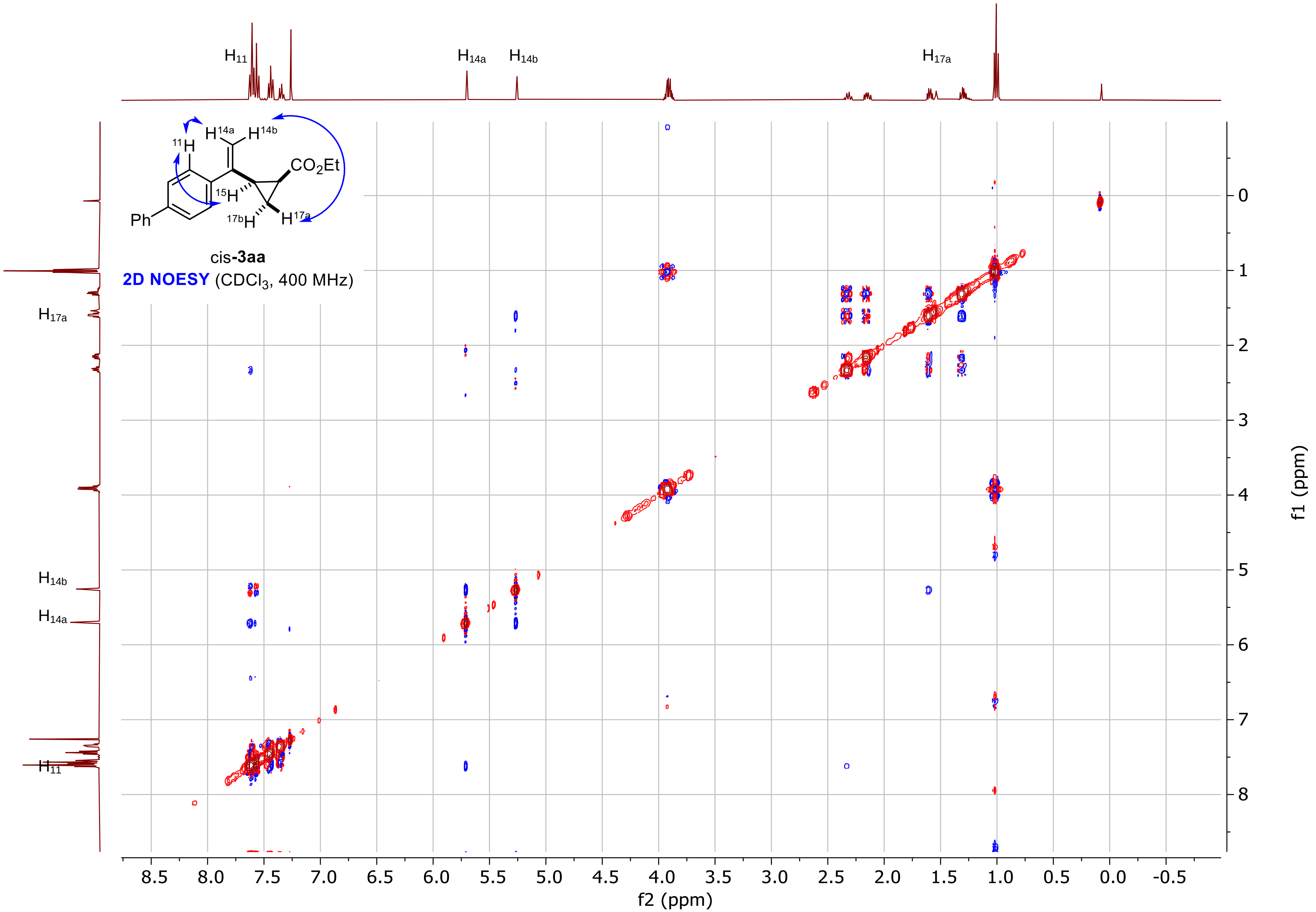
H11
H14a
H14b
H17a
CO2Et
Ph
cis-3aa
2D NOESY (CDCl3, 400 MHz)
f2 (ppm)
f1 (ppm)

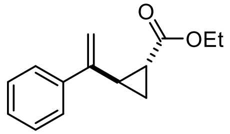

trans-**3ba**
^{1}H NMR ($CDCl_3$, 400 MHz)

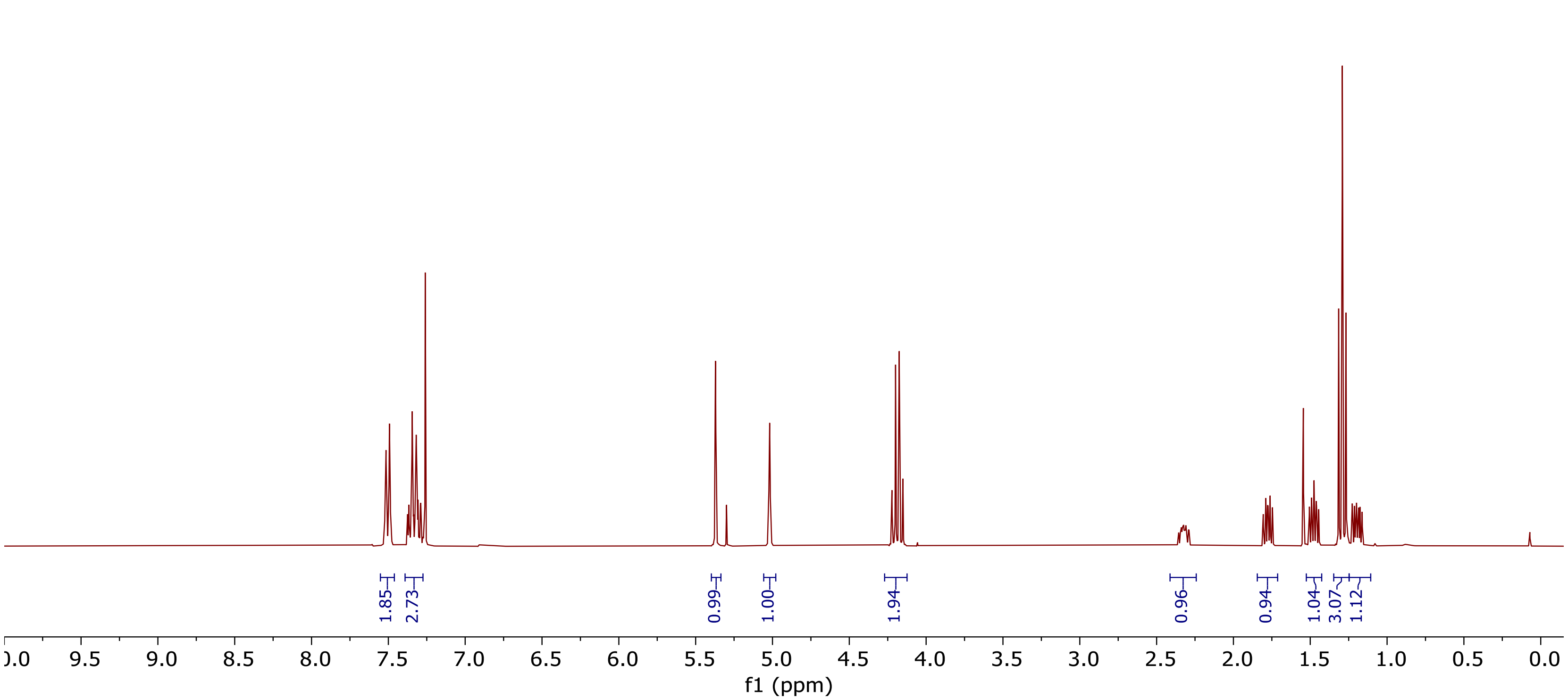

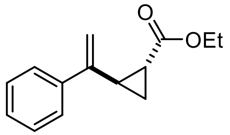

trans-**3ba**

$^{13}C\{^{1}H\}$ NMR ($CDCl_3$, 101 MHz)

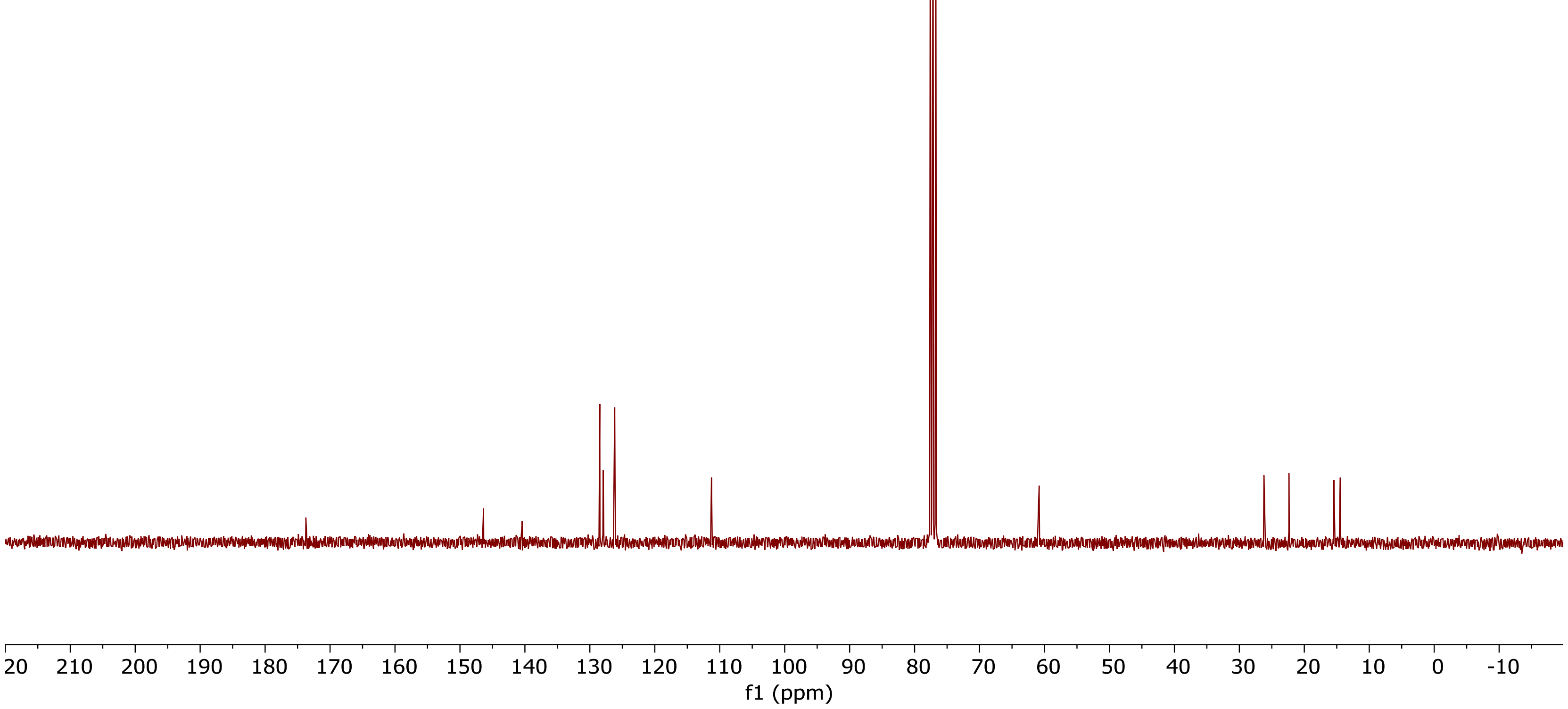

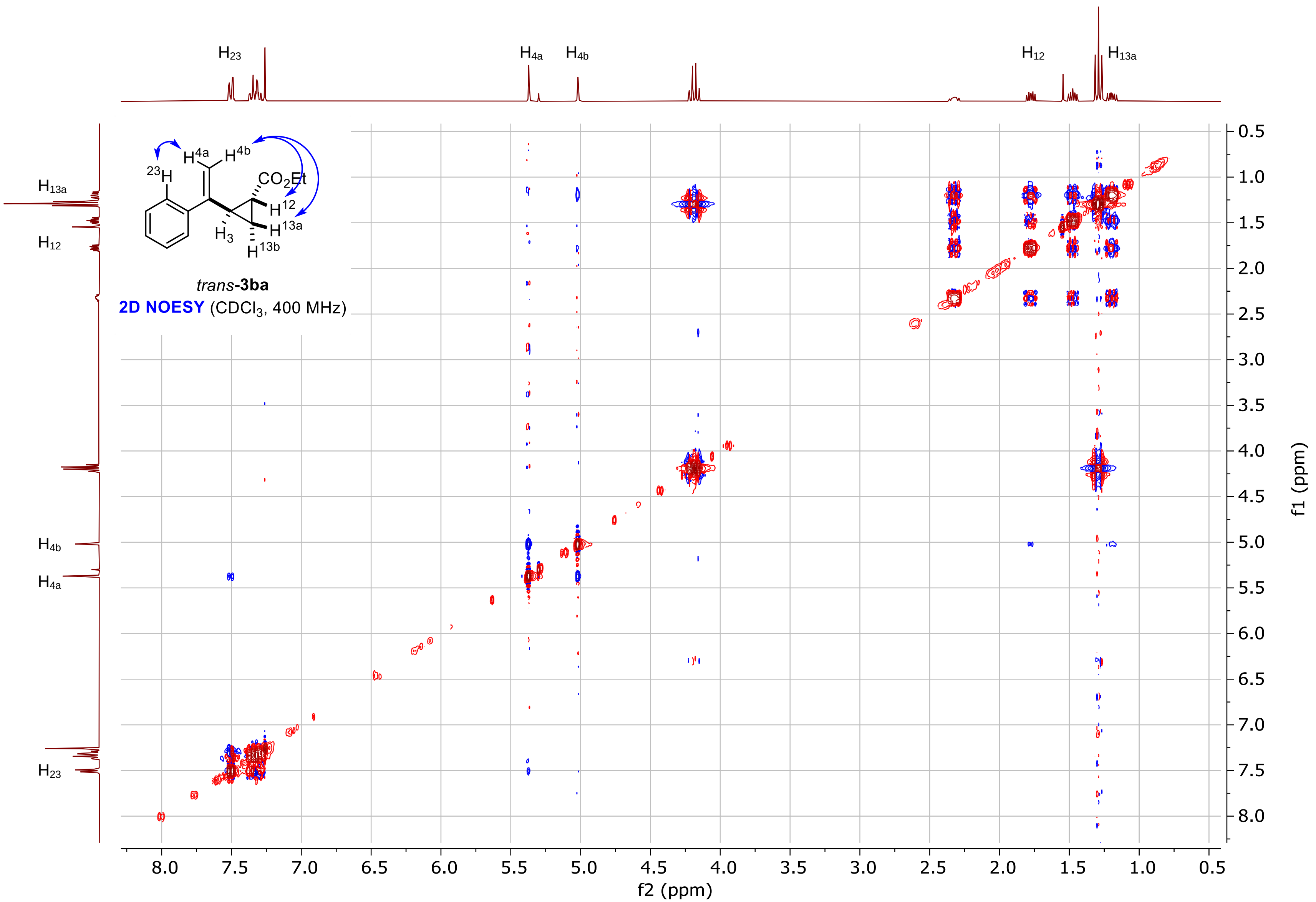
H23
H4a
H4b
H12
H13a
CO2Et
H3
H13b
trans-3ba
2D NOESY (CDCl3, 400 MHz)
f2 (ppm)
f1 (ppm)

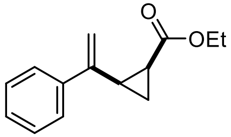

cis-**3ba**

^{1}H NMR (CD_2Cl_2, 400 MHz)

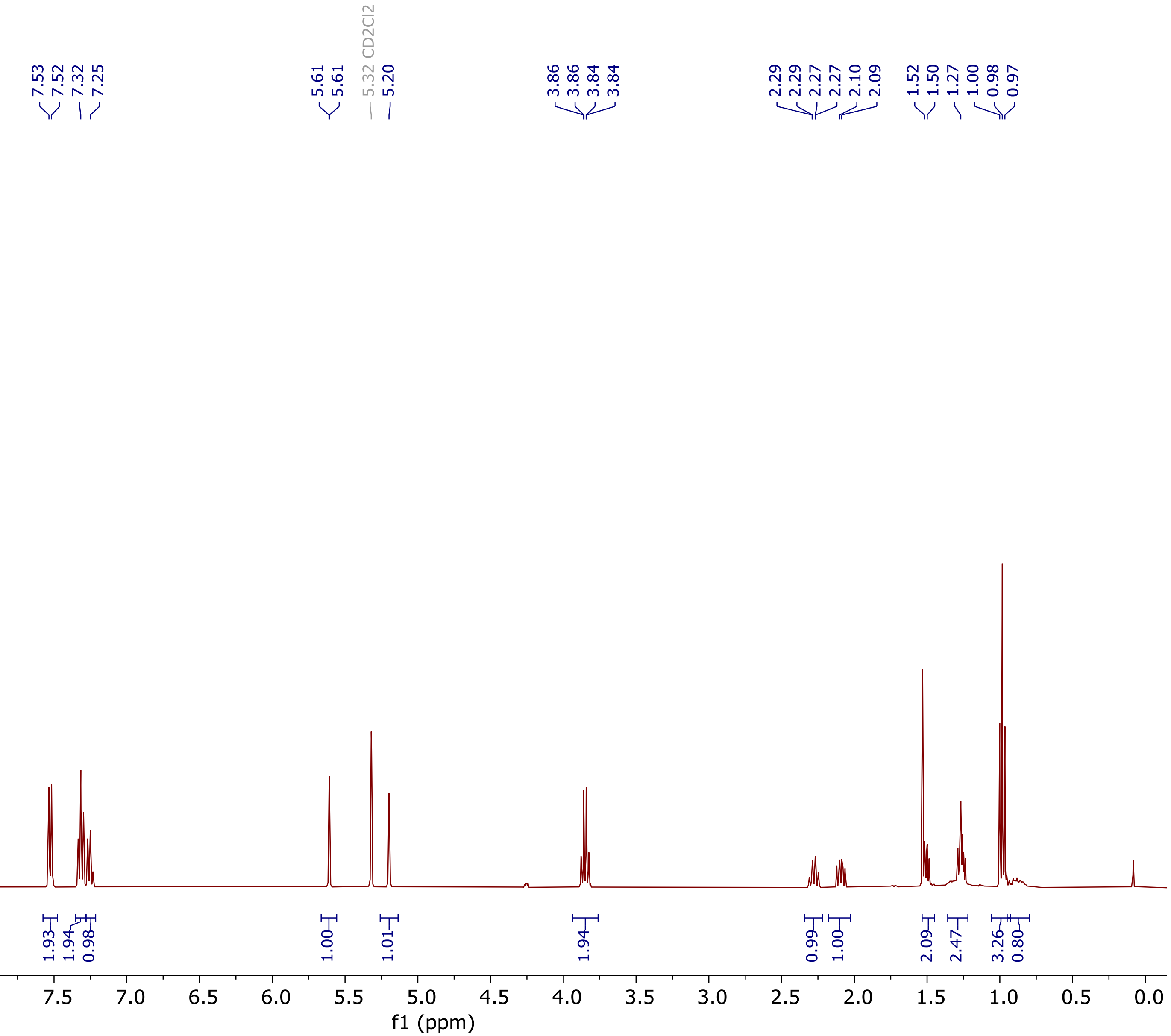

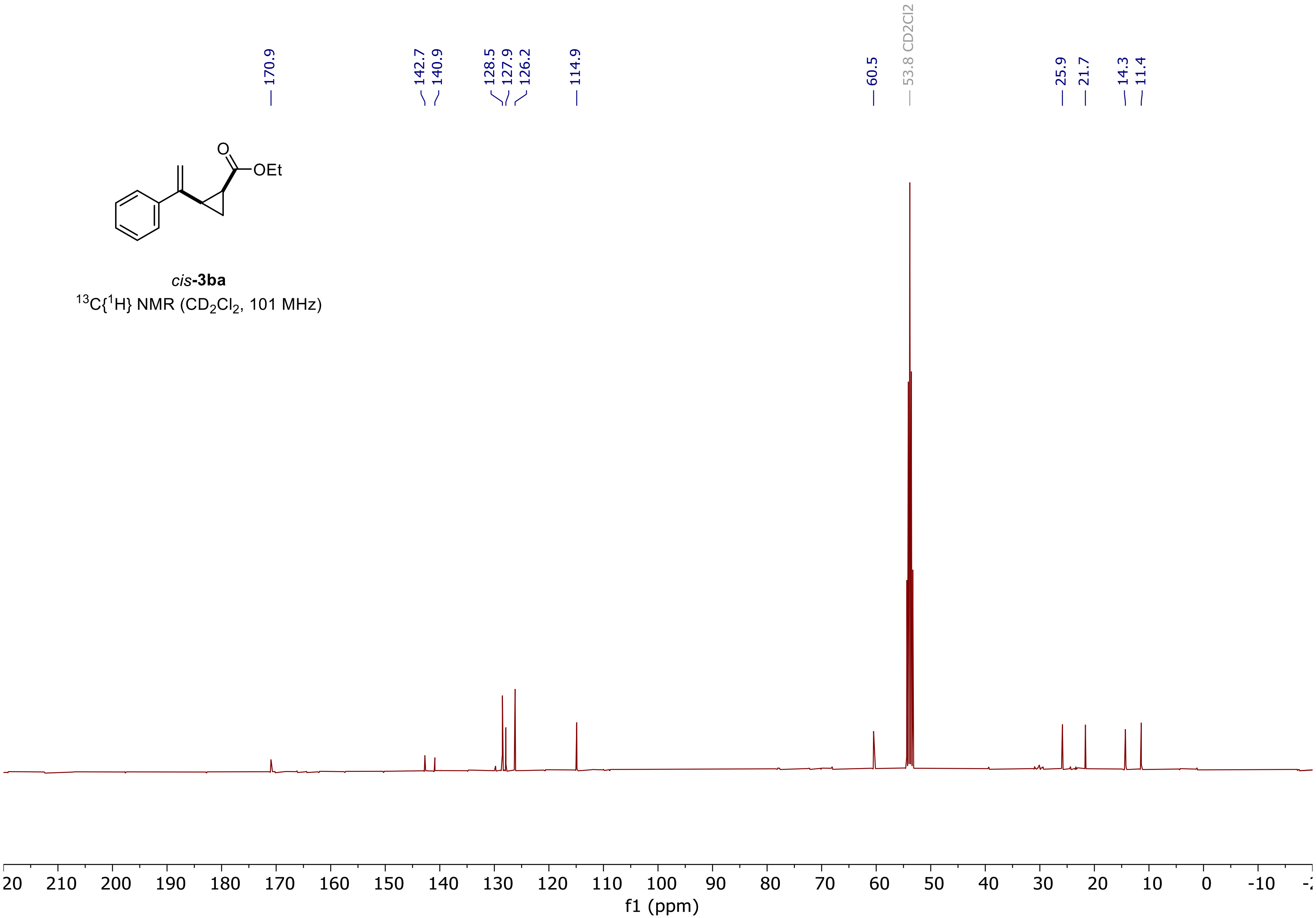

cis-**3ba**
$^{13}C\{^{1}H\}$ NMR (CD_2Cl_2, 101 MHz)

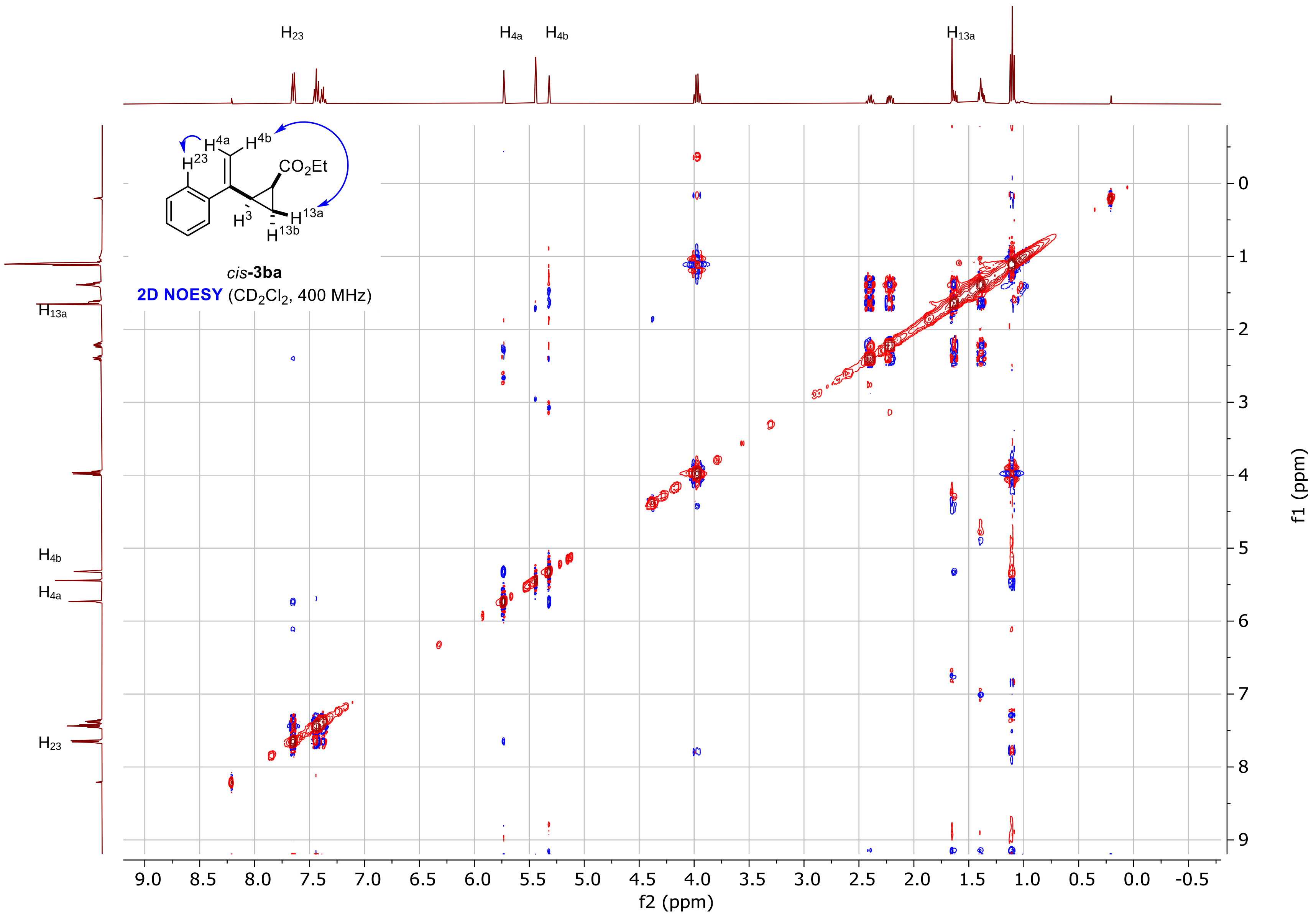
H23
H4a
H4b
H13a
H4a
H4b
H23
CO2Et
H3
H13a
H13b
cis-3ba
2D NOESY (CD2Cl2, 400 MHz)
H13a
H4b
H4a
H23
f2 (ppm)
f1 (ppm)

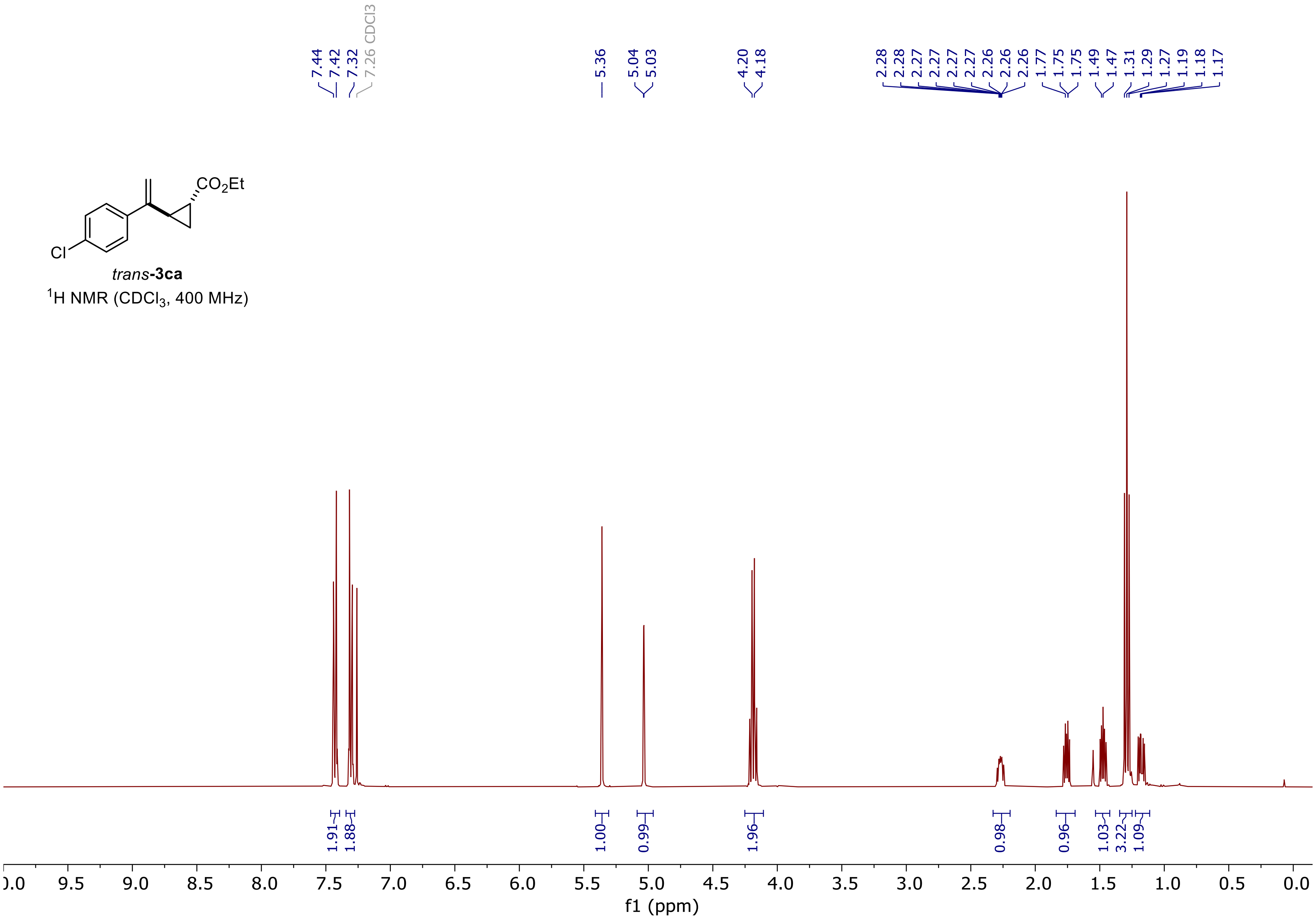
CO2Et
Cl
trans-3ca
1H NMR (CDCl3, 400 MHz)
7.44
7.42
7.32
7.26 CDCl3
5.36
5.04
5.03
4.20
4.18
2.28
2.28
2.27
2.27
2.27
2.27
2.26
2.26
2.26
1.77
1.75
1.75
1.49
1.47
1.31
1.29
1.27
1.19
1.18
1.17
1.91
1.88
1.00
0.99
1.96
0.98
0.96
1.03
3.22
1.09
f1 (ppm)

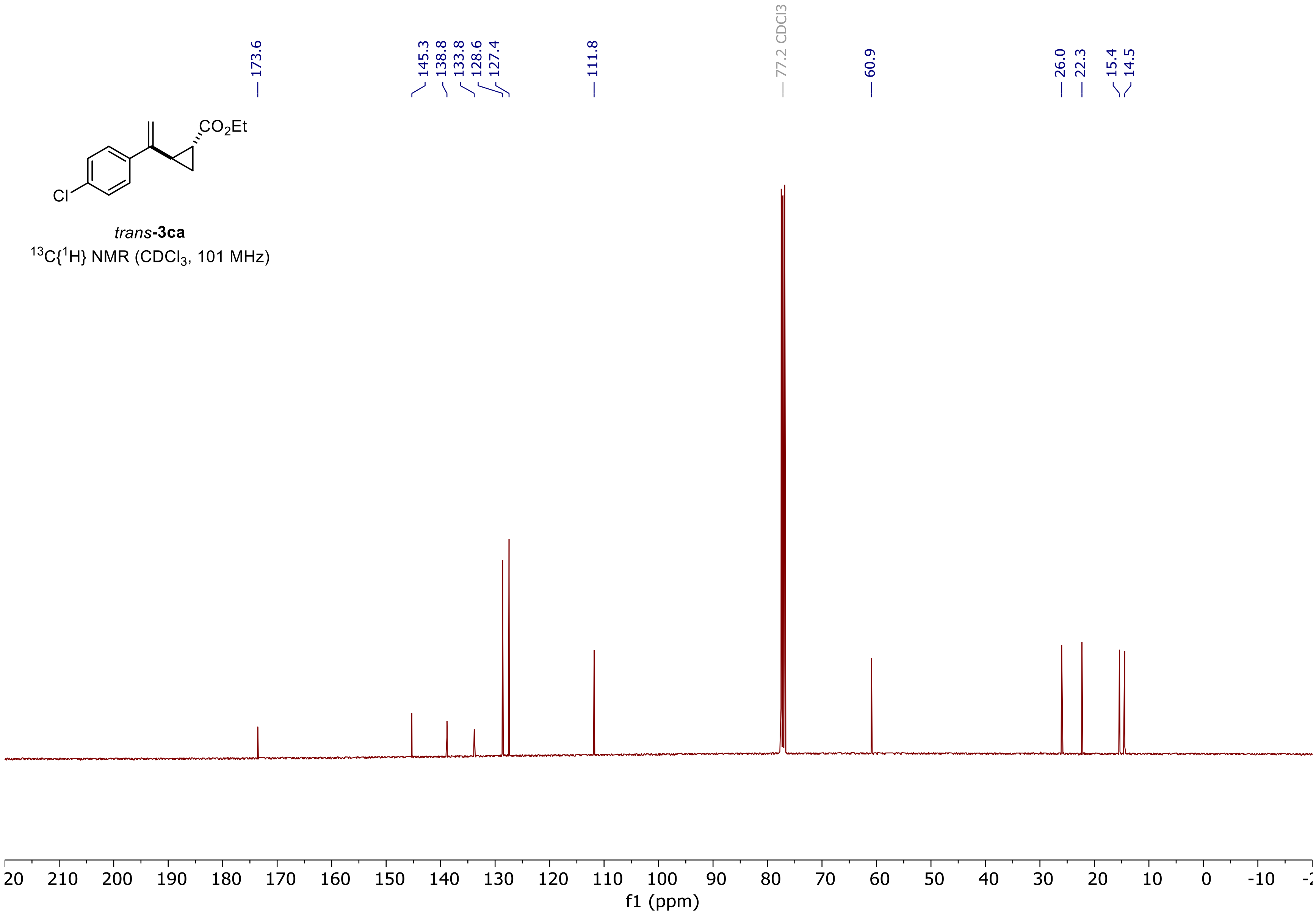

trans-**3ca**
$^{13}C\{^{1}H\}$ NMR ($CDCl_3$, 101 MHz)

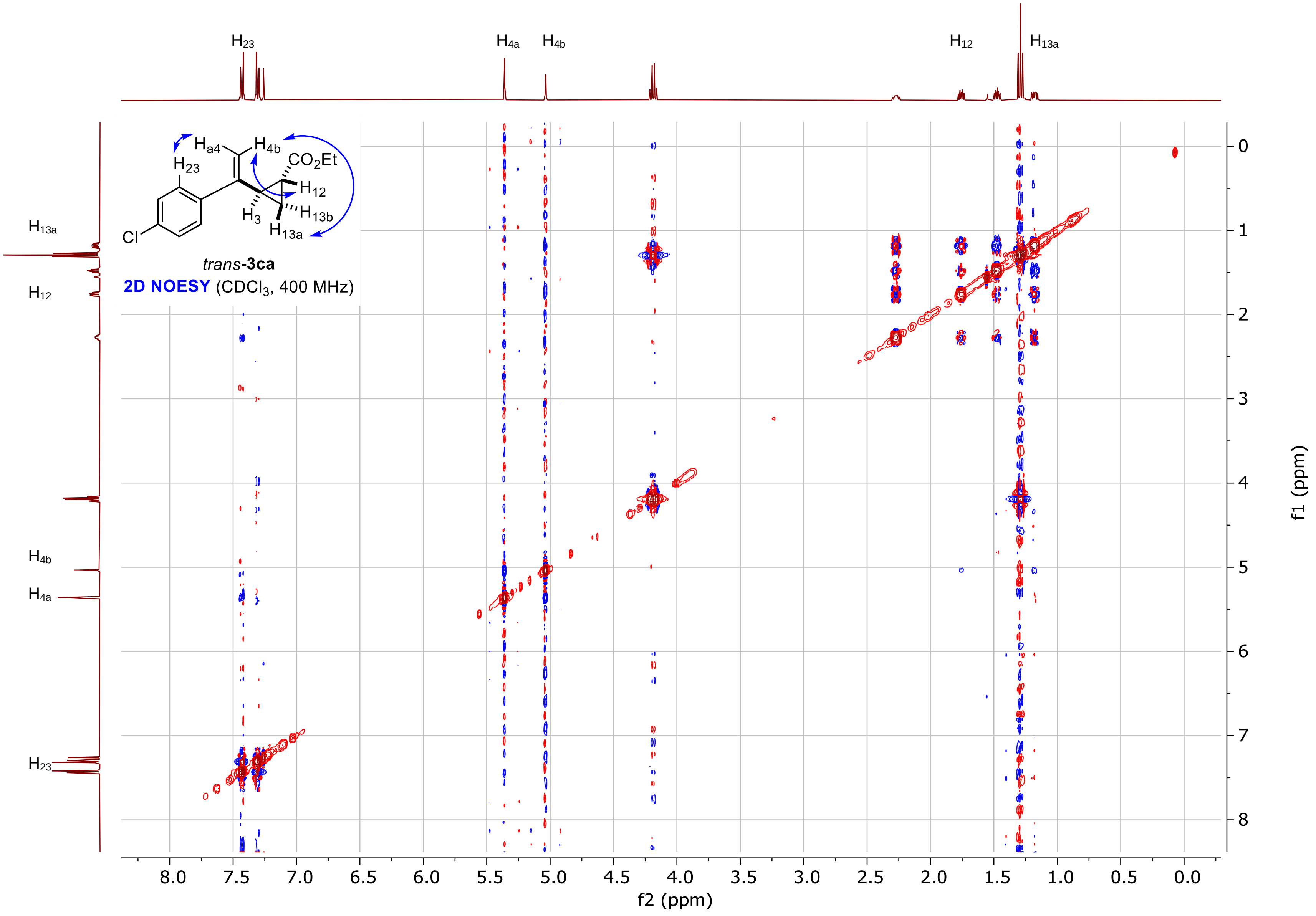

H23
H4a
H4b
H12
H13a
Ha4
CO2Et
H3
H13b
Cl
trans-3ca
2D NOESY (CDCl3, 400 MHz)
f2 (ppm)
f1 (ppm)

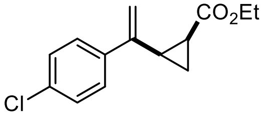

cis-**3ca**

^{1}H NMR ($CDCl_3$, 400 MHz)

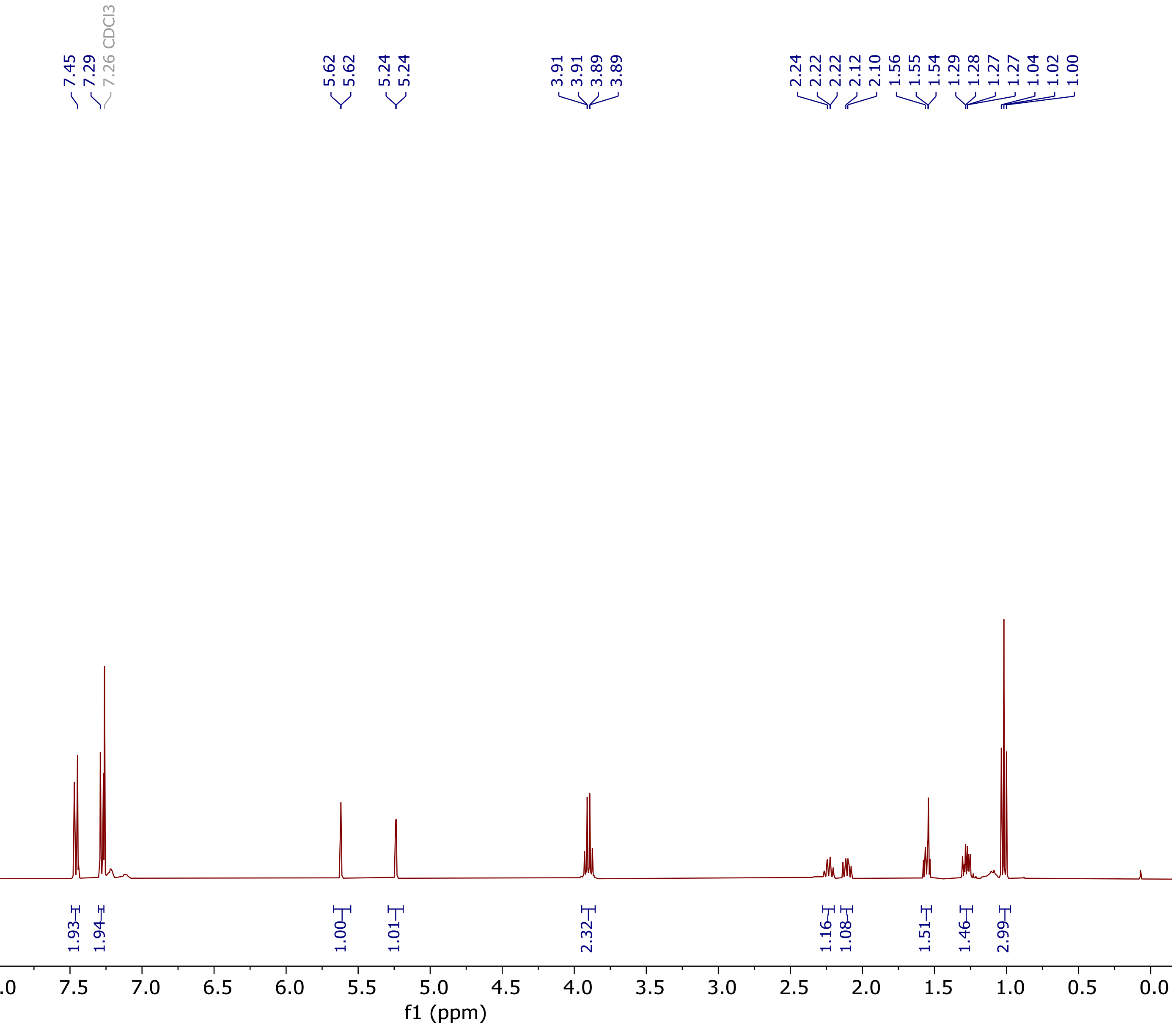

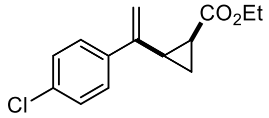

cis-**3ca**

$^{13}C\{^1H\}$ NMR ($CDCl_3$, 101 MHz)

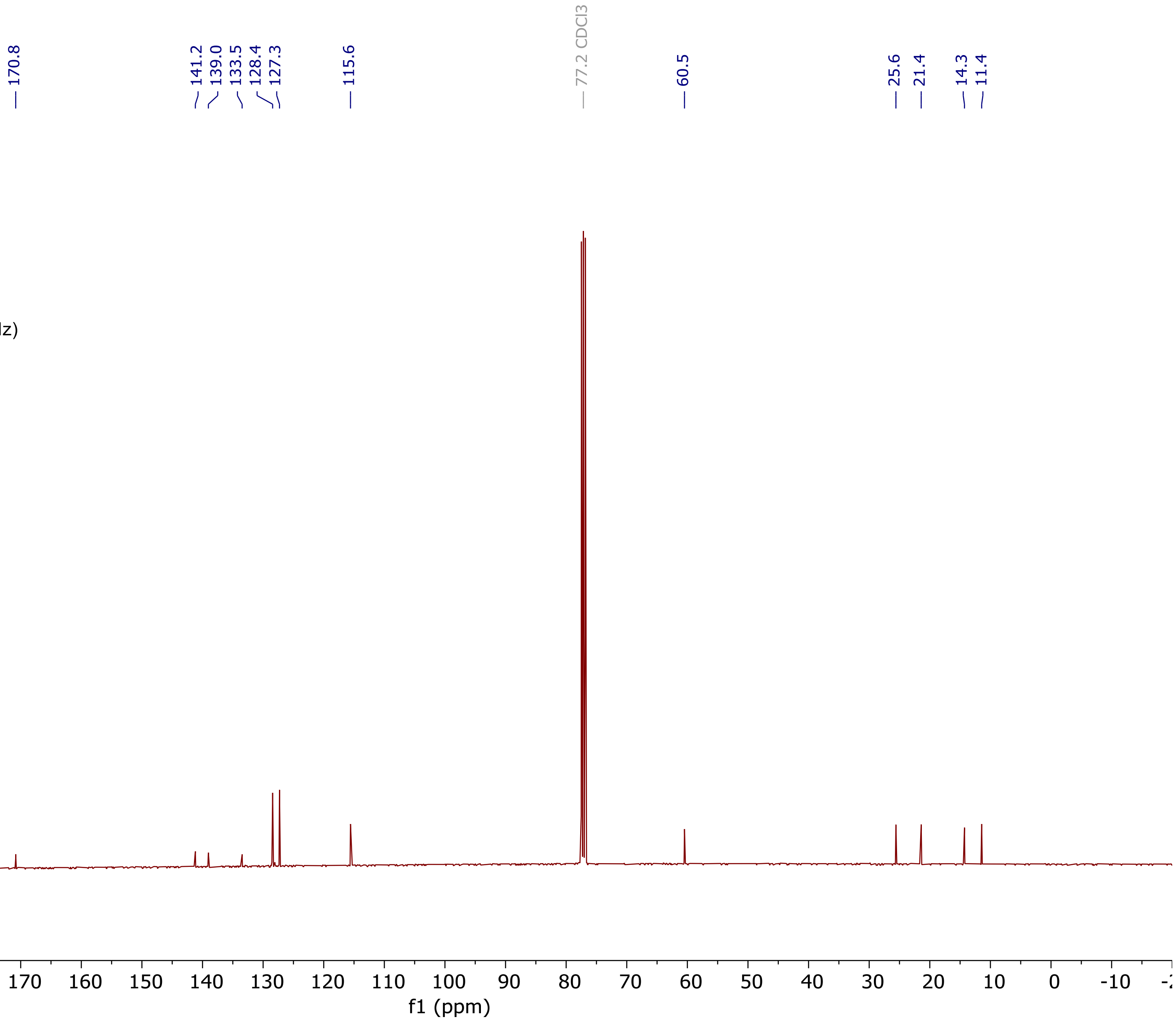

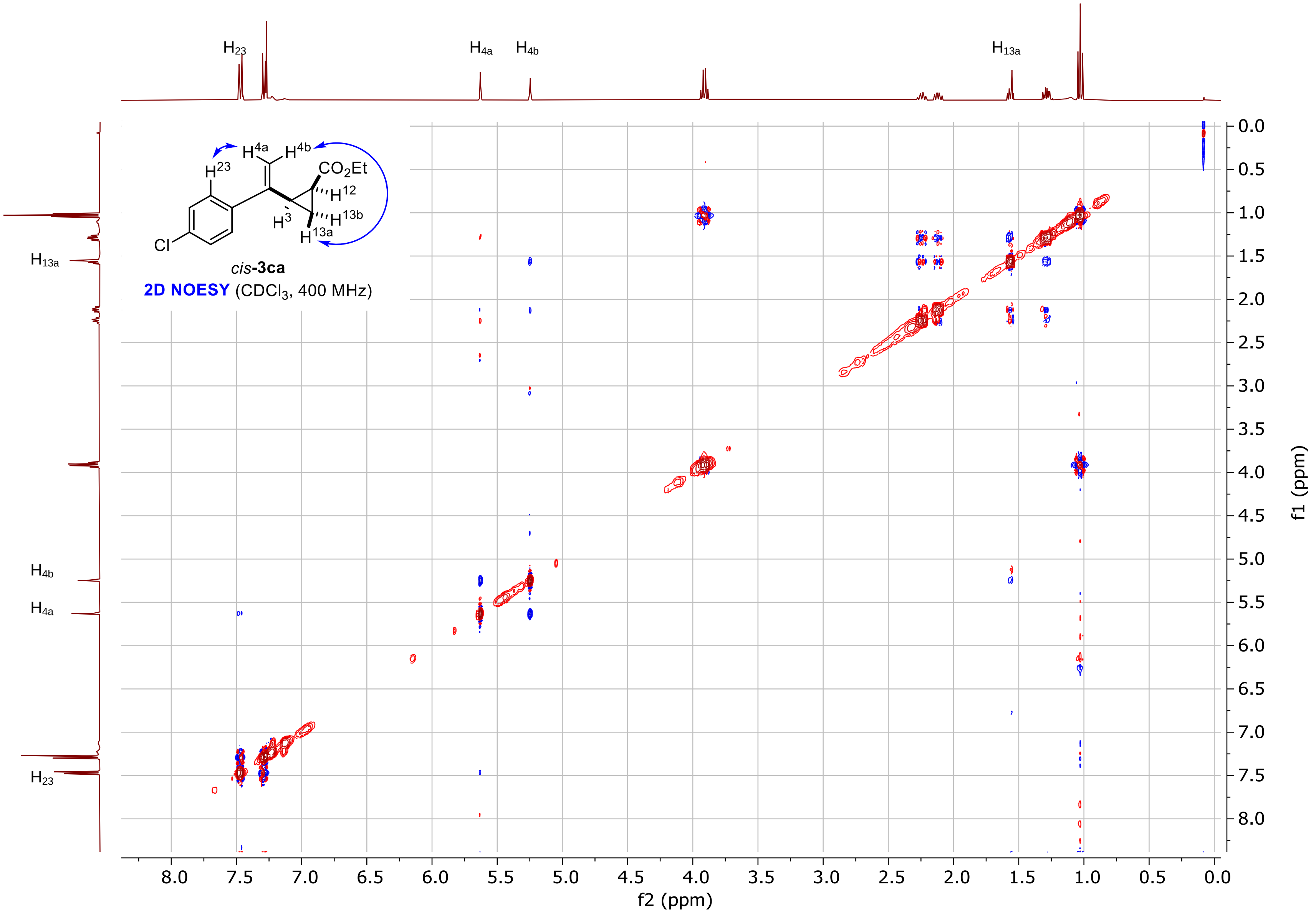
H23
H4a
H4b
H13a
H4a
H4b
H23
CO2Et
H12
H13b
H3
H13a
Cl
cis-3ca
2D NOESY (CDCl3, 400 MHz)
H13a
H4b
H4a
H23
f2 (ppm)
f1 (ppm)

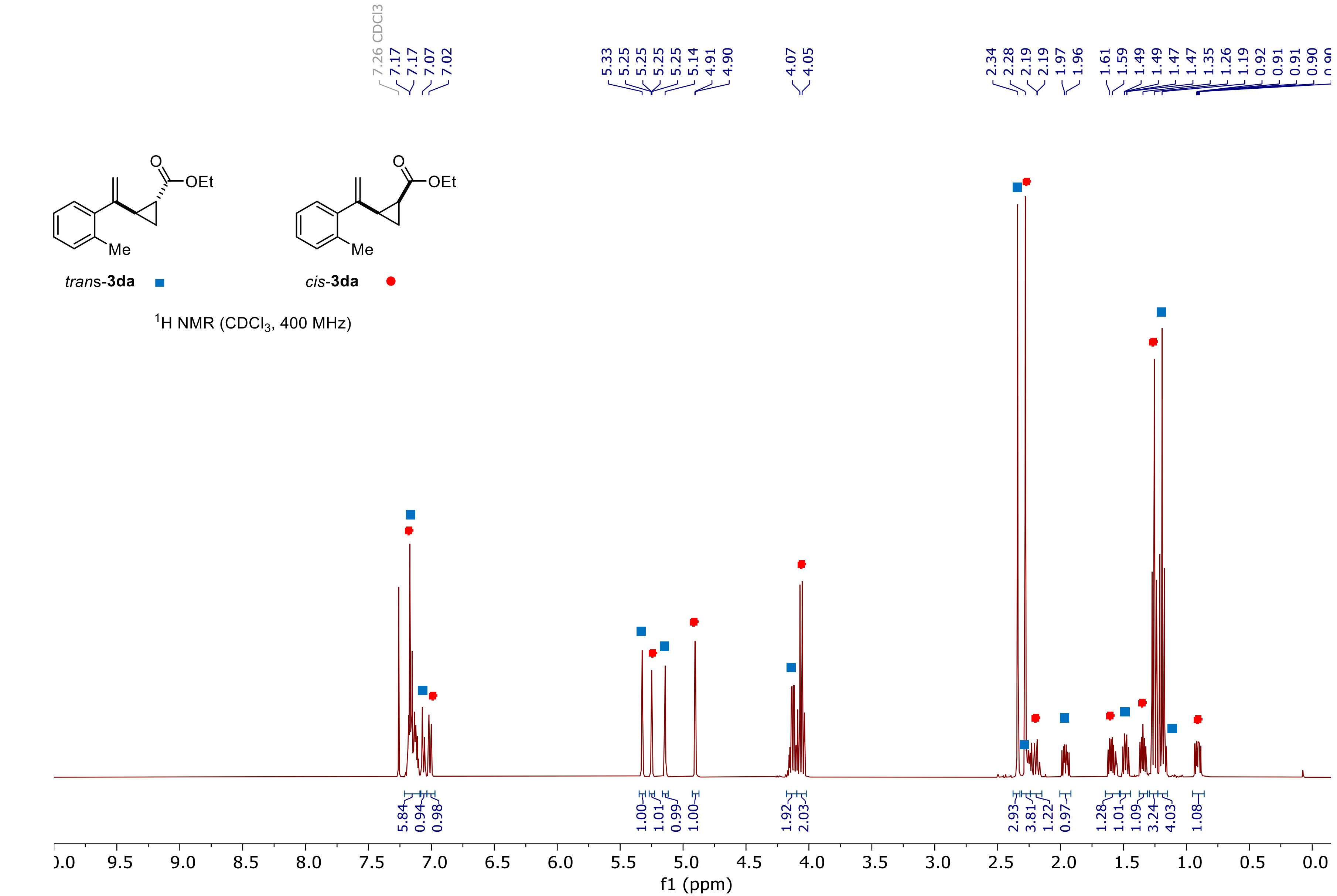

trans-3da
cis-3da
OEt
Me
O
^{1}H NMR (CDCl$_3$, 400 MHz)
7.26 CDCl3
f1 (ppm)

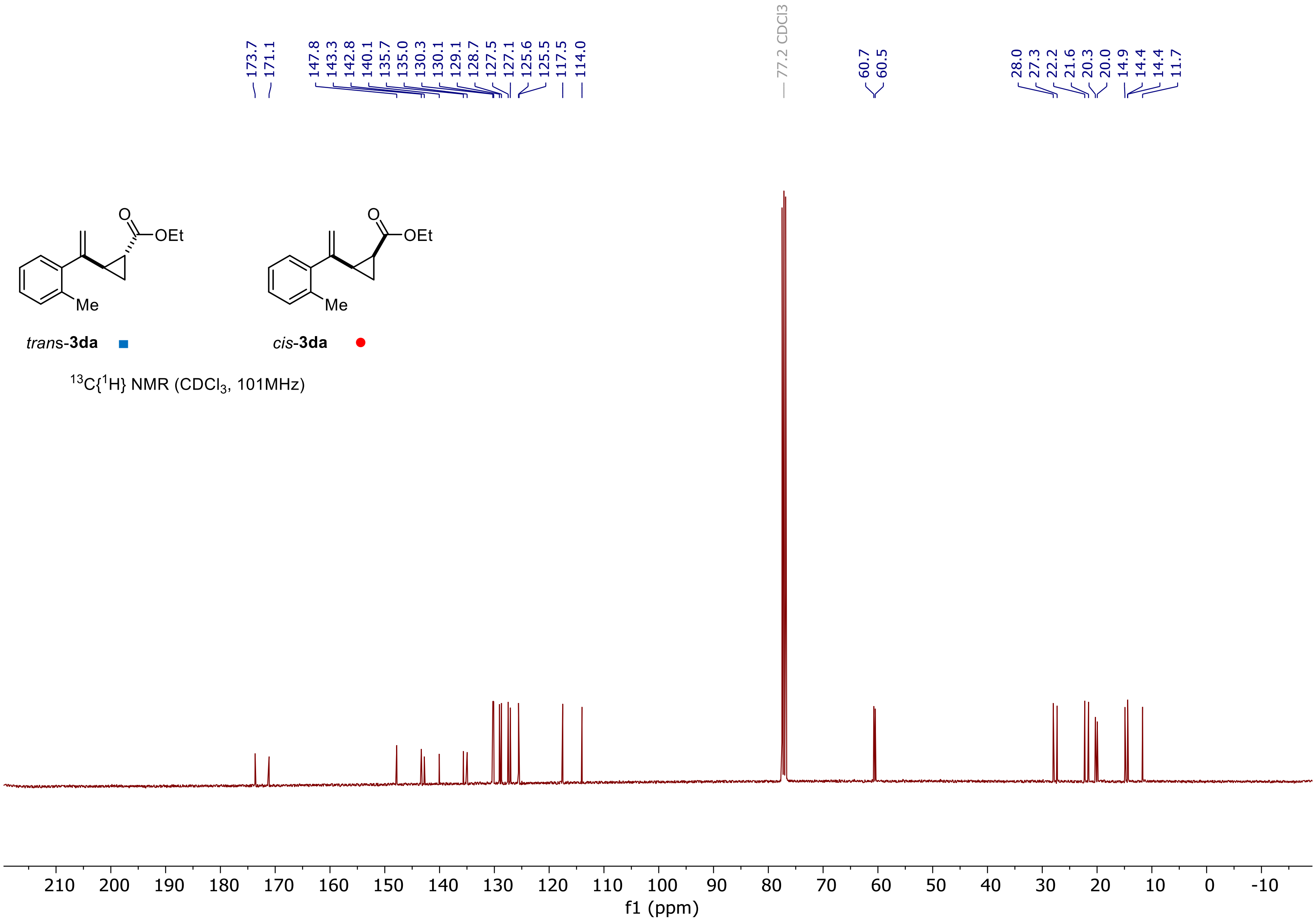
O
OEt
Me
trans-**3da** ■
O
OEt
Me
cis-**3da** ●
$^{13}C\{^{1}H\}$ NMR ($CDCl_3$, 101MHz)
173.7
171.1
147.8
143.3
142.8
140.1
135.7
135.0
130.3
130.1
129.1
128.7
127.5
127.1
125.6
125.5
117.5
114.0
77.2 CDCl3
60.7
60.5
28.0
27.3
22.2
21.6
20.3
20.0
14.9
14.4
14.4
11.7
210 200 190 180 170 160 150 140 130 120 110 100 90 80 70 60 50 40 30 20 10 0 -10
f1 (ppm)

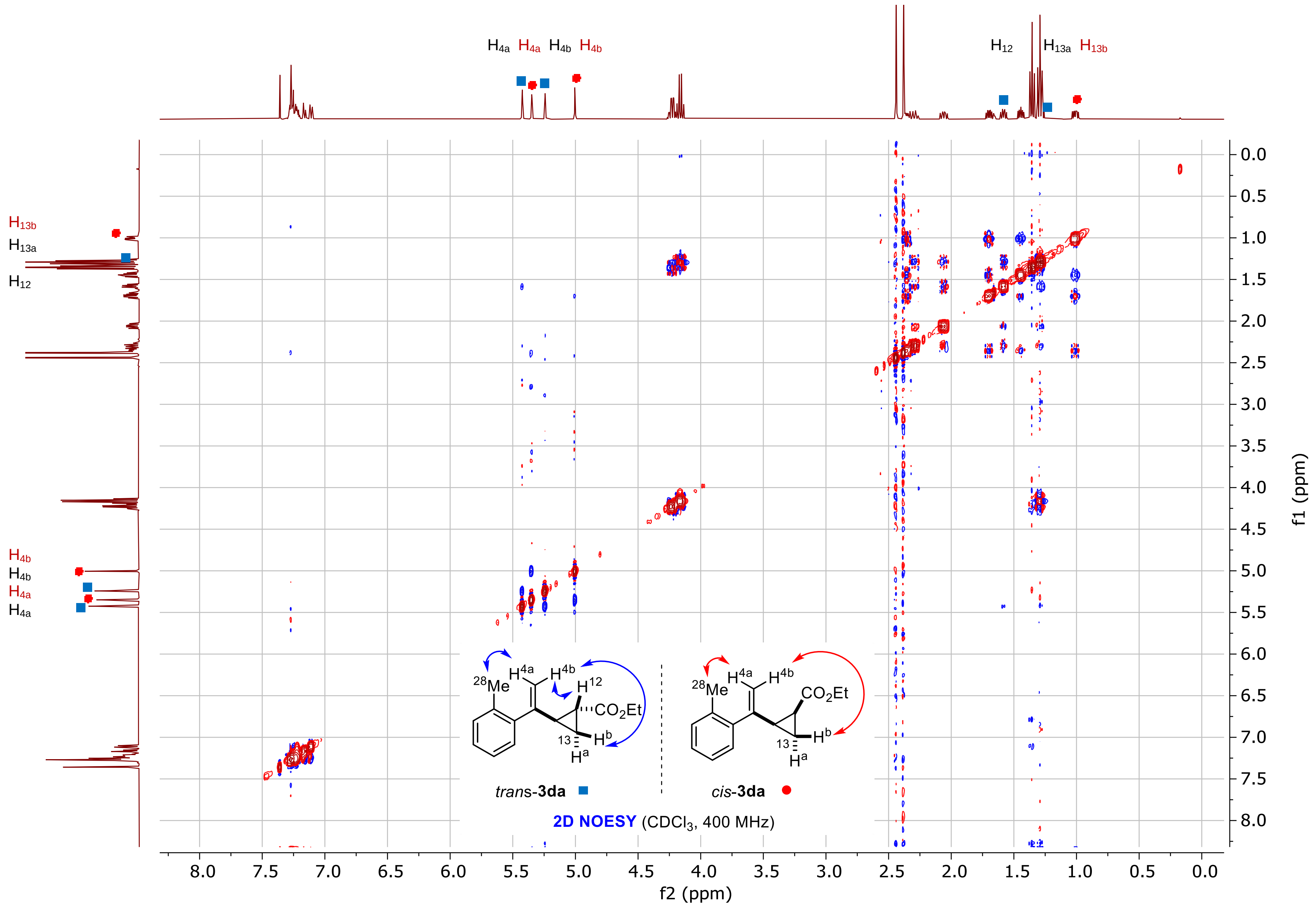
H4a H4a H4b H4b
H12 H13a H13b
H13b
H13a
H12
H4b
H4b
H4a
H4a
28Me
H4a
H4b
H12
CO2Et
13
Hb
Ha
trans-3da
28Me
H4a
H4b
CO2Et
13
Hb
Ha
cis-3da
2D NOESY (CDCl3, 400 MHz)
f2 (ppm)
f1 (ppm)

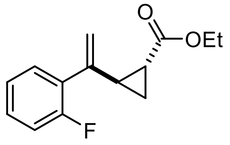

trans-**3ea**

^{1}H NMR ($CDCl_3$, 500 MHz)

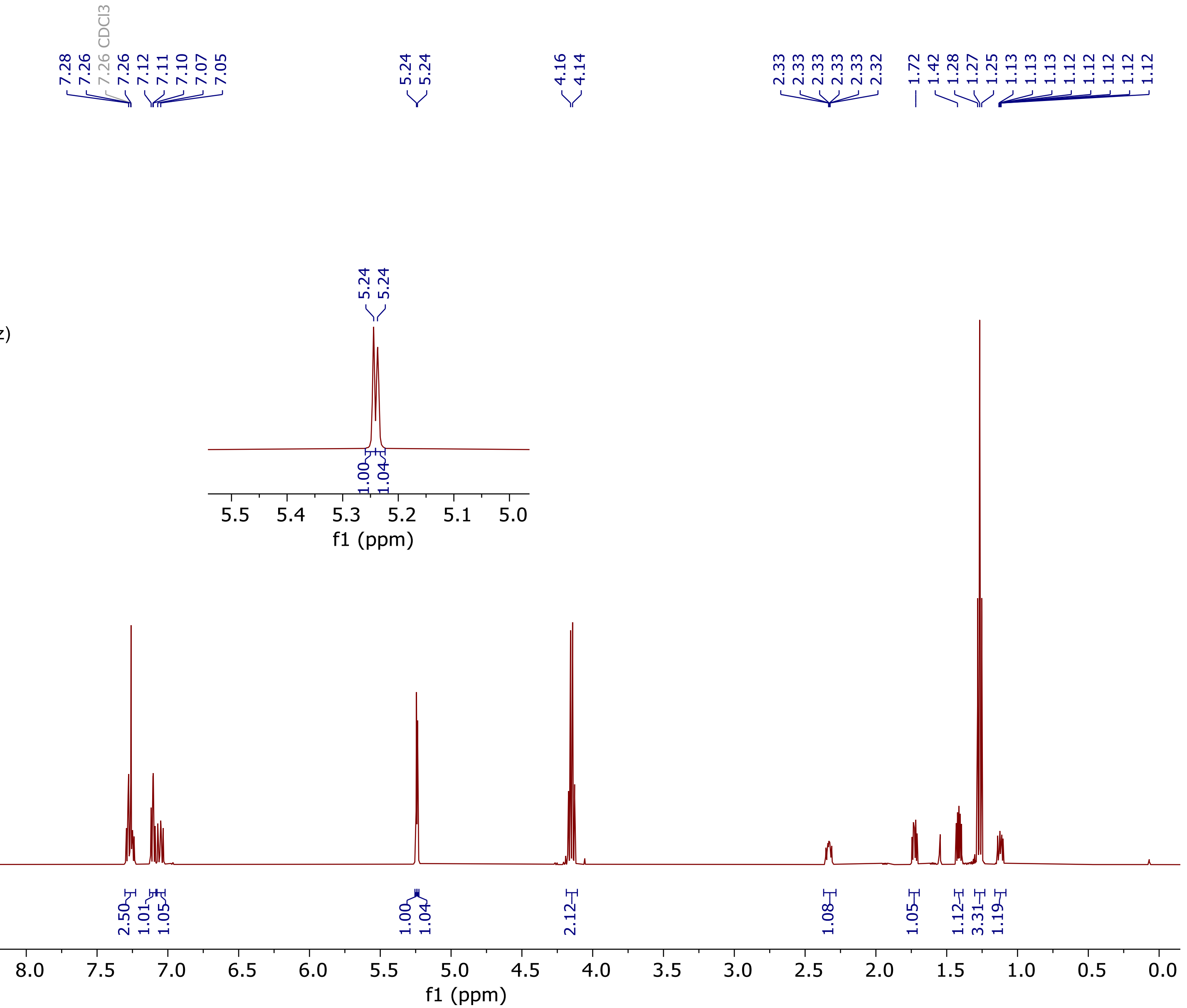

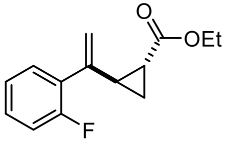

trans-**3ea**

$^{13}C\{^{1}H\}$ NMR ($CDCl_3$, 126 MHz)

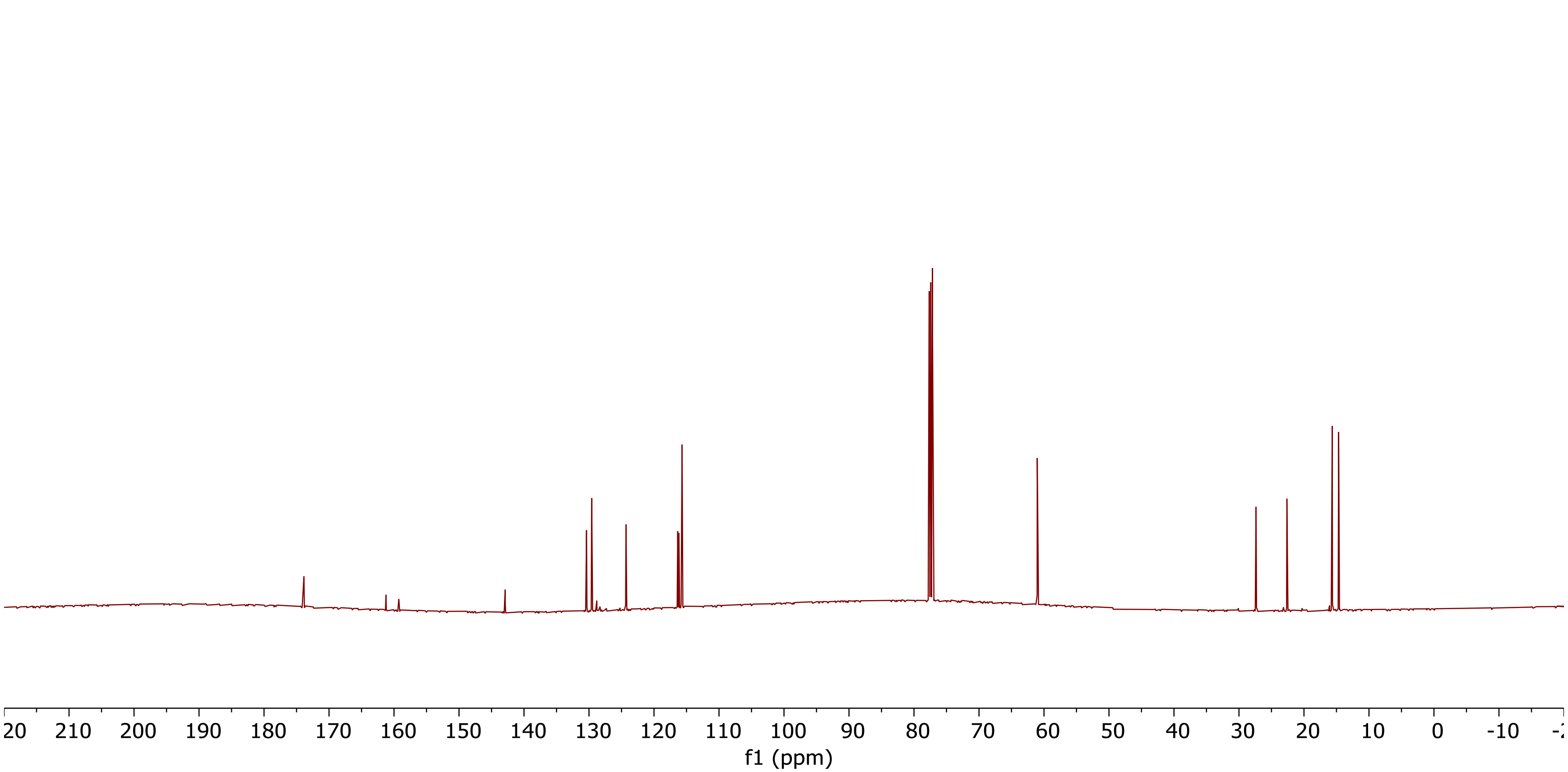

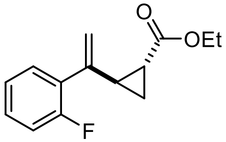

trans-**3ea**
^{19}F NMR ($CDCl_3$, 300 MHz)

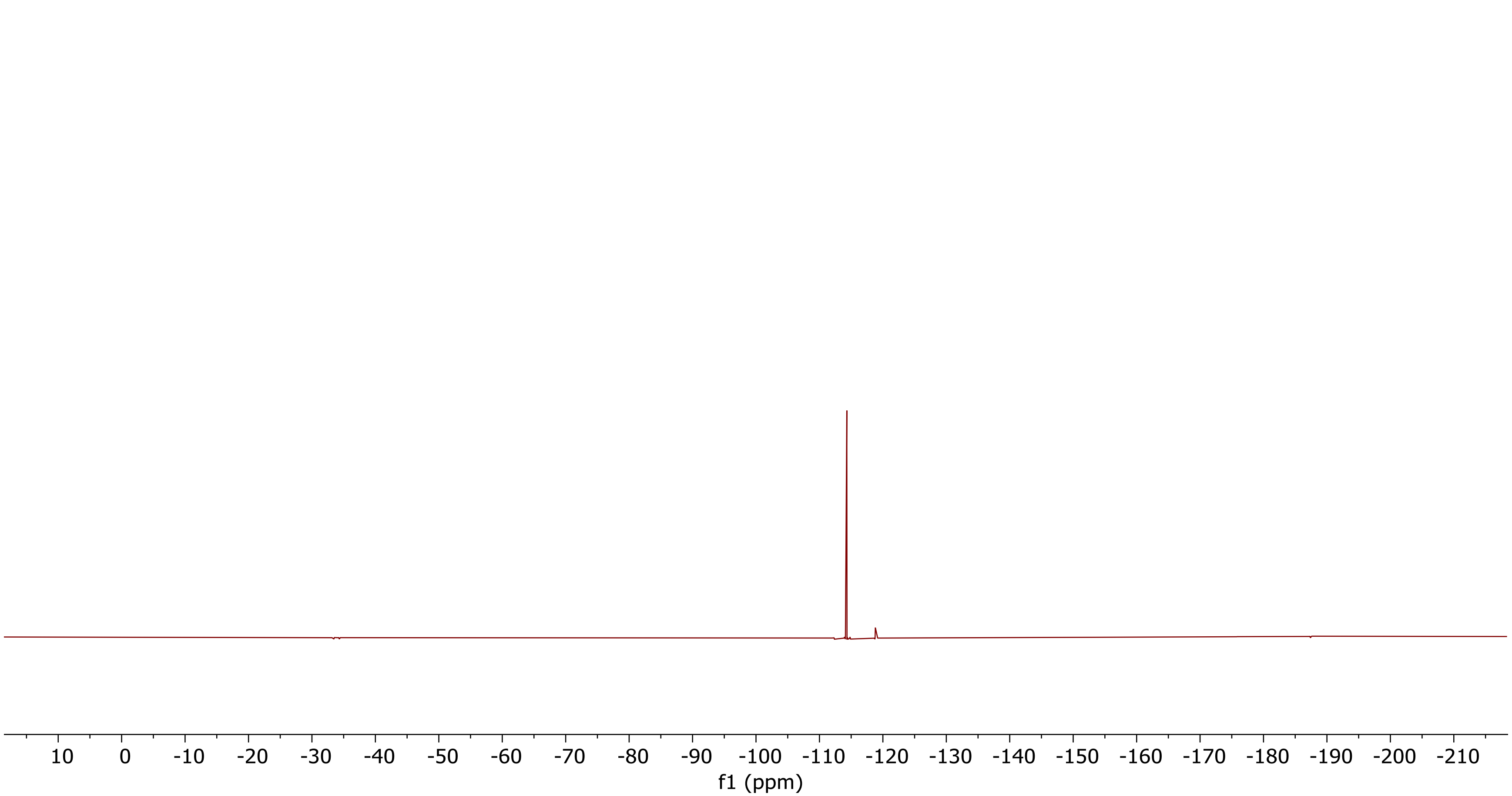

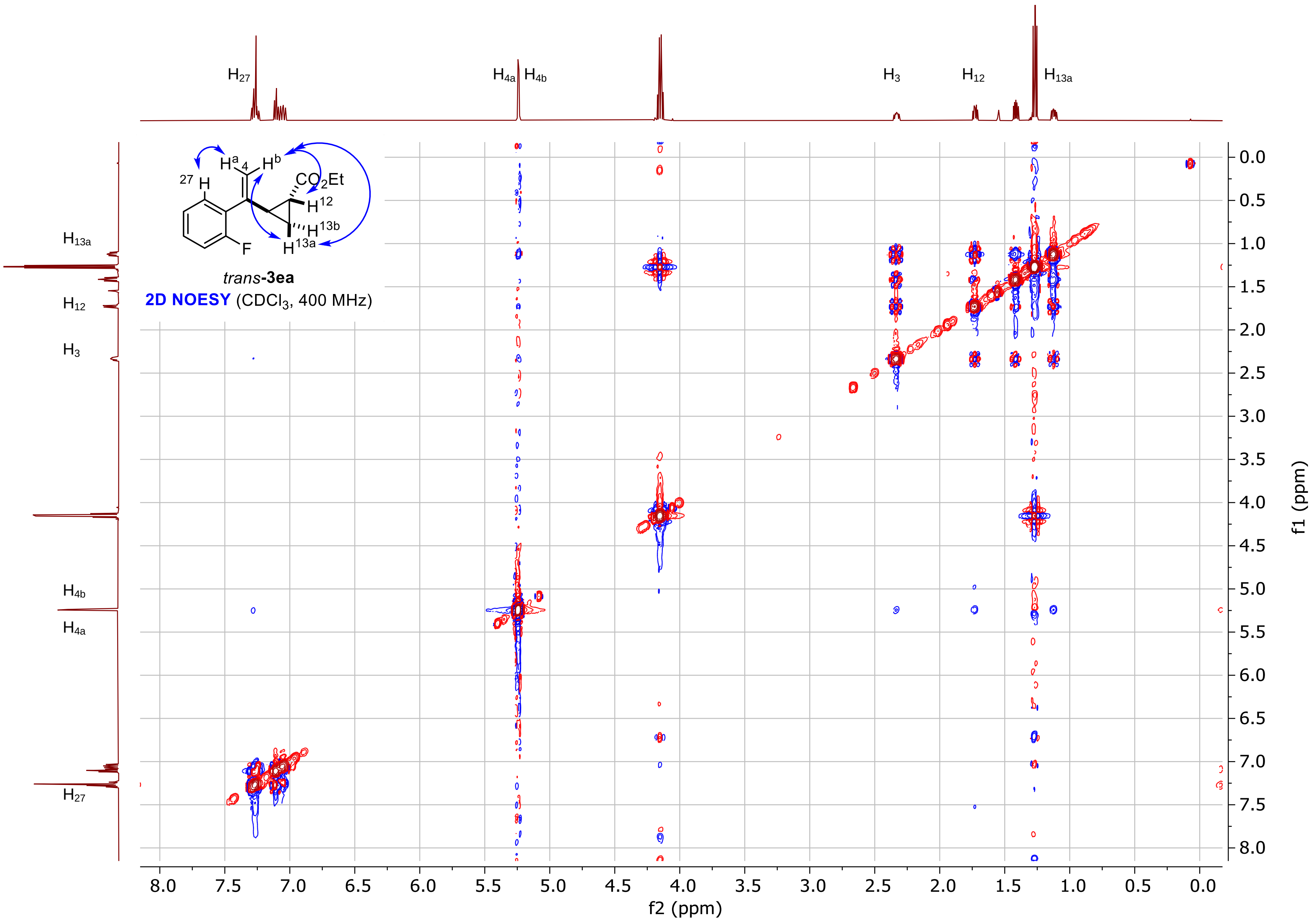
H27
H4a
H4b
H3
H12
H13a
CO2Et
F
trans-3ea
2D NOESY (CDCl3, 400 MHz)
f2 (ppm)
f1 (ppm)

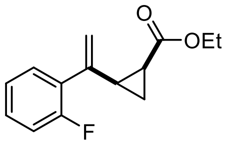

cis-**3ea**

^{1}H NMR (CD_2Cl_2, 400 MHz)

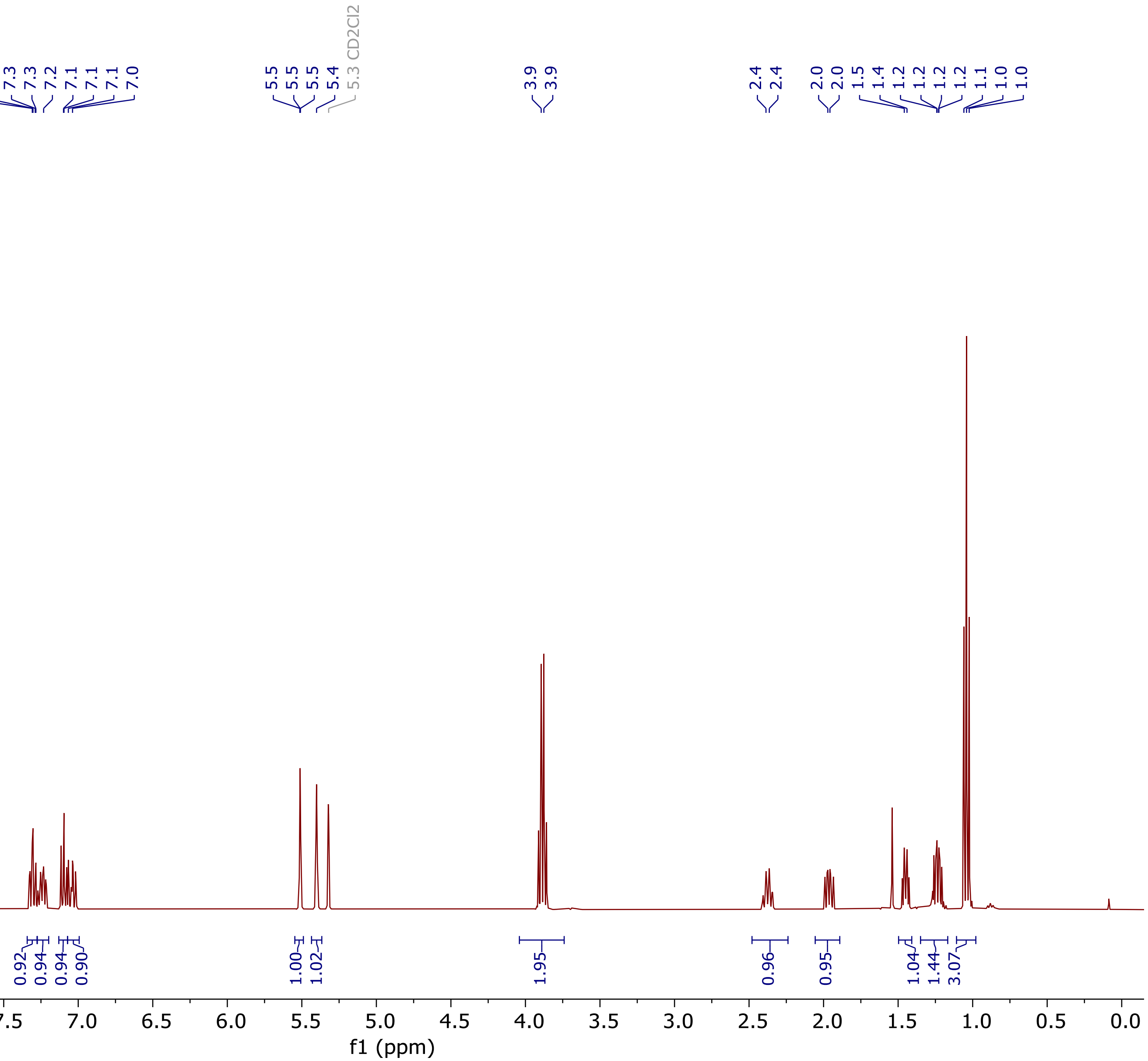

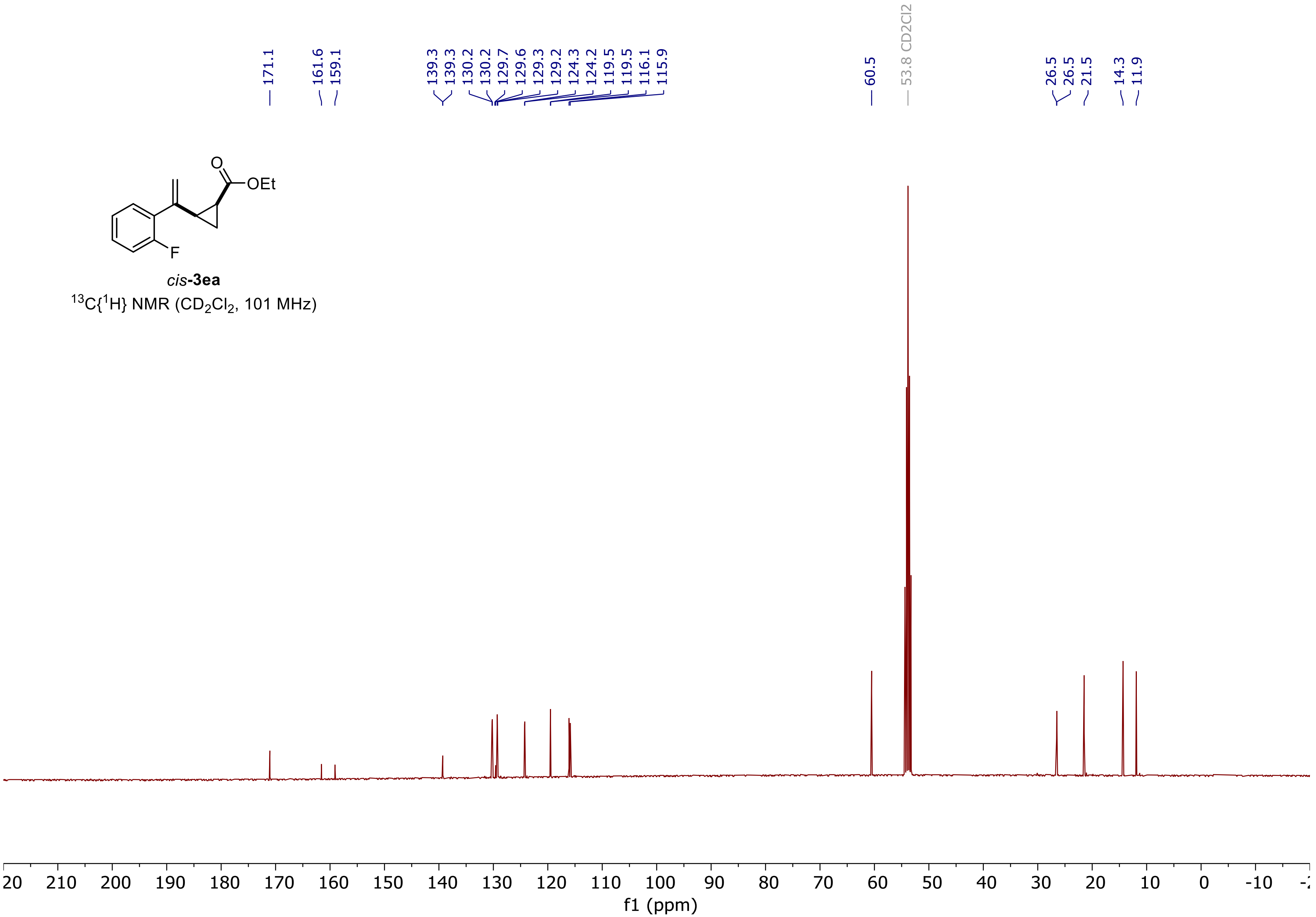

cis-**3ea**

$^{13}C\{^{1}H\}$ NMR (CD_2Cl_2, 101 MHz)

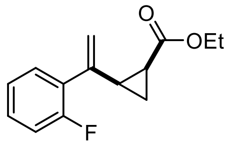

cis-**3ea**
$^{19}F\{^{1}H\}$ NMR (282 MHz, CD_2Cl_2)

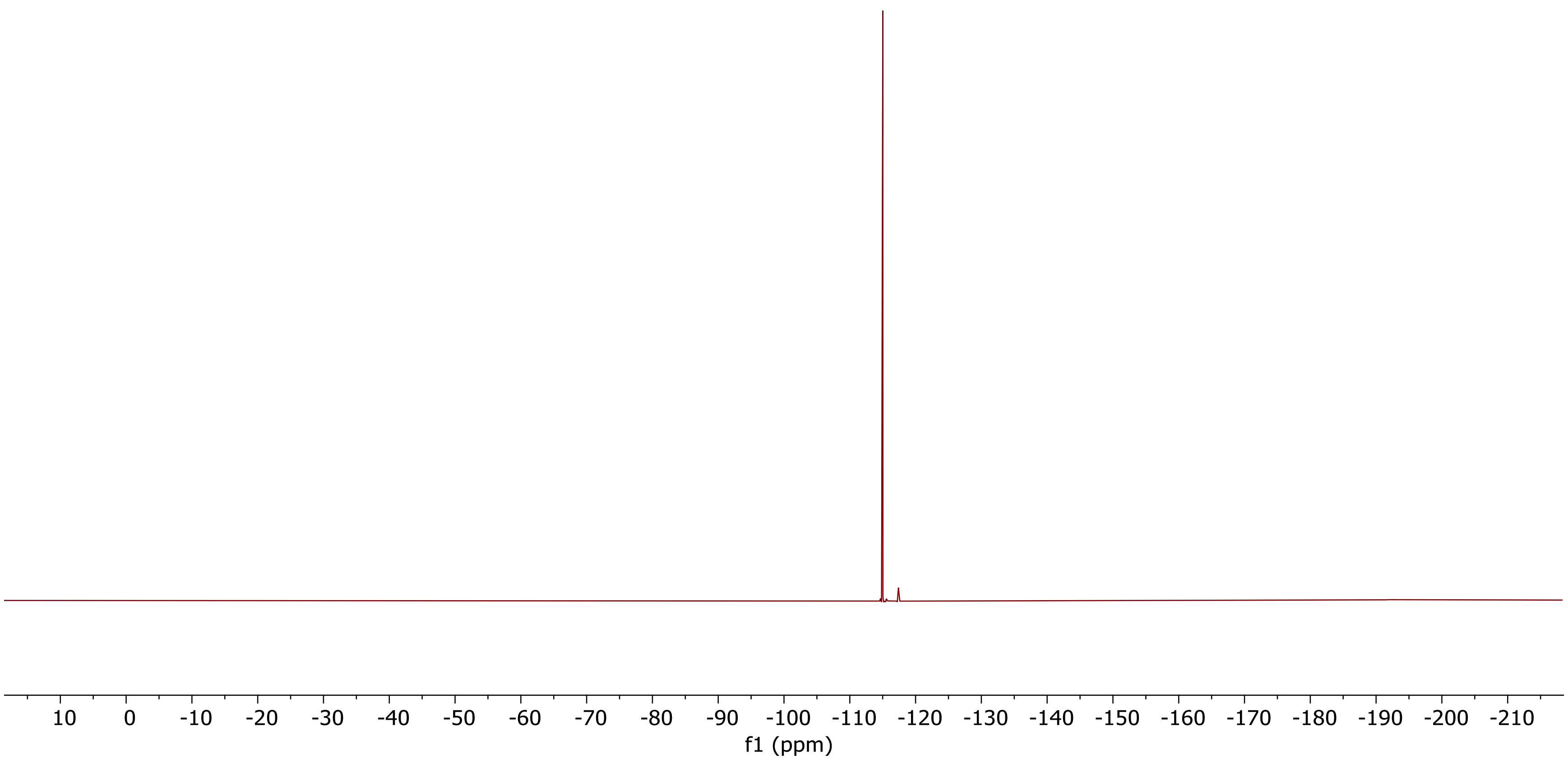

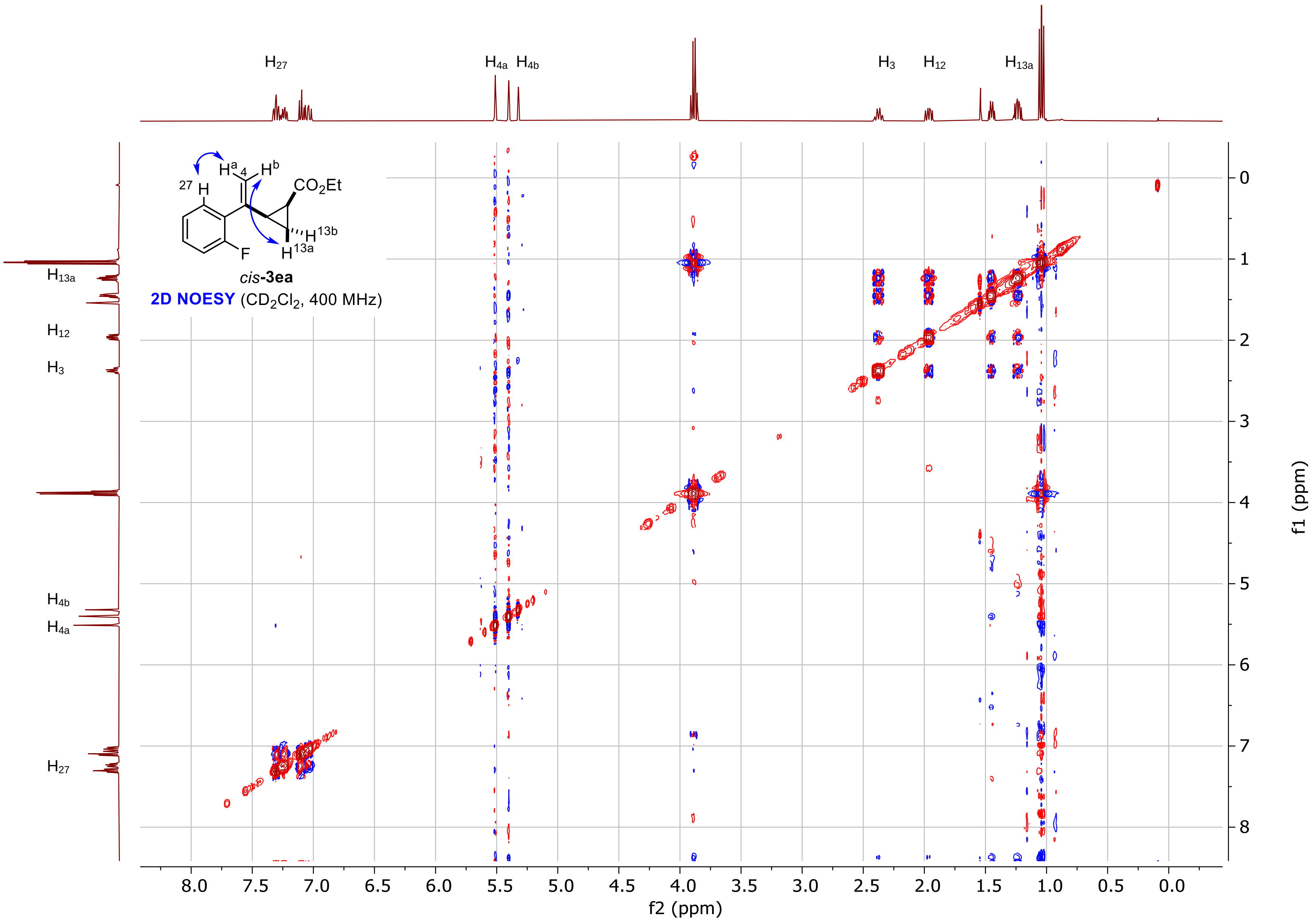
H27
H4a H4b
H3 H12 H13a
27 H
Ha 4 Hb
CO2Et
H13b
H13a
F
cis-3ea
2D NOESY (CD2Cl2, 400 MHz)
H13a
H12
H3
H4b
H4a
H27
f1 (ppm)
f2 (ppm)

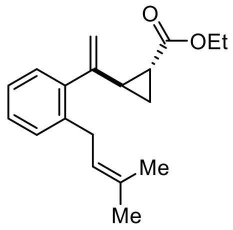

trans-**3fa**

^{1}H NMR ($CDCl_3$, 400 MHz)

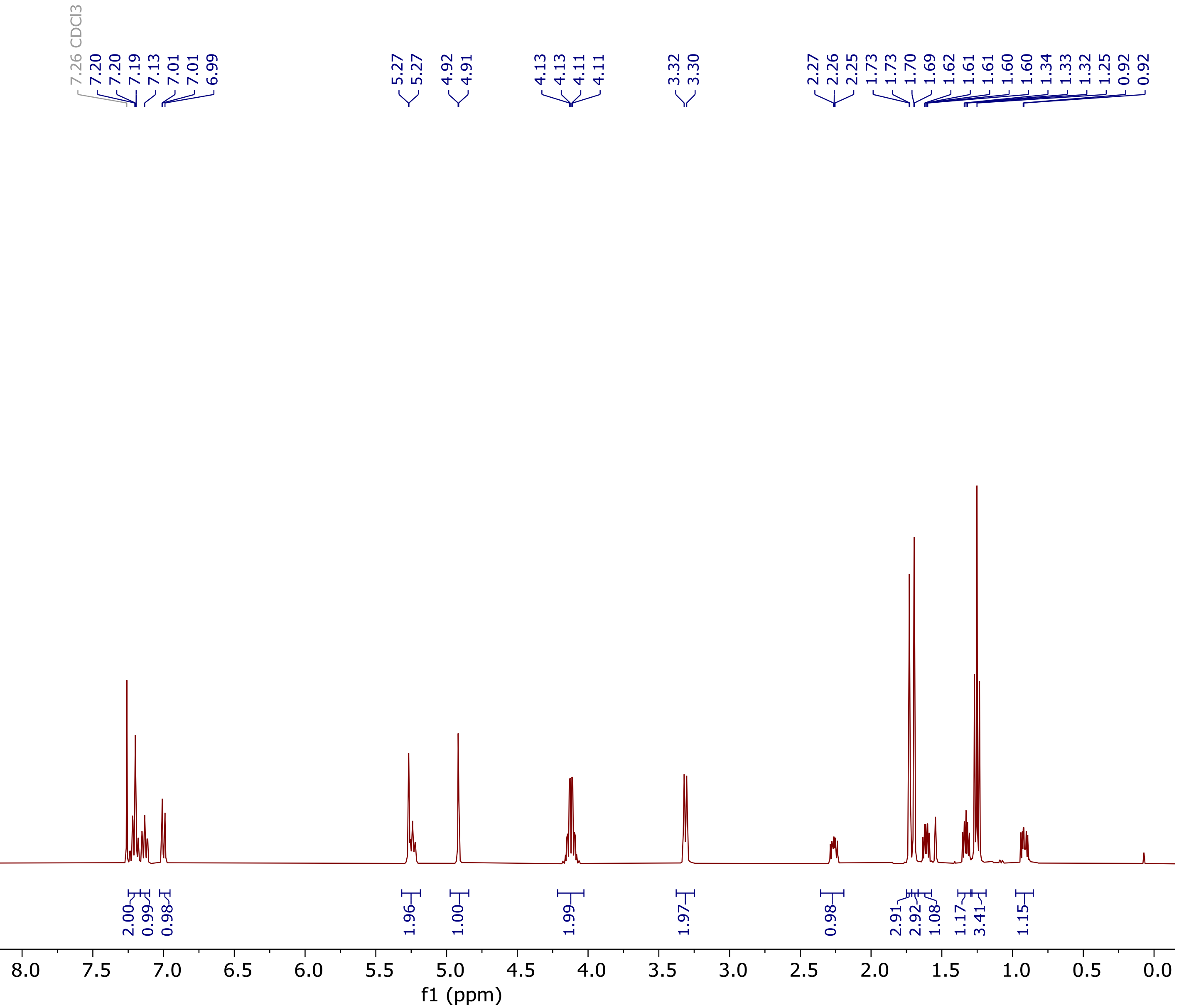

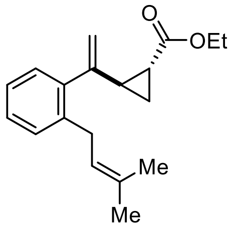

trans-**3fa**

$^{13}C\{^{1}H\}$ NMR (CD_2Cl_2, 101 MHz)

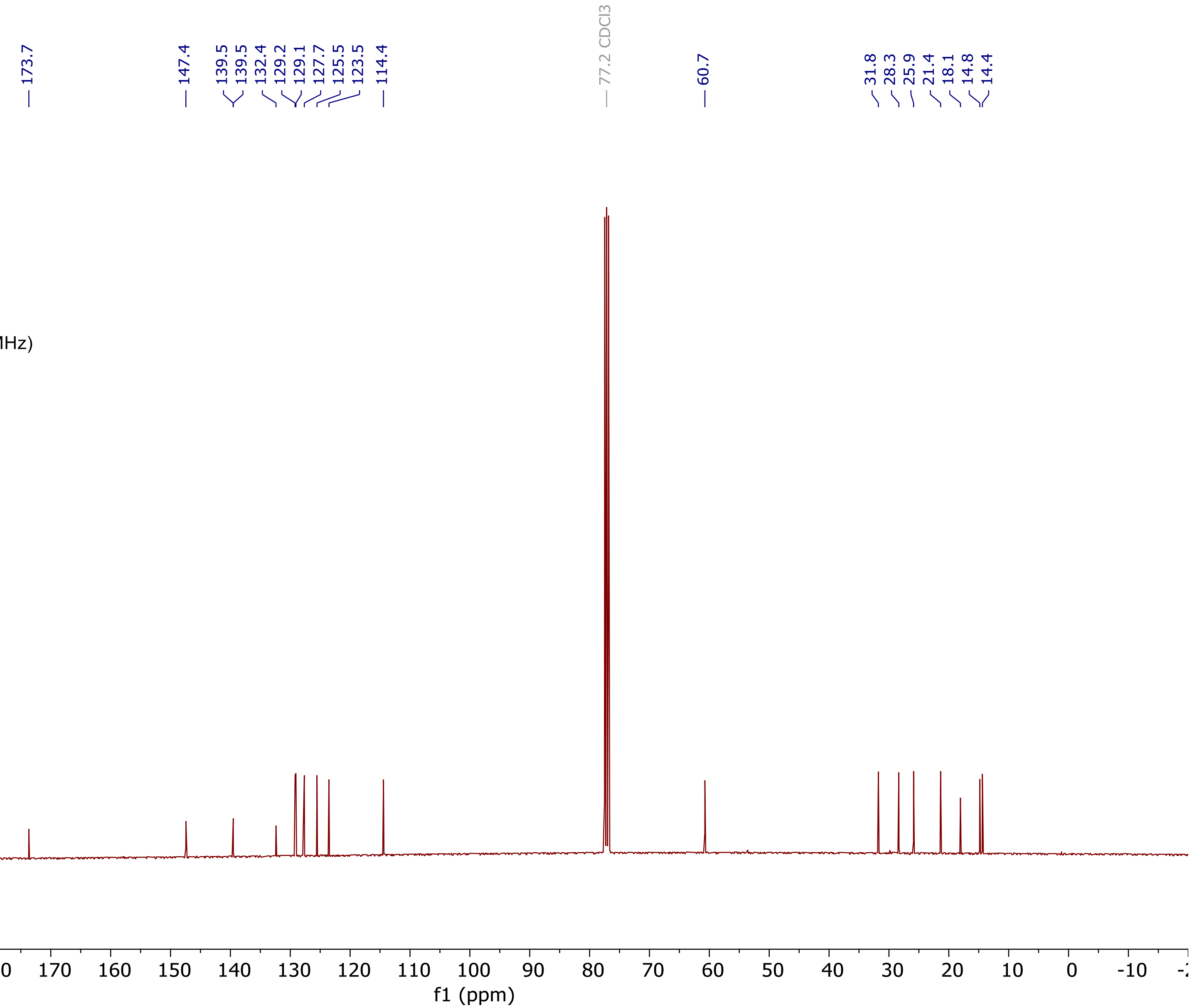

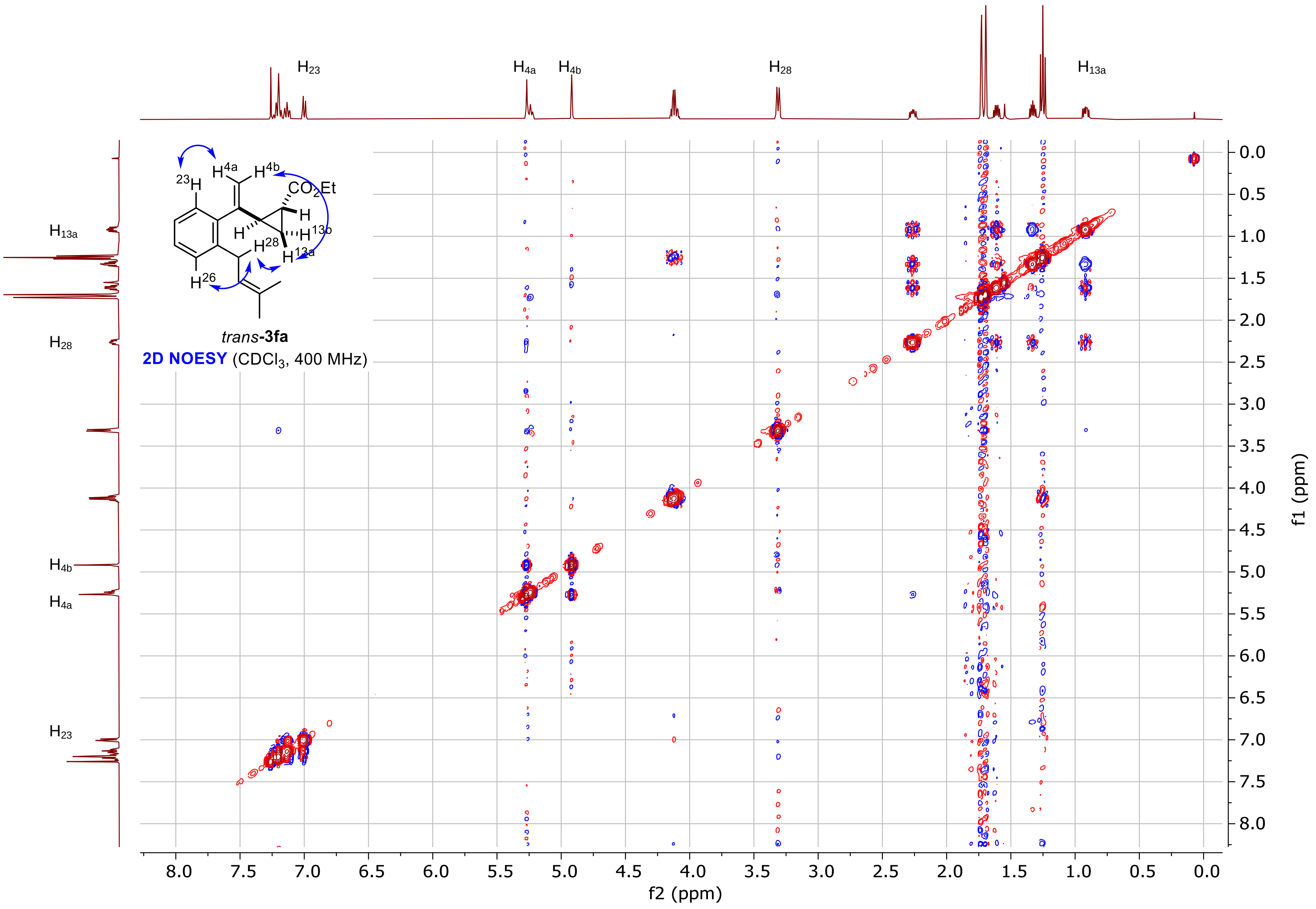

H23
H4a
H4b
H28
H13a
23H
H4a
H4b
CO2Et
H
H
H28
H13b
H13a
H26
trans-**3fa**
2D NOESY (CDCl3, 400 MHz)
f2 (ppm)
f1 (ppm)

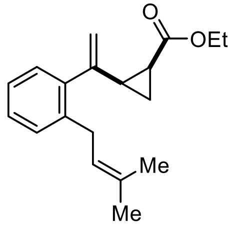

cis-**3fa**

^{1}H NMR ($CDCl_3$, 400 MHz)

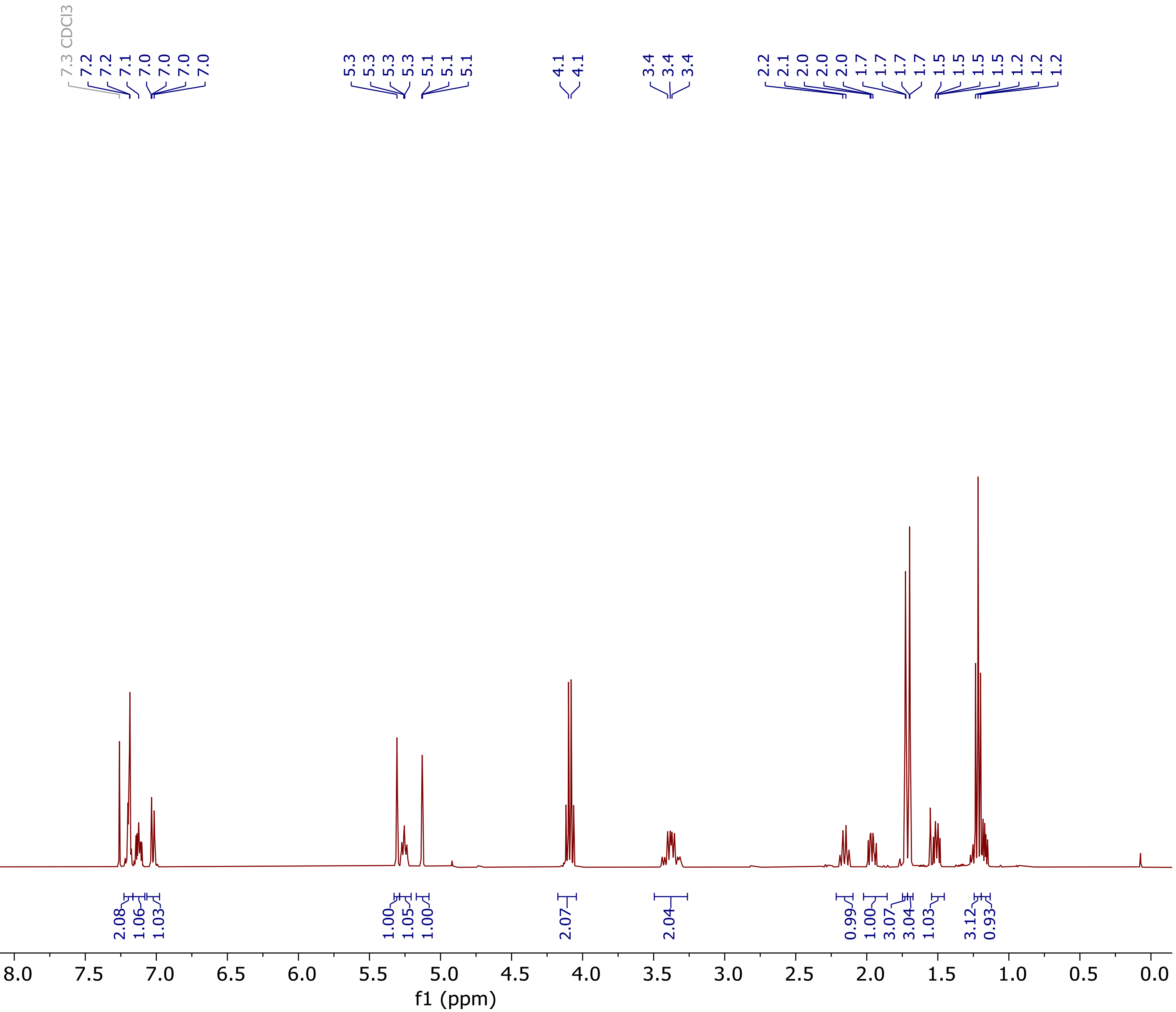

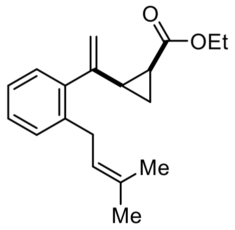

cis-**3fa**

$^{13}C\{^1H\}$ NMR (CD_2Cl_2, 101 MHz)

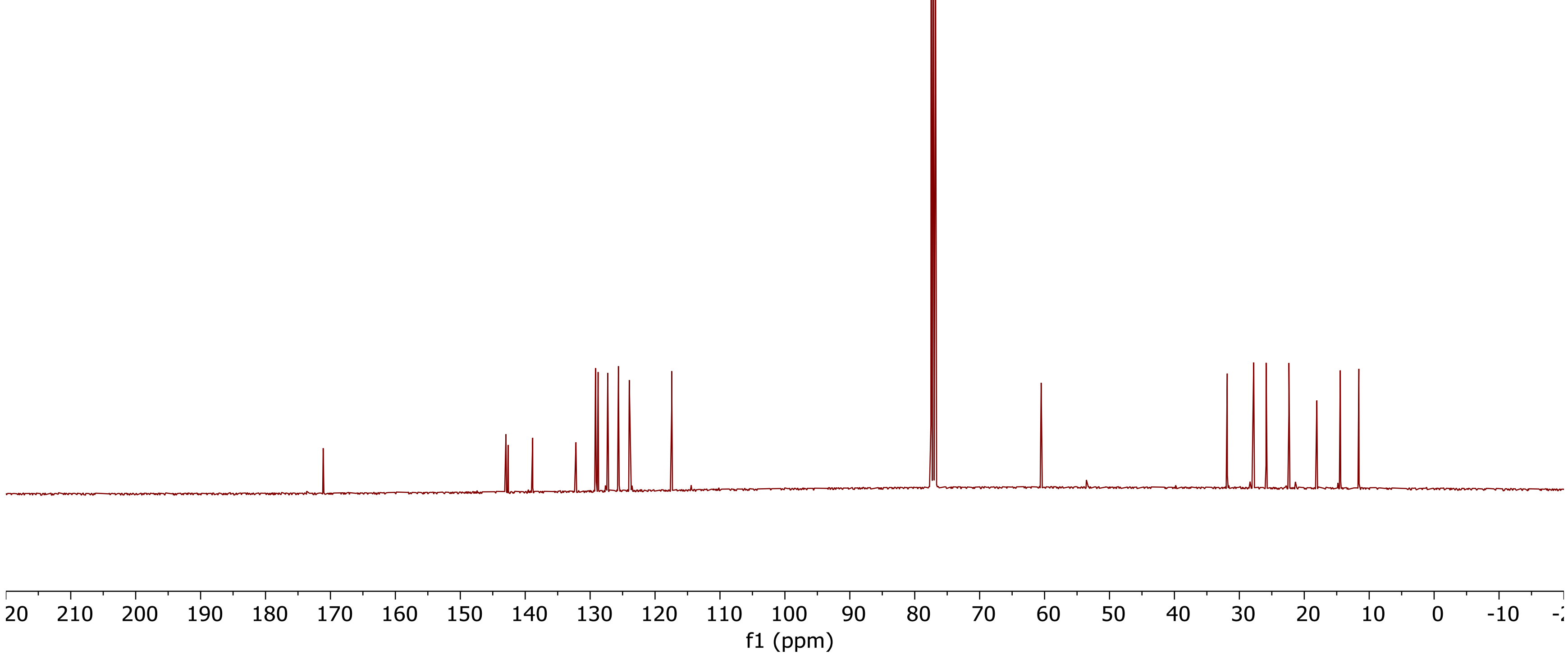

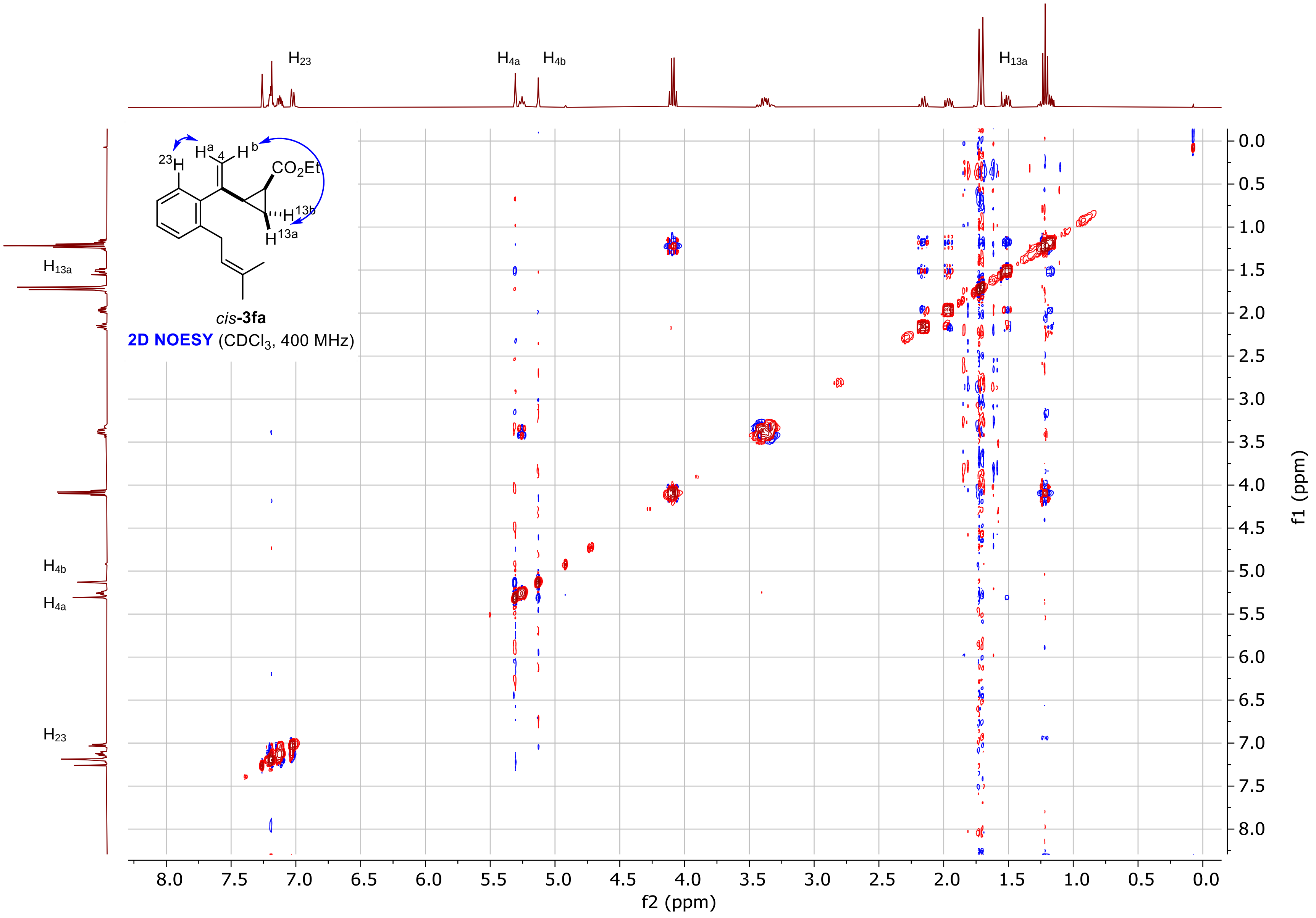

cis-3fa
2D NOESY (CDCl3, 400 MHz)
H23
H4a
H4b
H13a
H13b
CO2Et
f2 (ppm)
f1 (ppm)

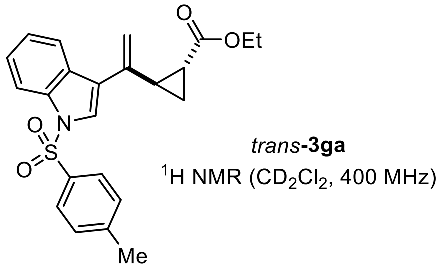

trans-**3ga**

^{1}H NMR (CD_2Cl_2, 400 MHz)

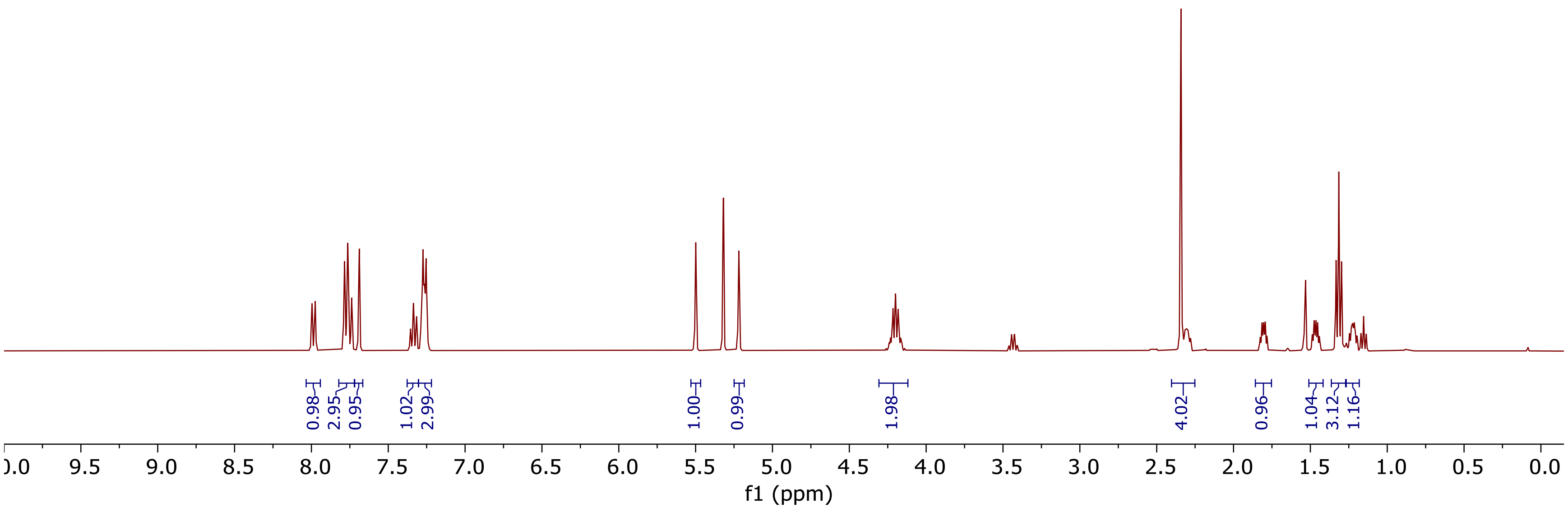

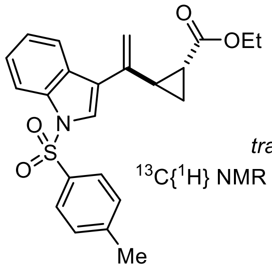

trans-**3ga**

$^{13}C\{^{1}H\}$ NMR (CD_2Cl_2, 101 MHz)

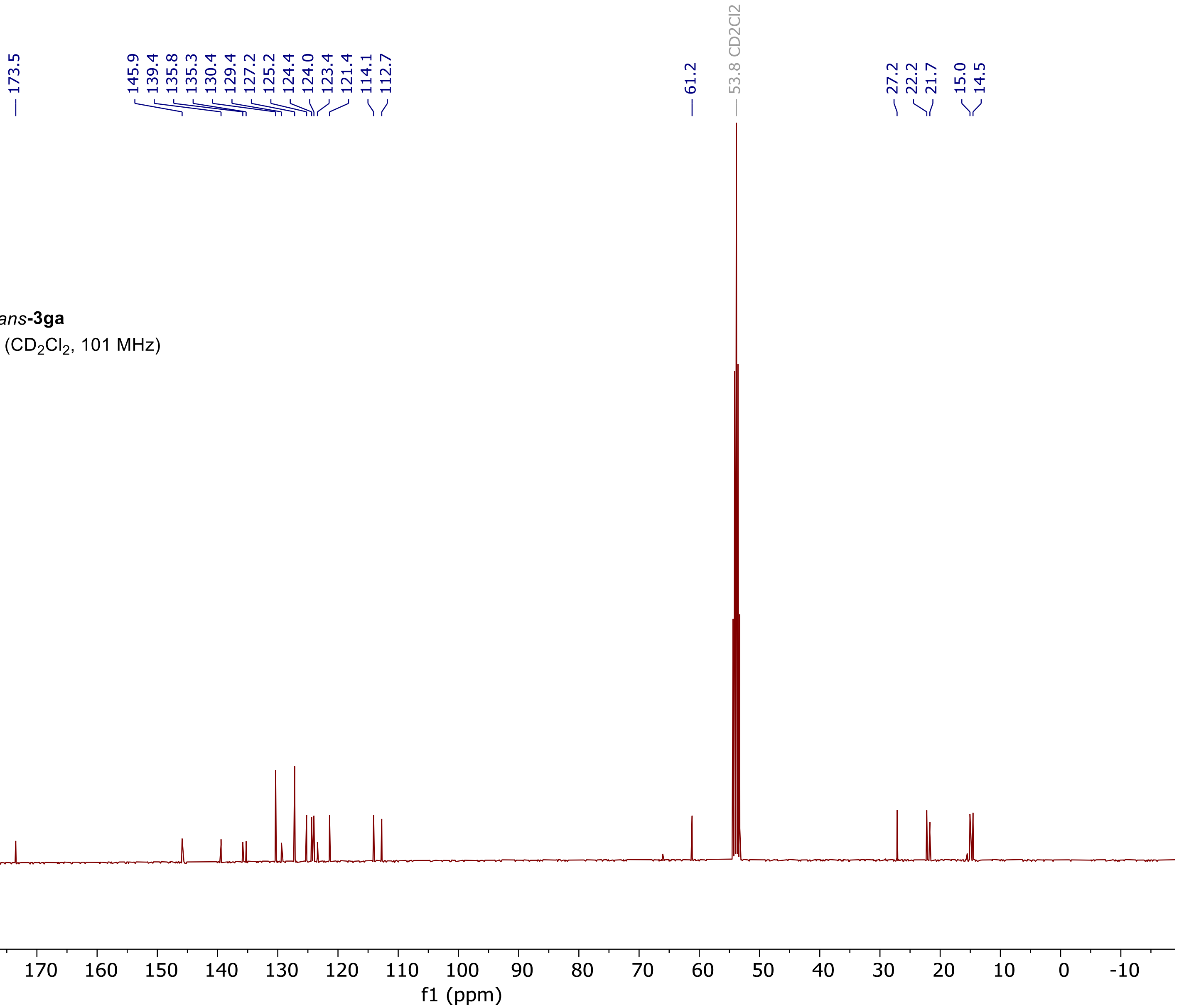

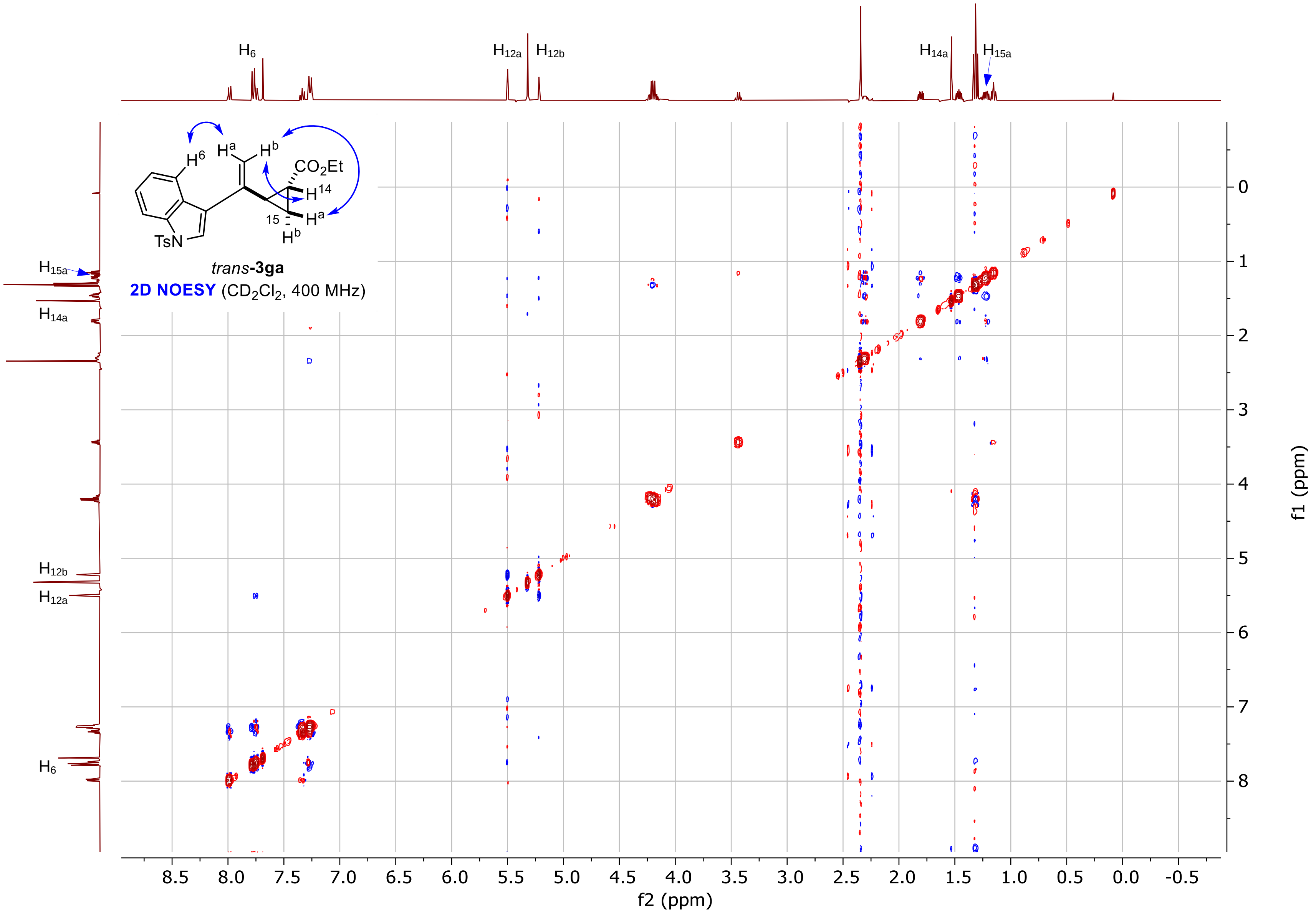
H6
H12a
H12b
H14a
H15a
TsN
Ha
Hb
CO2Et
H14
15
trans-3ga
2D NOESY (CD2Cl2, 400 MHz)
f2 (ppm)
f1 (ppm)

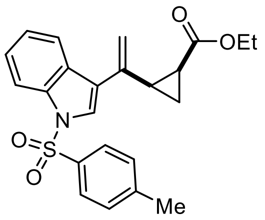

cis-**3ga**

^{1}H NMR (CD_2Cl_2, 400 MHz)

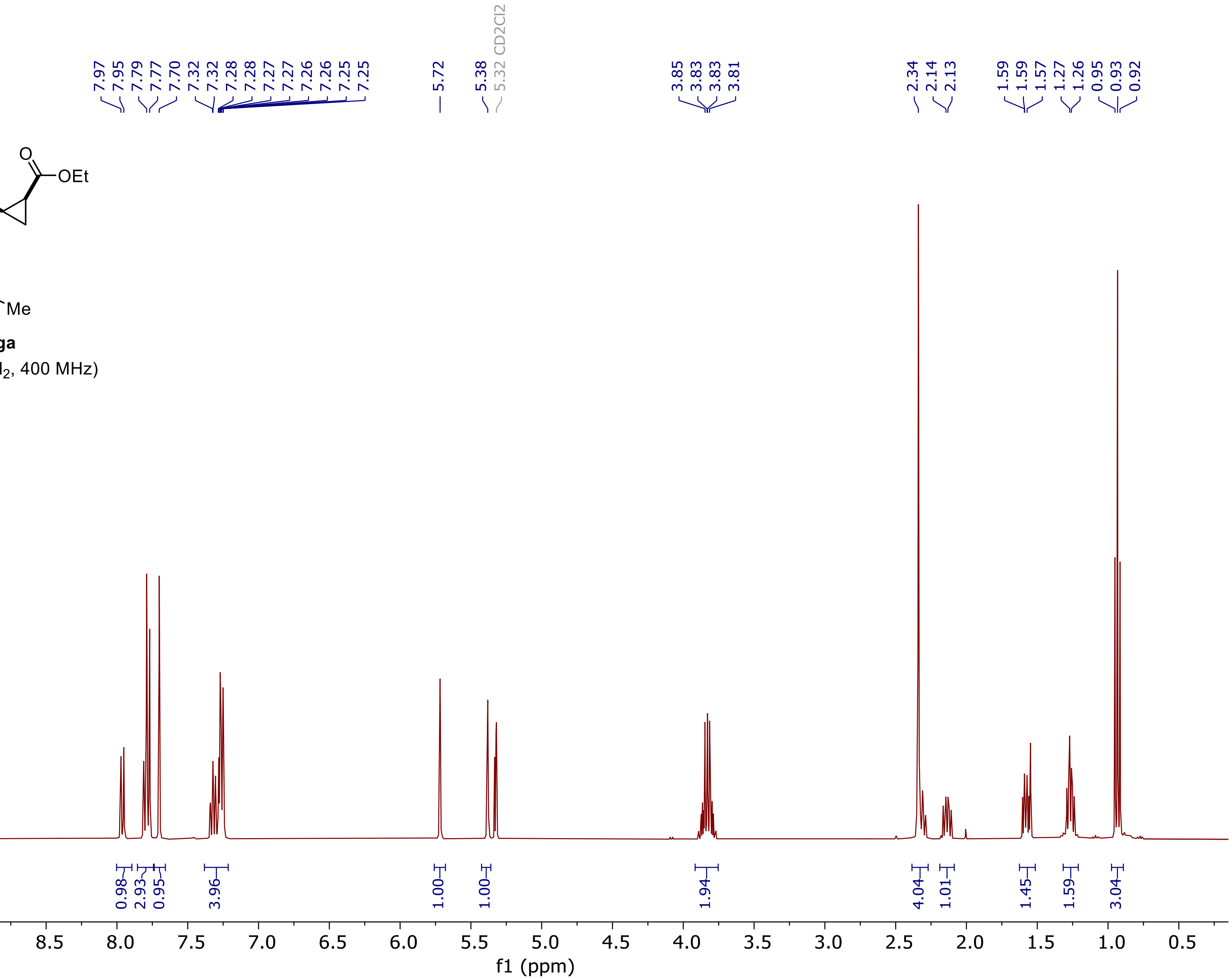

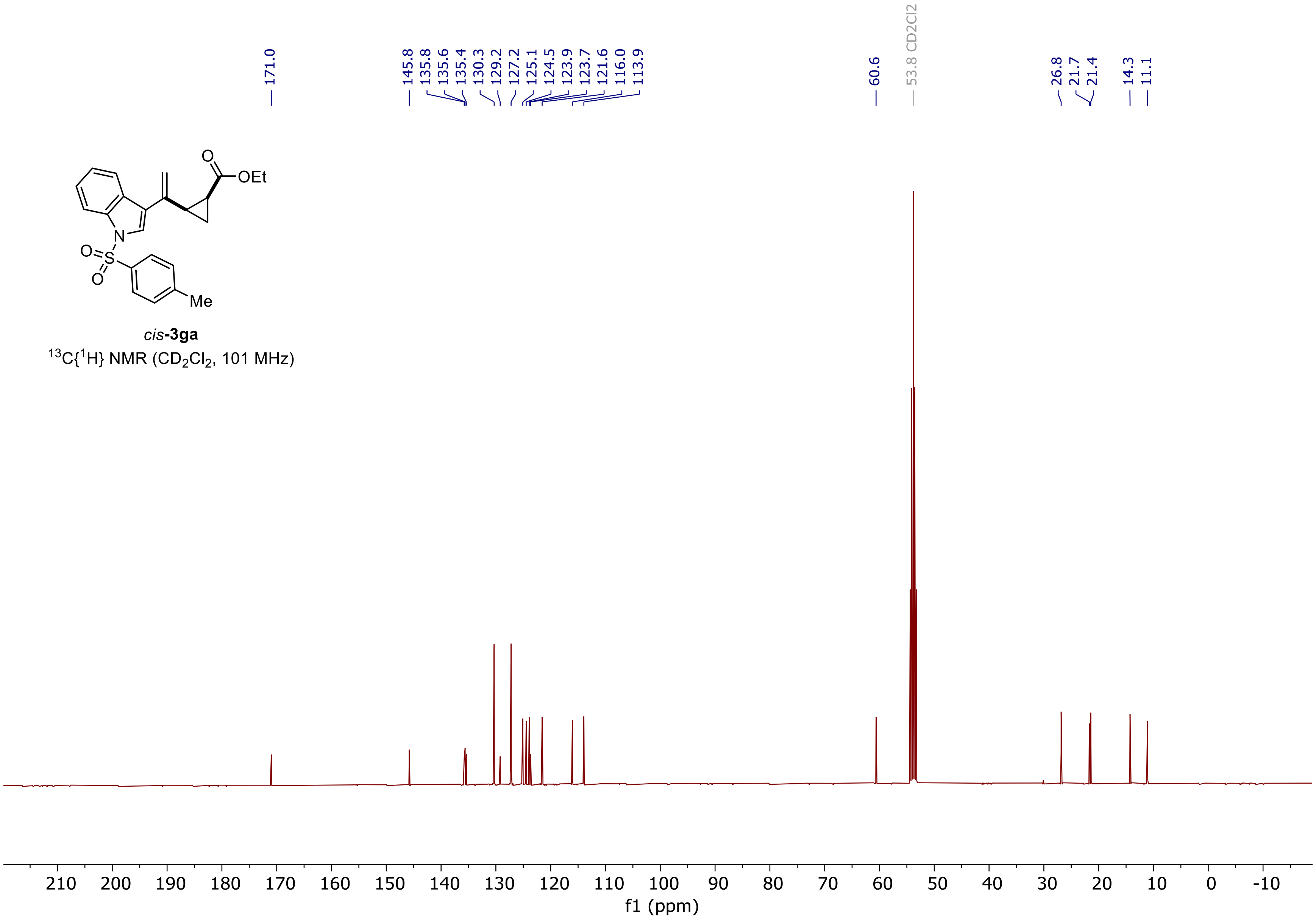
O
OEt
N
O=S
O
Me
cis-3ga
$^{13}C\{^1H\}$ NMR (CD_2Cl_2, 101 MHz)
171.0
145.8
135.8
135.6
135.4
130.3
129.2
127.2
125.1
124.5
123.9
123.7
121.6
116.0
113.9
60.6
53.8 CD2Cl2
26.8
21.7
21.4
14.3
11.1
210
200
190
180
170
160
150
140
130
120
110
100
90
80
70
60
50
40
30
20
10
0
-10
f1 (ppm)

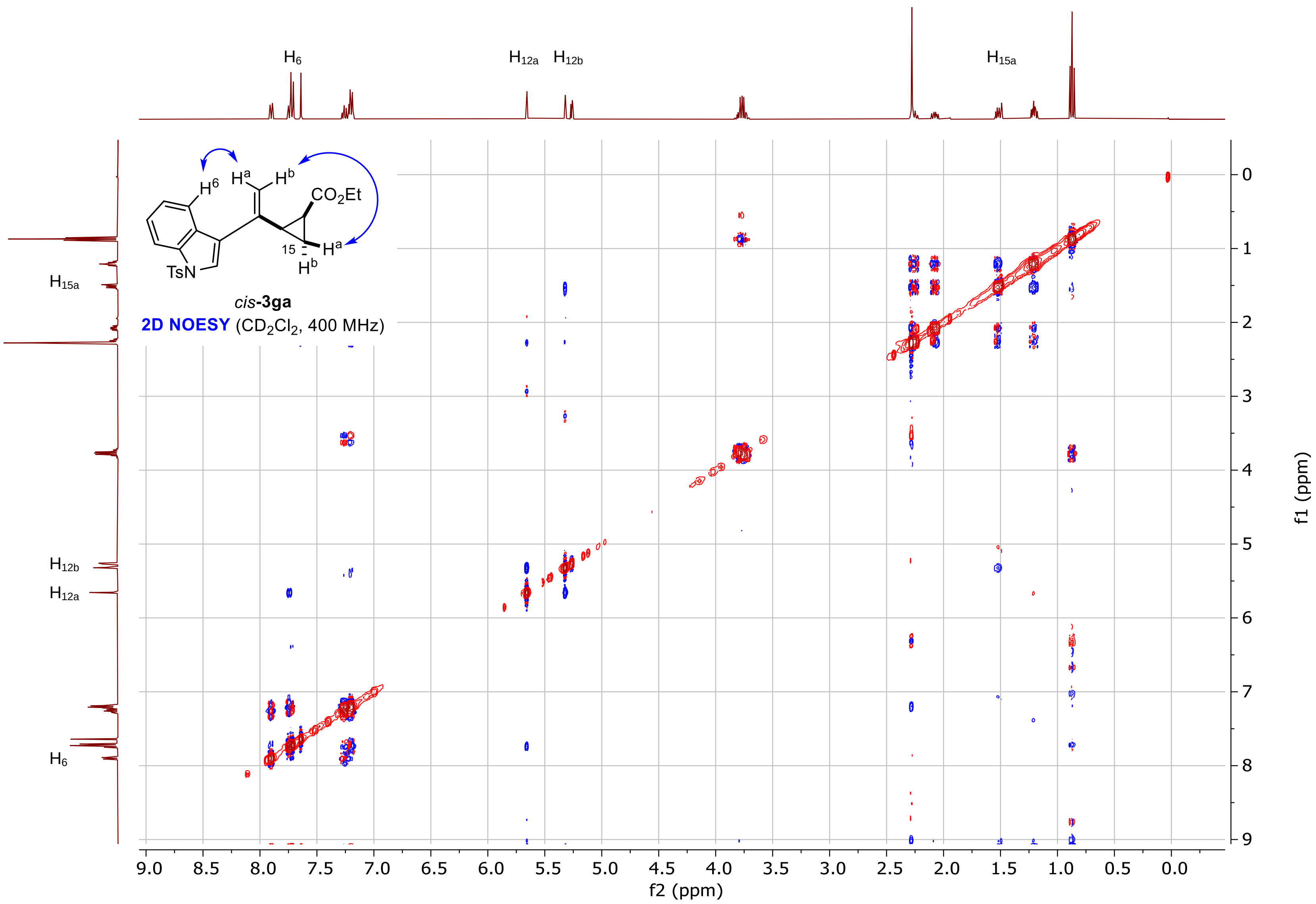
H6
H12a
H12b
H15a
H6
Ha
Hb
CO2Et
15
Ha
Hb
TsN
cis-3ga
2D NOESY (CD2Cl2, 400 MHz)
f2 (ppm)
f1 (ppm)

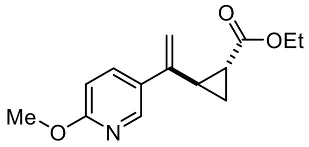

trans-**3ha**

^{1}H NMR ($CDCl_3$, 400 MHz)

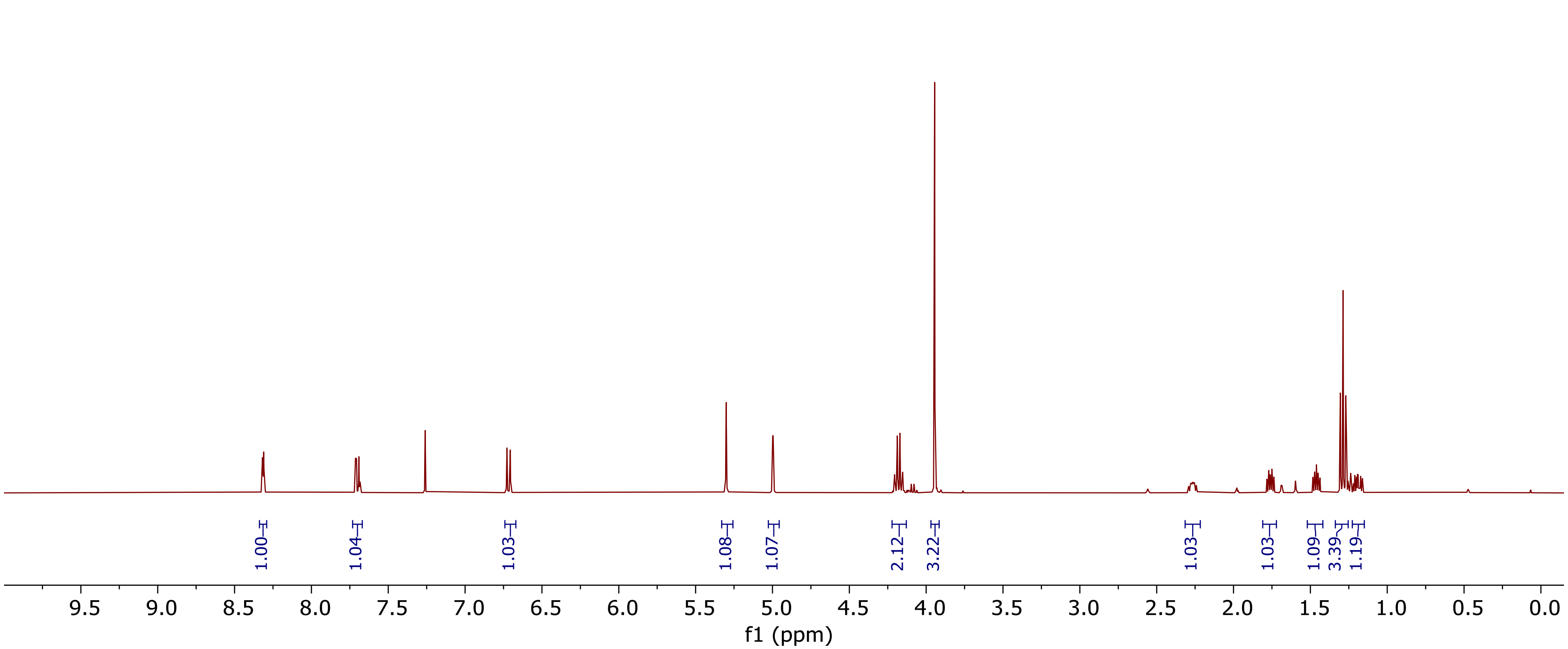

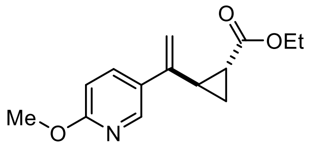

trans-**3ha**
$^{13}C\{^1H\}$ NMR ($CDCl_3$, 101 MHz)

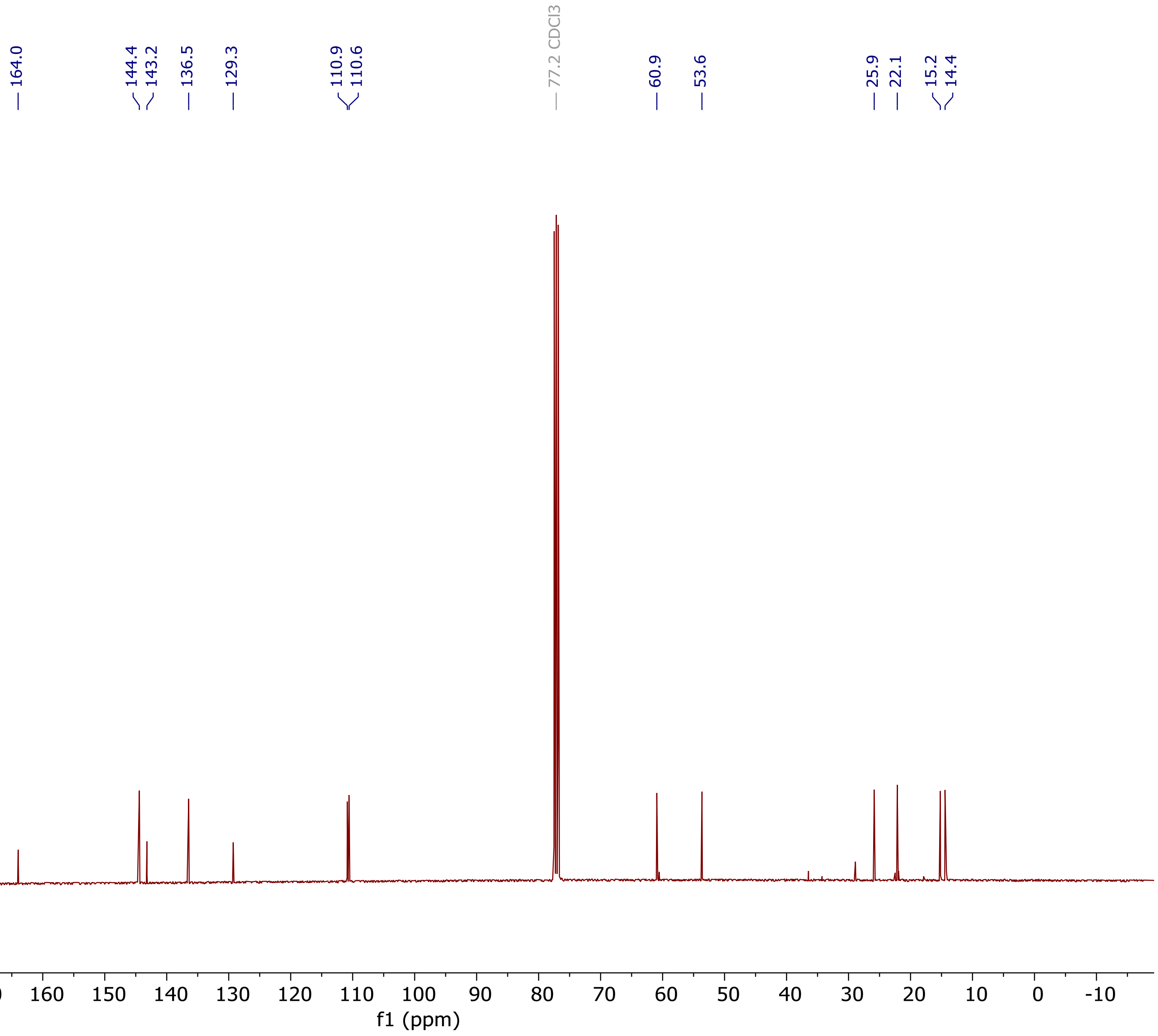

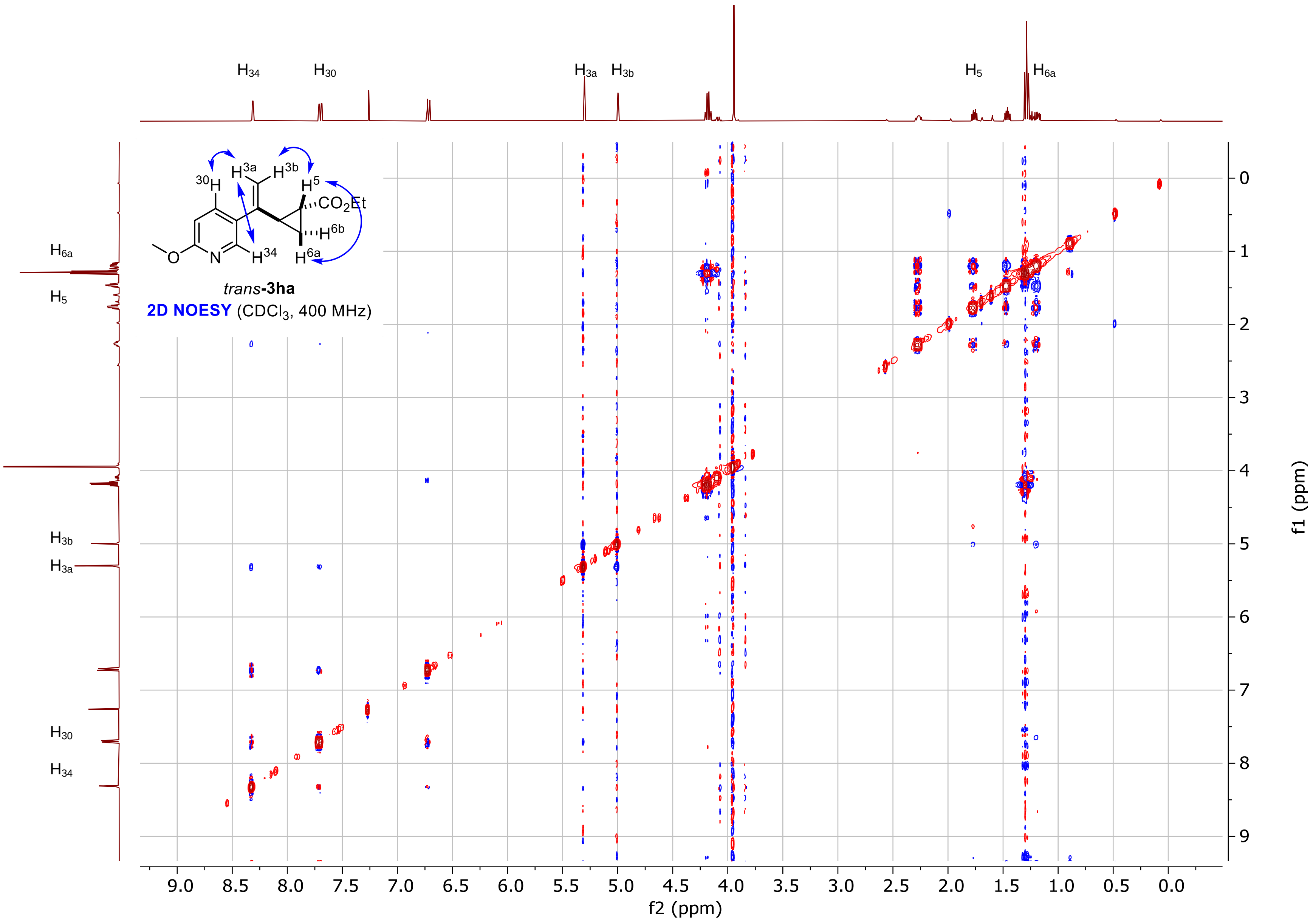
H34
H30
H3a
H3b
H5
H6a
trans-3ha
2D NOESY (CDCl3, 400 MHz)
f2 (ppm)
f1 (ppm)

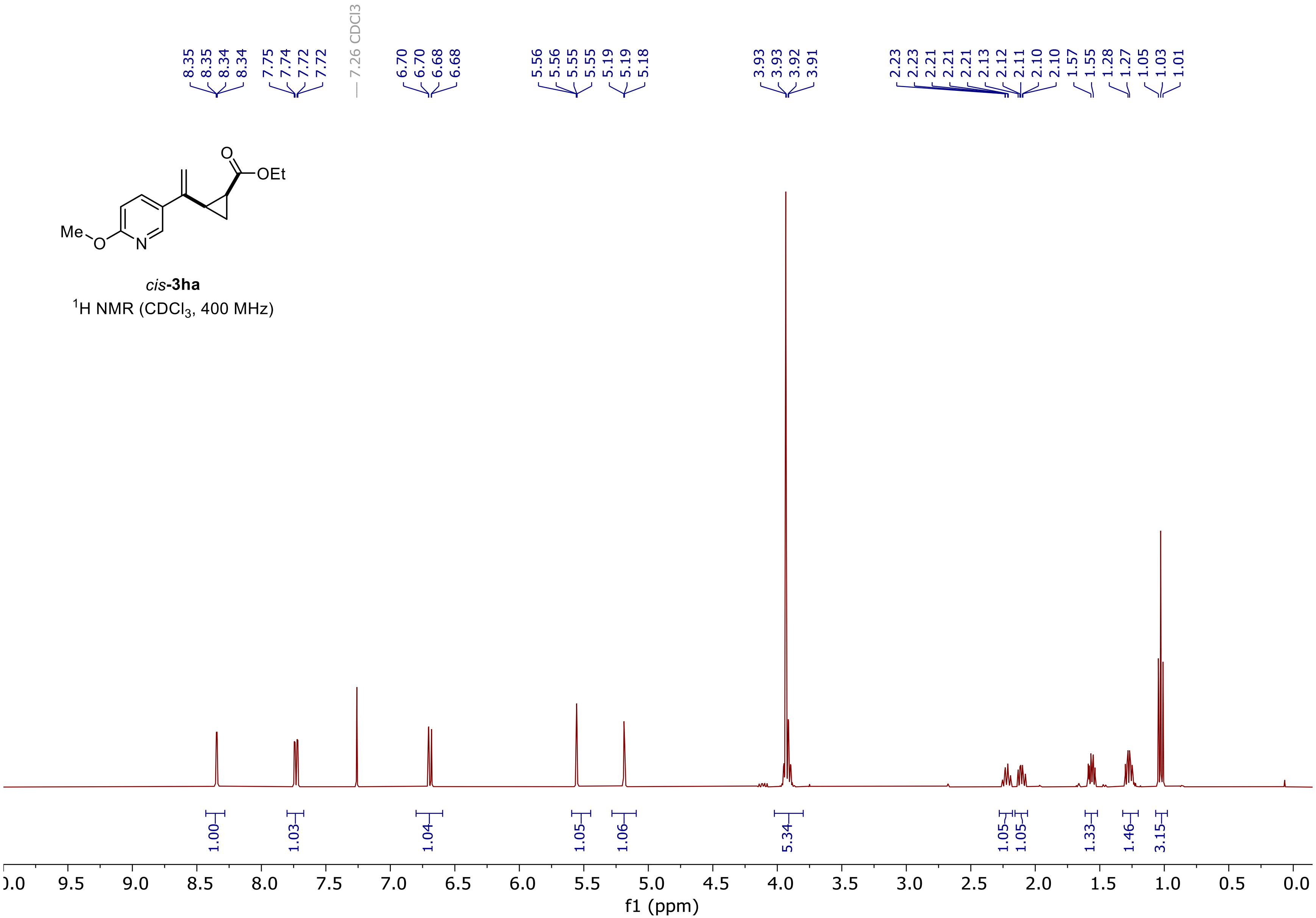

cis-**3ha**

^{1}H NMR ($CDCl_3$, 400 MHz)

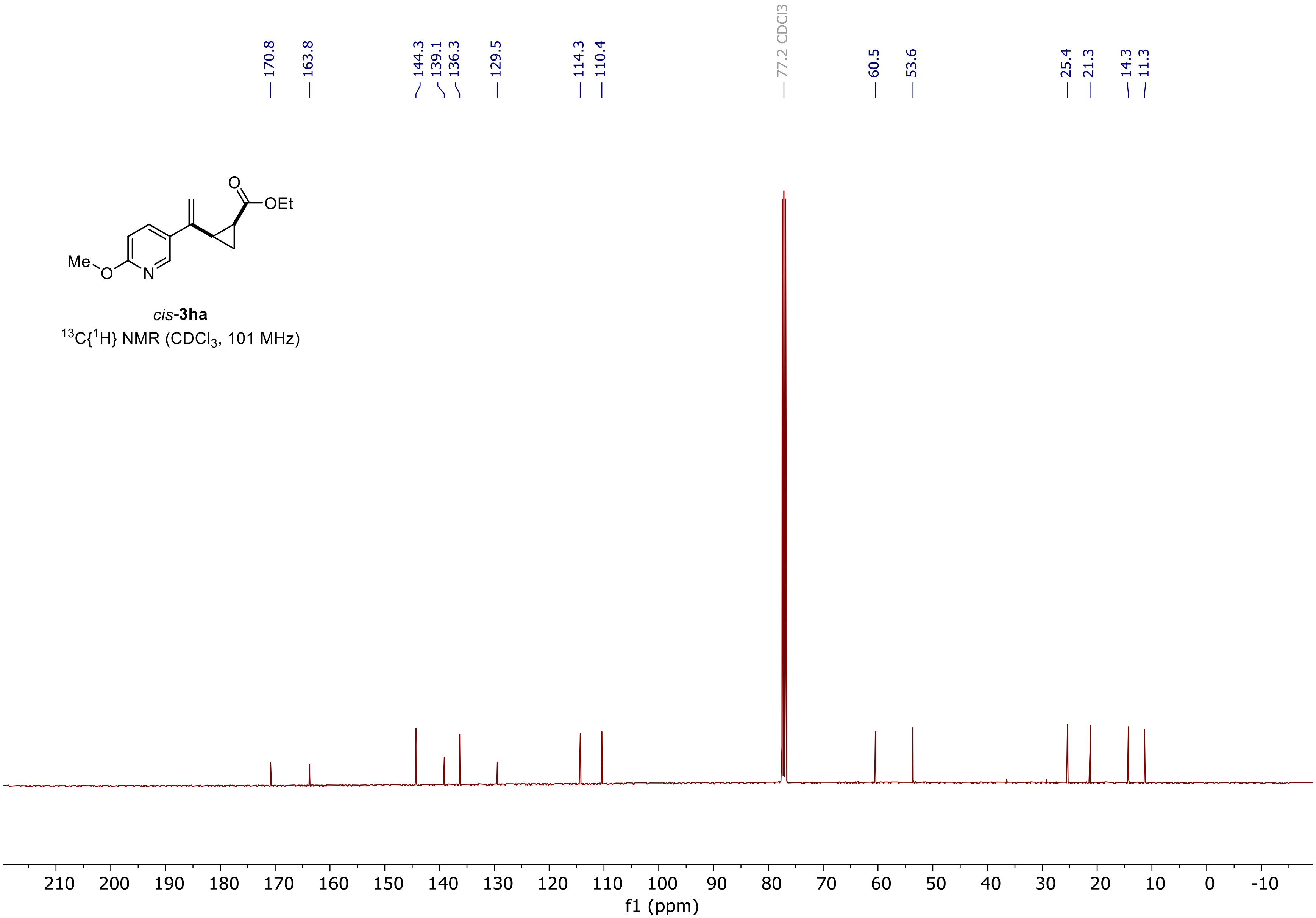

cis-**3ha**

$^{13}C\{^{1}H\}$ NMR ($CDCl_3$, 101 MHz)

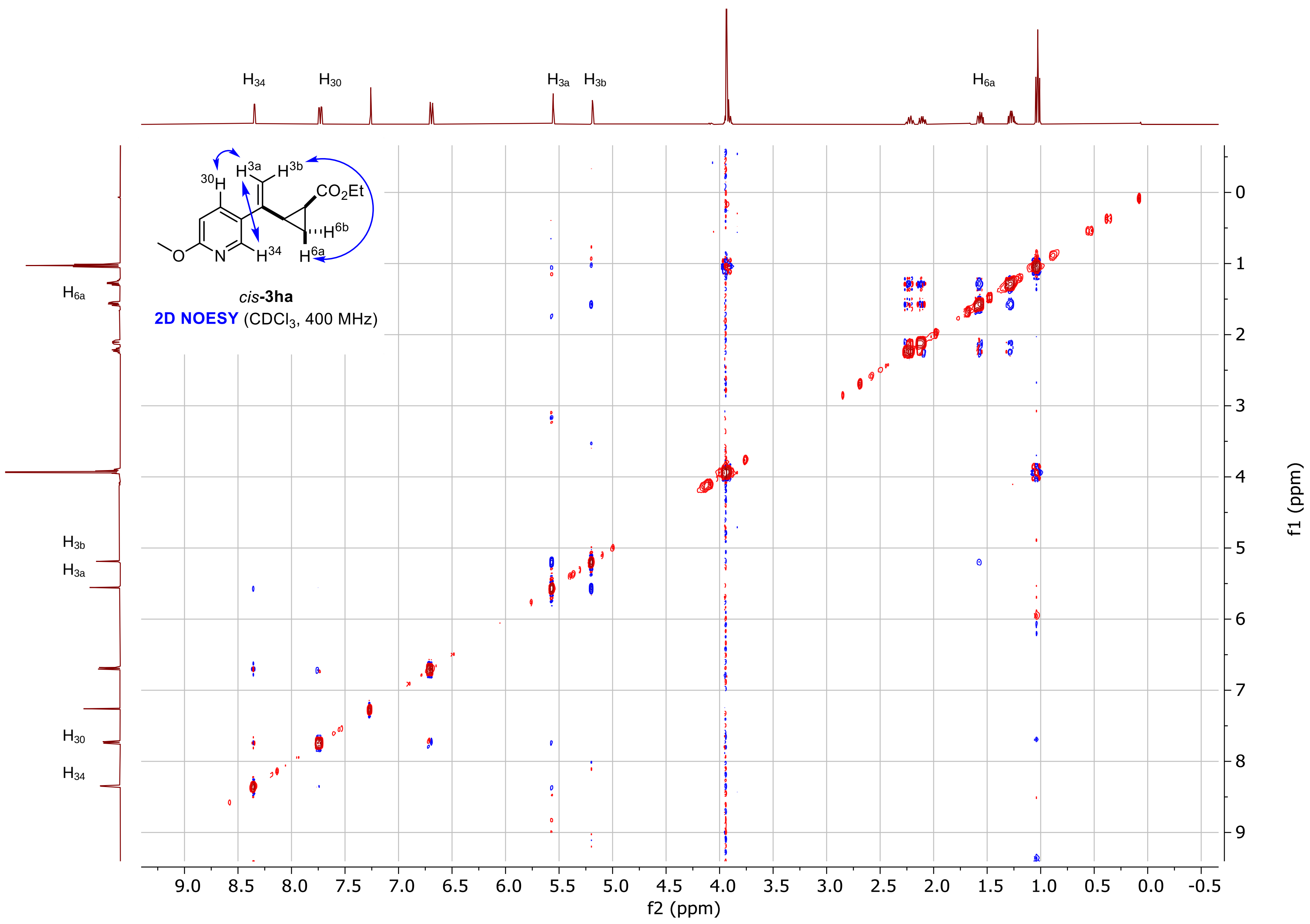
H34
H30
H3a
H3b
H6a
30H
H3a
H3b
CO2Et
H6b
H34
H6a
O
N
cis-3ha
2D NOESY (CDCl3, 400 MHz)
H6a
H3b
H3a
H30
H34
f2 (ppm)
f1 (ppm)

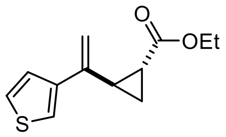

trans-**3ia**

^{1}H NMR ($CDCl_3$, 400 MHz)

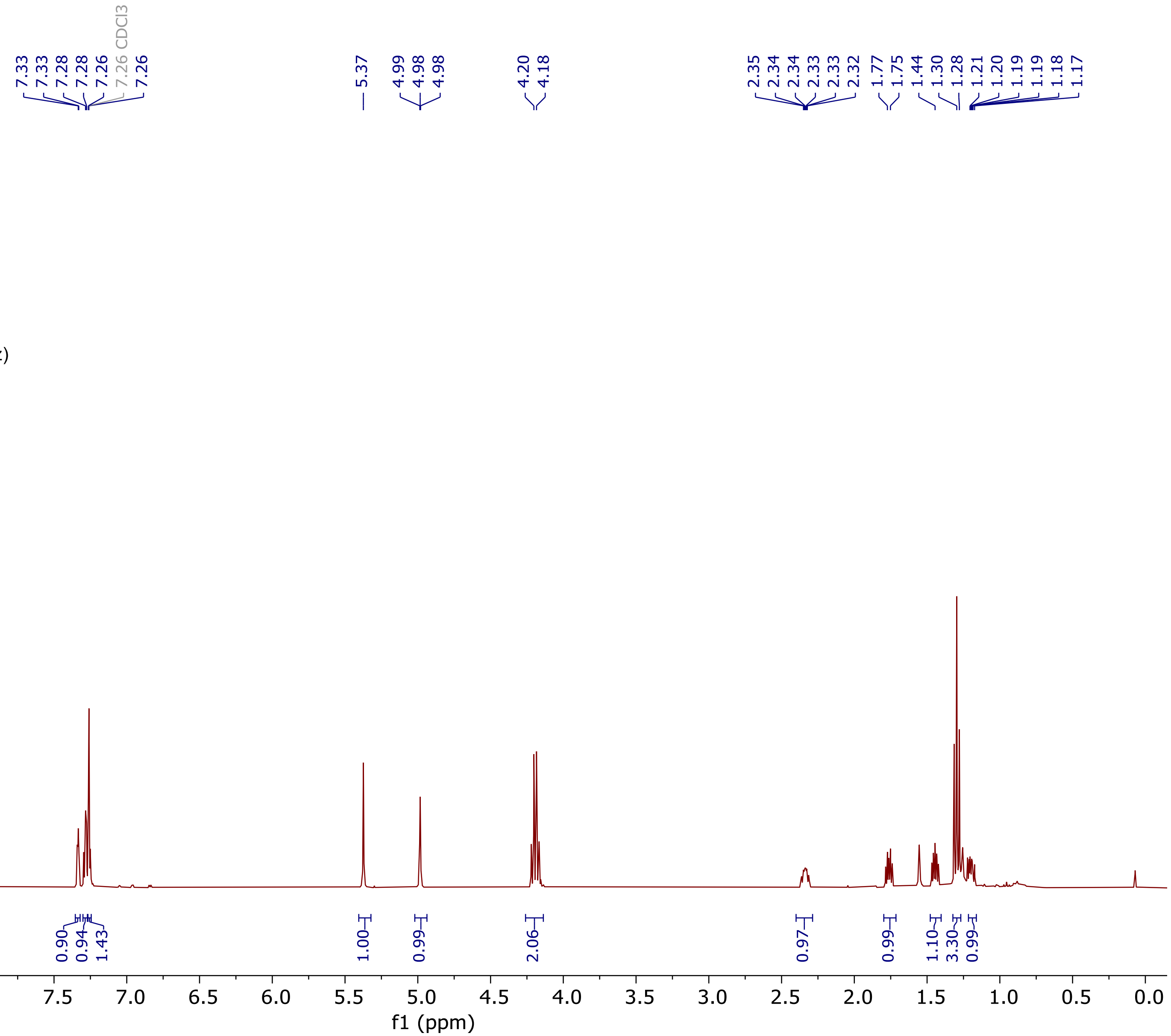

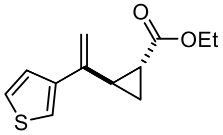

trans-**3ia**

$^{13}C\{^{1}H\}$ NMR ($CDCl_3$, 101 MHz)

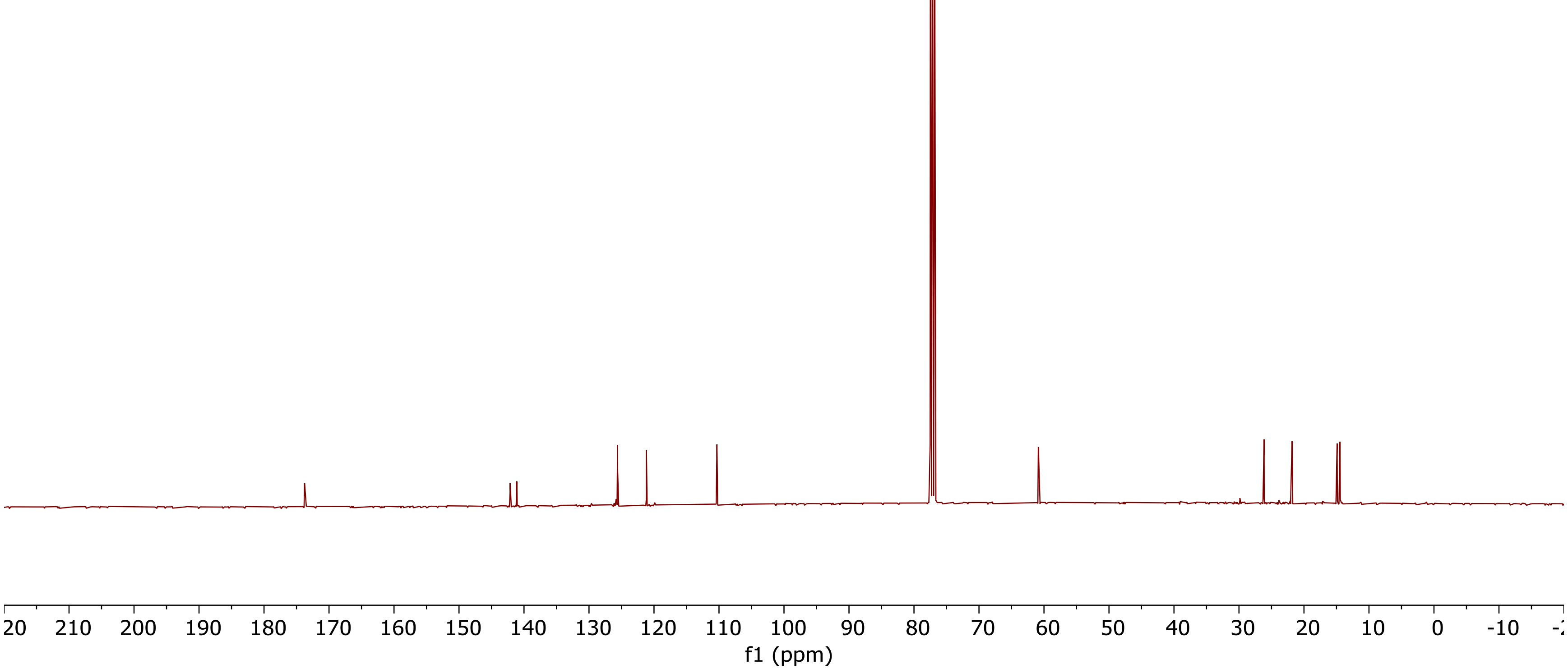

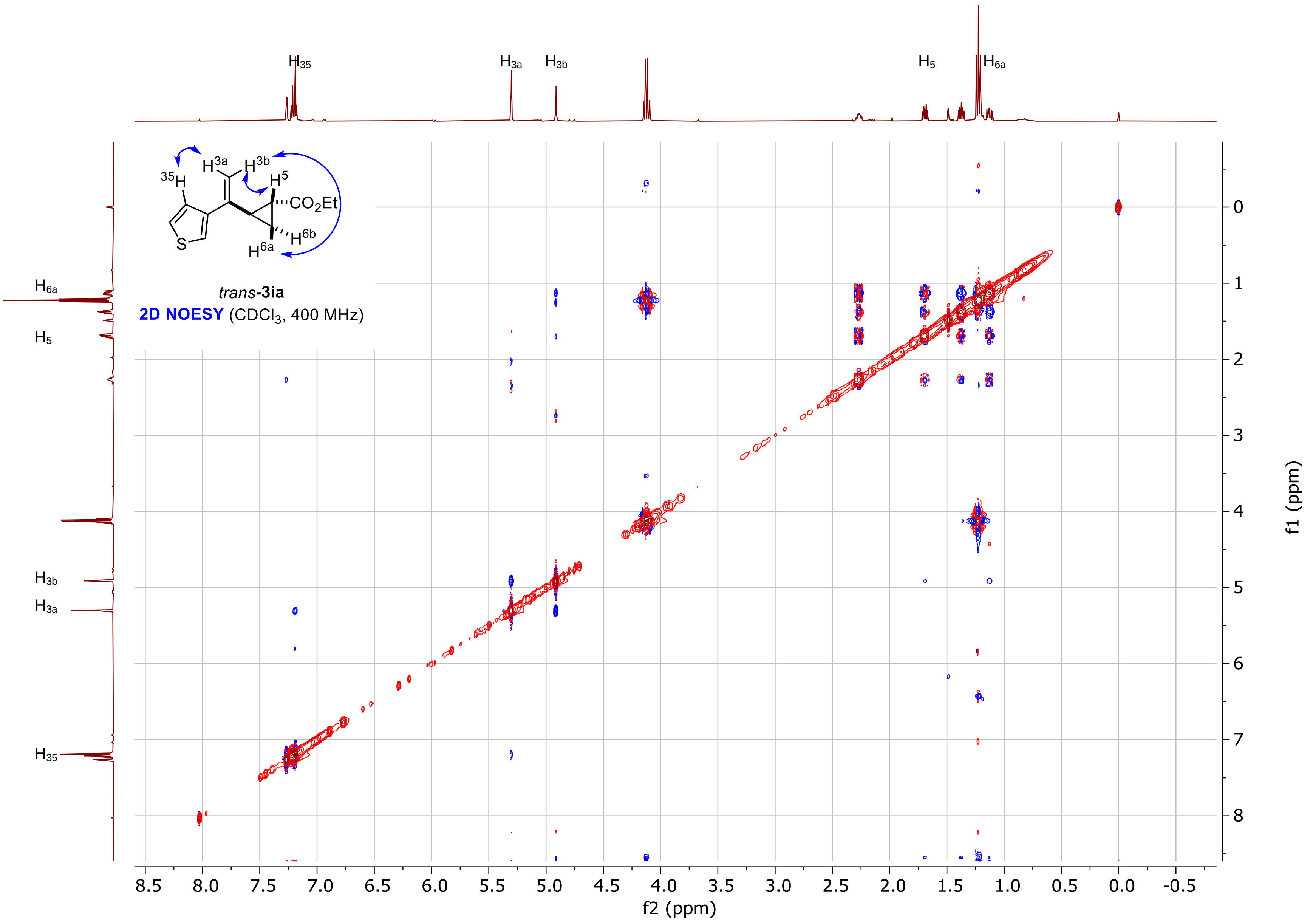
H35
H3a
H3b
H5
H6a
35H
H3a
H3b
H5
CO2Et
S
H6b
H6a
trans-3ia
2D NOESY (CDCl3, 400 MHz)
f2 (ppm)
f1 (ppm)

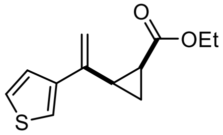

cis-**3ia**

^{1}H NMR ($CDCl_3$, 400 MHz)

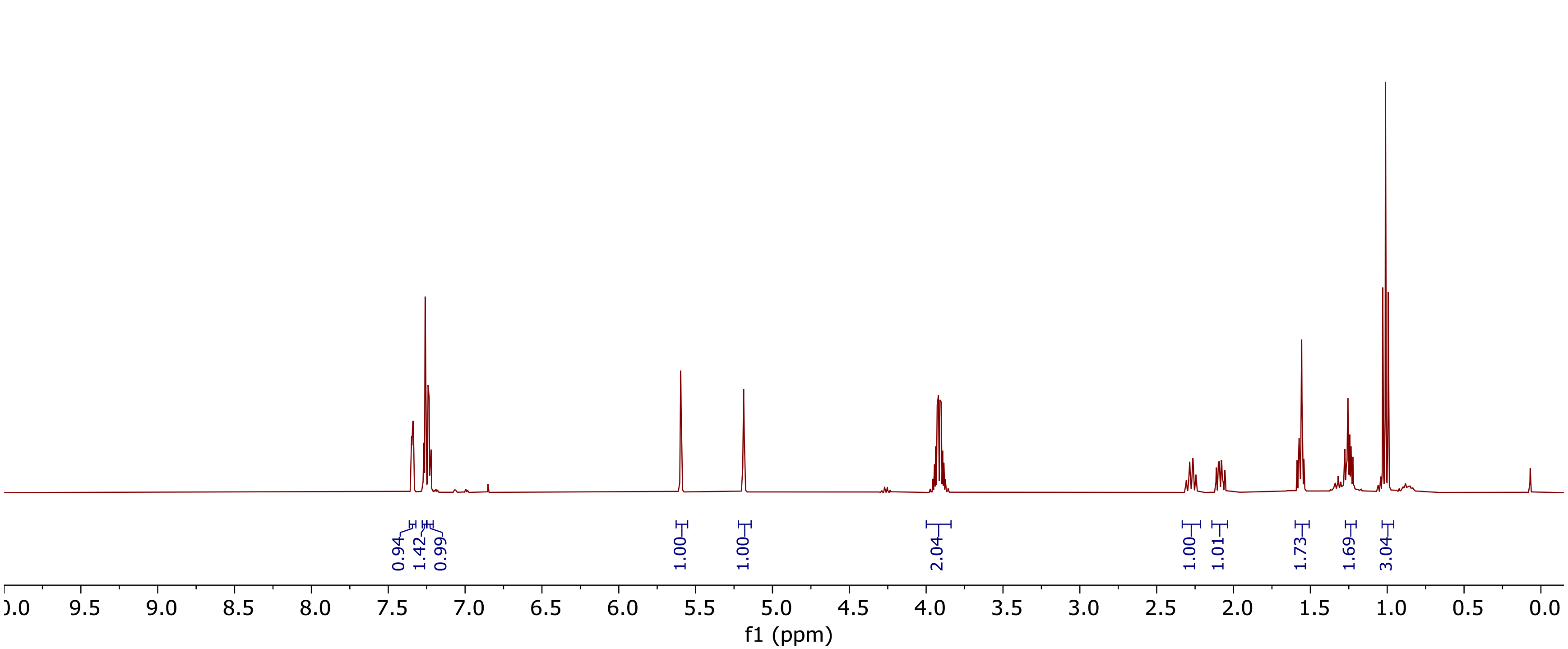

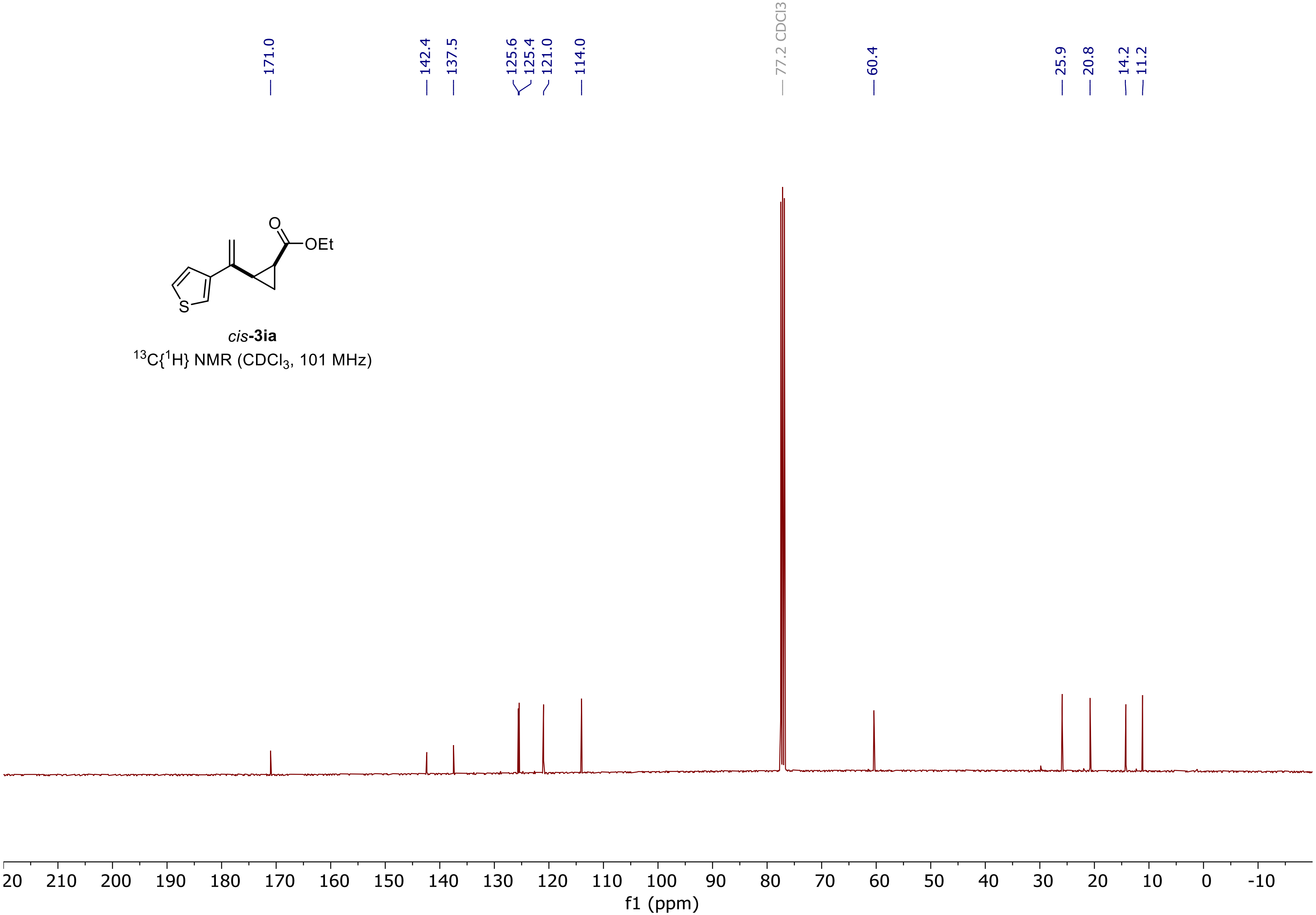

cis-**3ia**

$^{13}C\{^{1}H\}$ NMR ($CDCl_3$, 101 MHz)

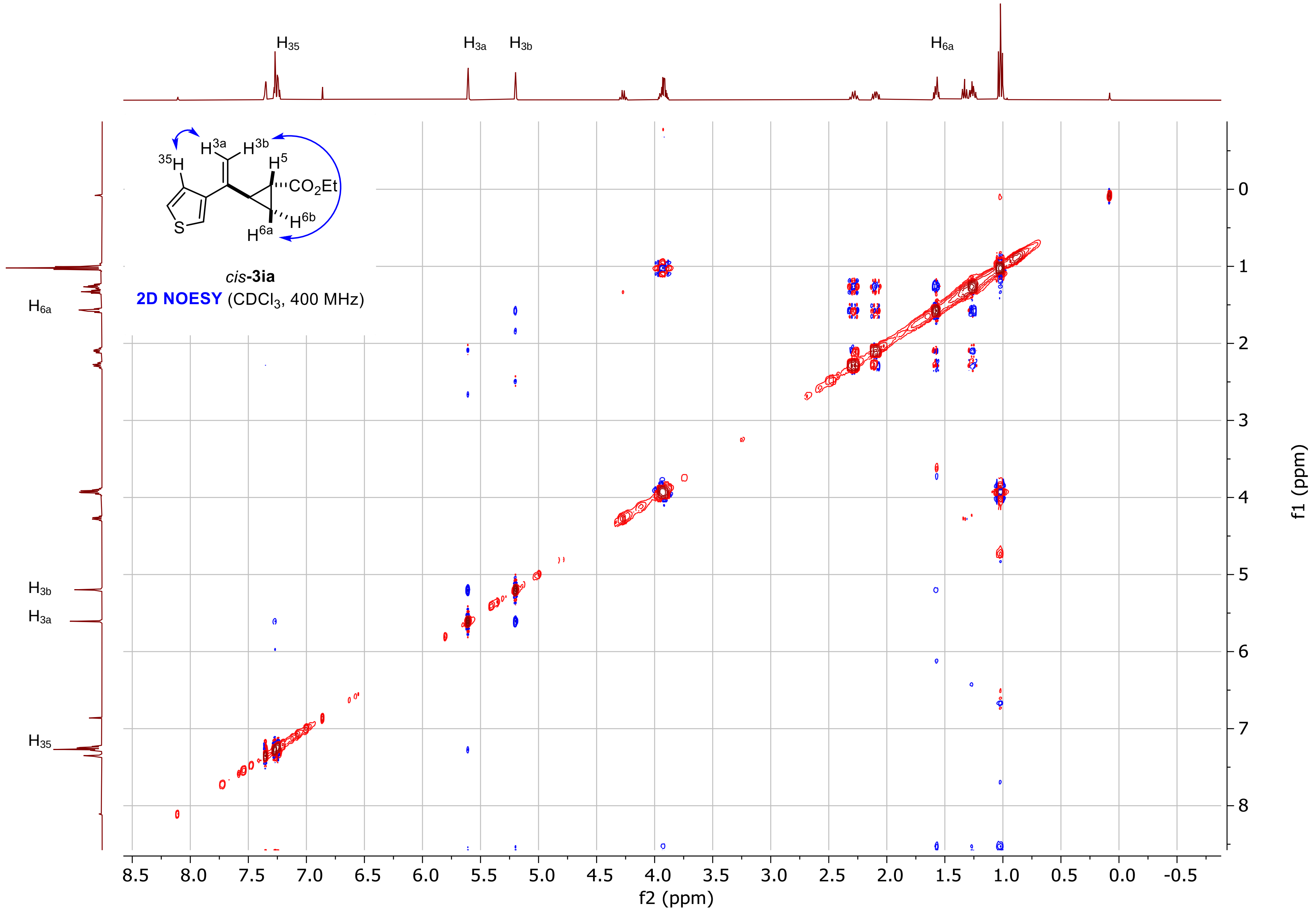
H35
H3a
H3b
H6a
35H
H3a
H3b
H5
CO2Et
H6b
H6a
S
cis-3ia
2D NOESY (CDCl3, 400 MHz)
f2 (ppm)
f1 (ppm)

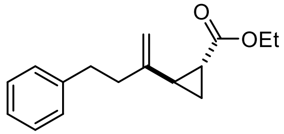

trans-**3ja**
^{1}H NMR (CD_2Cl_2, 400 MHz)

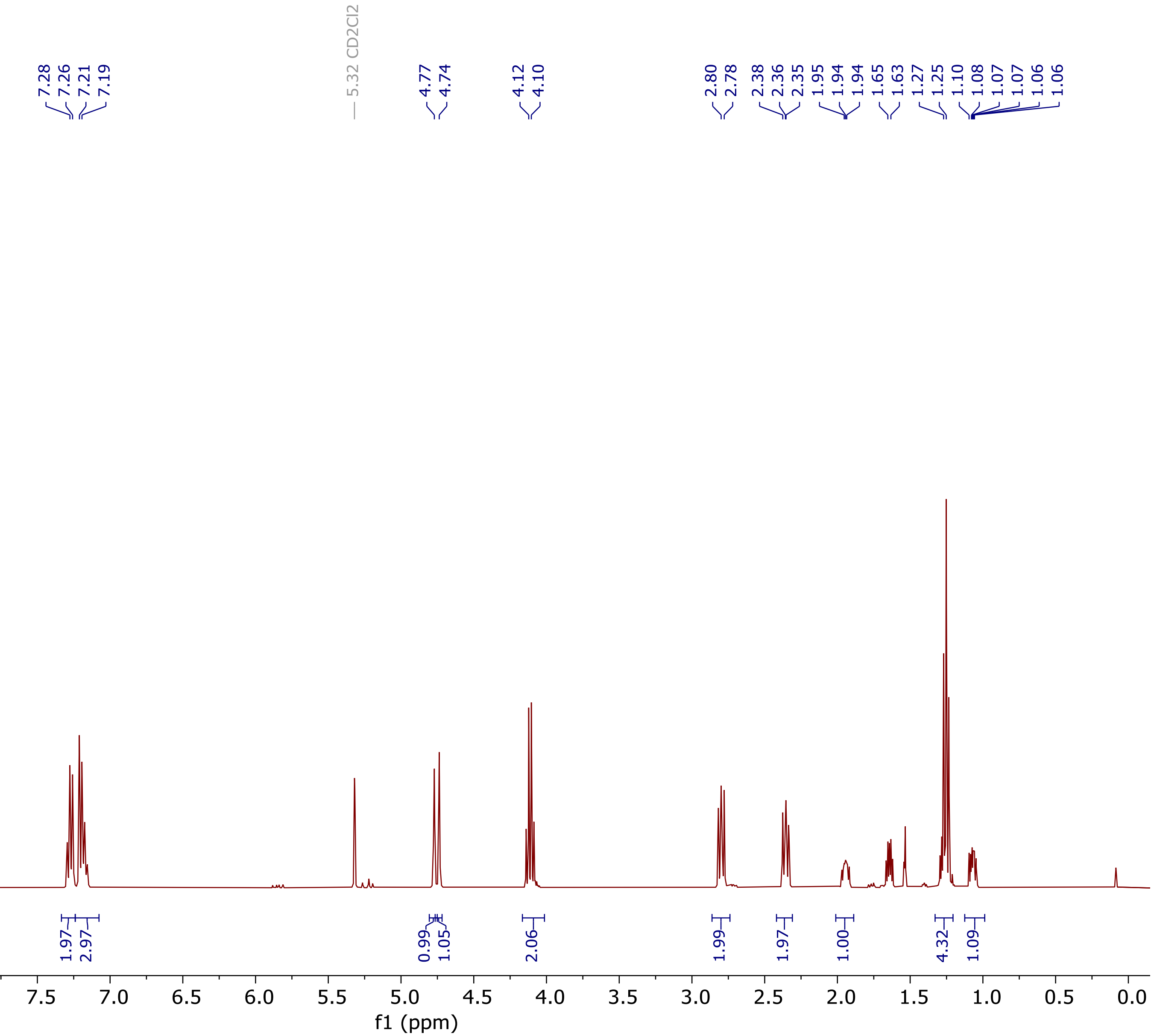

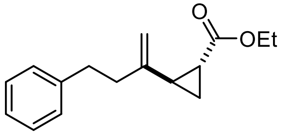

trans-**3ja**

$^{13}C\{^{1}H\}$ NMR (CD_2Cl_2, 101 MHz)

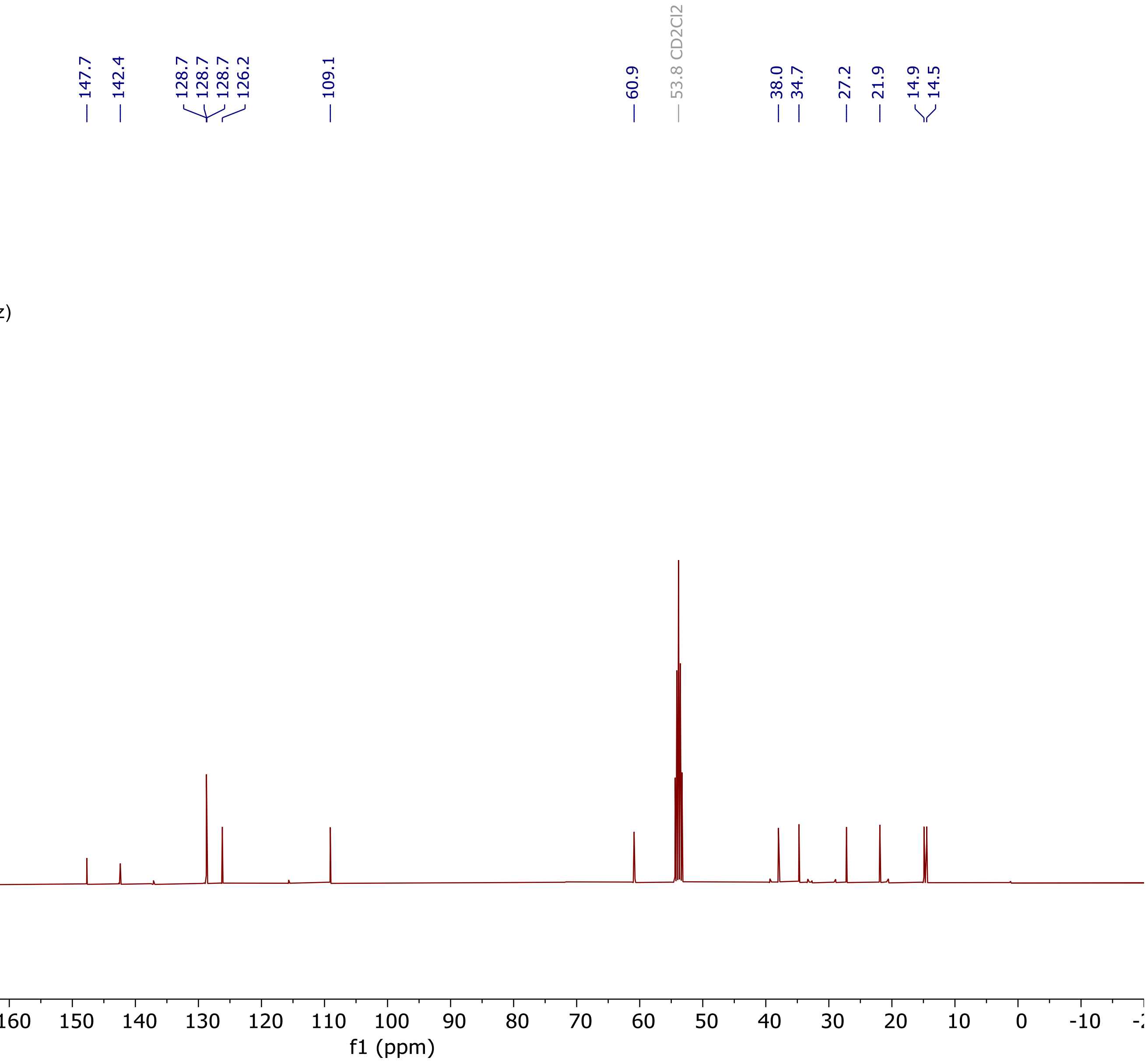

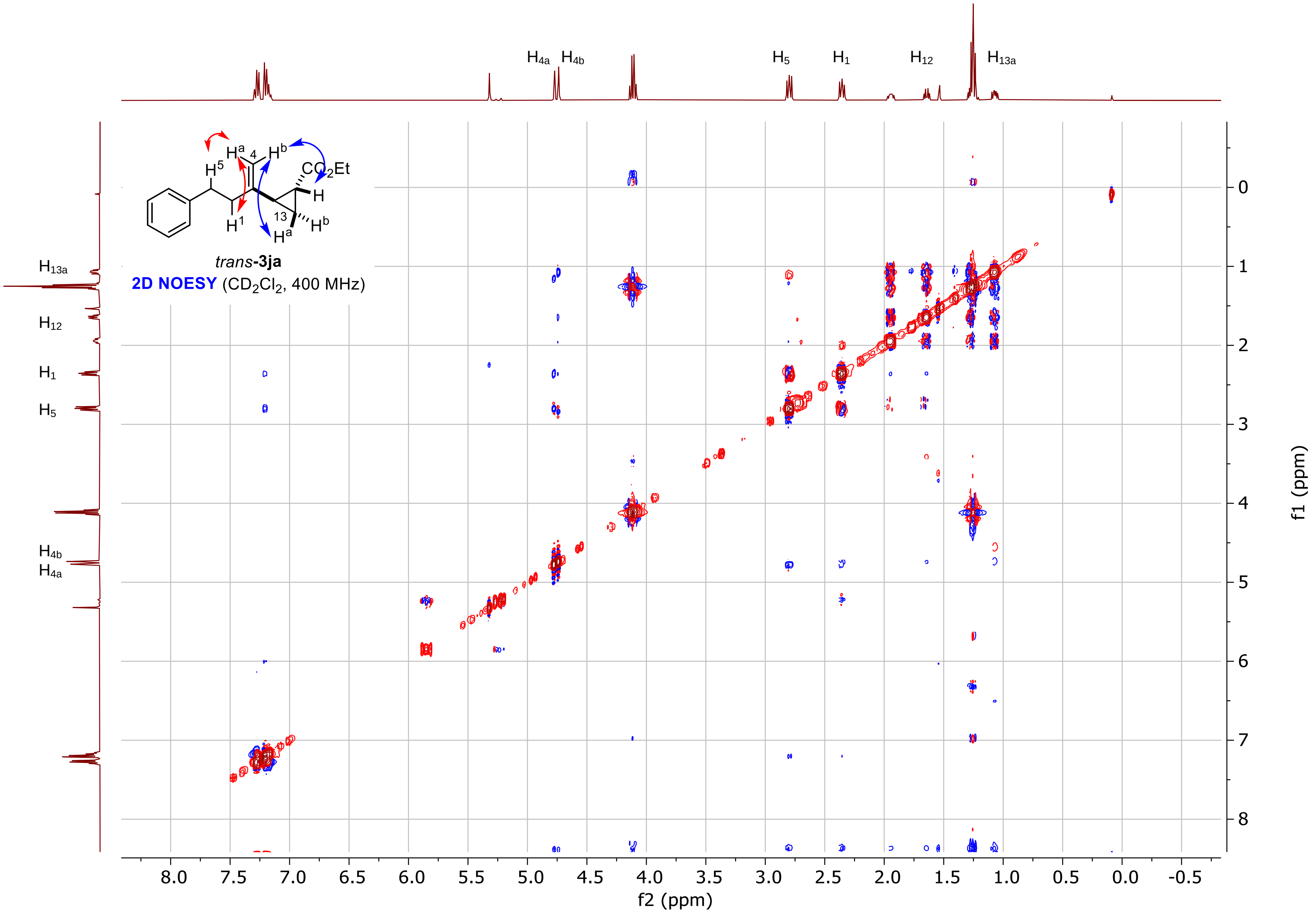
H4a H4b H5 H1 H12 H13a
H13a
H12
H1
H5
H4b
H4a
Ha
4
Hb
H5
CO2Et
H
H1
13
Ha
Hb
trans-3ja
2D NOESY (CD2Cl2, 400 MHz)
8.0 7.5 7.0 6.5 6.0 5.5 5.0 4.5 4.0 3.5 3.0 2.5 2.0 1.5 1.0 0.5 0.0 -0.5
f2 (ppm)
0 1 2 3 4 5 6 7 8
f1 (ppm)

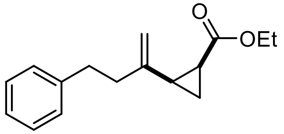

cis-**3ja**

^{1}H NMR (CD_2Cl_2, 400 MHz)

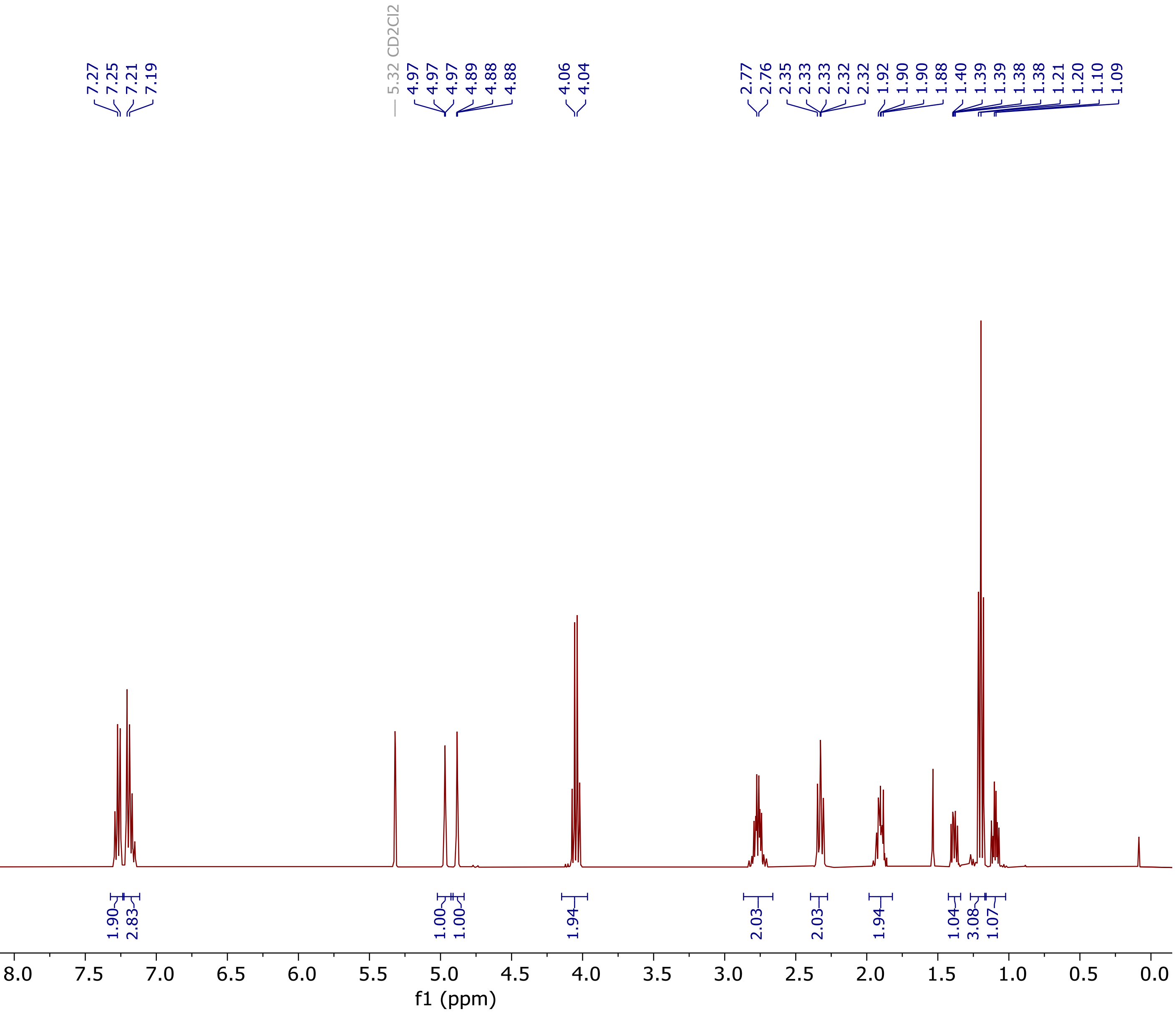

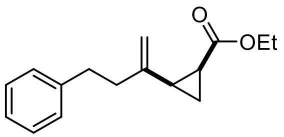

cis-**3ja**

$^{13}C\{^{1}H\}$ NMR (CD_2Cl_2, 101 MHz)

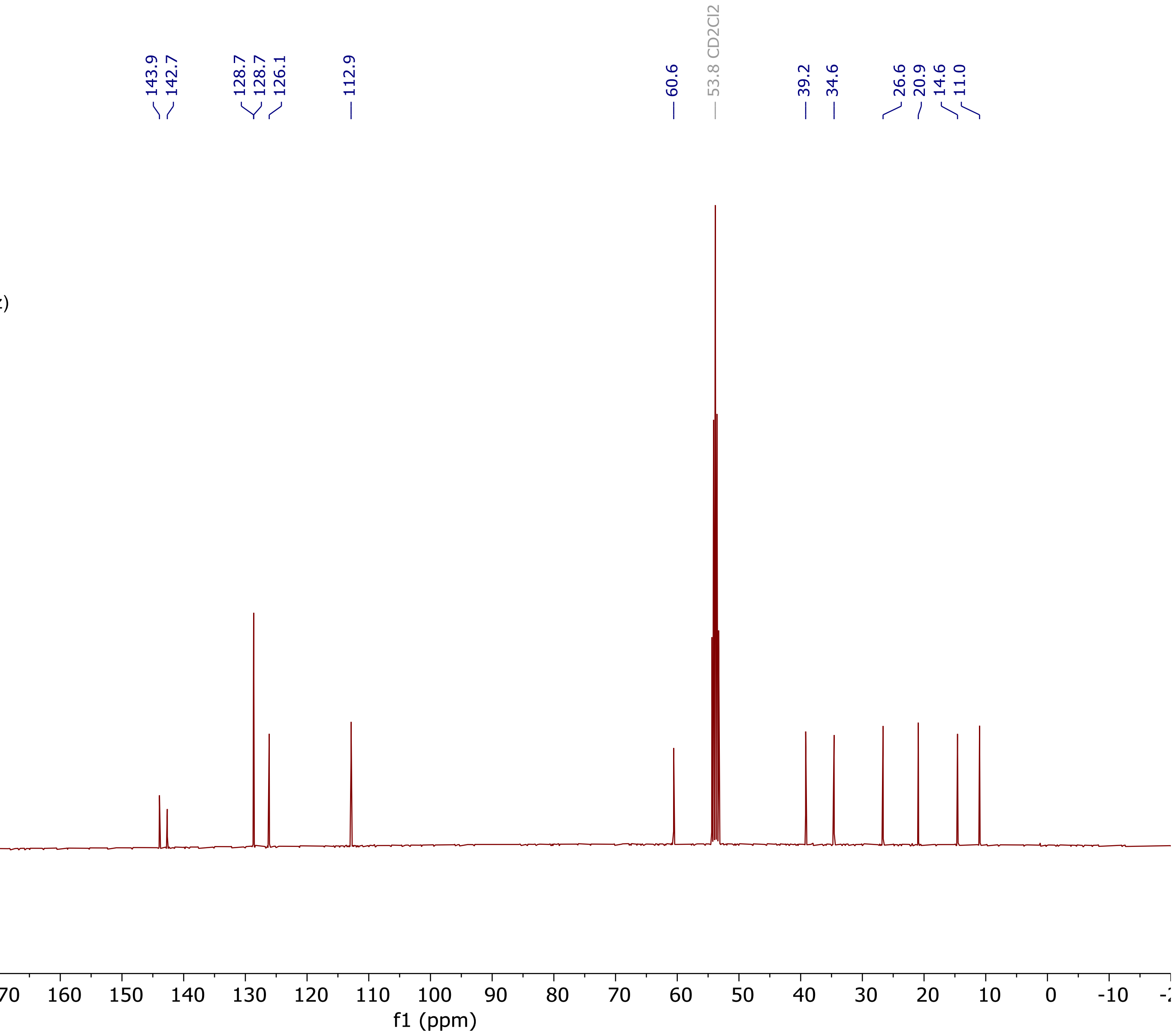

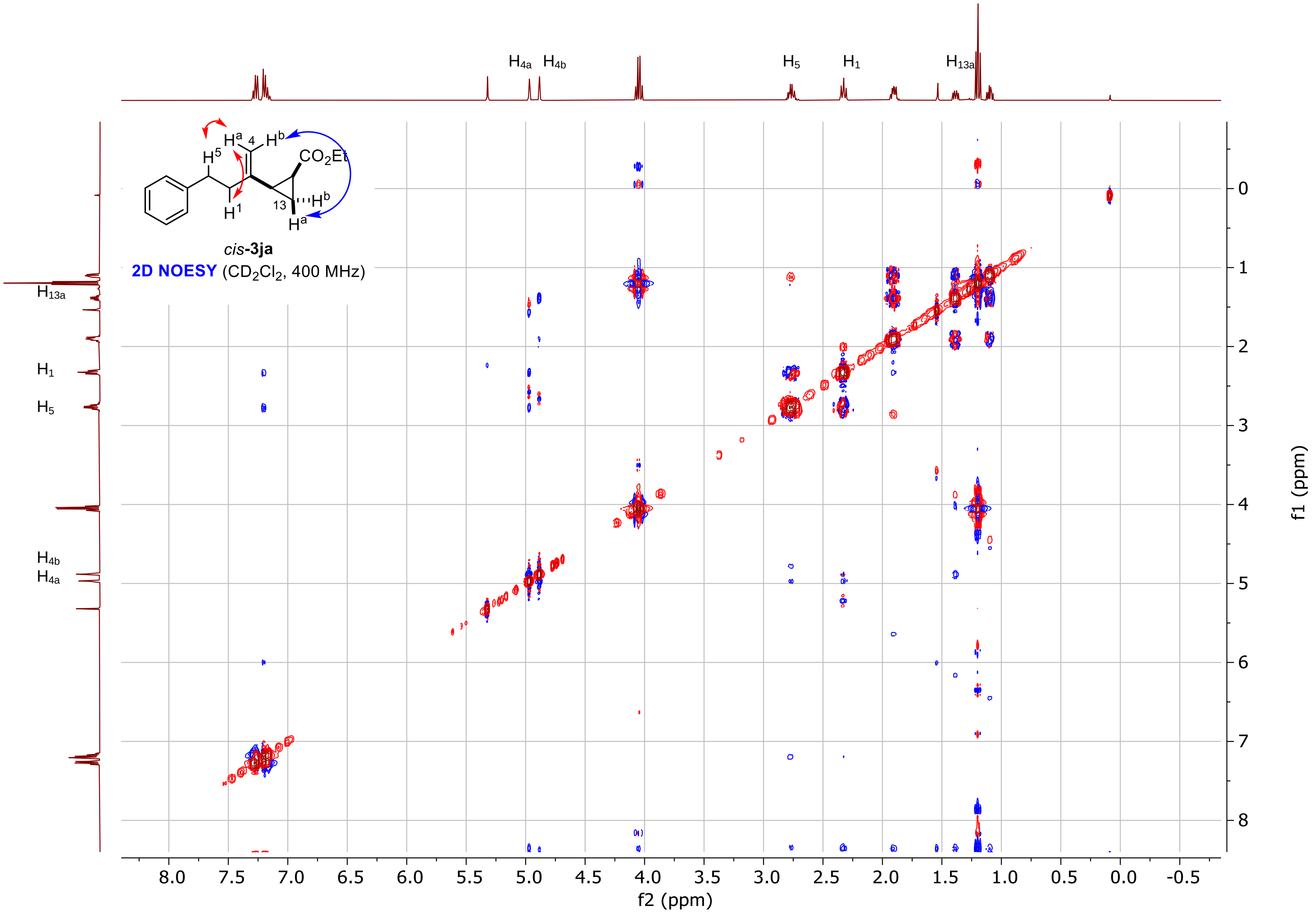

H4a H4b
H5 H1 H13a
Ha 4 Hb
H5
CO2Et
13
Hb
H1
Ha
cis-3ja
2D NOESY (CD2Cl2, 400 MHz)
H13a
H1
H5
H4b
H4a
f2 (ppm)
f1 (ppm)

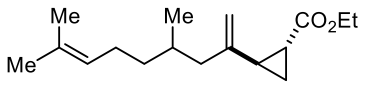

trans-**3ka**

^{1}H NMR ($CDCl_3$, 400 MHz)

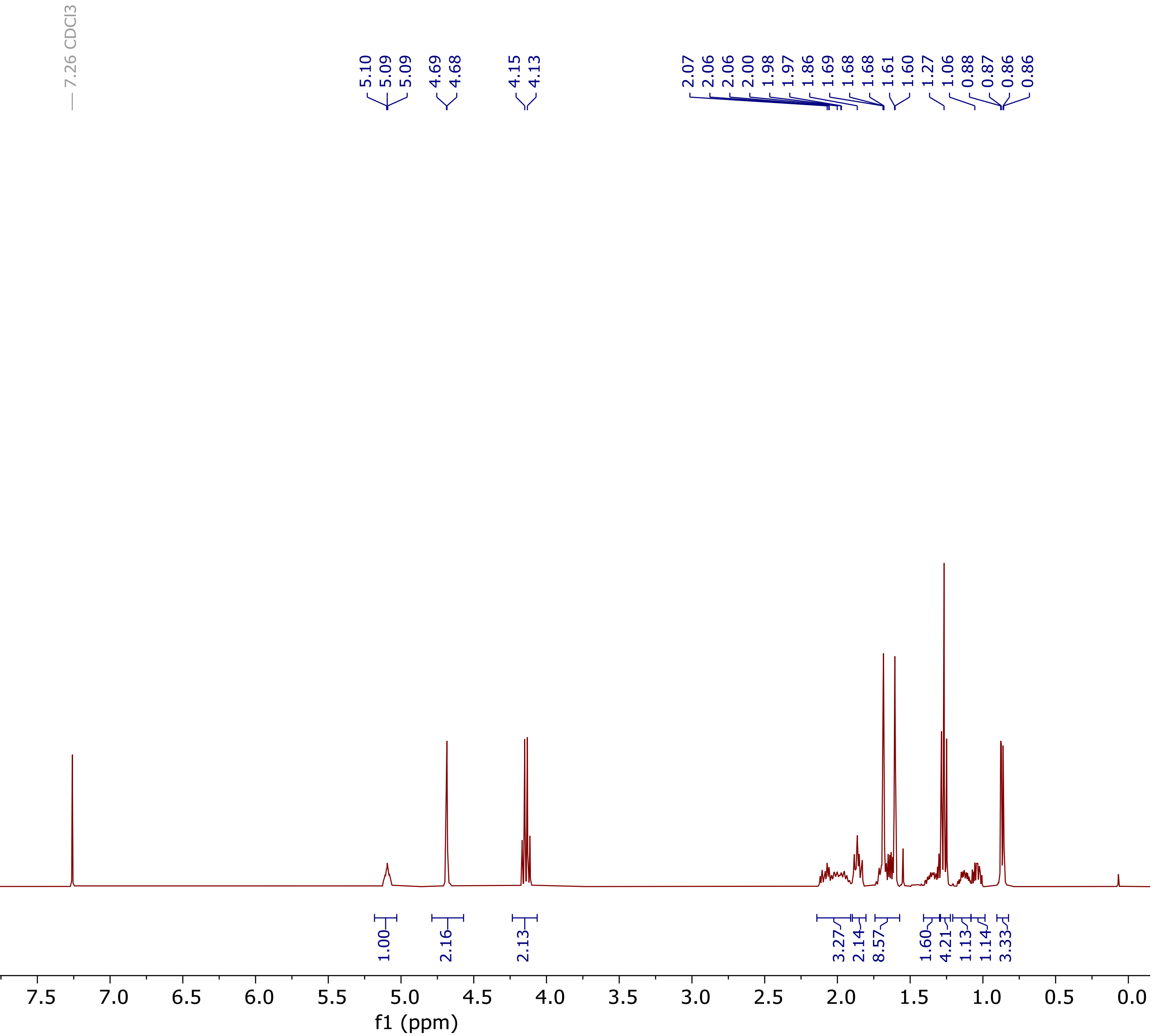

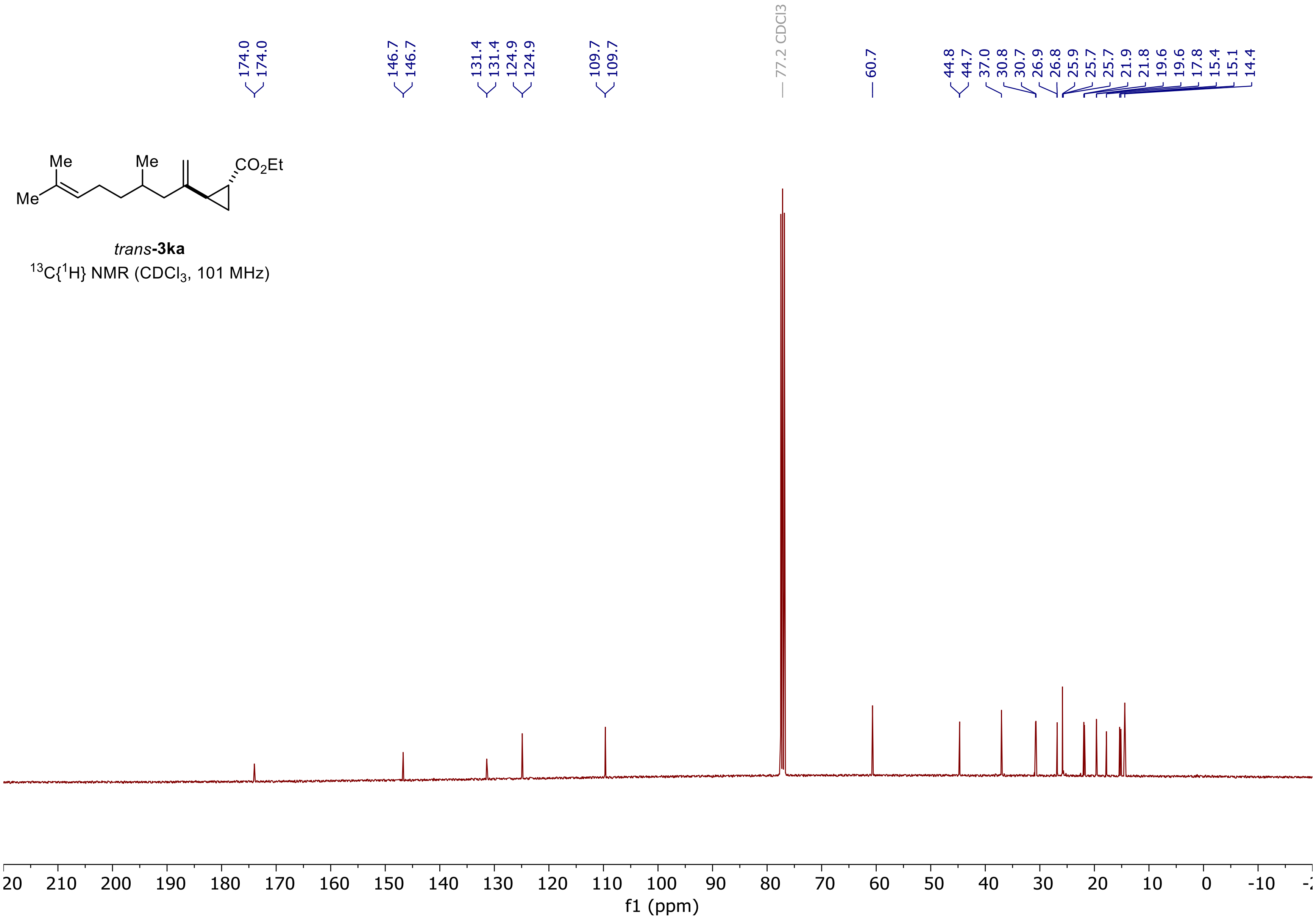

trans-**3ka**

$^{13}C\{^1H\}$ NMR ($CDCl_3$, 101 MHz)

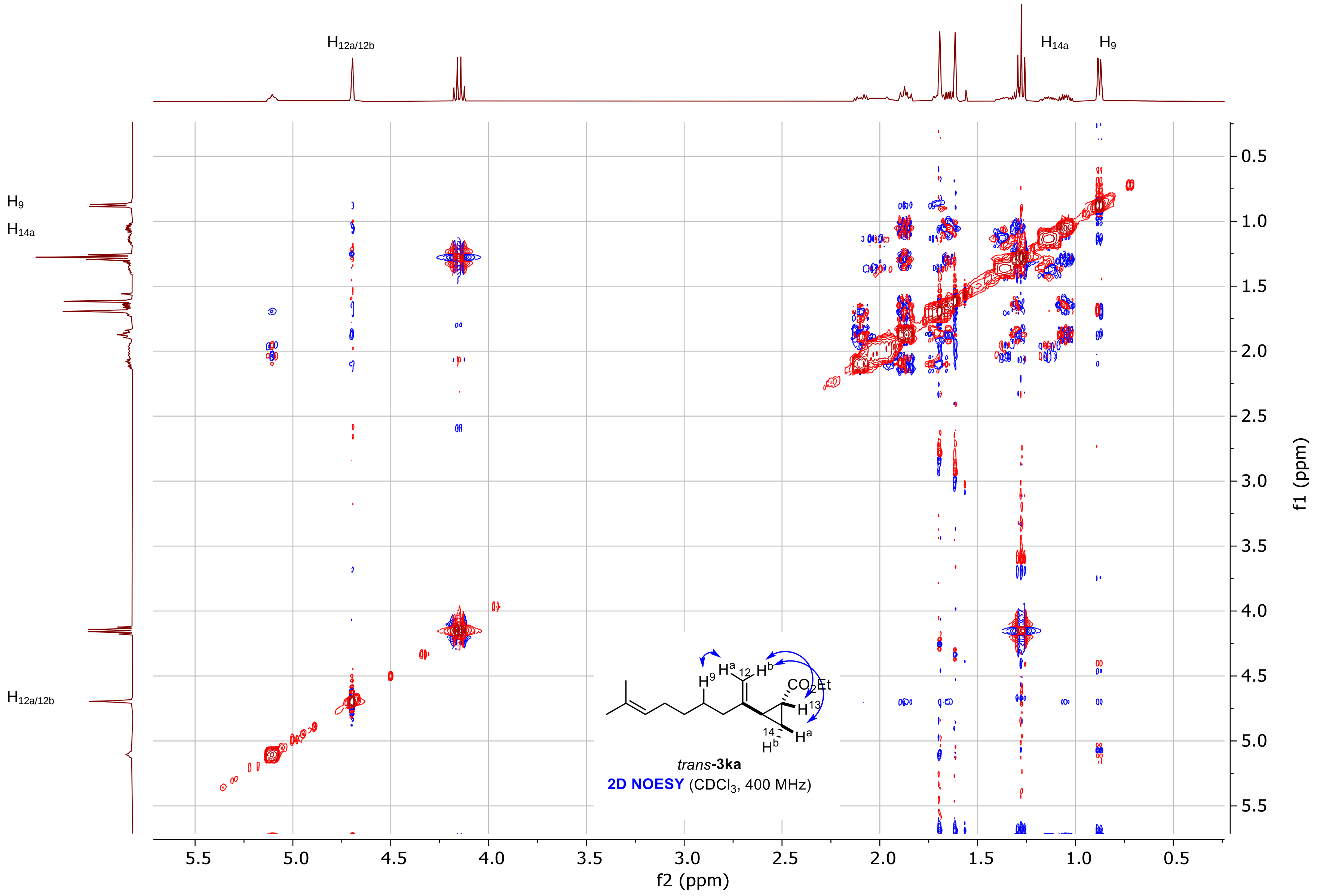

trans-**3ka**

2D NOESY ($CDCl_3$, 400 MHz)

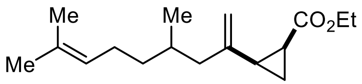

cis-**3ka**

^{1}H NMR ($CDCl_3$, 400 MHz)

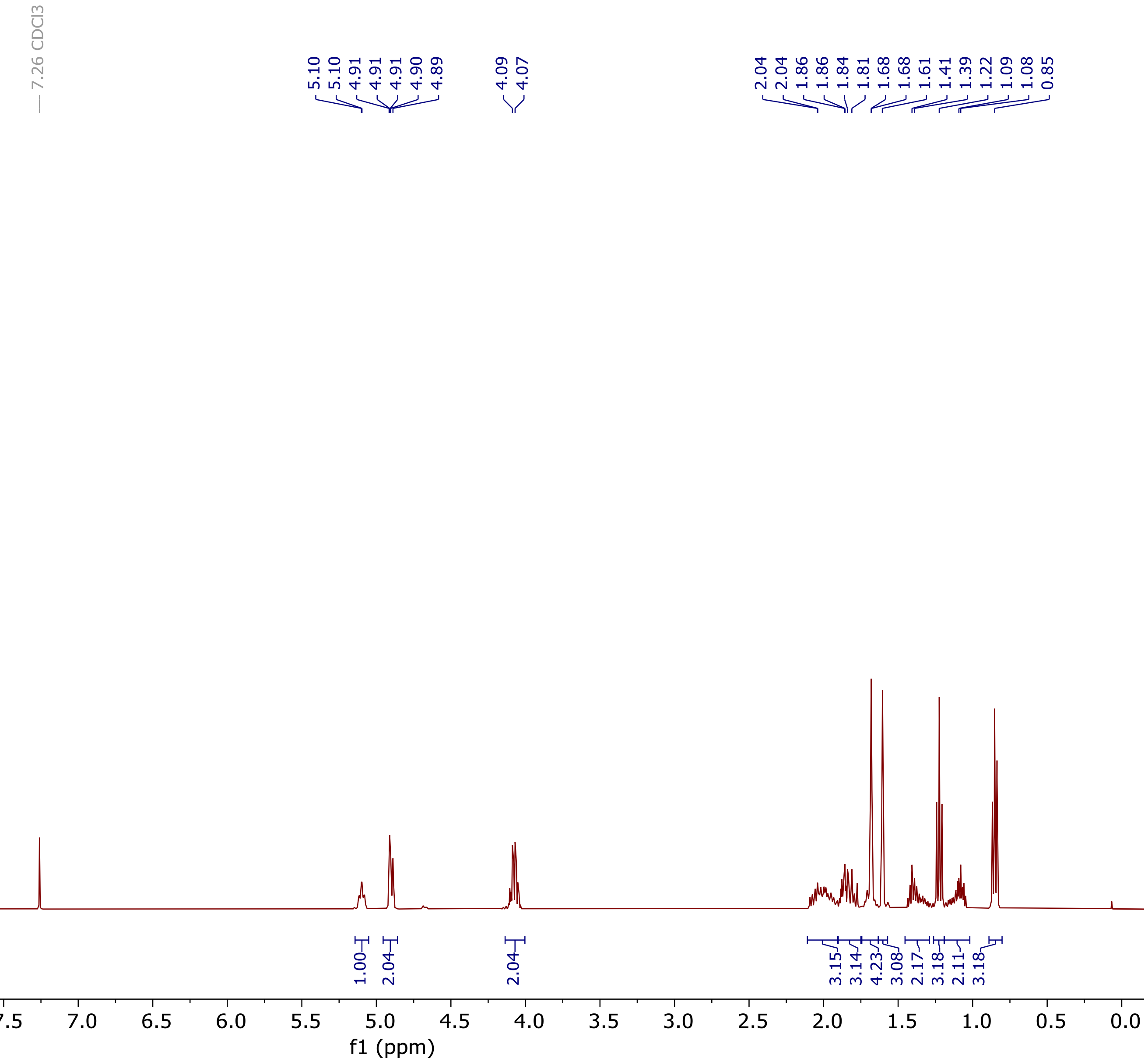

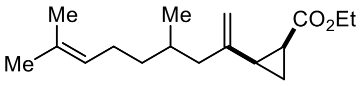

cis-**3ka**

$^{13}C\{^{1}H\}$ NMR ($CDCl_3$, 101 MHz)

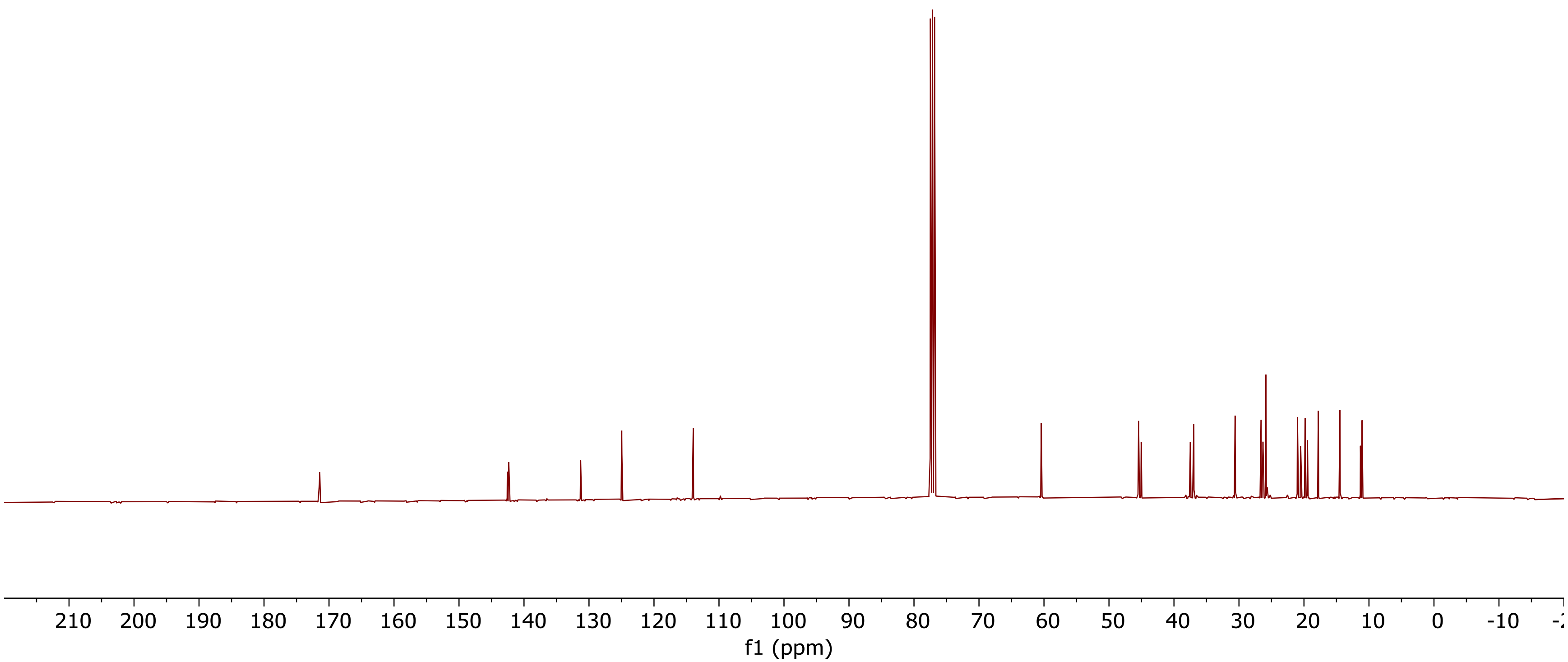

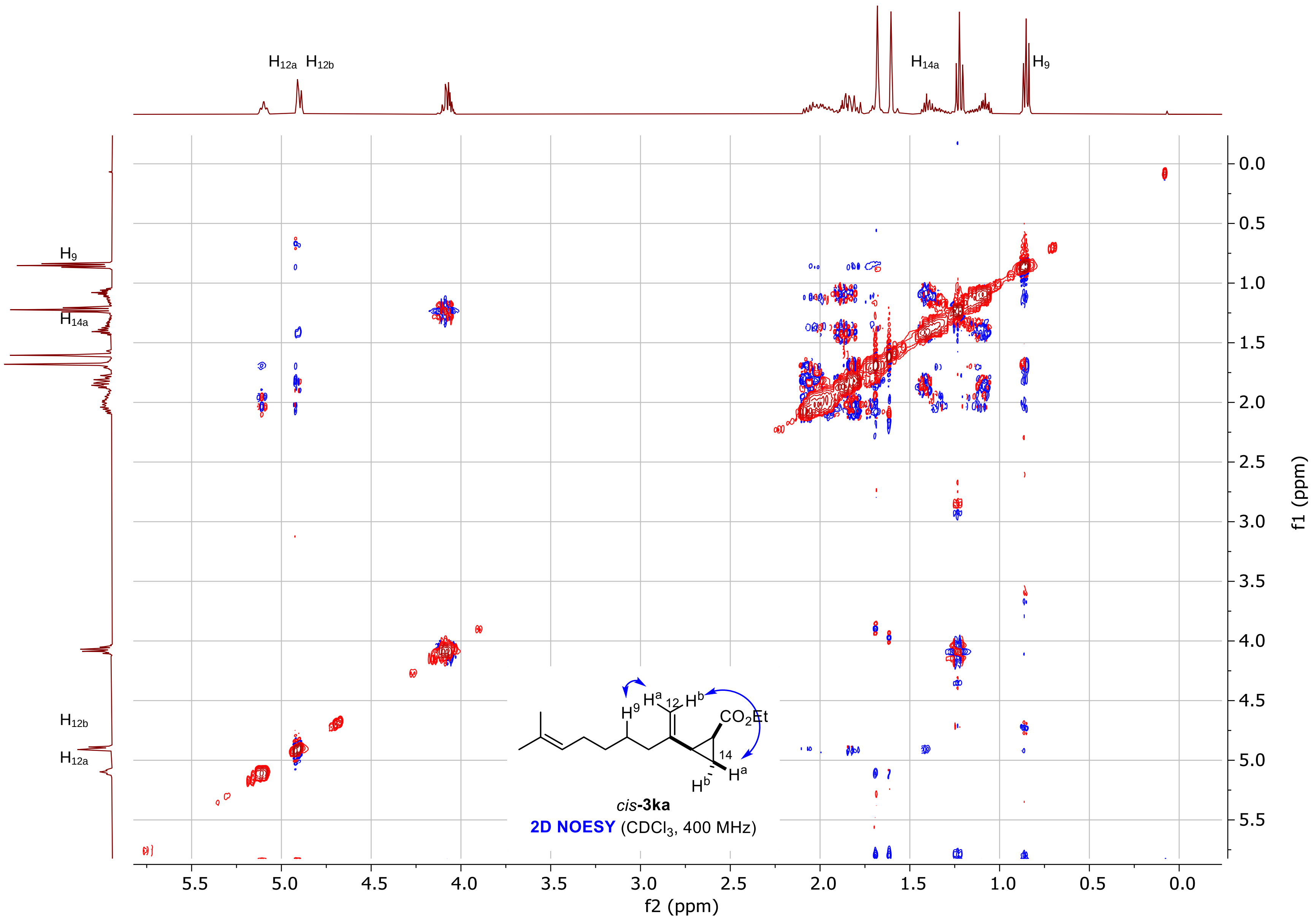

cis-**3ka**

2D NOESY ($CDCl_3$, 400 MHz)

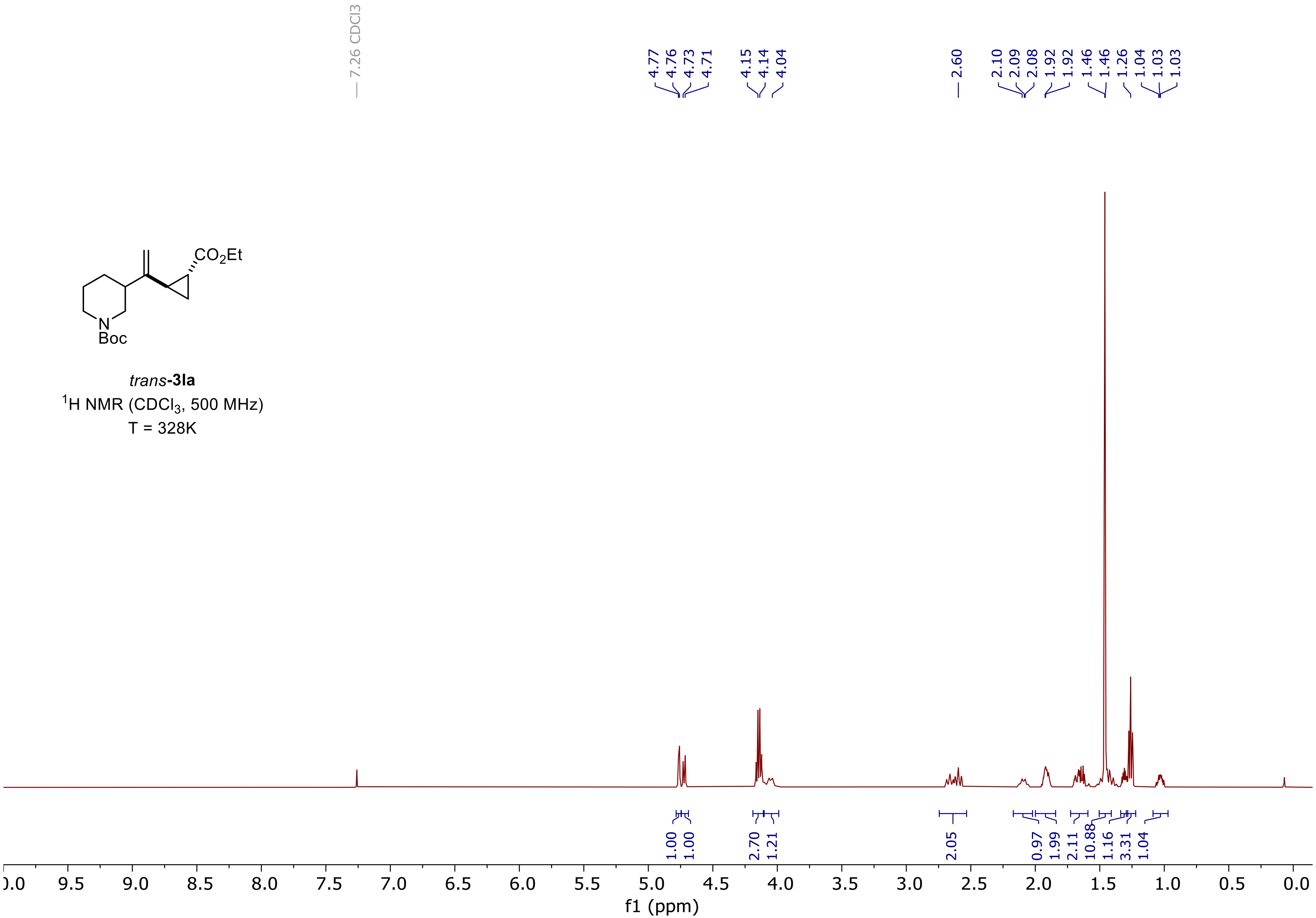

trans-**3la**
^{1}H NMR ($CDCl_3$, 500 MHz)
T = 328K

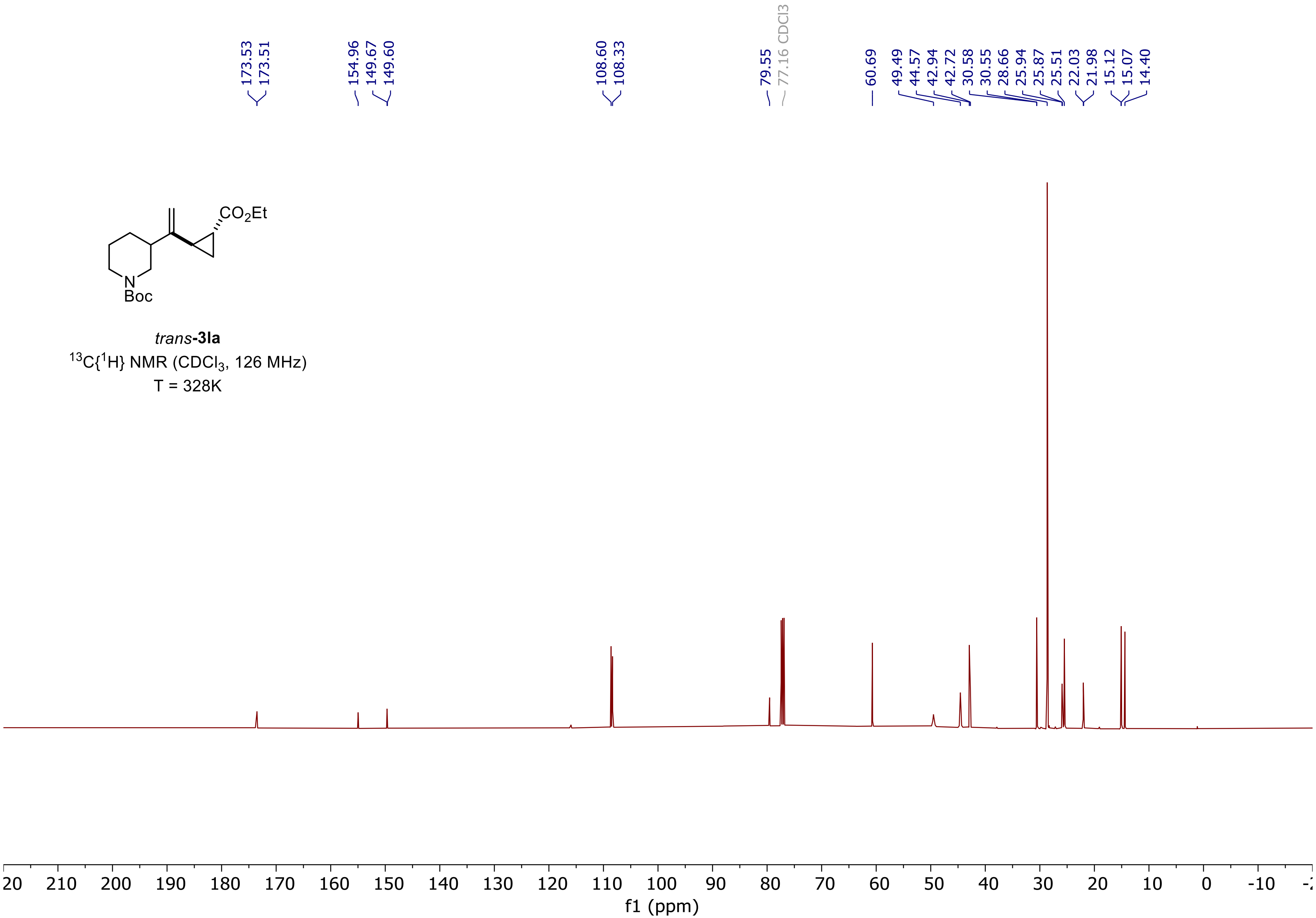

trans-**3la**
$^{13}C\{^{1}H\}$ NMR ($CDCl_3$, 126 MHz)
T = 328K

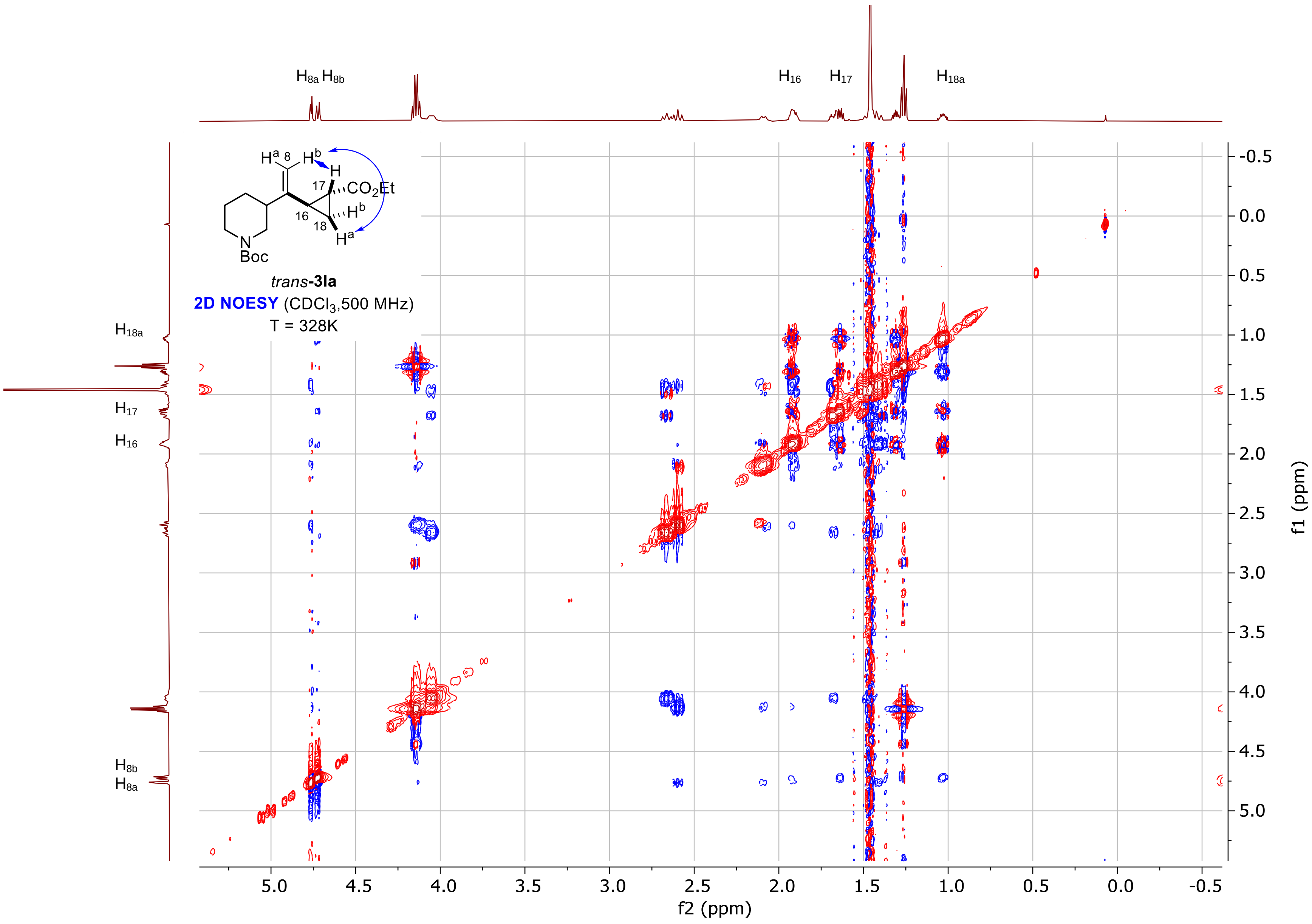

H8a H8b
H16
H17
H18a
trans-3la
2D NOESY (CDCl3,500 MHz)
T = 328K
Boc
CO2Et
f2 (ppm)
f1 (ppm)

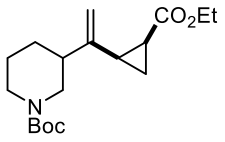

cis-3la
^{1}H NMR ($CDCl_3$, 500 MHz)
T = 328K

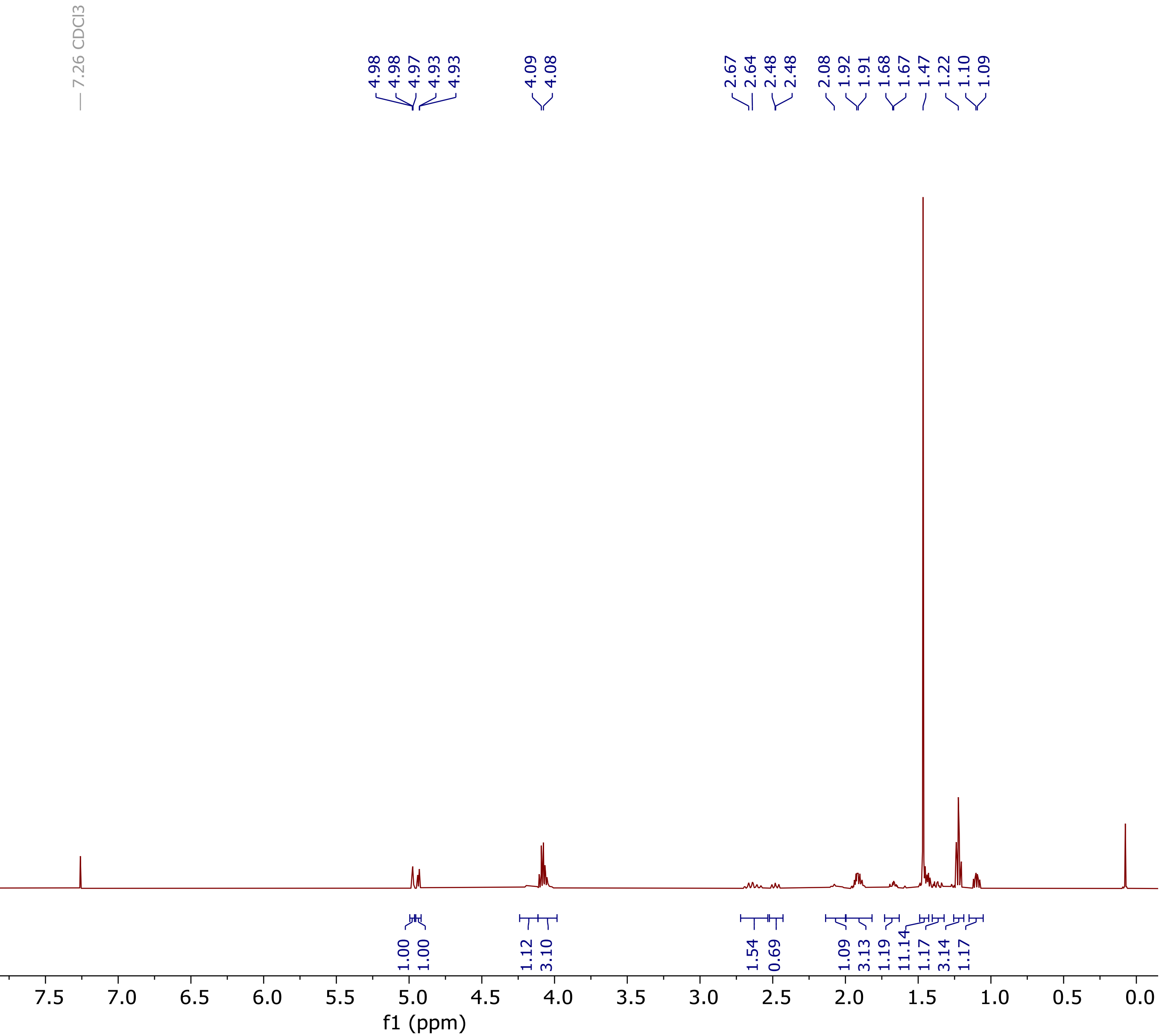

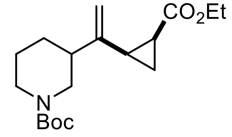

cis-3la

$^{13}C\{^{1}H\}$ NMR ($CDCl_3$, 126 MHz)

T = 328K

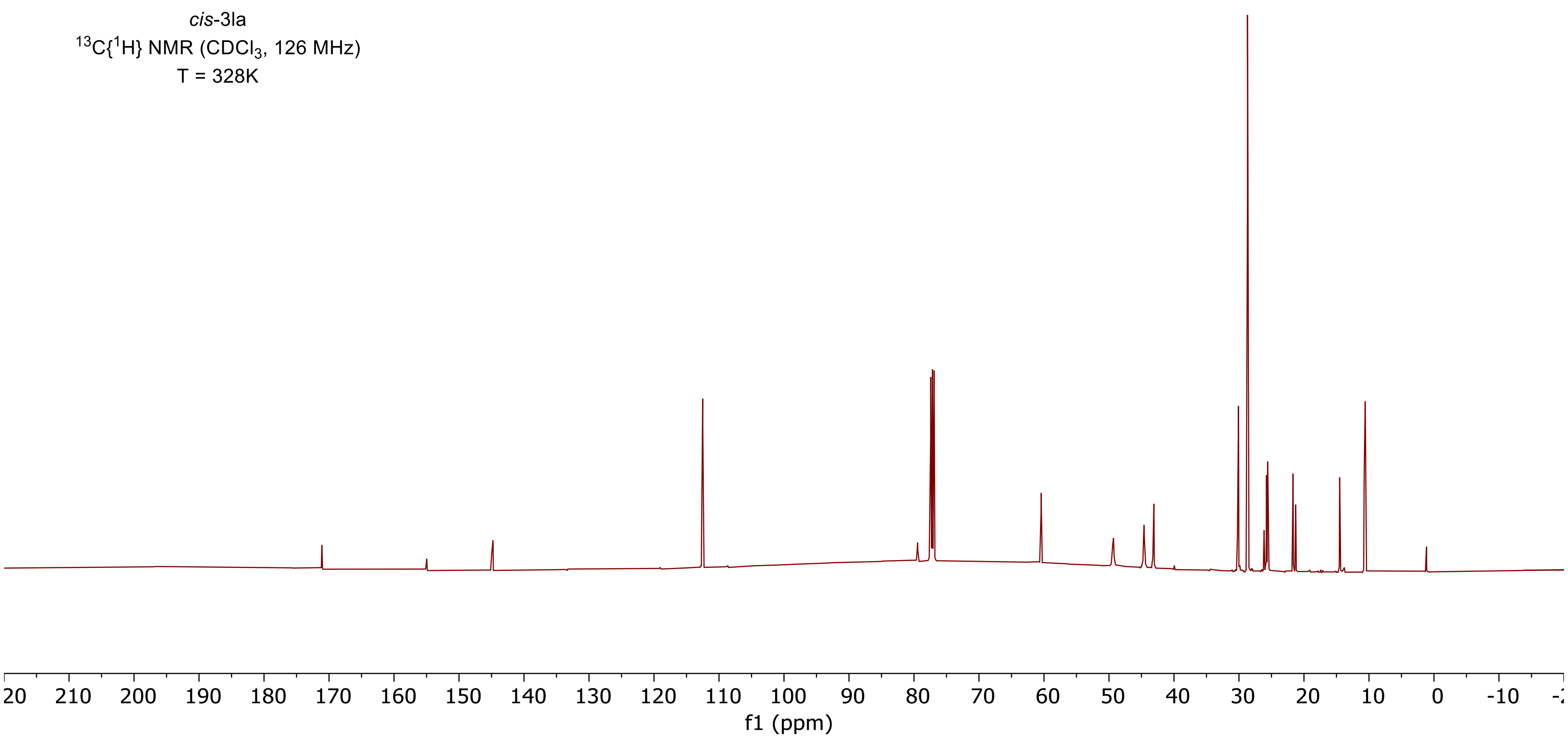

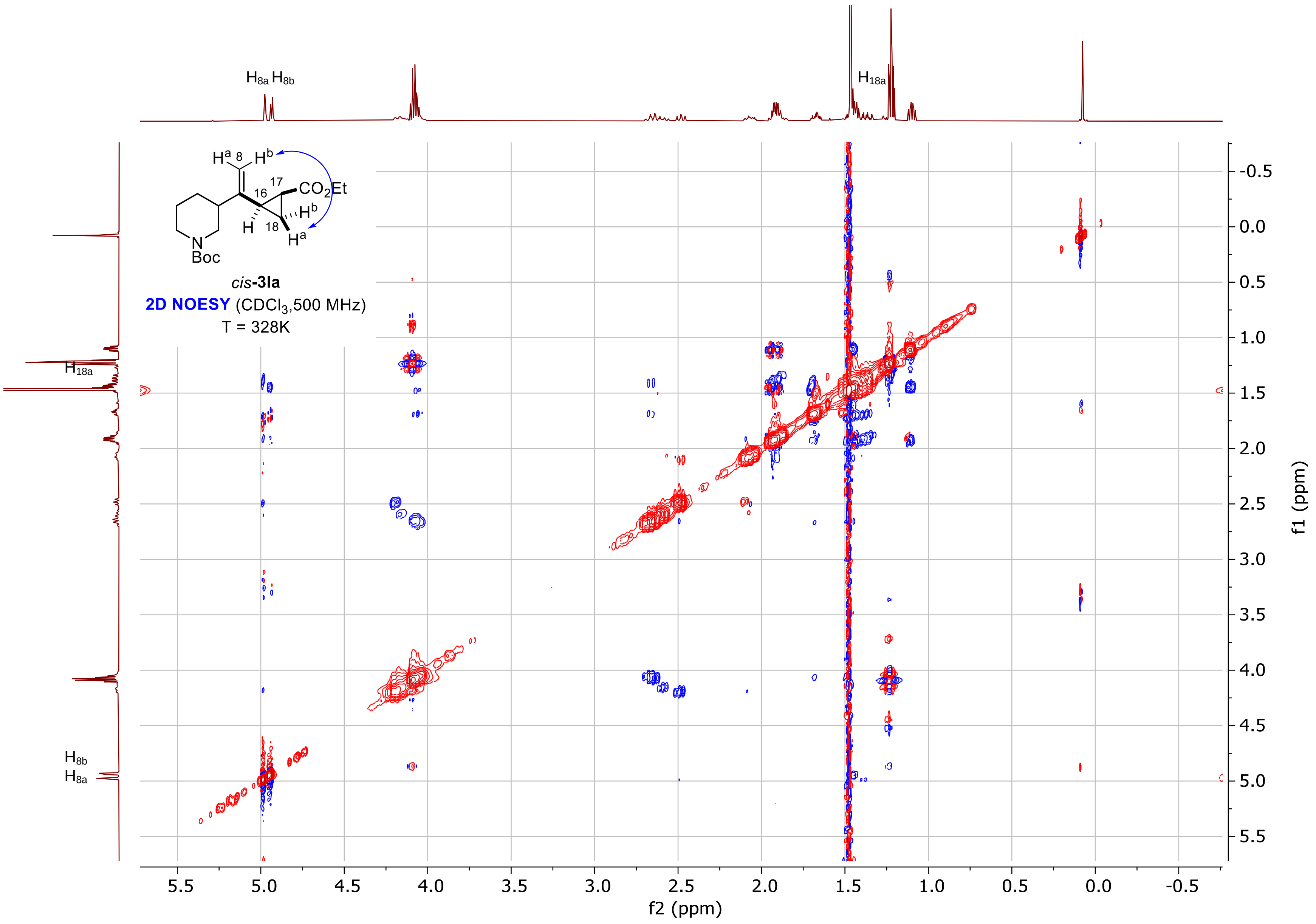
H8a H8b
H18a
cis-3la
2D NOESY (CDCl3,500 MHz)
T = 328K
f2 (ppm)
f1 (ppm)
H8b
H8a
Boc

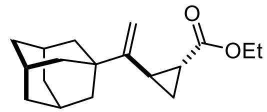

trans-**3ma**
^{1}H NMR ($CDCl_3$, 400 MHz)

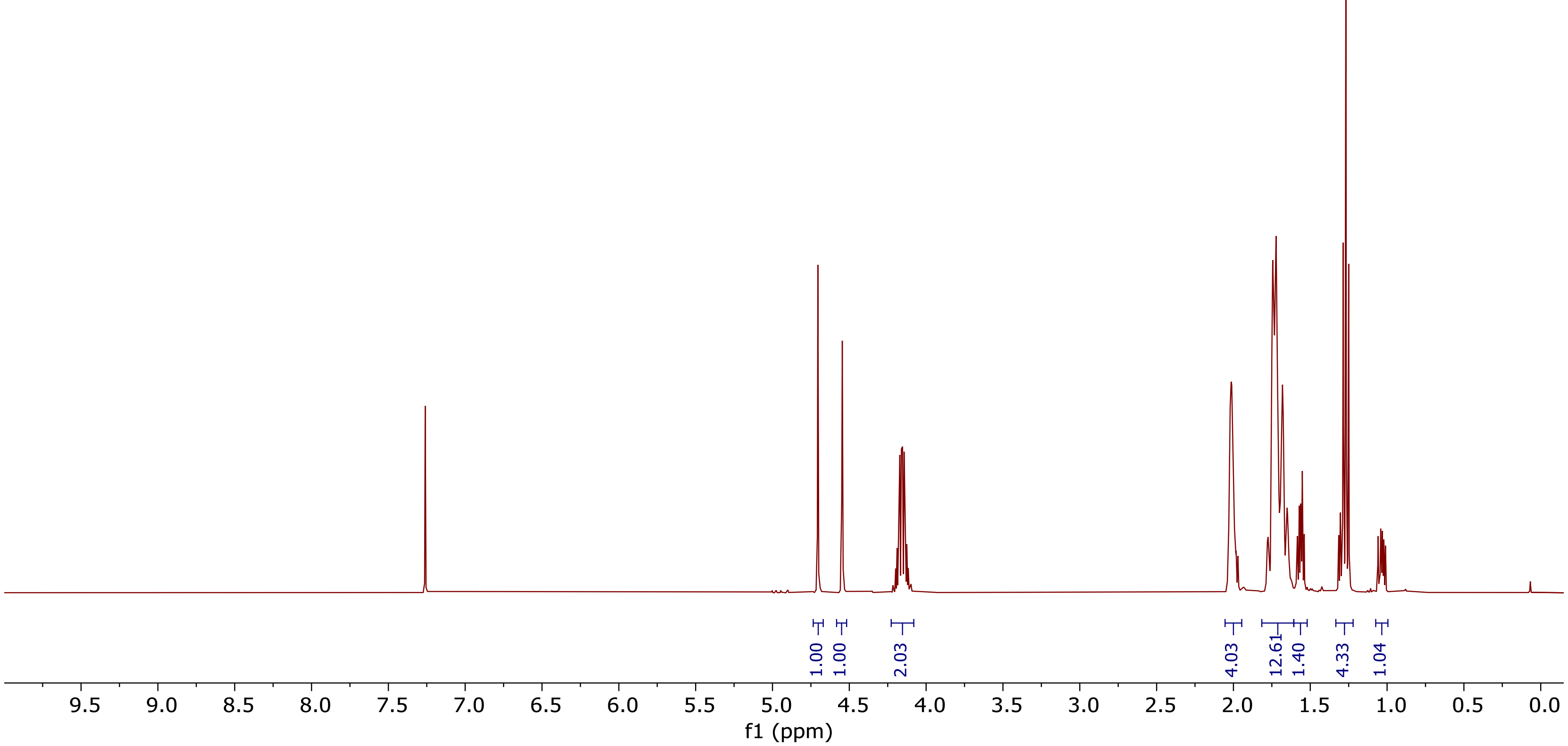

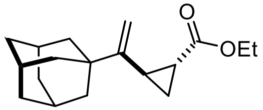

trans-**3ma**
$^{13}C\{^{1}H\}$ NMR ($CDCl_3$, 101 MHz)

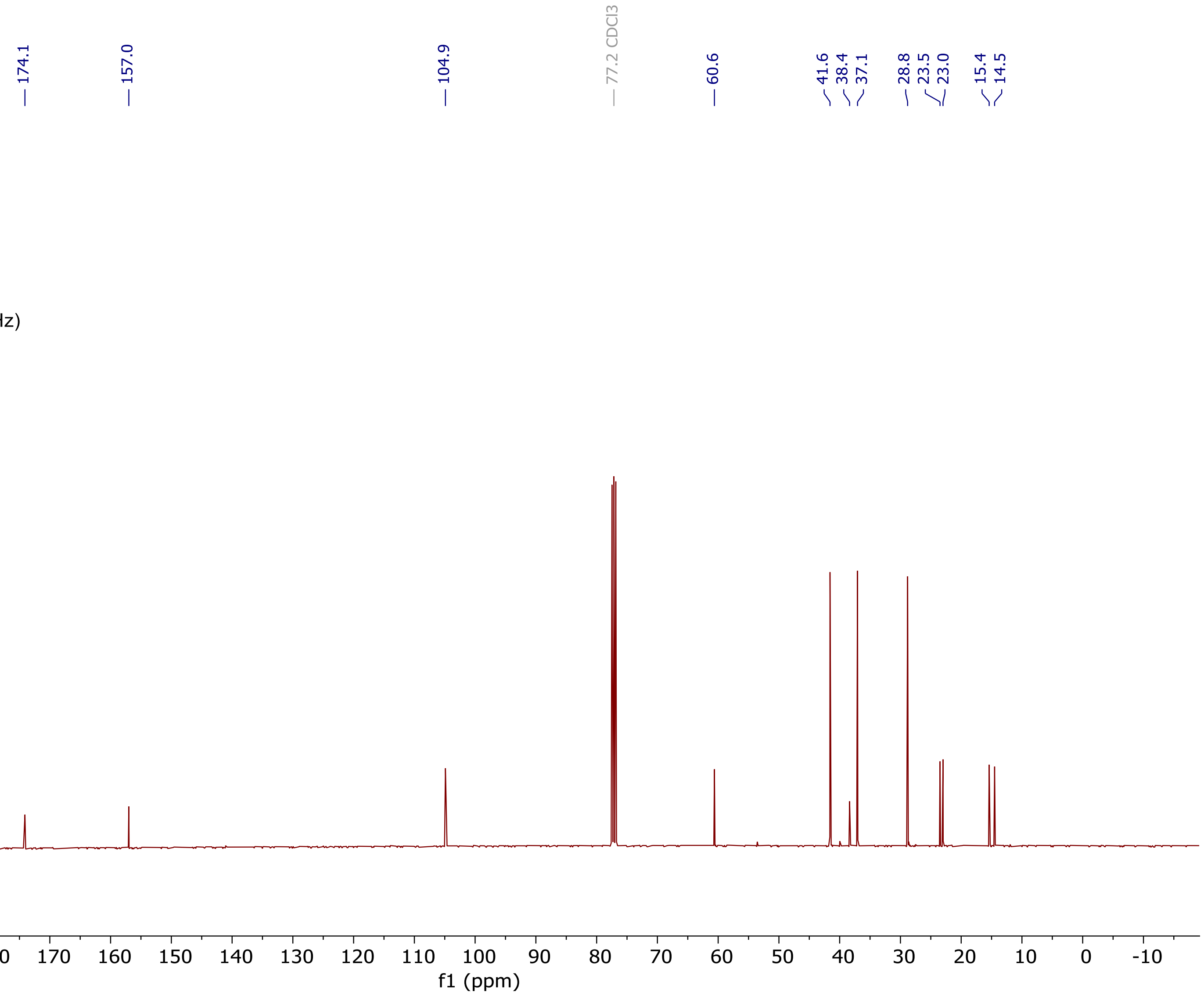

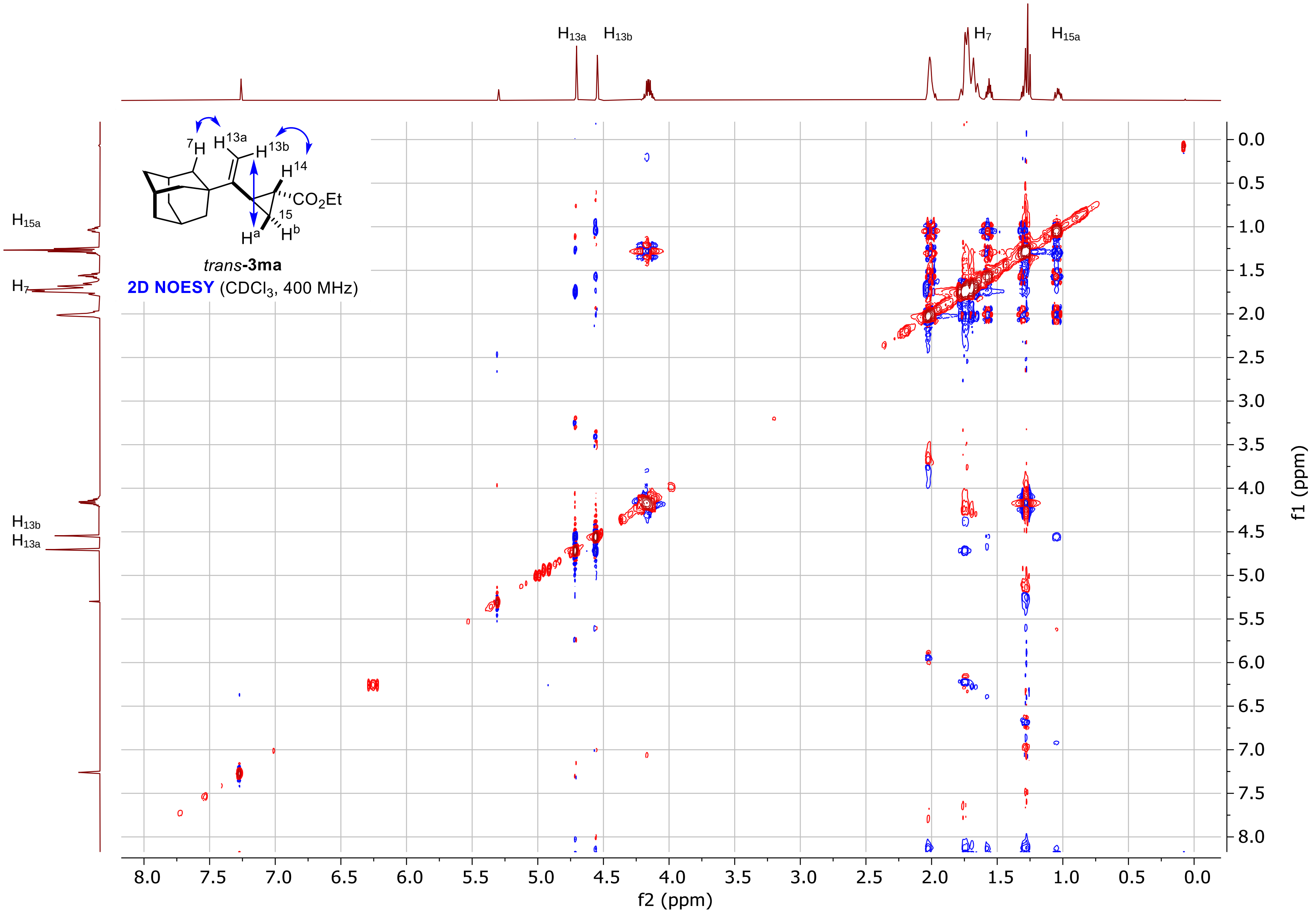

H13a
H13b
H7
H15a
7H
H13a
H13b
H14
CO2Et
15
Ha
Hb
trans-3ma
2D NOESY (CDCl3, 400 MHz)
f2 (ppm)
f1 (ppm)

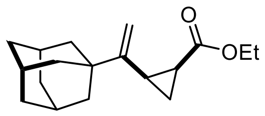

cis-**3ma**
^{1}H NMR ($CDCl_3$, 400 MHz)

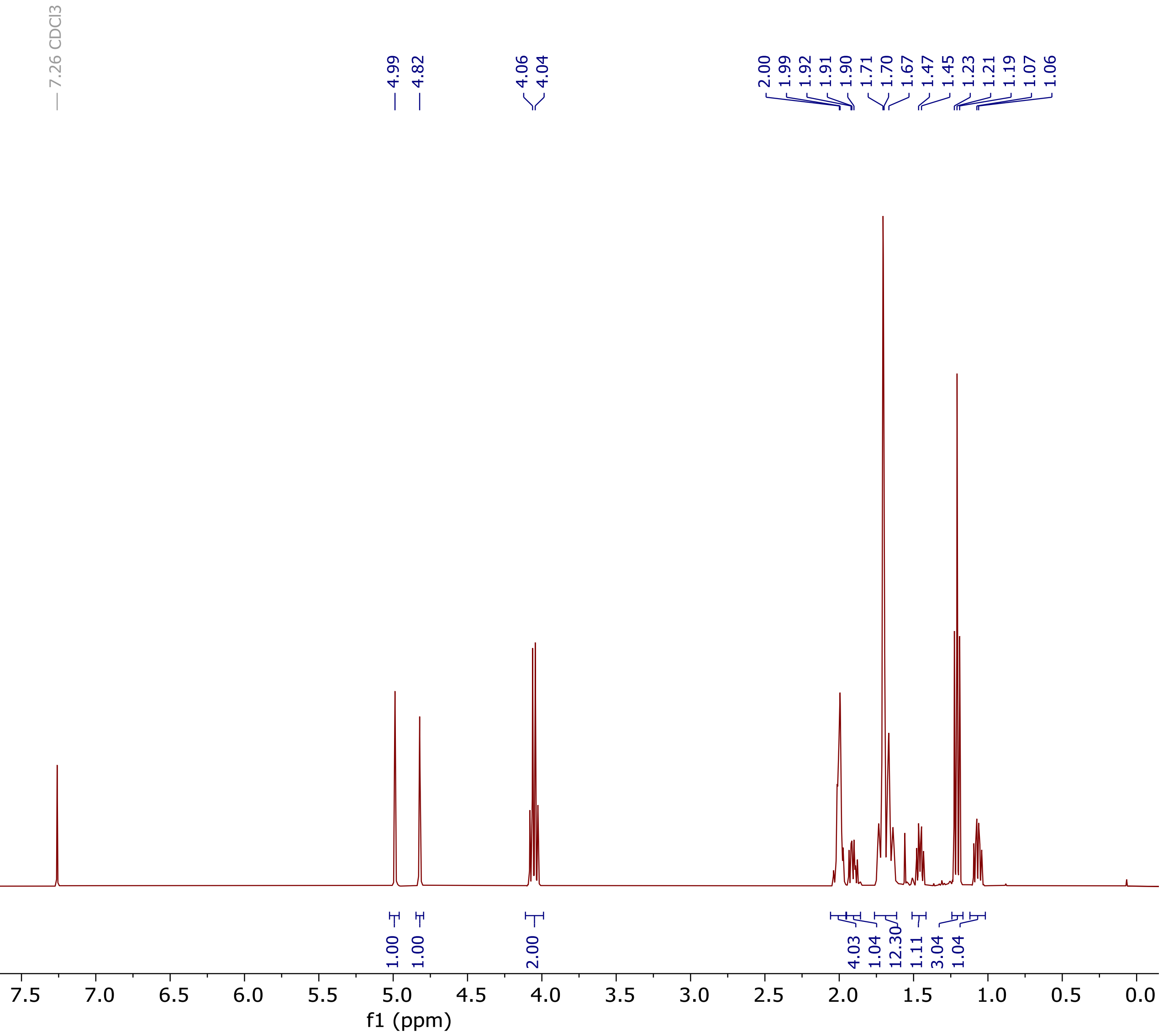

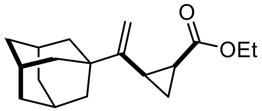

cis-**3ma**

$^{13}C\{^1H\}$ NMR ($CDCl_3$, 101 MHz)

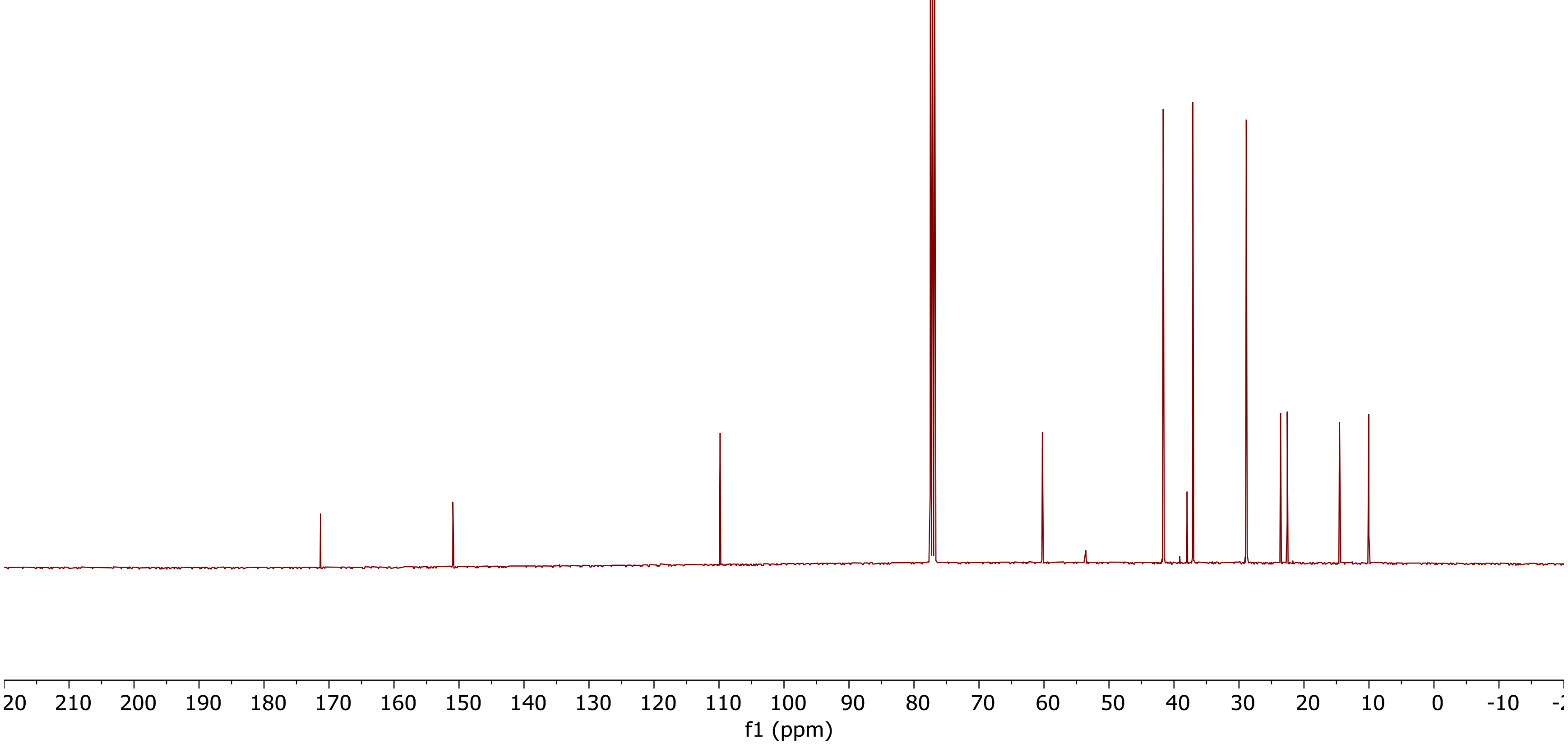

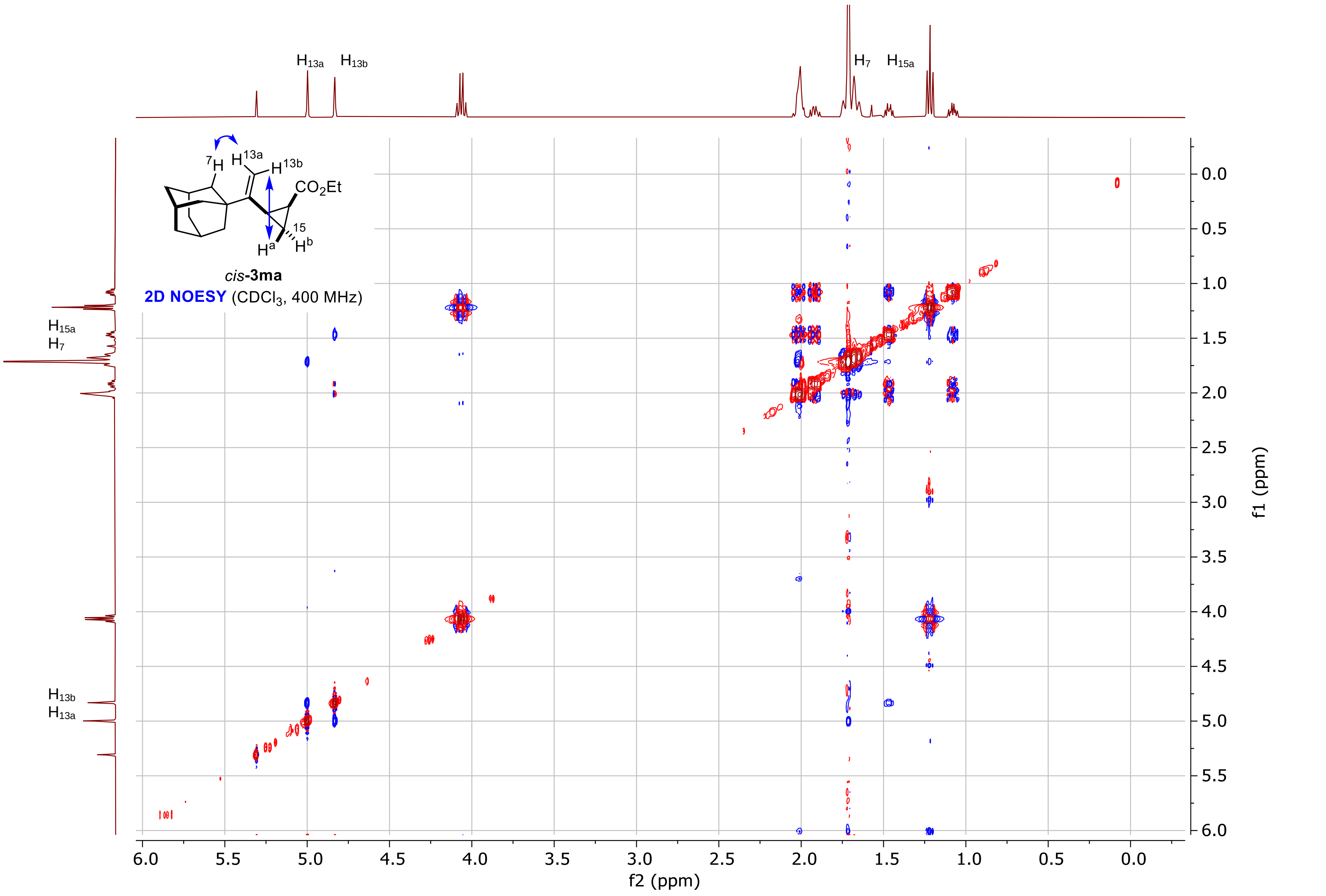
H13a
H13b
H7
H15a
7H
CO2Et
15
Ha
Hb
cis-3ma
2D NOESY (CDCl3, 400 MHz)
f2 (ppm)
f1 (ppm)

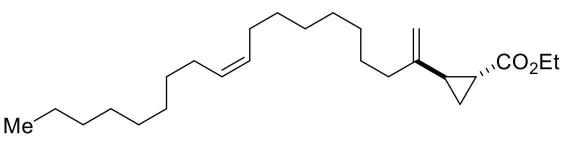

trans-**3na**

^{1}H NMR ($CDCl_3$, 400 MHz)

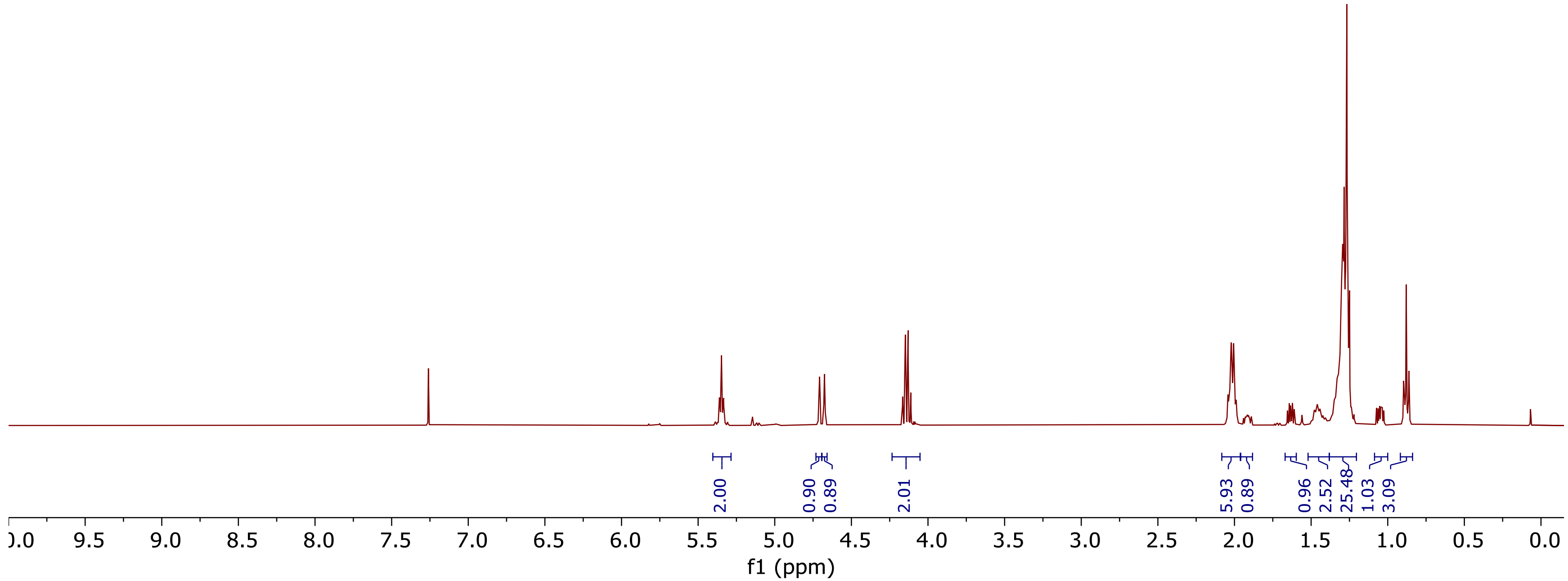

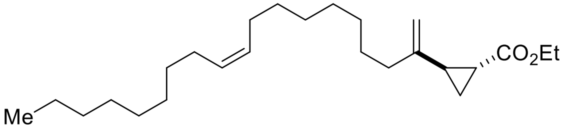

trans-**3na**

$^{13}C\{^1H\}$ NMR ($CDCl_3$, 101 MHz)

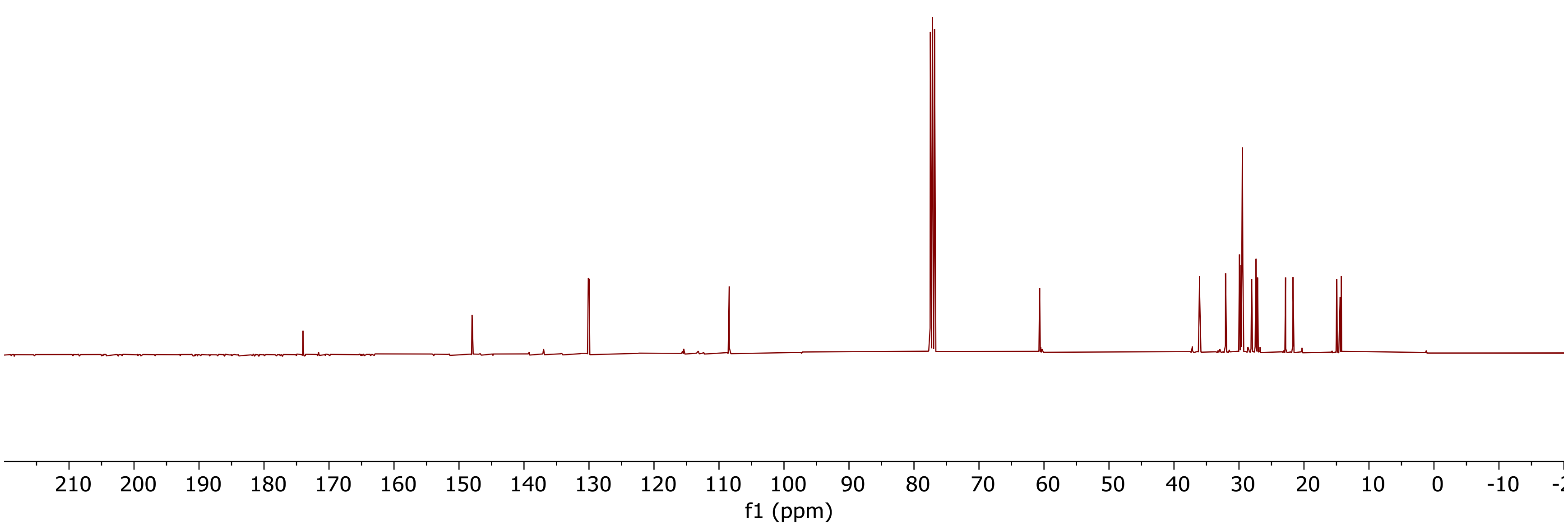

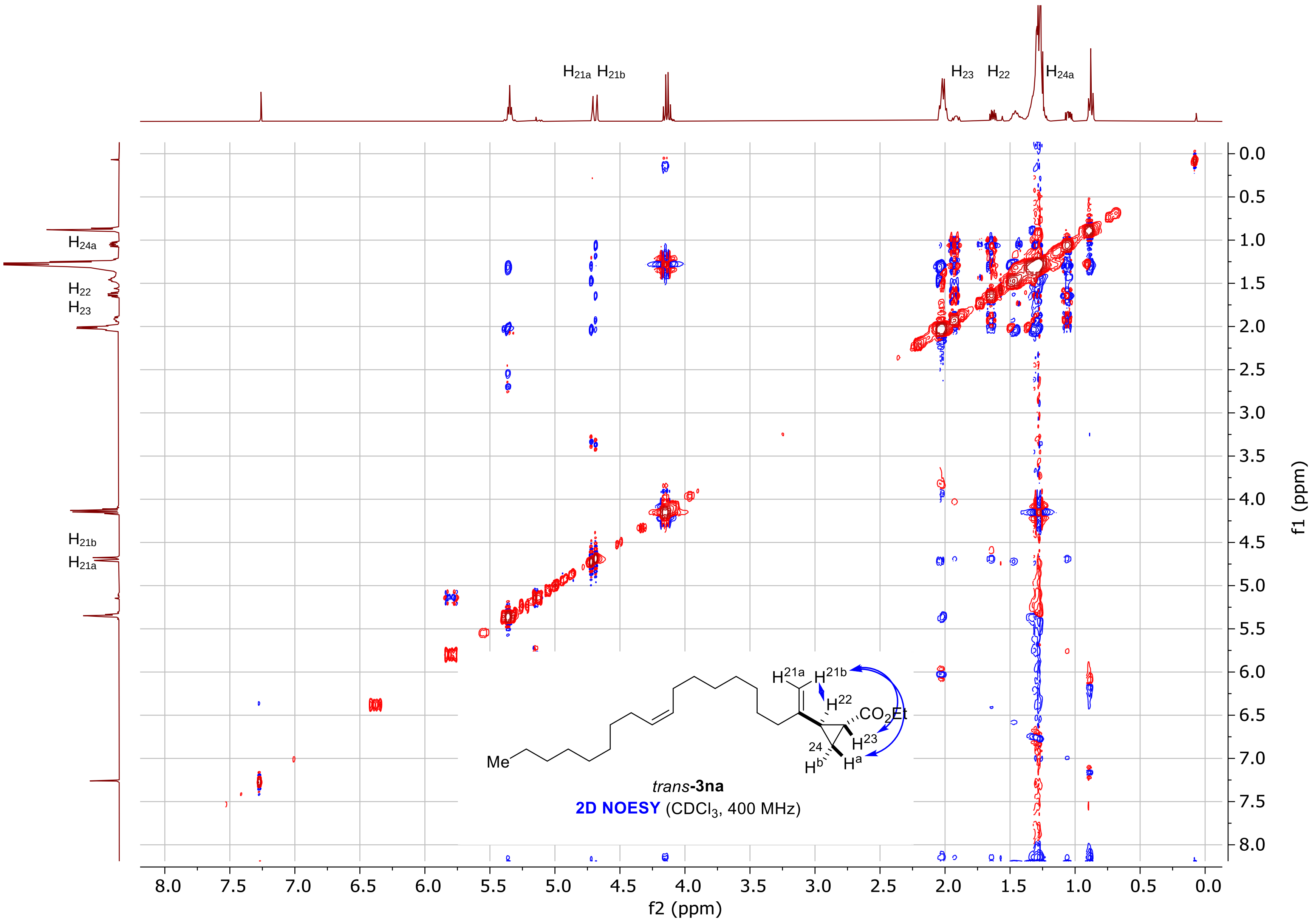
H21a H21b
H23 H22 H24a
H24a
H22
H23
H21b
H21a
H21a H21b
H22
CO2Et
H23
24
Hb Ha
Me
trans-**3na**
2D NOESY (CDCl3, 400 MHz)
f2 (ppm)
f1 (ppm)

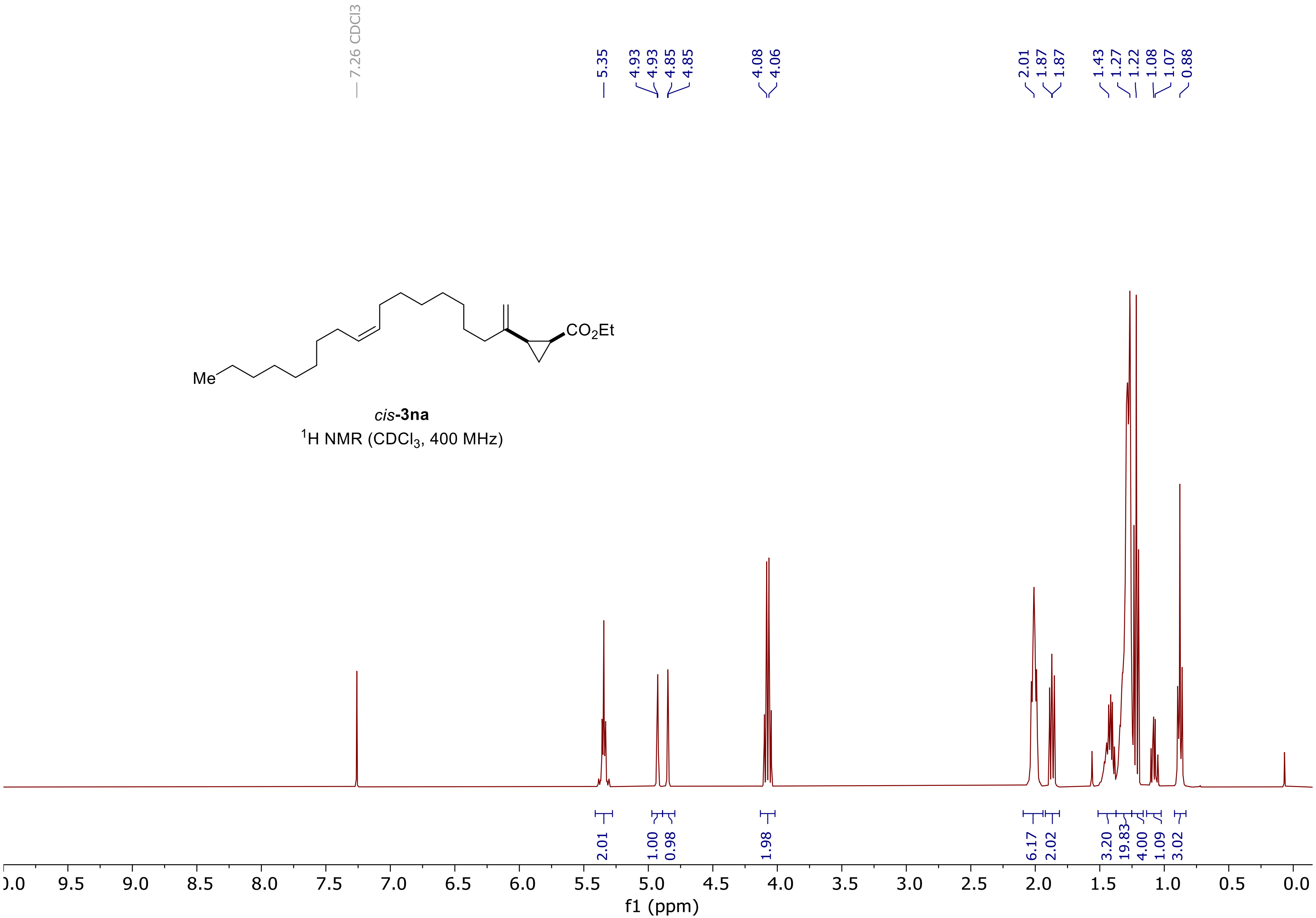

cis-**3na**
^{1}H NMR ($CDCl_3$, 400 MHz)

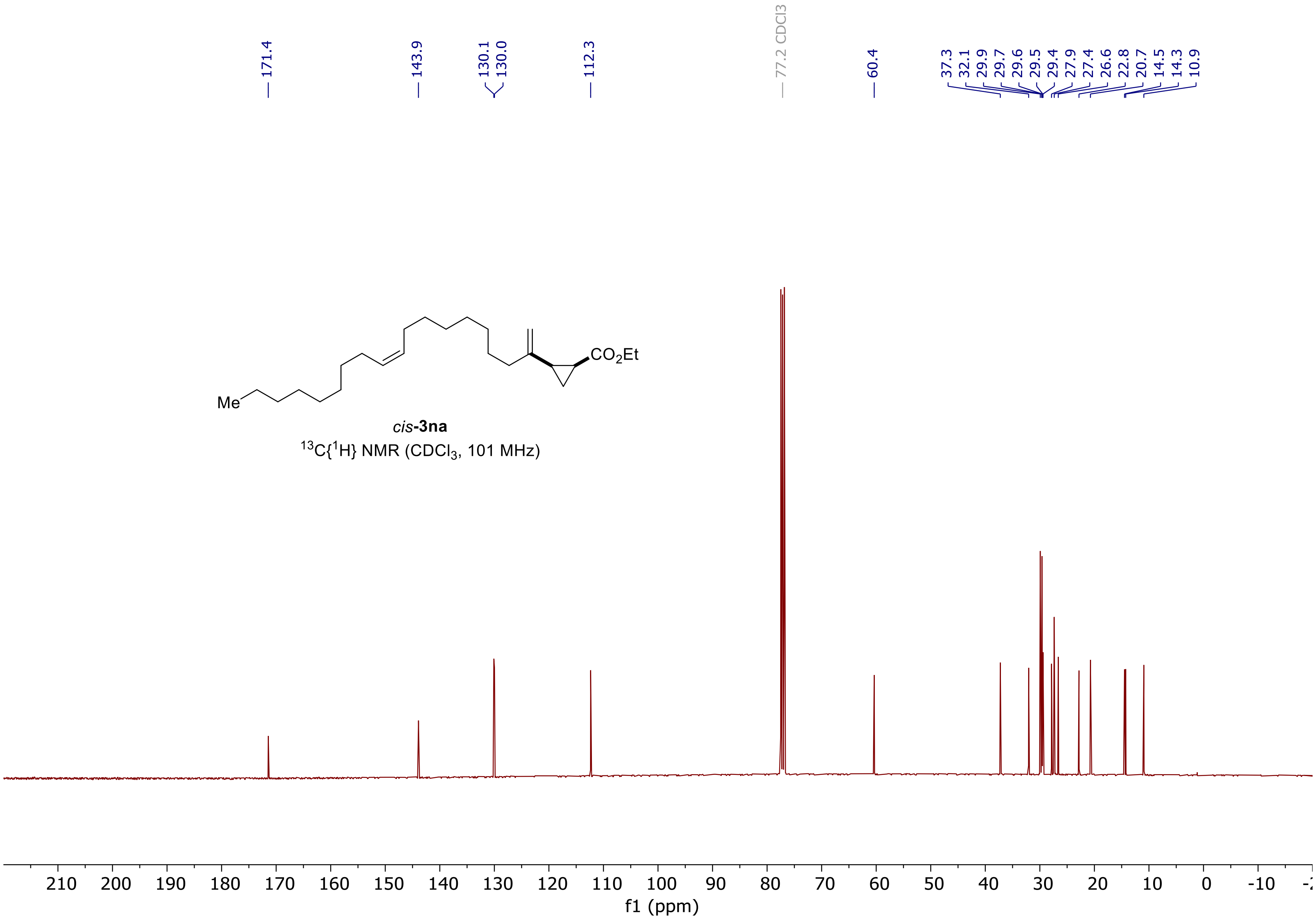

cis-**3na**

$^{13}C\{^{1}H\}$ NMR ($CDCl_3$, 101 MHz)

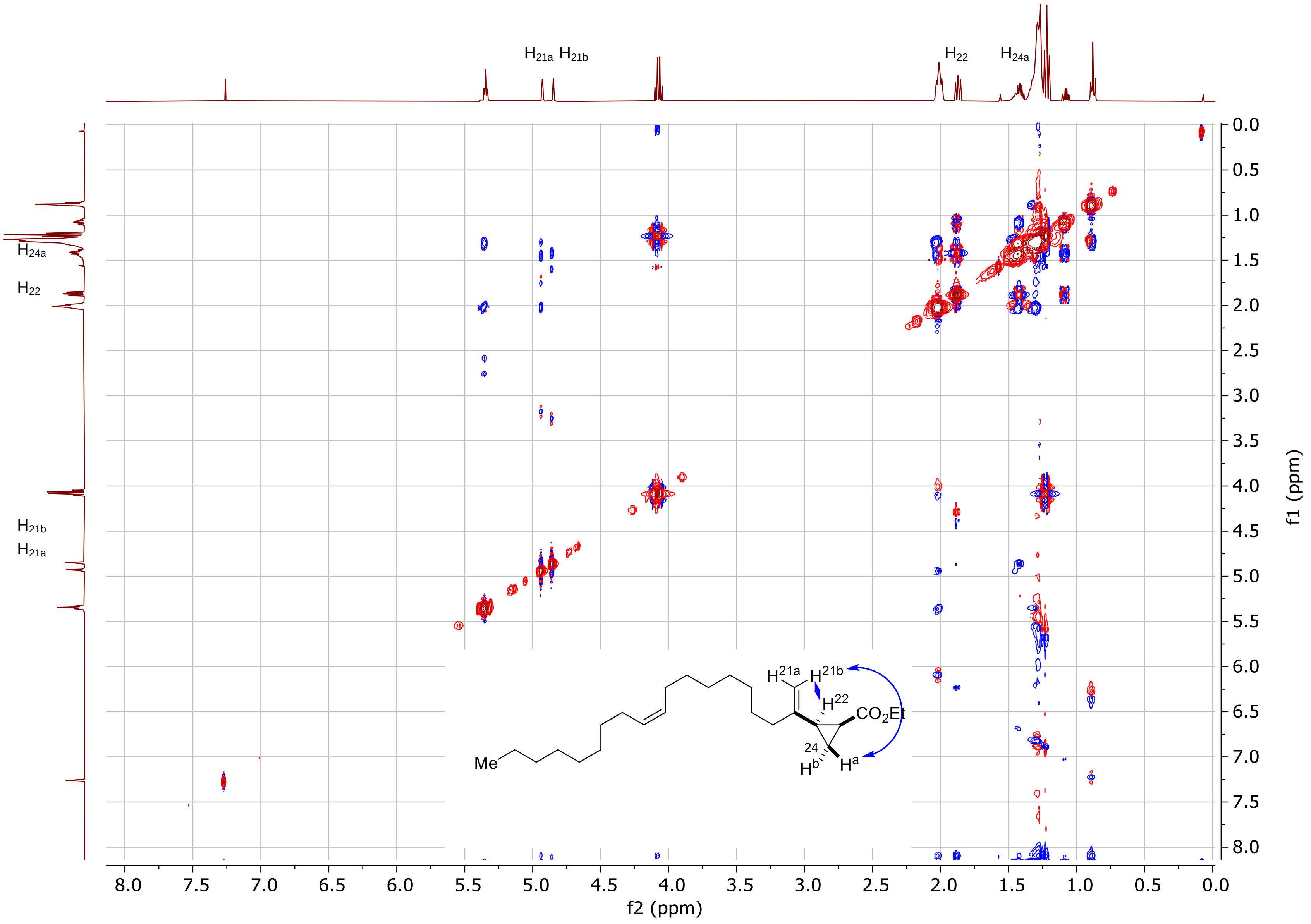
H21a H21b
H22
H24a
H24a
H22
H21b
H21a
f2 (ppm)
f1 (ppm)
H21a H21b
H22
CO2Et
24
Hb
Ha
Me

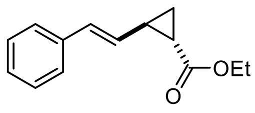

trans-**3oa**
^{1}H NMR ($CDCl_3$, 400 MHz)

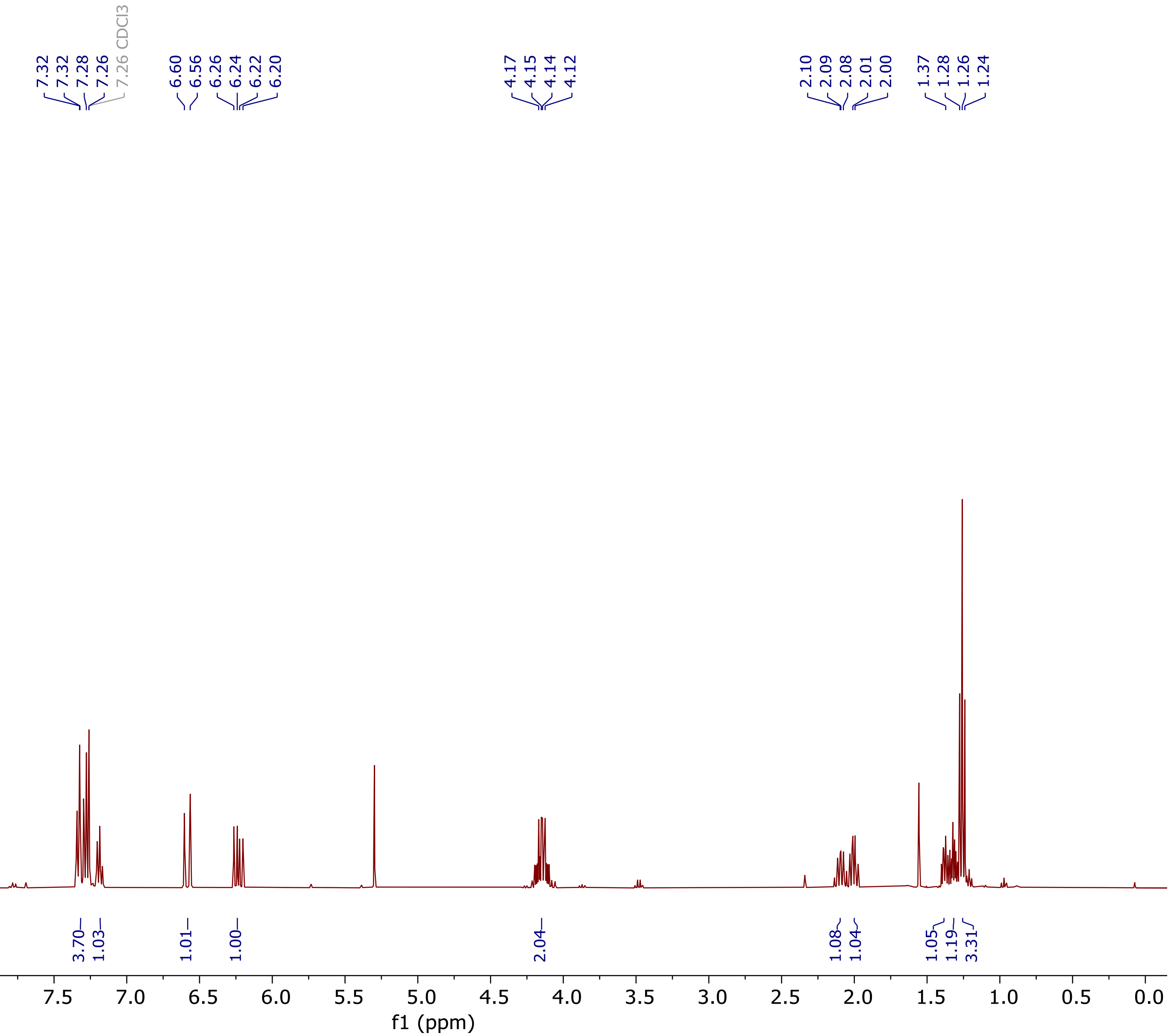

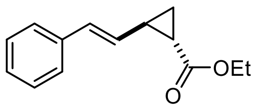

trans-**3oa**

$^{13}C\{^1H\}$ NMR ($CDCl_3$, 101 MHz)

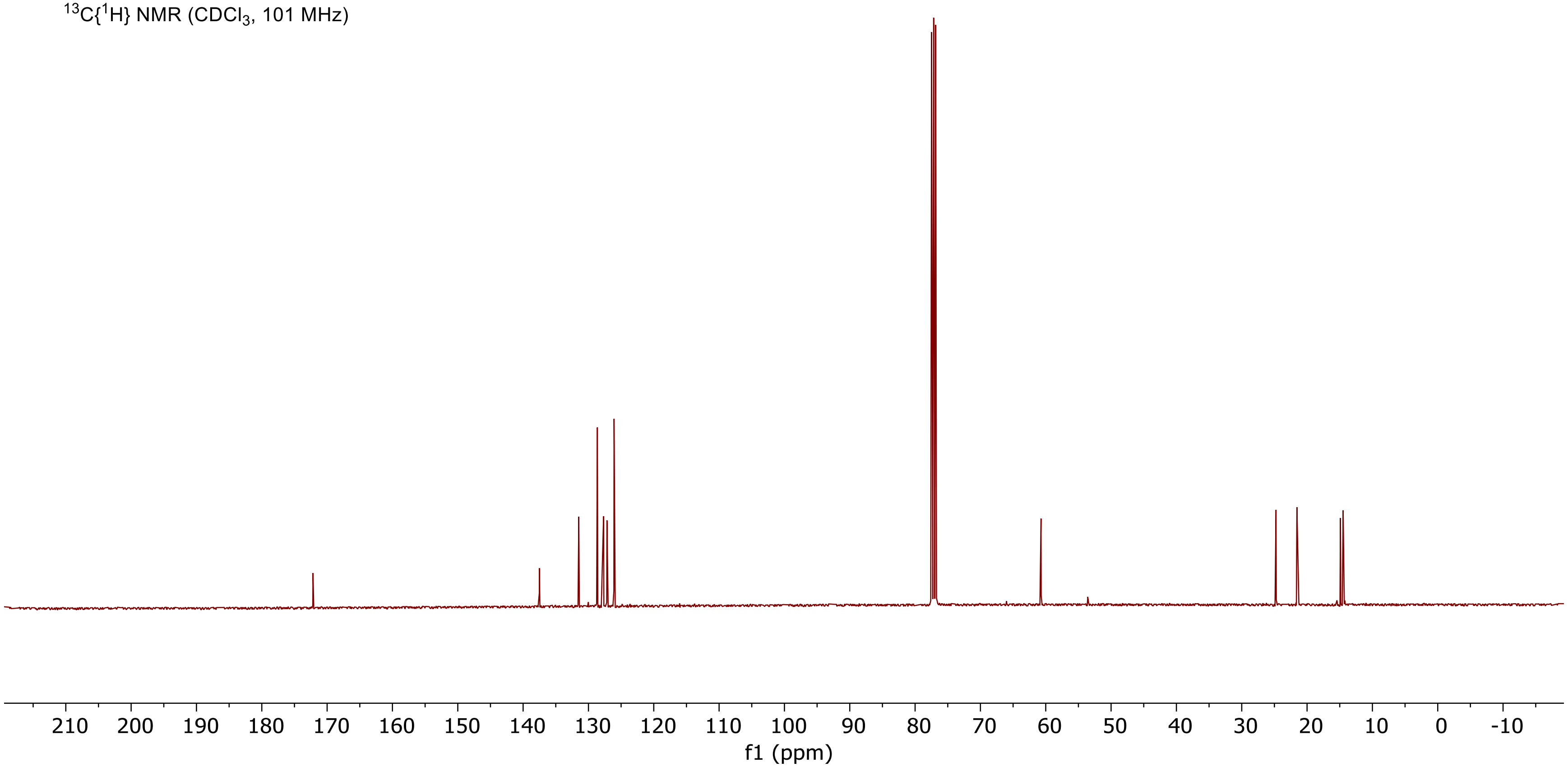

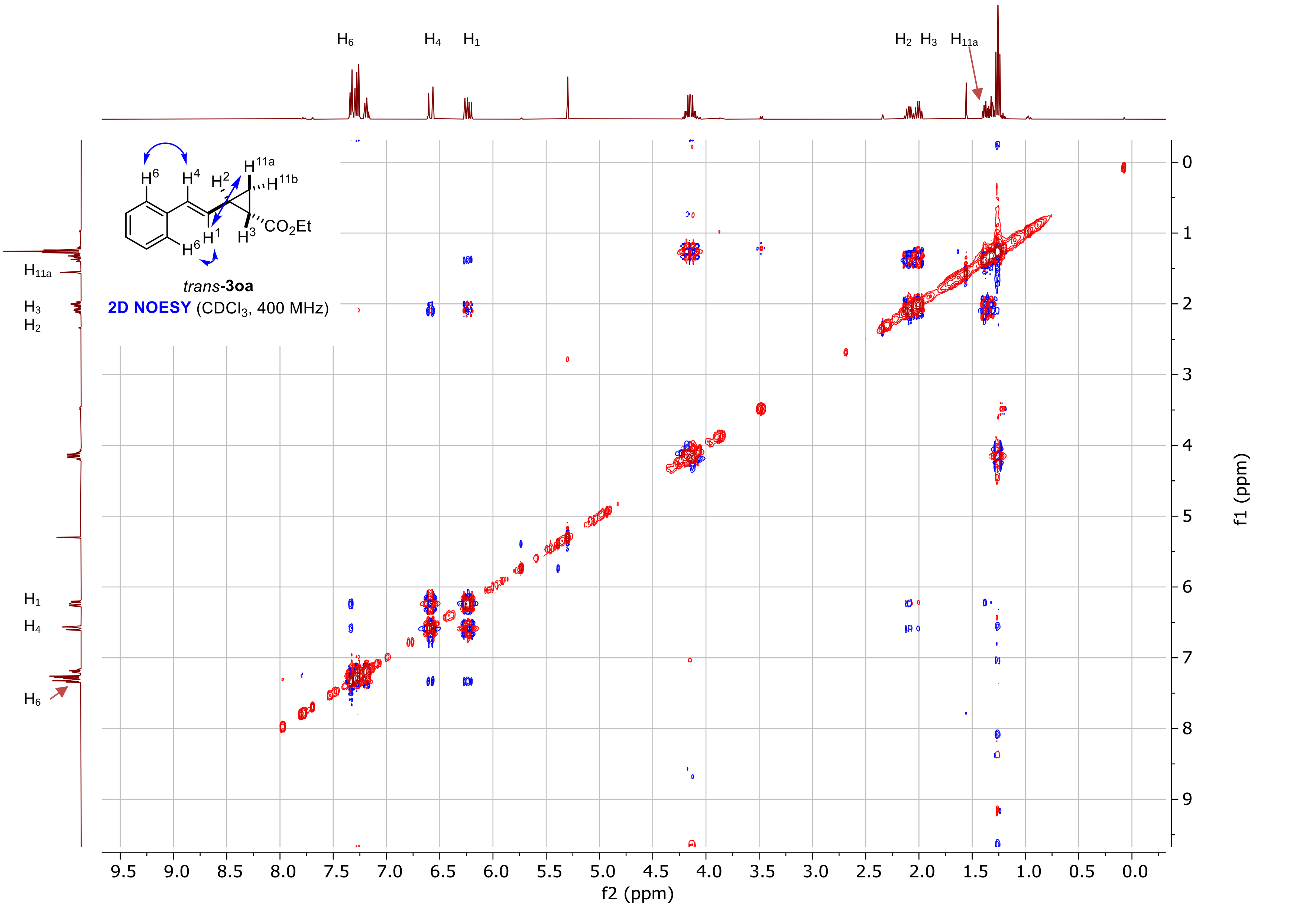

H6 H4 H1 H2 H3 H11a
H6 H4 H2 H11a H11b H6 H1 H3 CO2Et
trans-3oa
2D NOESY (CDCl3, 400 MHz)
H11a
H3
H2
H1
H4
H6
f2 (ppm)
f1 (ppm)

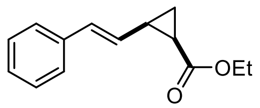

cis-**3oa**
^{1}H NMR ($CDCl_3$, 400 MHz)

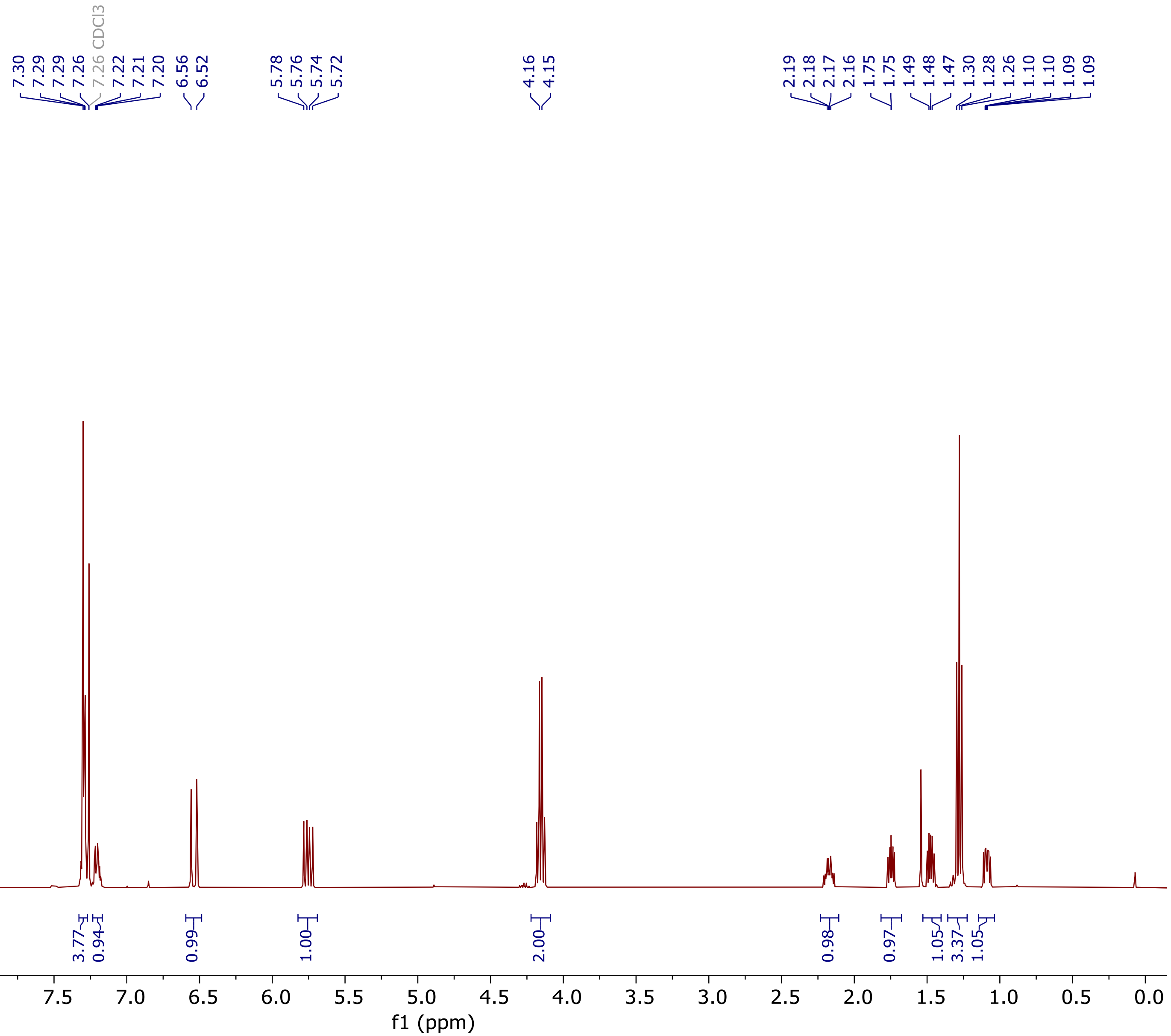

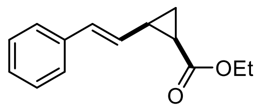

cis-**3oa**

$^{13}C\{^{1}H\}$ NMR ($CDCl_3$, 101 MHz)

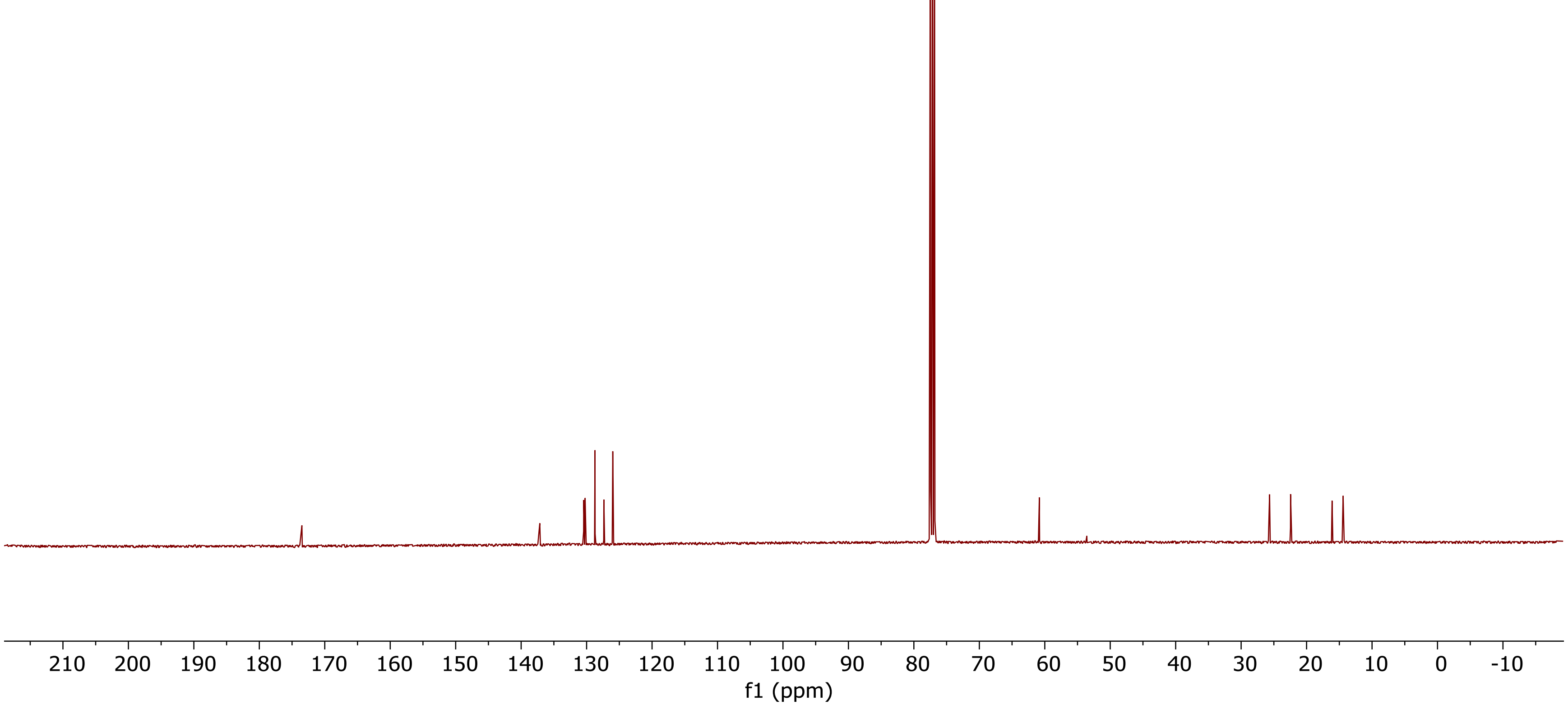

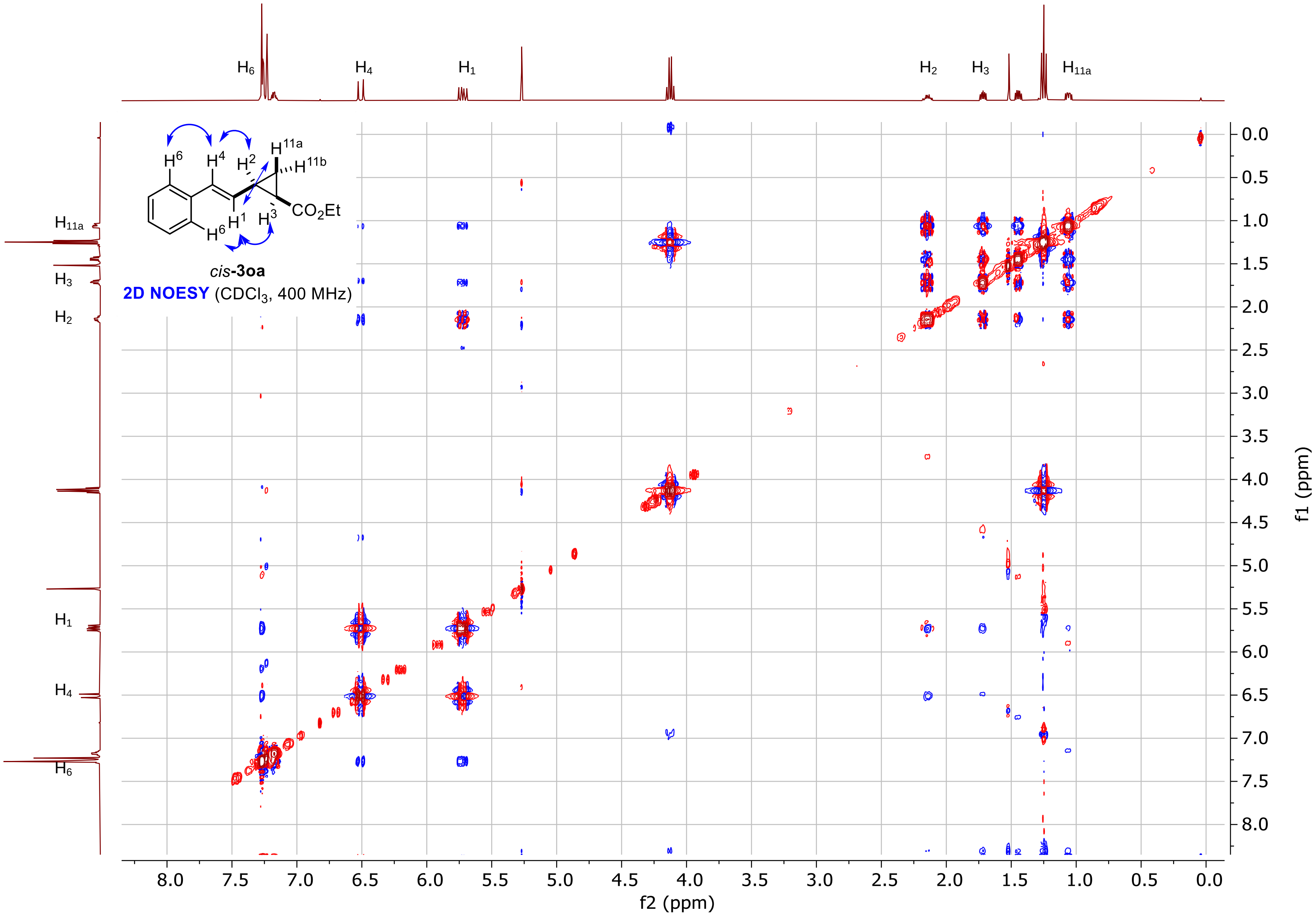
H6
H4
H1
H2
H3
H11a
H11a
H11b
CO2Et
cis-3oa
2D NOESY (CDCl3, 400 MHz)
f2 (ppm)
f1 (ppm)

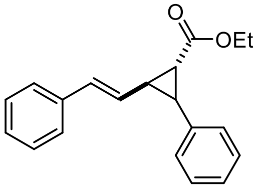

3pa

^{1}H NMR ($CDCl_3$, 400 MHz)

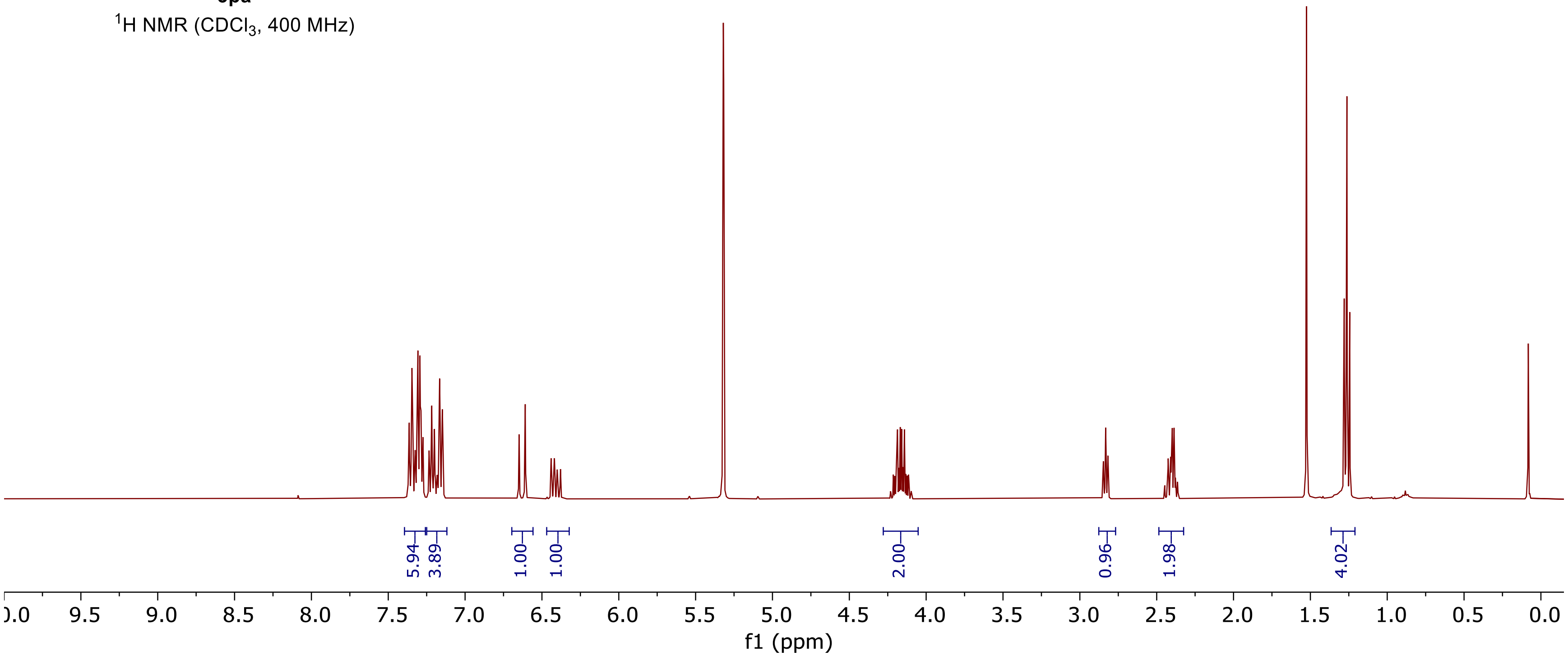

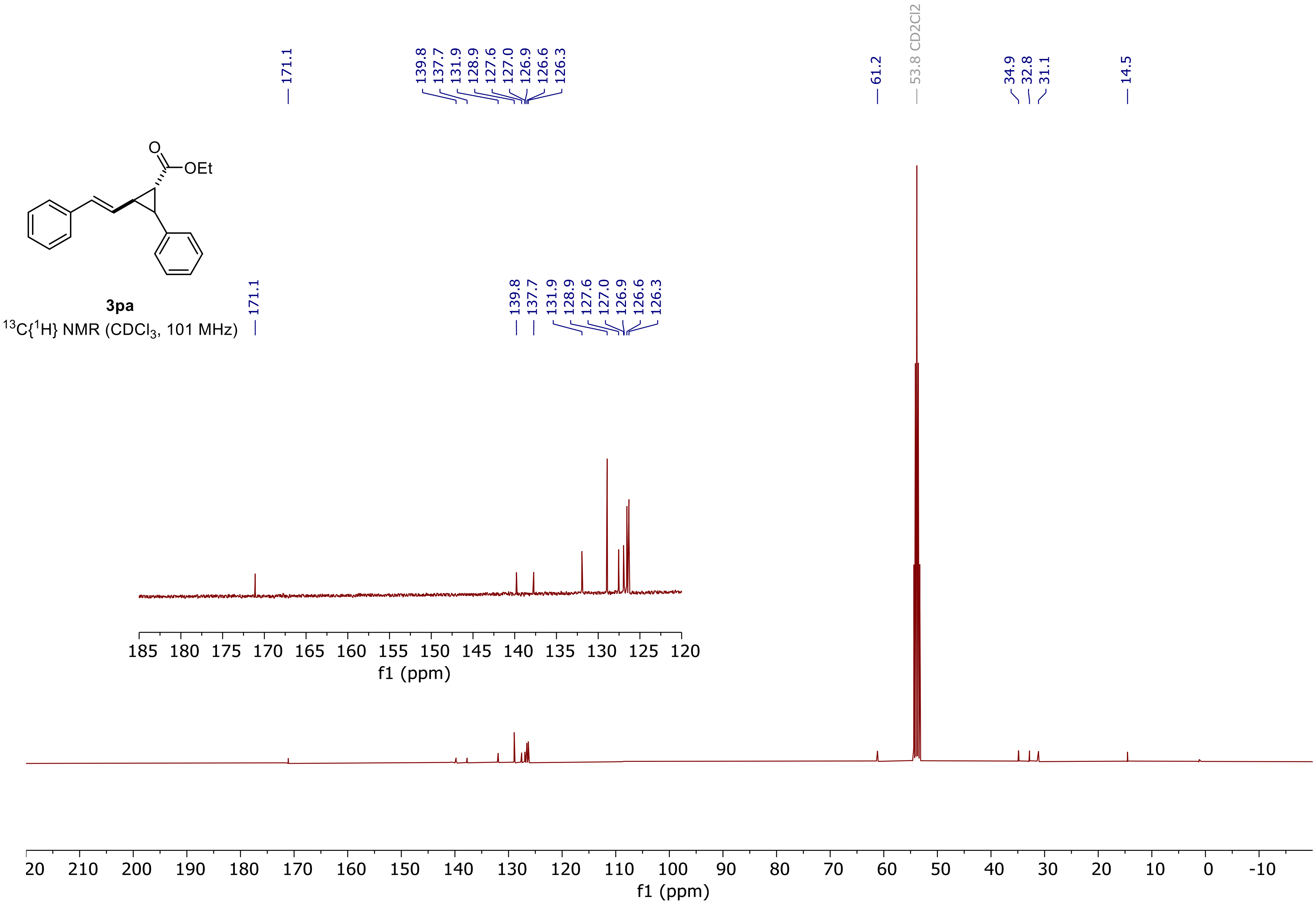
171.1
139.8
137.7
131.9
128.9
127.6
127.0
126.9
126.6
126.3
61.2
53.8 CD2Cl2
34.9
32.8
31.1
14.5
O
OEt
3pa
$^{13}C\{^{1}H\}$ NMR ($CDCl_3$, 101 MHz)
185 180 175 170 165 160 155 150 145 140 135 130 125 120
f1 (ppm)
20 210 200 190 180 170 160 150 140 130 120 110 100 90 80 70 60 50 40 30 20 10 0 -10
f1 (ppm)

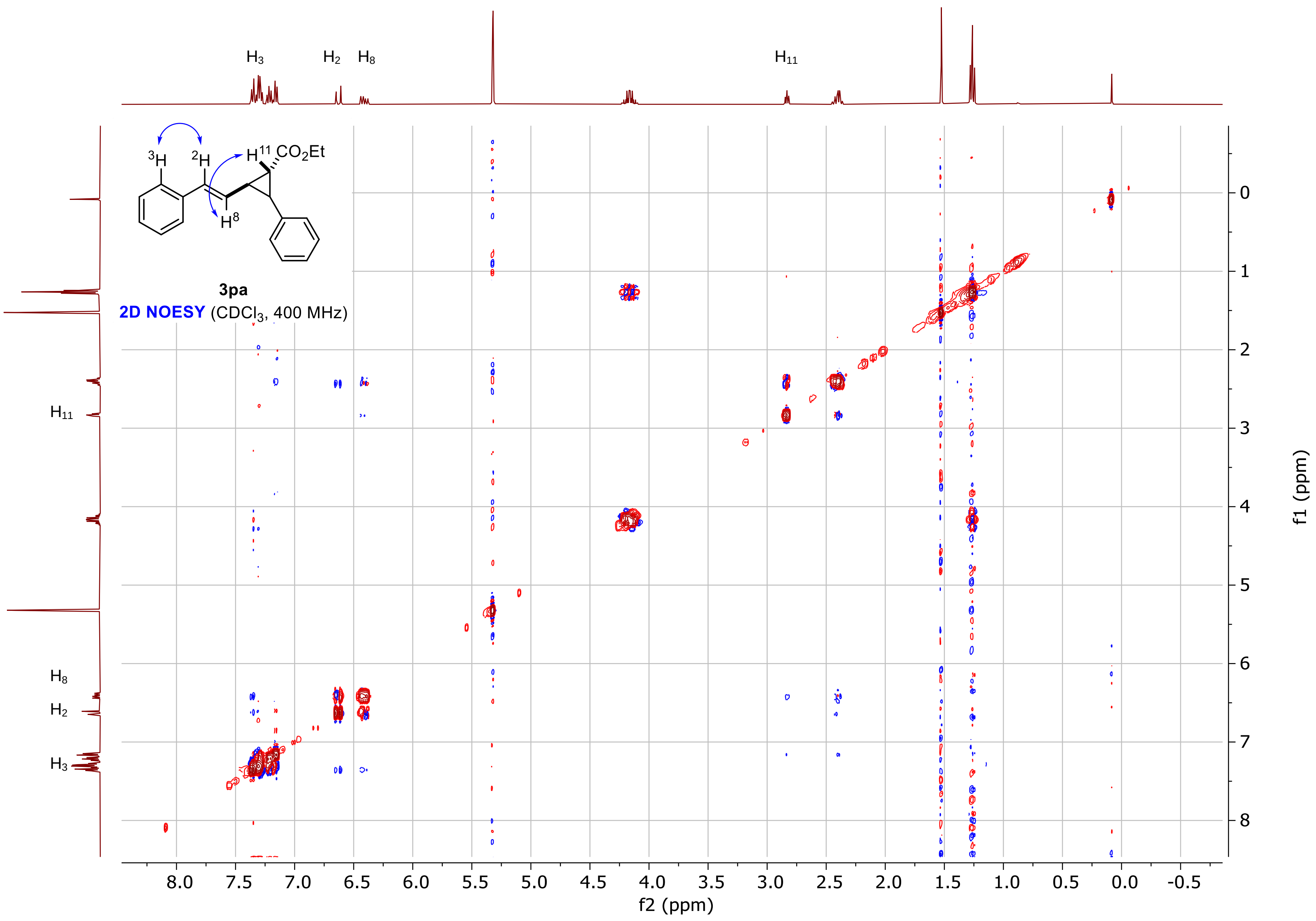
H3
H2
H8
H11
3H
2H
H11 CO2Et
H8
3pa
2D NOESY (CDCl3, 400 MHz)
f2 (ppm)
f1 (ppm)

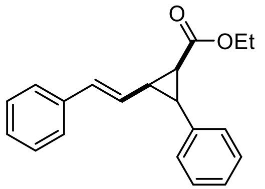

3pa'

^{1}H NMR ($CDCl_3$, 500 MHz)

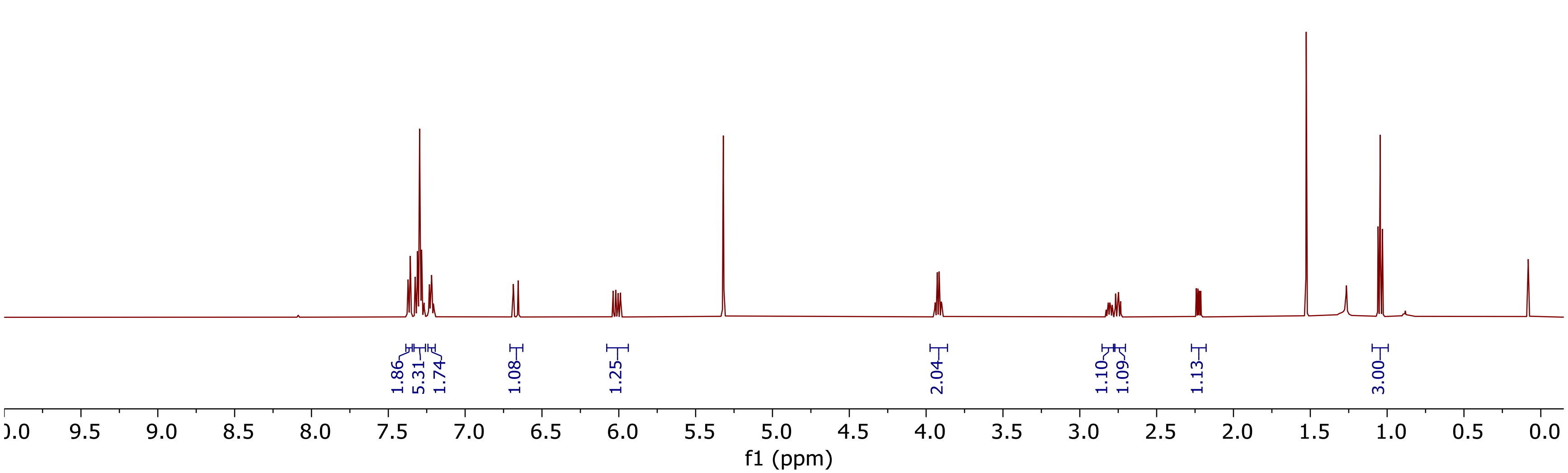

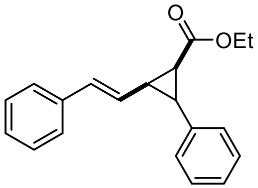

3pa'

$^{13}C\{^{1}H\}$ NMR ($CDCl_3$, 126 MHz)

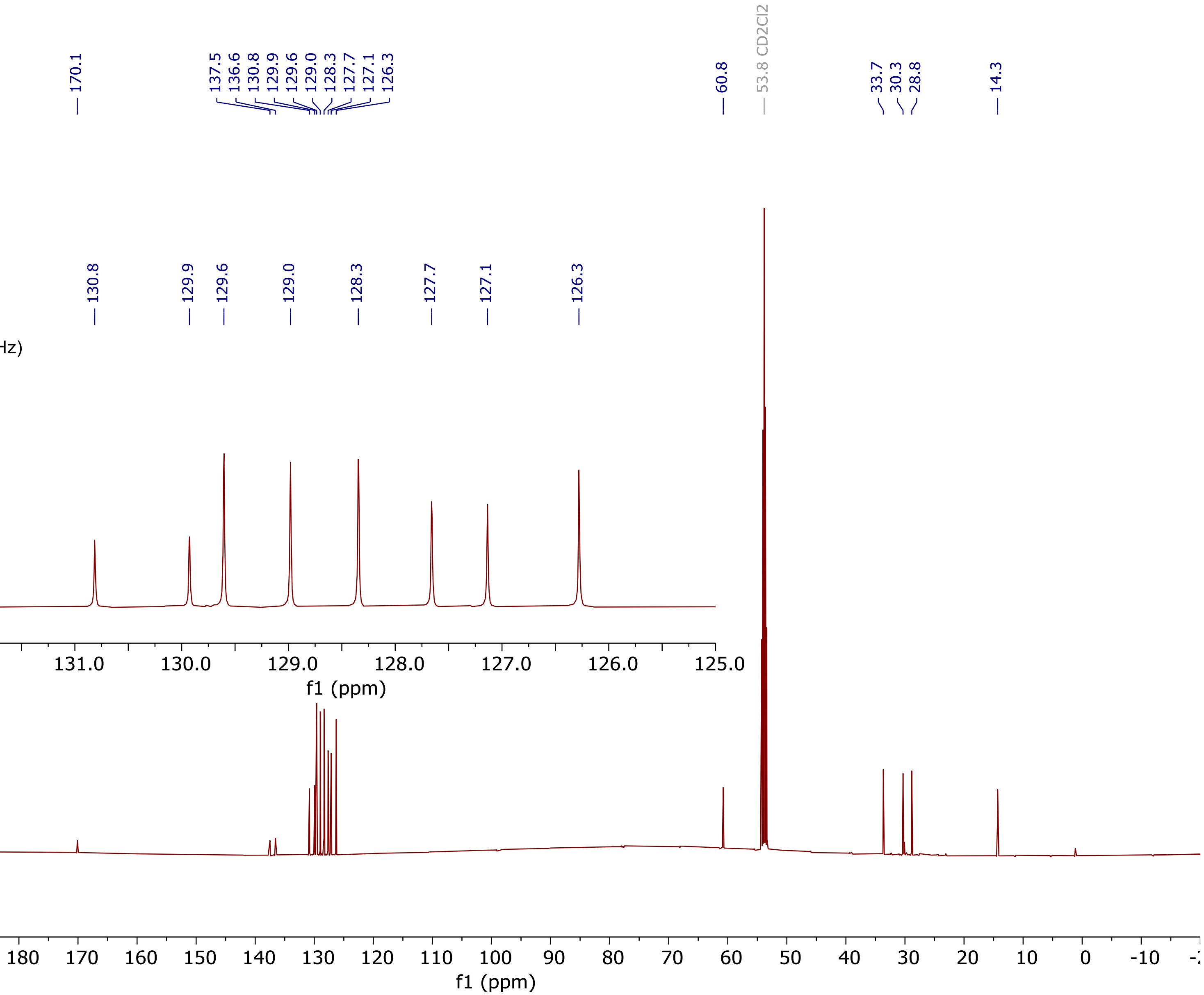

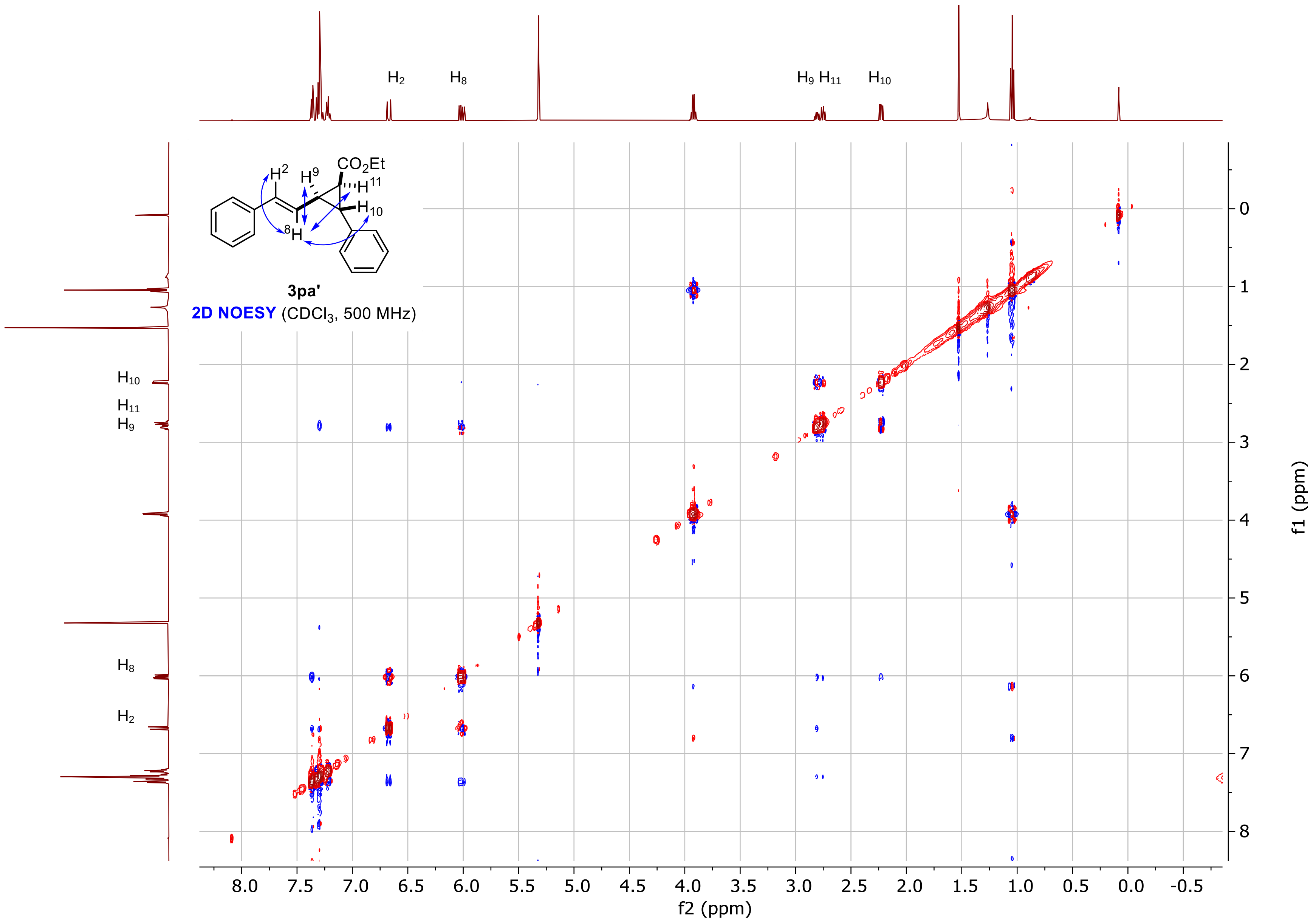
H2
H8
H9 H11
H10
CO2Et
H2
H9
H11
H10
8H
3pa'
2D NOESY (CDCl3, 500 MHz)
H10
H11
H9
H8
H2
f2 (ppm)
f1 (ppm)

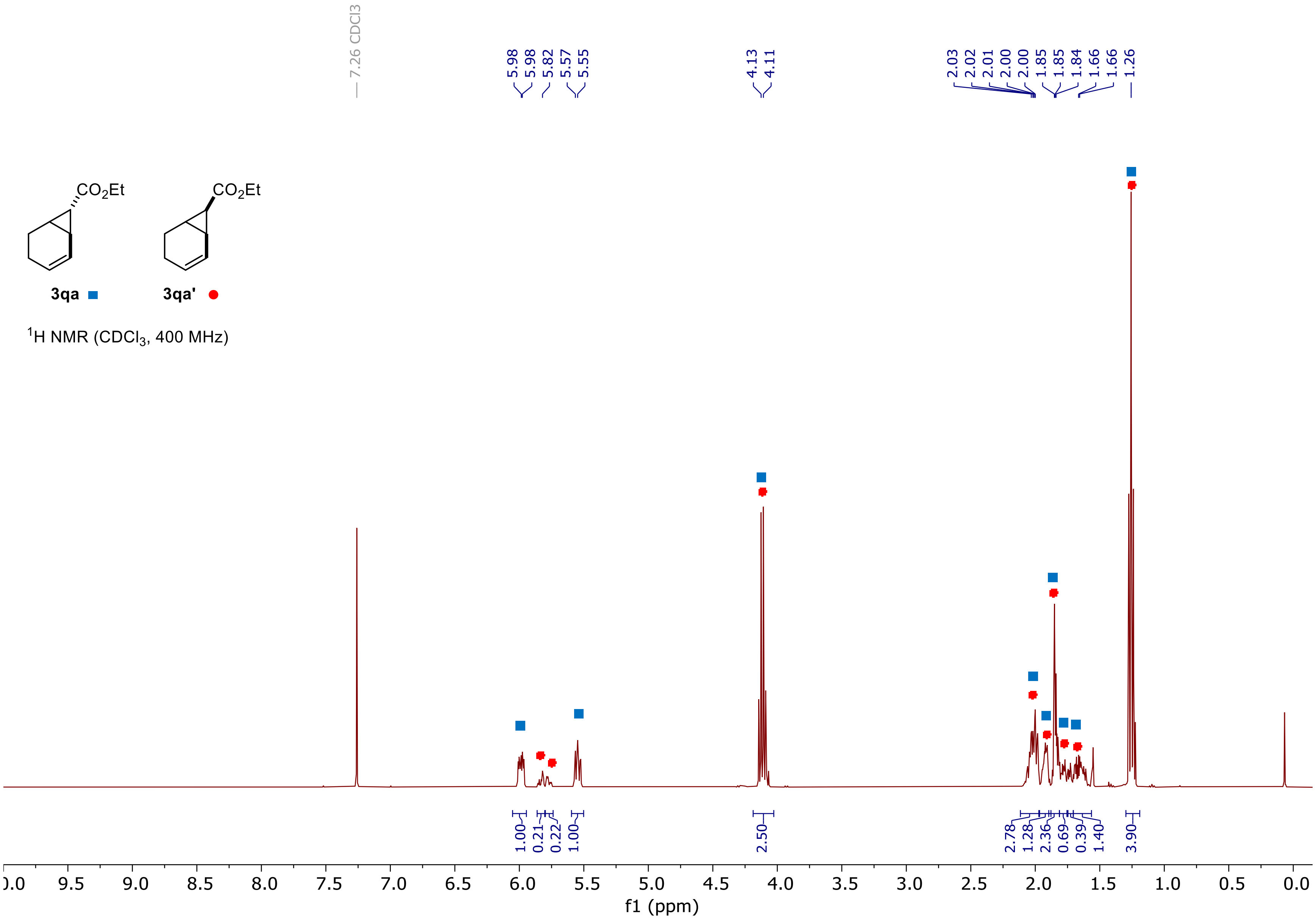

7.26 CDCl3
5.98
5.98
5.82
5.57
5.55
4.13
4.11
2.03
2.02
2.01
2.00
2.00
1.85
1.85
1.84
1.66
1.66
1.26
CO2Et
CO2Et
3qa
3qa'
1H NMR (CDCl3, 400 MHz)
1.00
0.21
0.22
1.00
2.50
2.78
1.28
2.36
0.69
0.39
1.40
3.90
f1 (ppm)

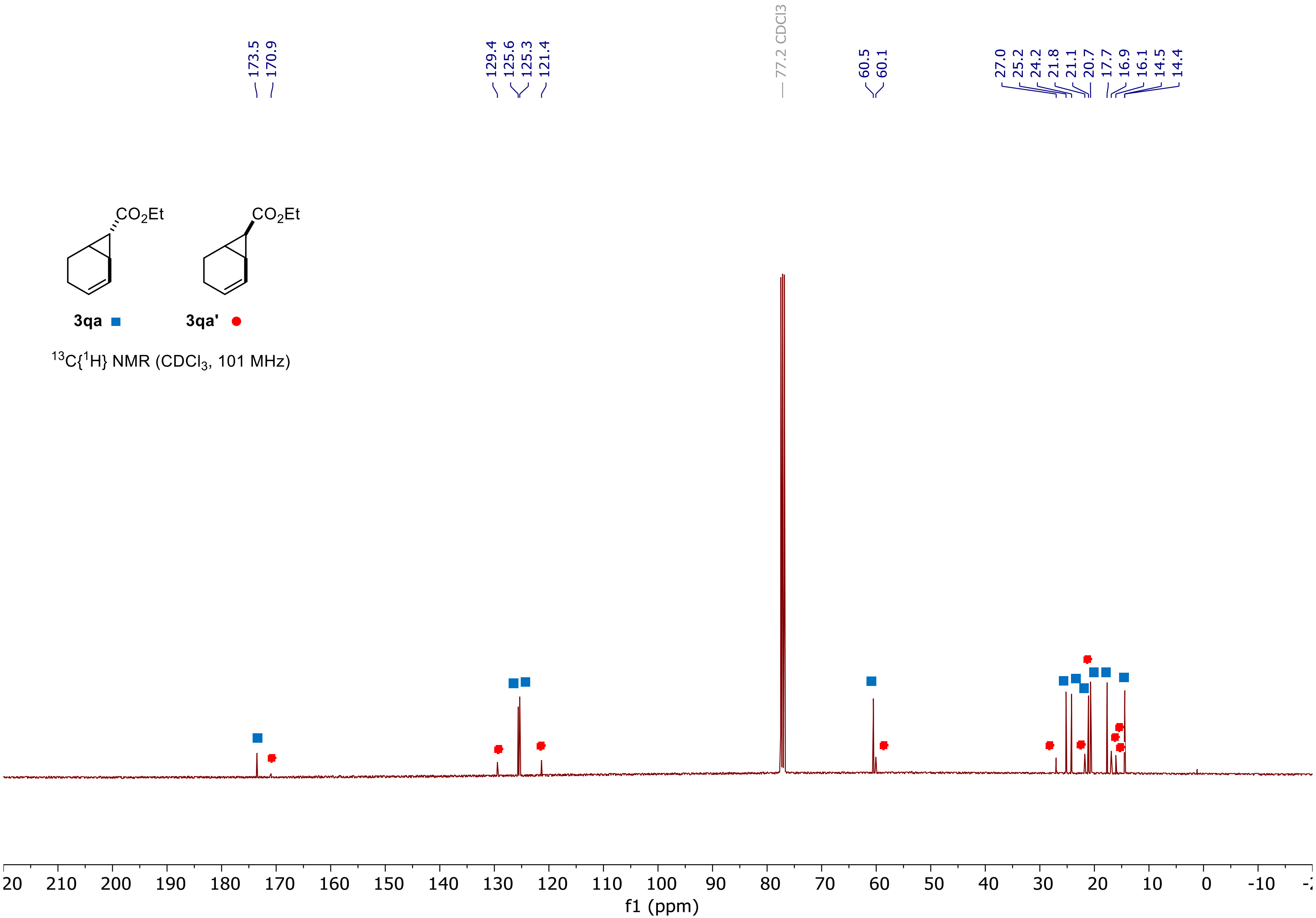

CO2Et
CO2Et
3qa
3qa'
13C{1H} NMR (CDCl3, 101 MHz)
173.5
170.9
129.4
125.6
125.3
121.4
77.2 CDCl3
60.5
60.1
27.0
25.2
24.2
21.8
21.1
20.7
17.7
16.9
16.1
14.5
14.4
f1 (ppm)

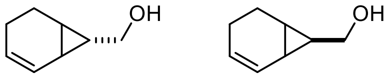

11qa-major **11qa'-minor**

^{1}H NMR ($CDCl_3$, 400 MHz)

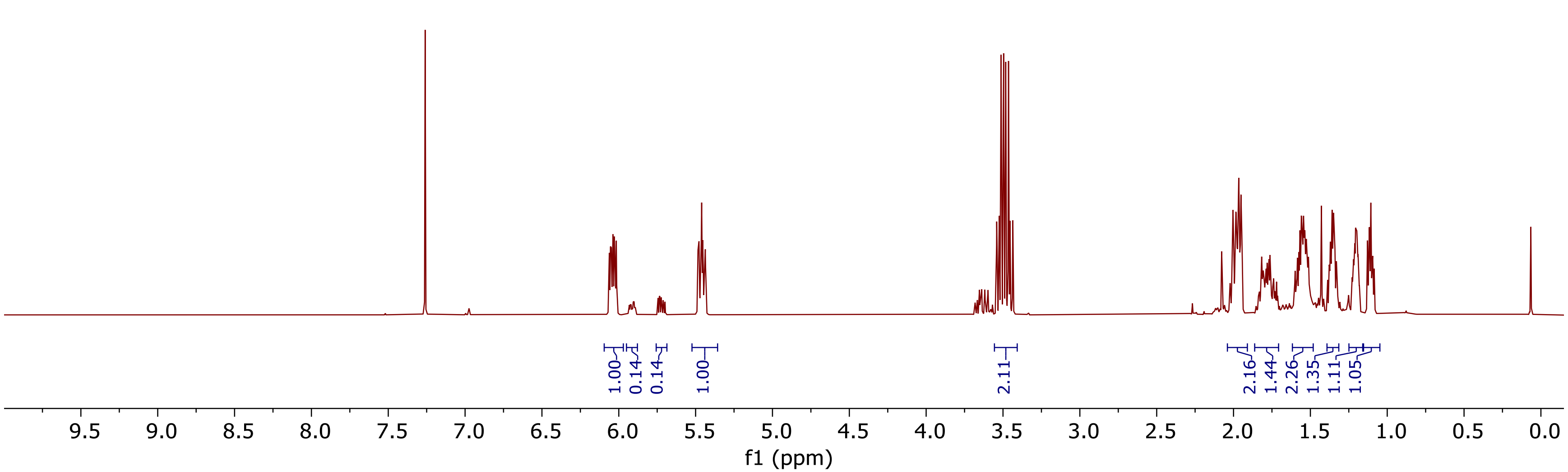

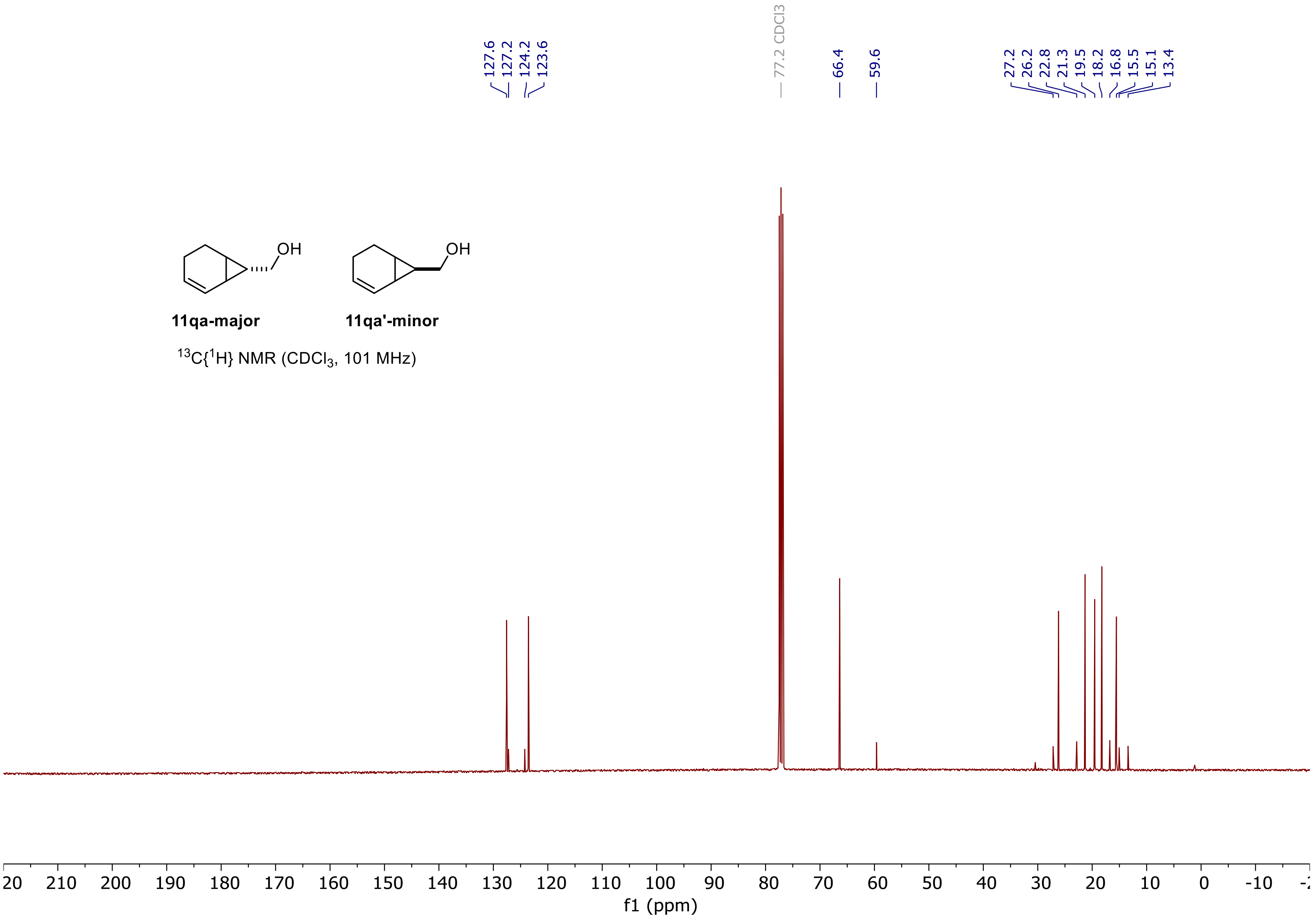

127.6
127.2
124.2
123.6
77.2 CDCl3
66.4
59.6
27.2
26.2
22.8
21.3
19.5
18.2
16.8
15.5
15.1
13.4
OH
OH
11qa-major
11qa'-minor
$^{13}C\{^1H\}$ NMR ($CDCl_3$, 101 MHz)
f1 (ppm)

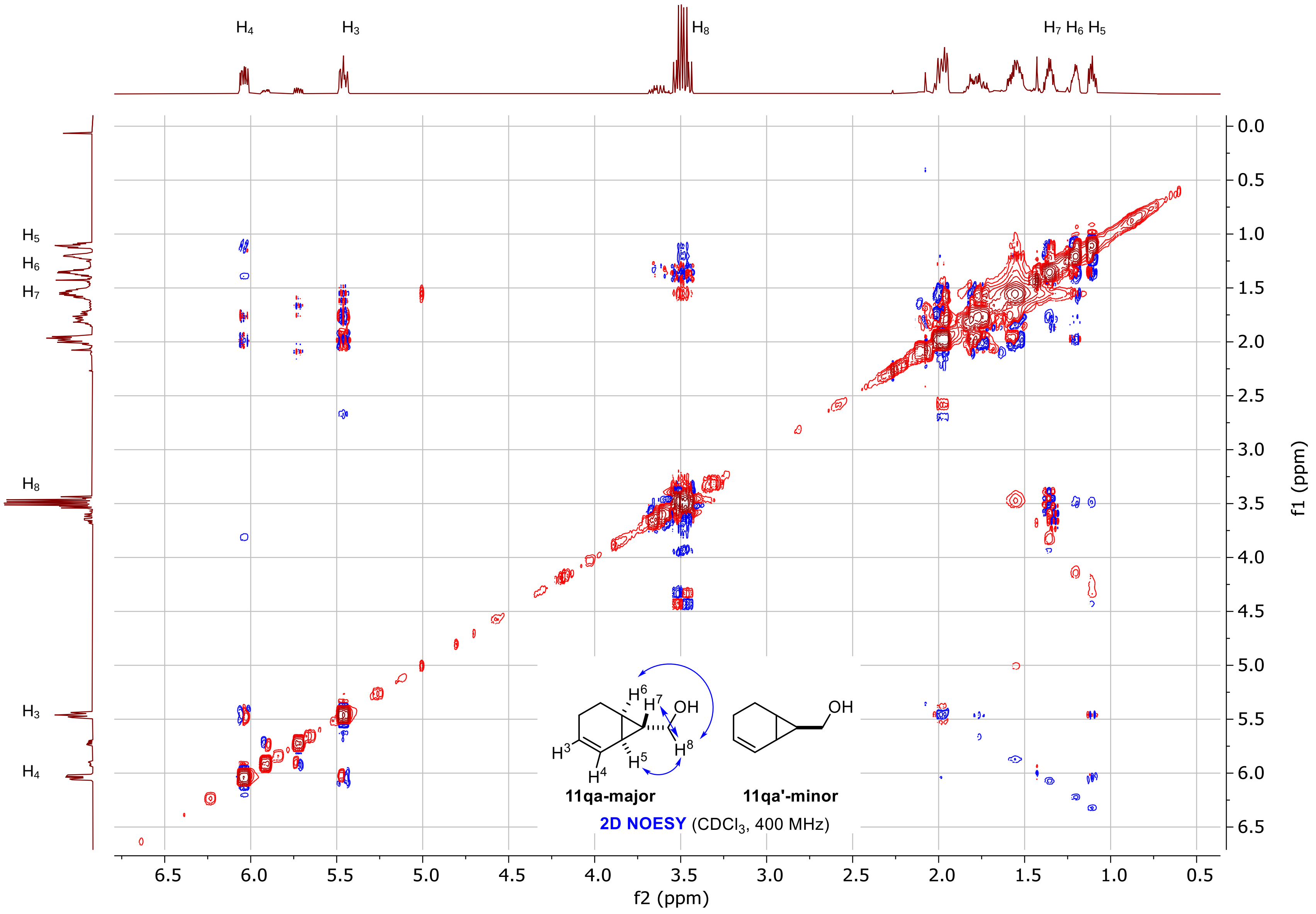
H4
H3
H8
H7 H6 H5
H5
H6
H7
H8
H3
H4
f1 (ppm)
f2 (ppm)
H6
H7
OH
H3
H8
H4
H5
OH
11qa-major
11qa'-minor
2D NOESY (CDCl3, 400 MHz)

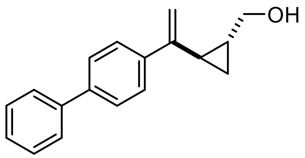

trans-**5aa**
^1H NMR ($CDCl_3$, 400 MHz)

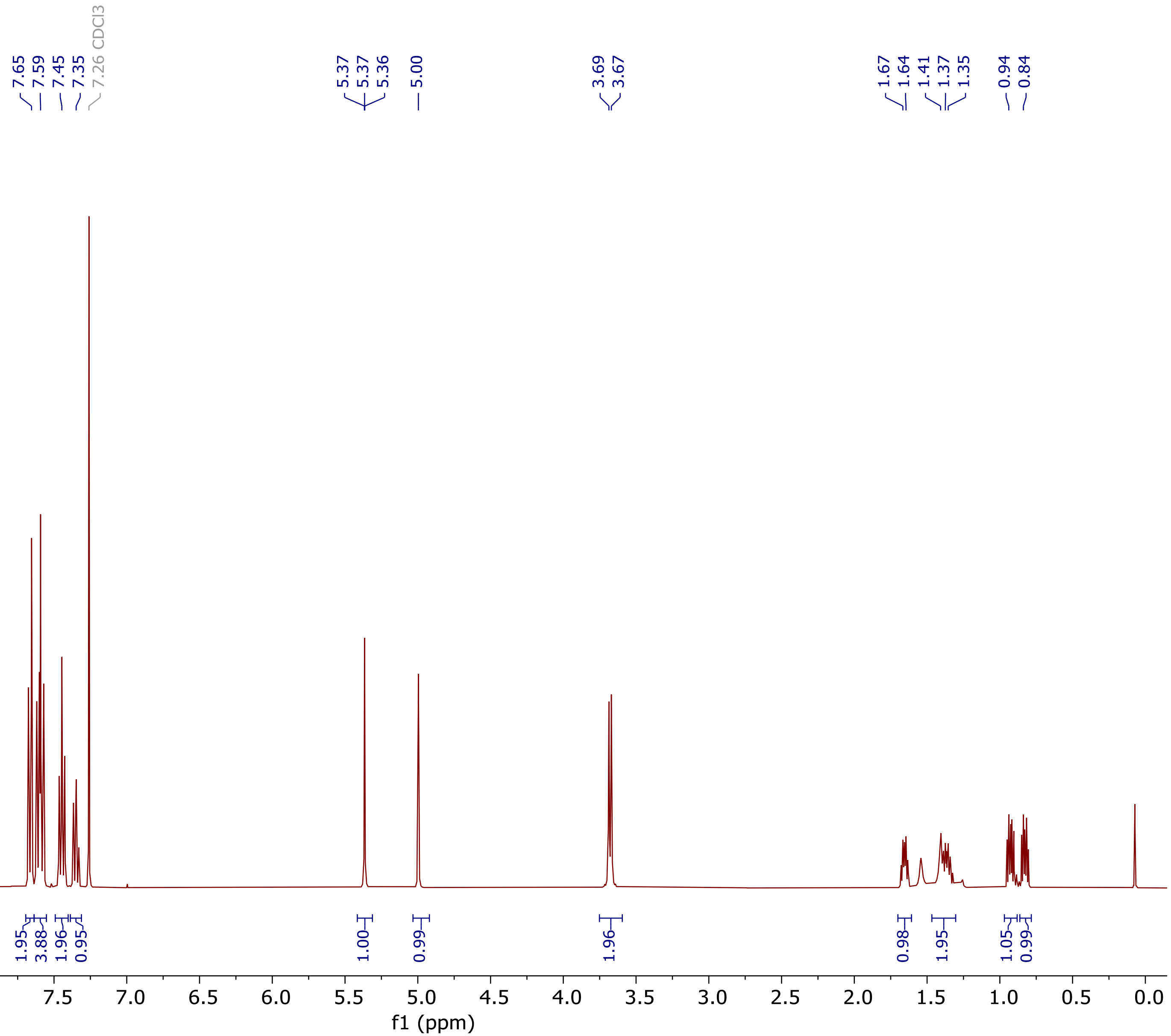

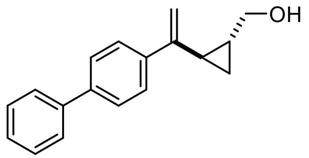

trans-**5aa**

$^{13}C\{^{1}H\}$ NMR ($CDCl_3$, 101 MHz)

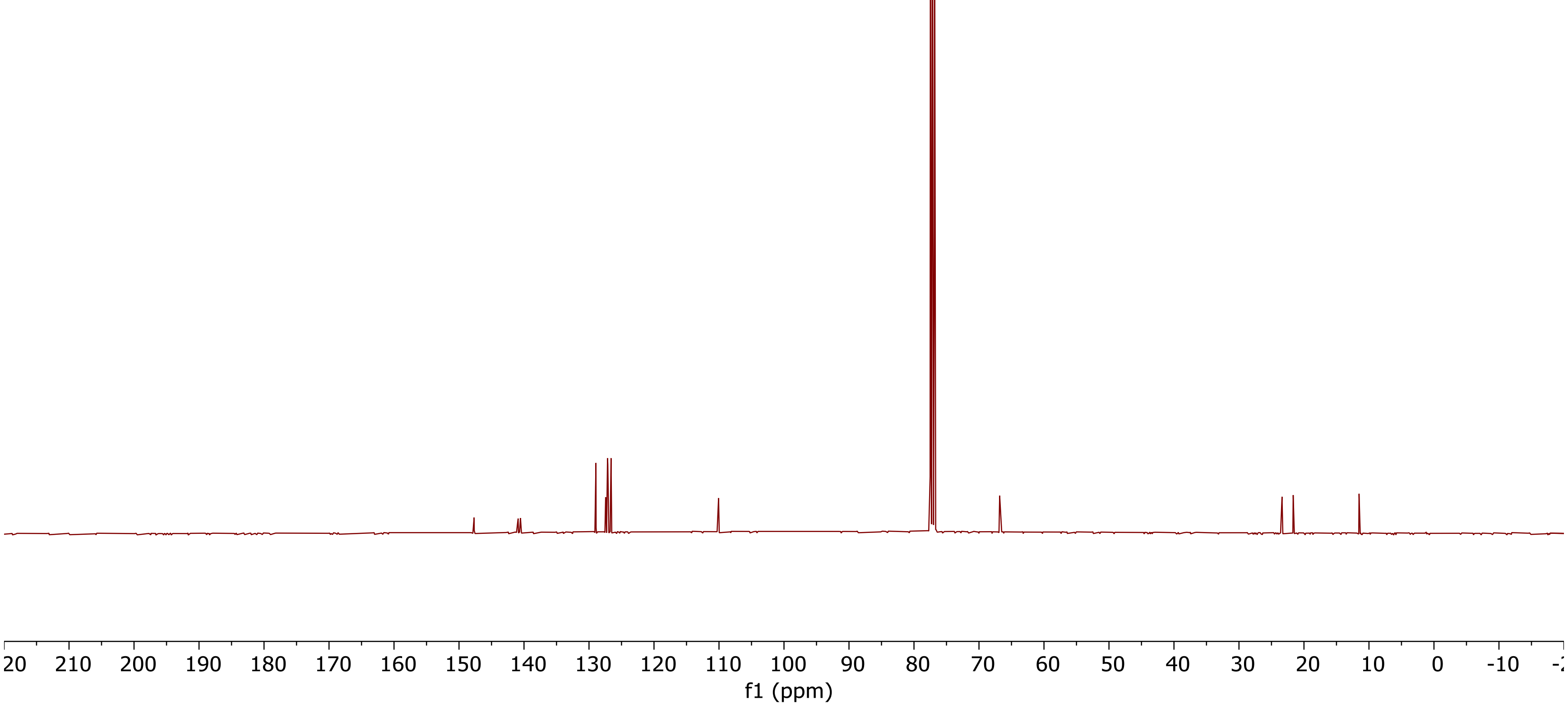

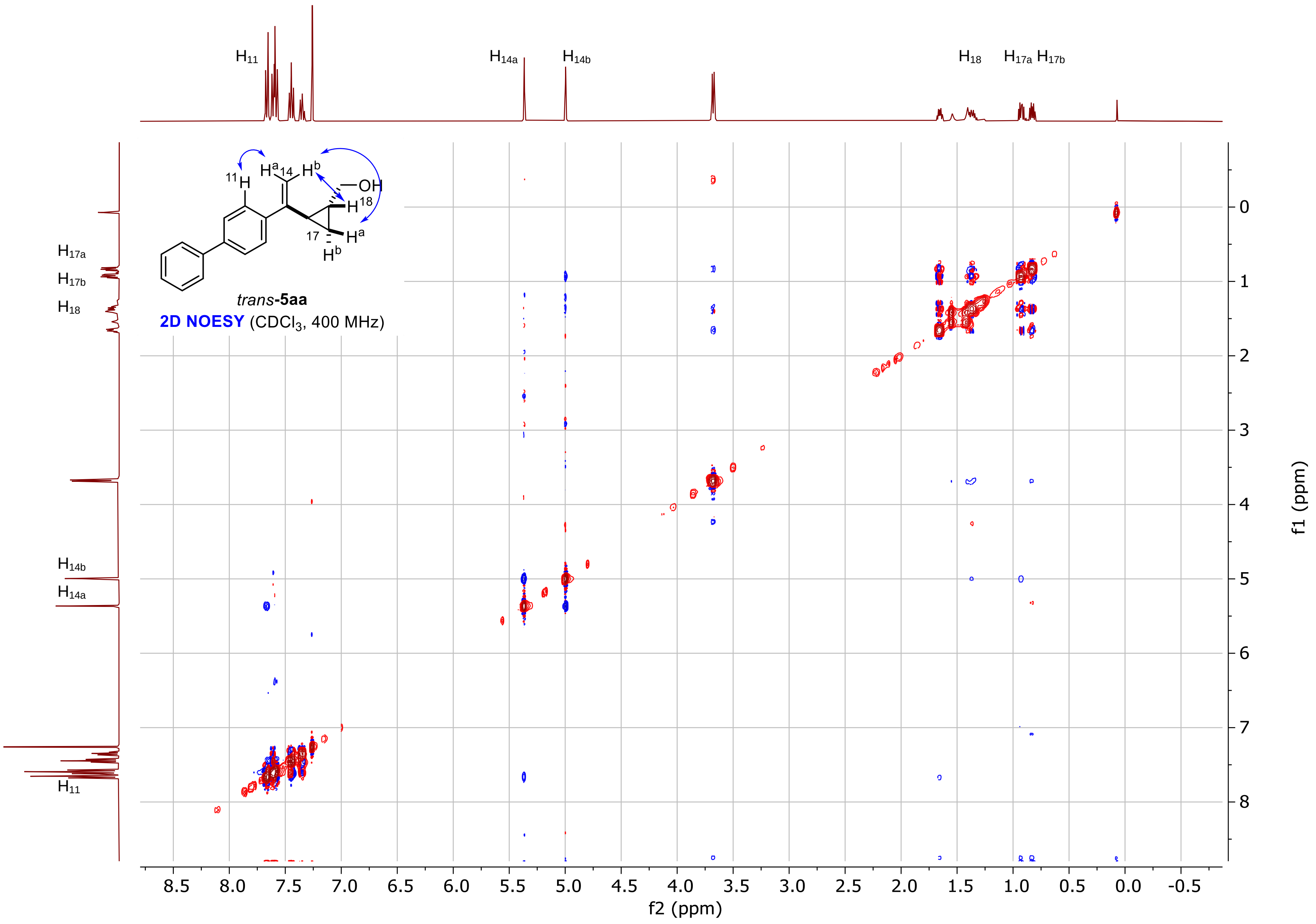
H11
H14a
H14b
H18
H17a
H17b
OH
trans-5aa
2D NOESY (CDCl3, 400 MHz)
f2 (ppm)
f1 (ppm)

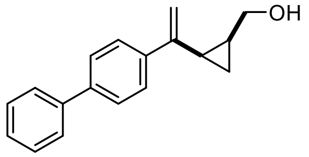

cis-**5aa**

^{1}H NMR ($CDCl_3$, 400 MHz)

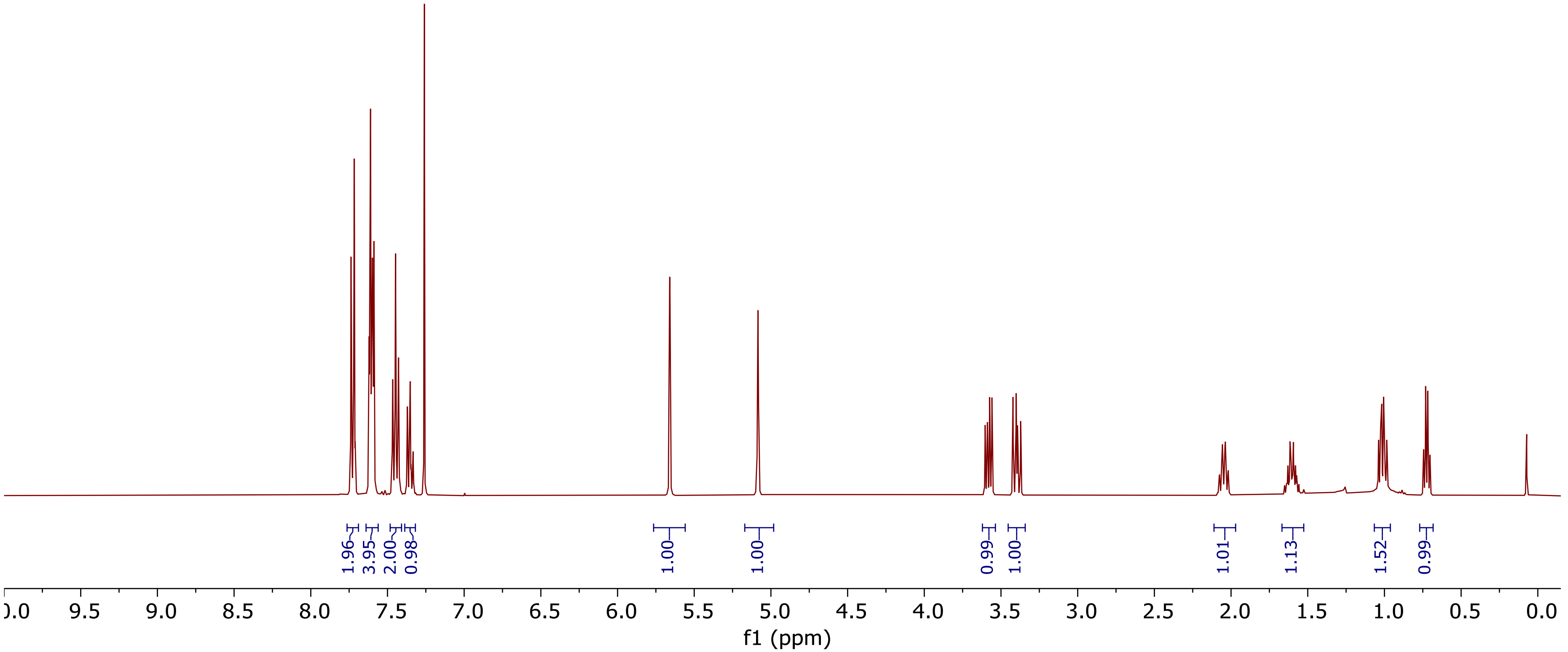

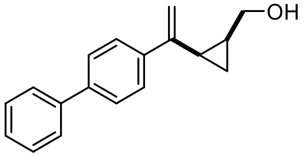

cis-**5aa**

$^{13}C\{^1H\}$ NMR ($CDCl_3$, 101 MHz)

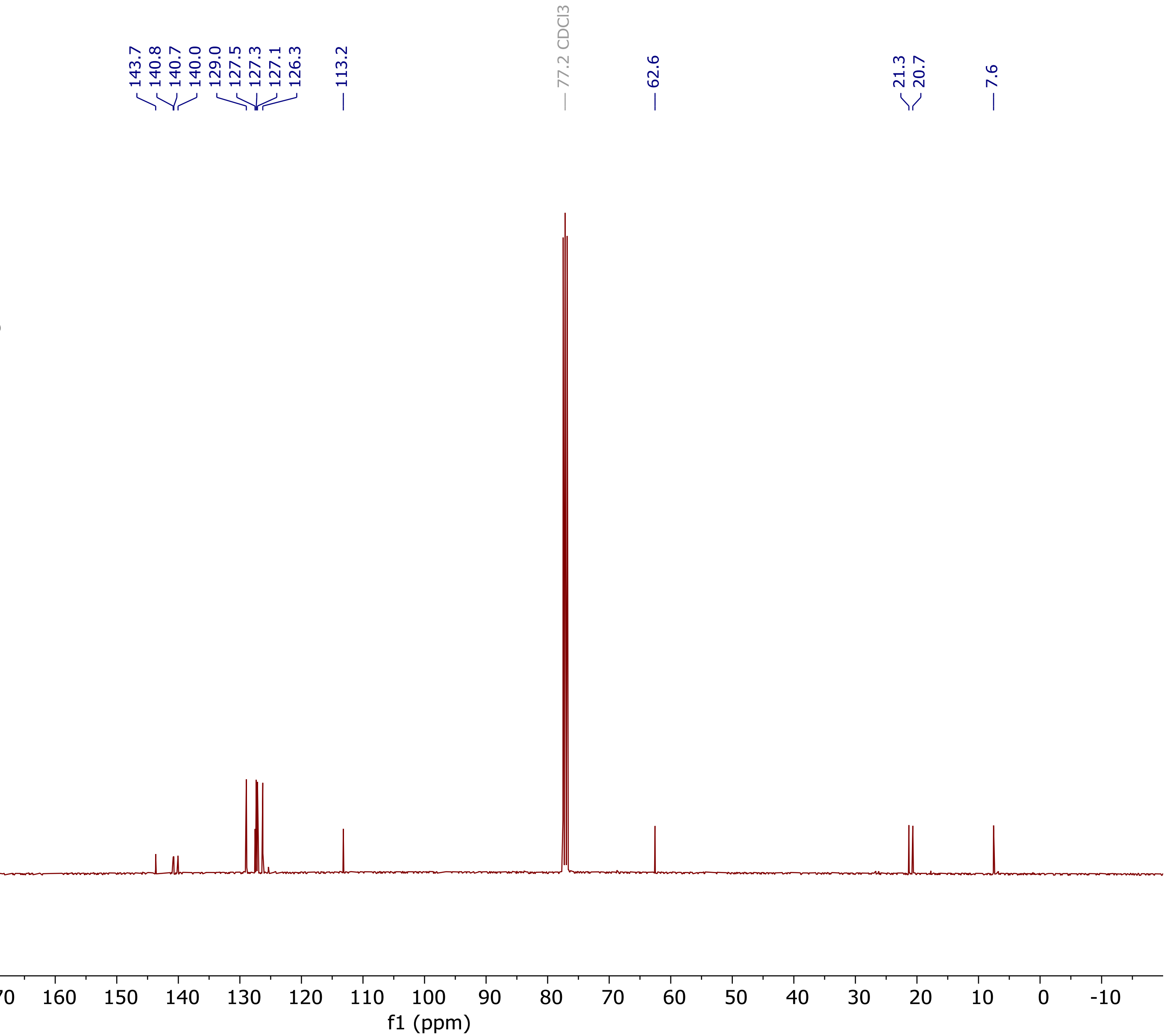

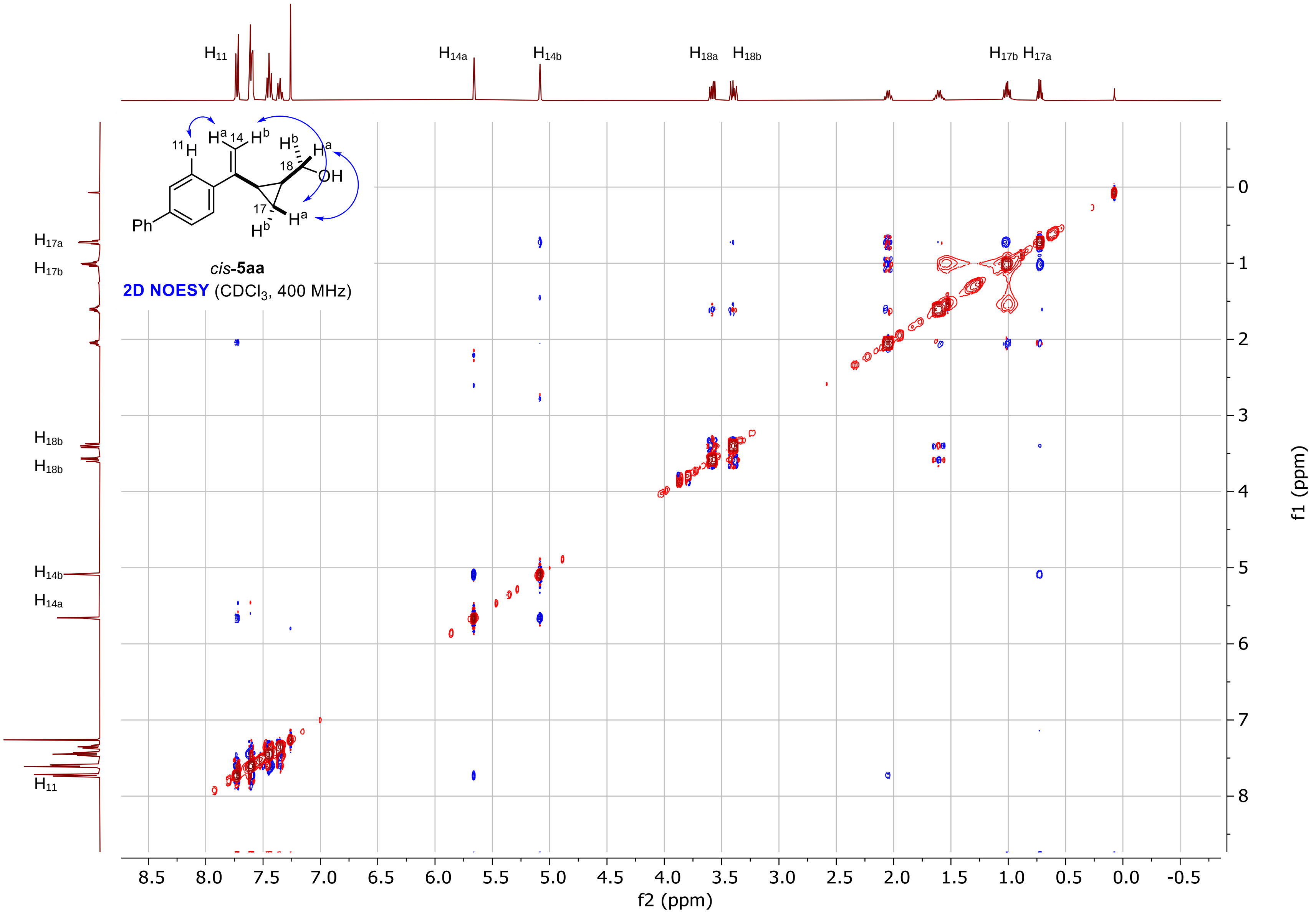

H11
H14a
H14b
H18a
H18b
H17b
H17a
H17a
H17b
H18b
H18b
H14b
H14a
H11
11H
Ha 14 Hb
Hb
Ha
18
OH
17
Hb
Ha
Ph
cis-5aa
2D NOESY (CDCl3, 400 MHz)
8.5
8.0
7.5
7.0
6.5
6.0
5.5
5.0
4.5
4.0
3.5
3.0
2.5
2.0
1.5
1.0
0.5
0.0
-0.5
f2 (ppm)
0
1
2
3
4
5
6
7
8
f1 (ppm)

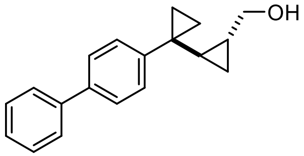

trans-**6aa**

^{1}H NMR ($CDCl_3$, 400 MHz)

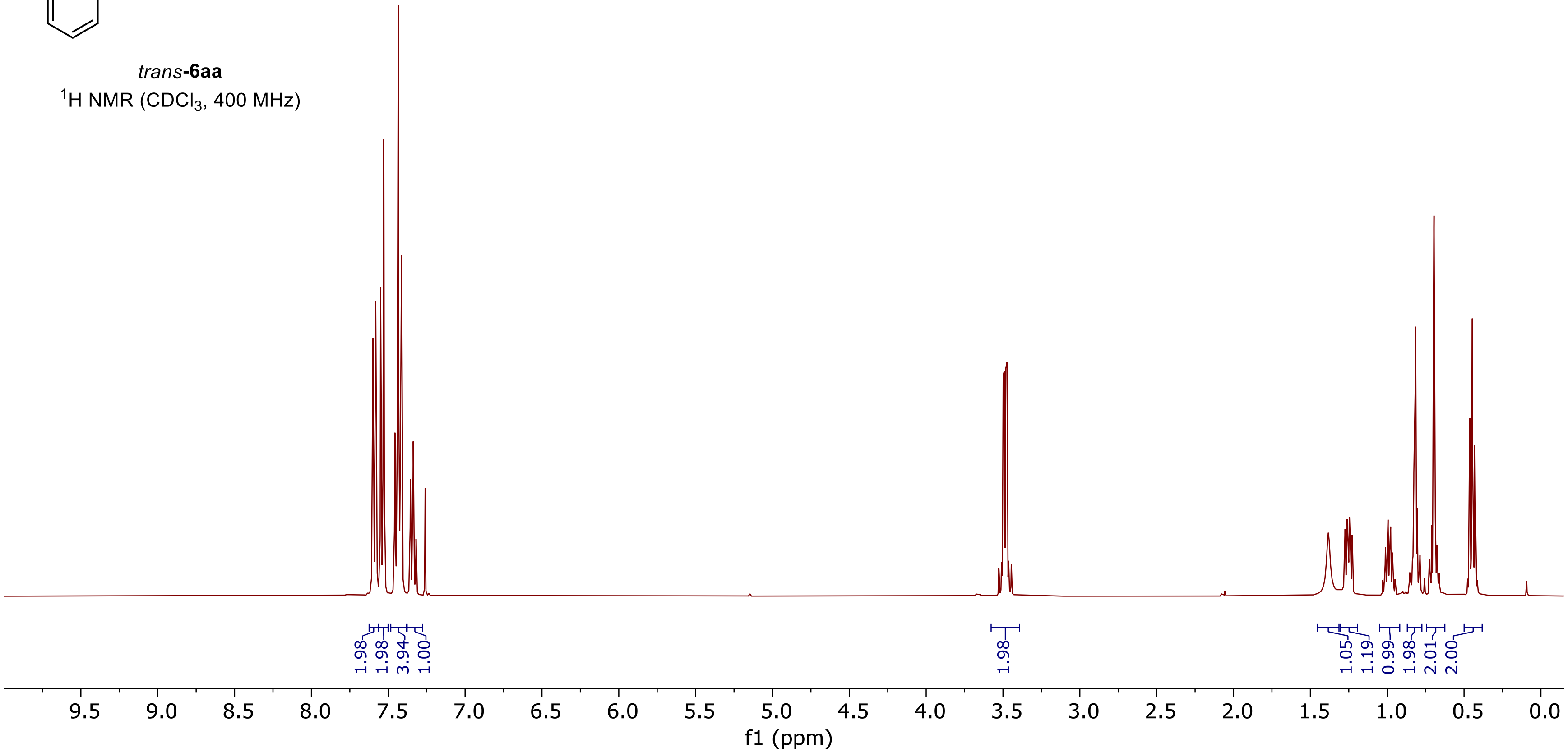

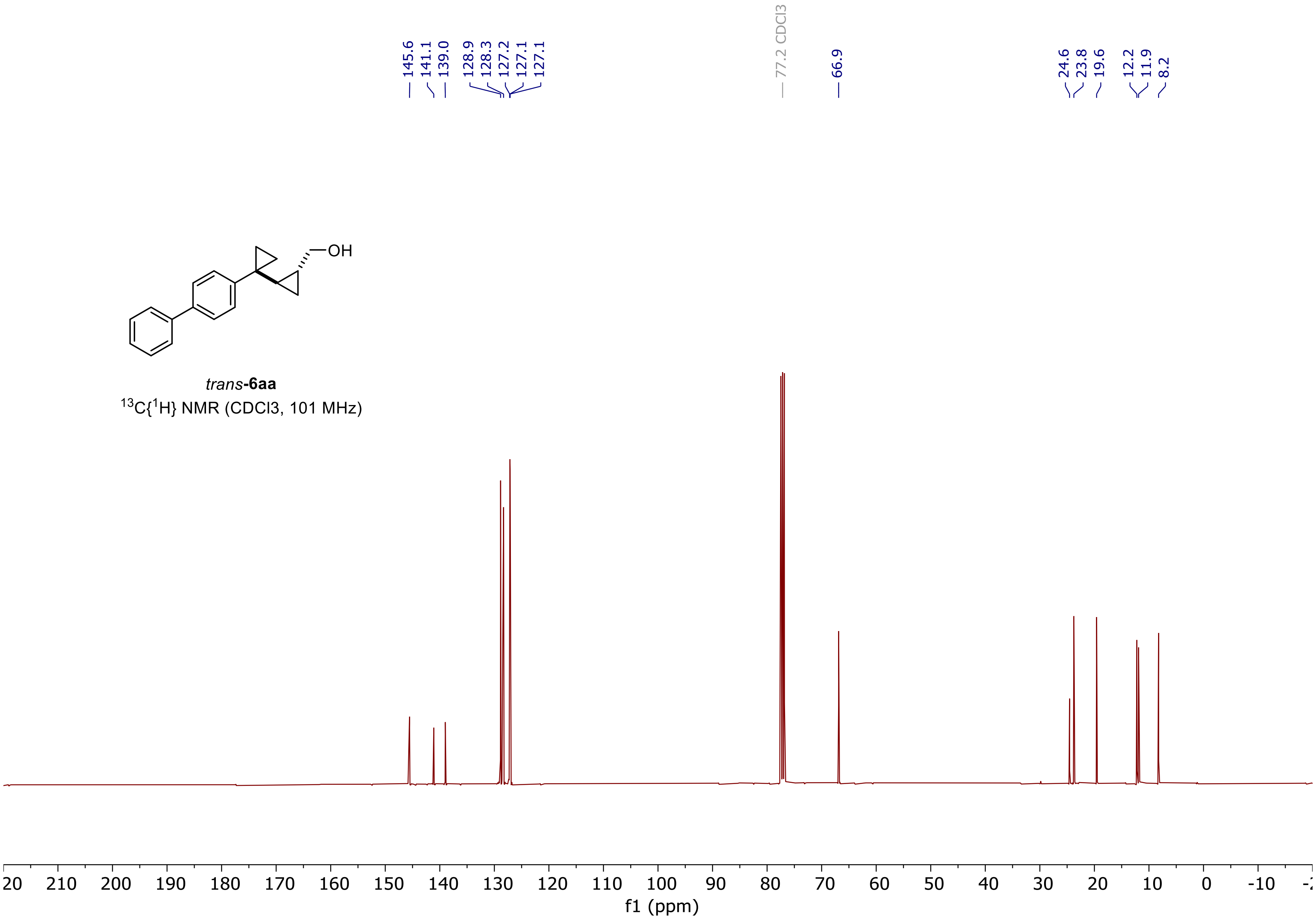

trans-**6aa**

$^{13}C\{^1H\}$ NMR (CDCl3, 101 MHz)

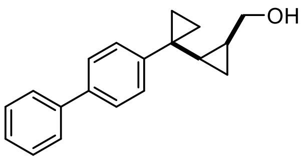

cis-**6aa**

^{1}H NMR ($CDCl_3$, 400 MHz)

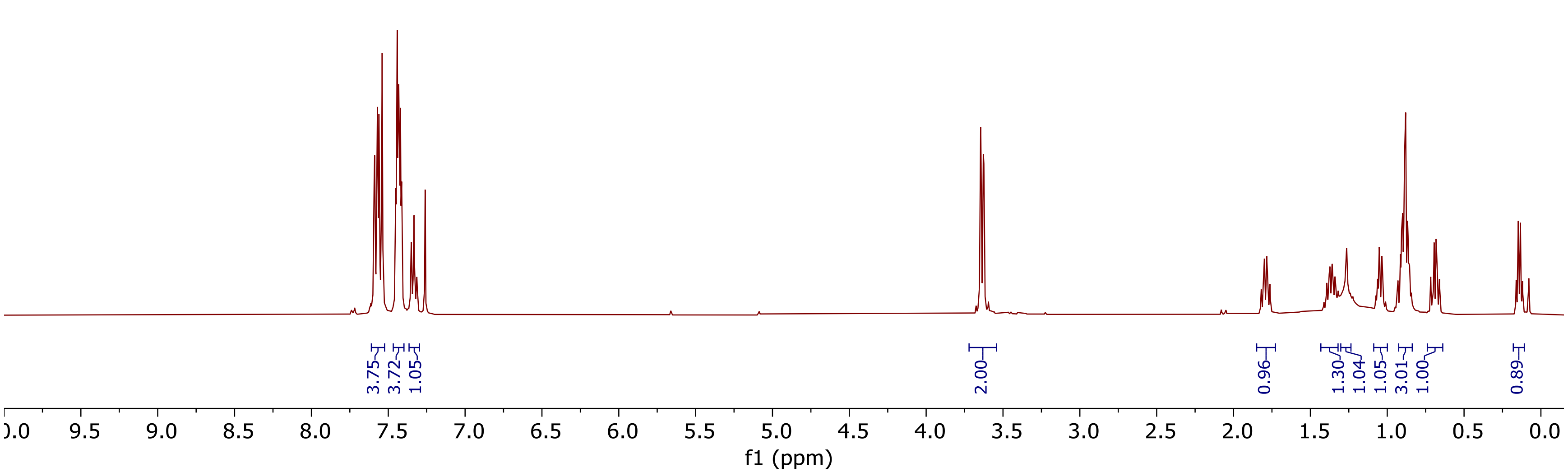

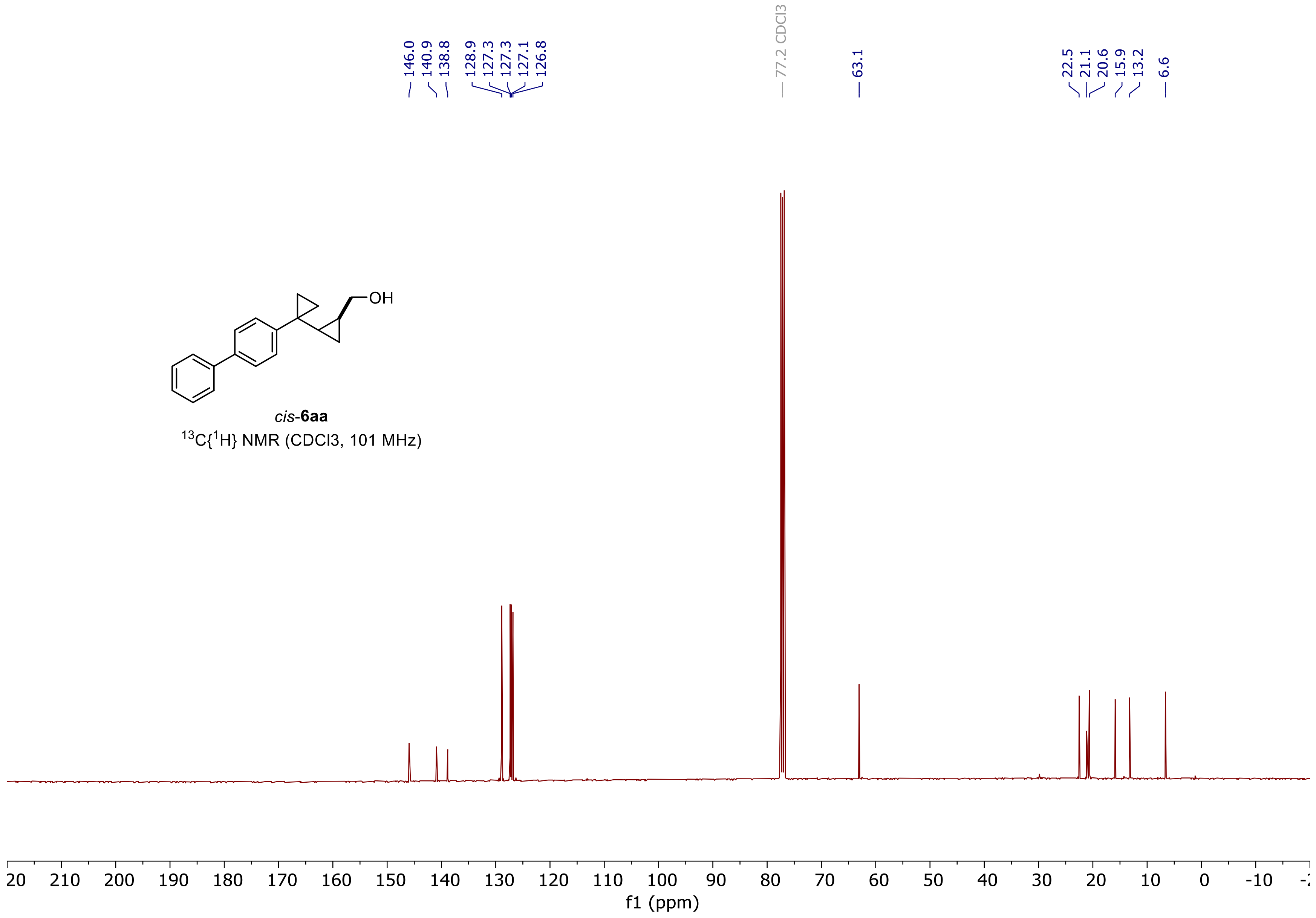
146.0
140.9
138.8
128.9
127.3
127.3
127.1
126.8
77.2 CDCl3
63.1
22.5
21.1
20.6
15.9
13.2
6.6
OH
cis-6aa
13C{1H} NMR (CDCl3, 101 MHz)
f1 (ppm)

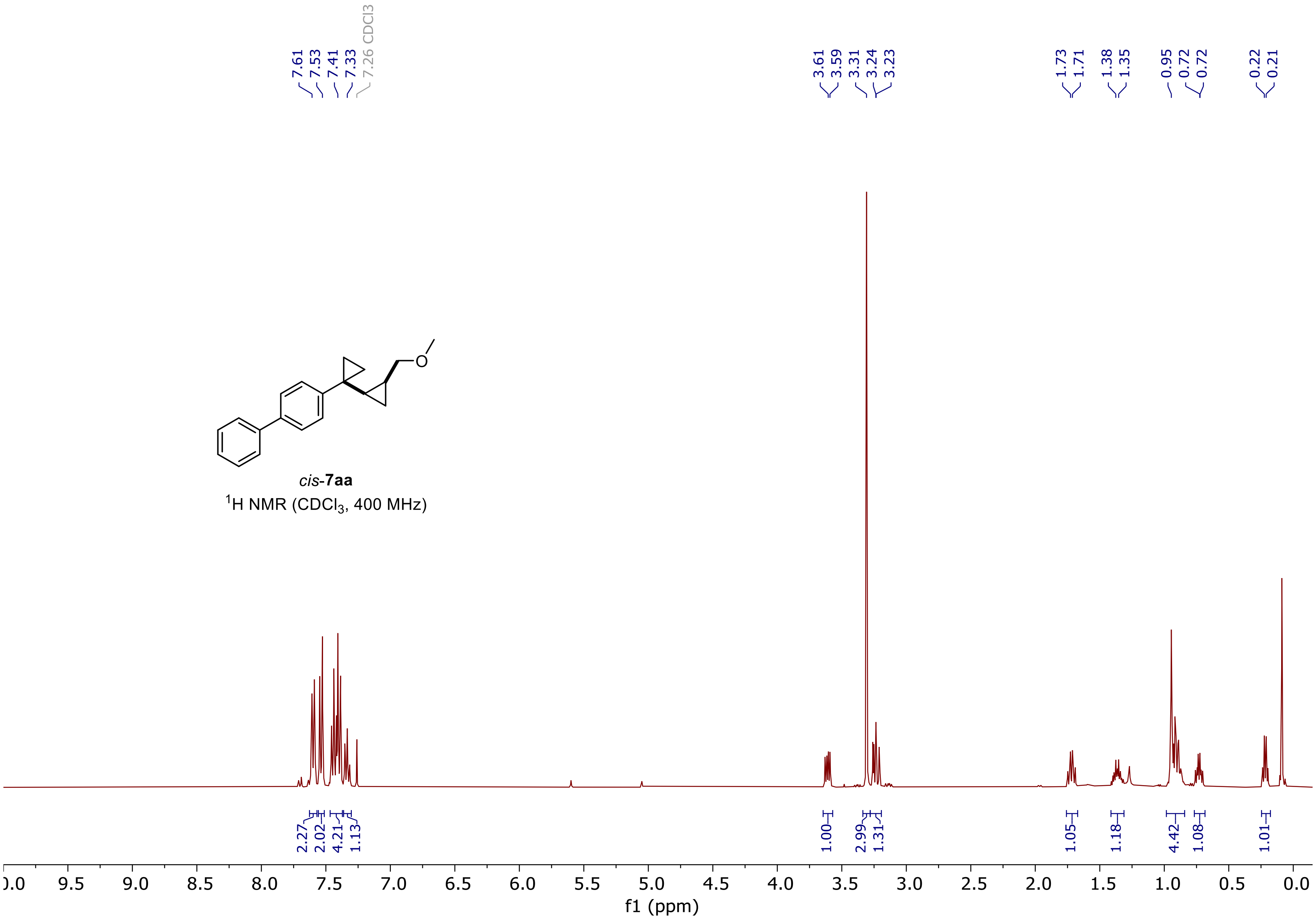

cis-**7aa**
^1H NMR ($CDCl_3$, 400 MHz)

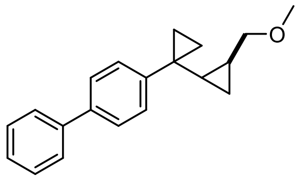

cis-**7aa**

$^{13}C\{^{1}H\}$ NMR (CDC$_{l3}$, 101 MHz)

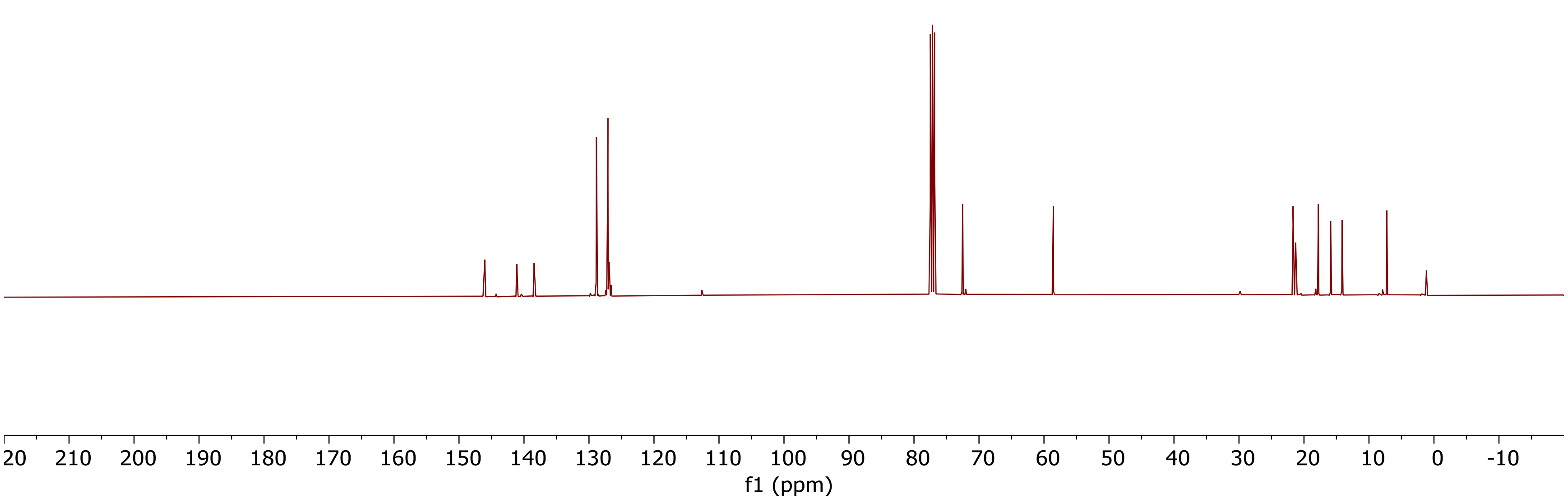

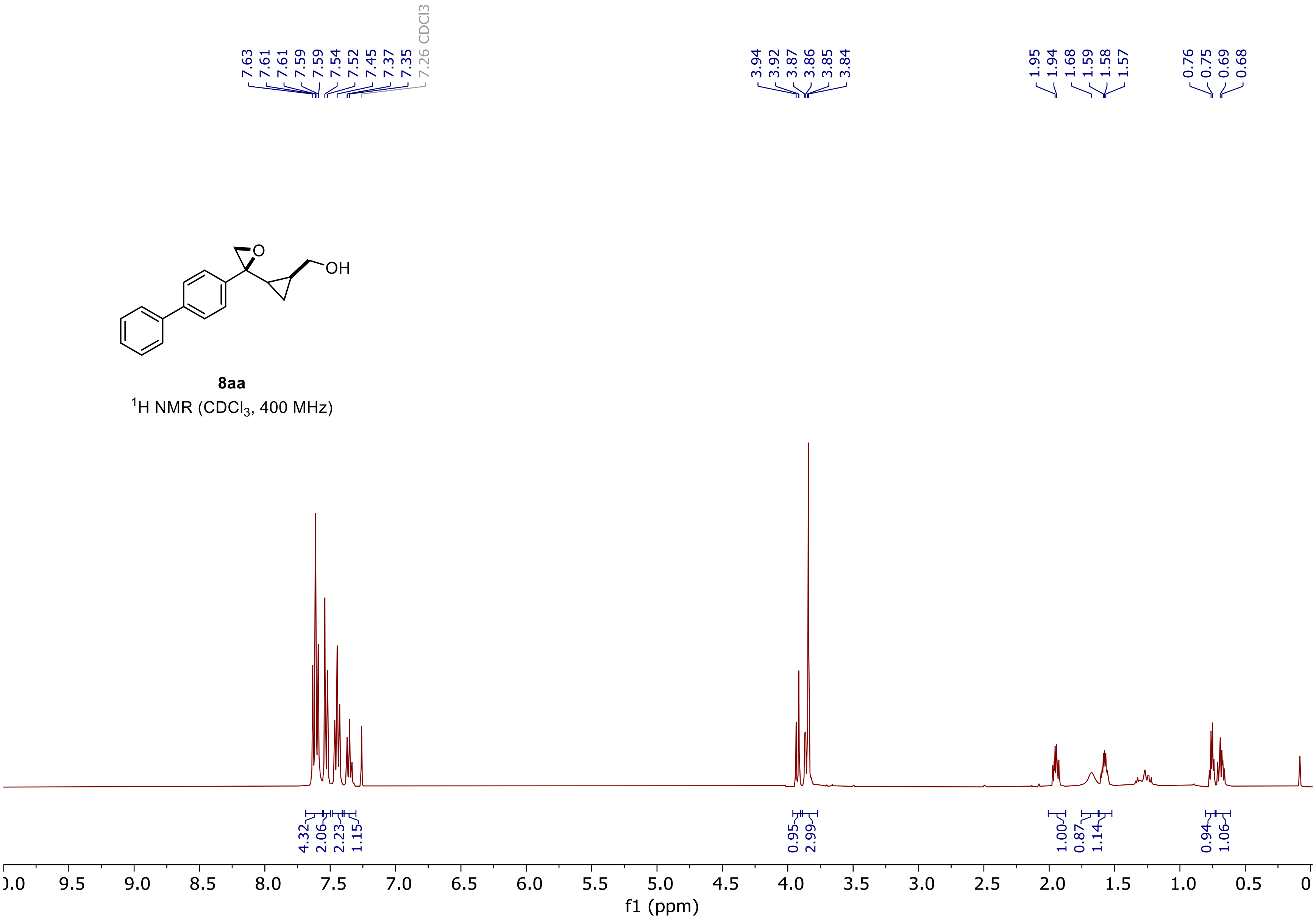

8aa

^{1}H NMR ($CDCl_3$, 400 MHz)

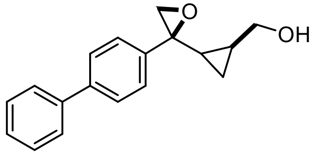

8aa

$^{13}C\{^1H\}$ NMR ($CDCl_3$, 101 MHz)

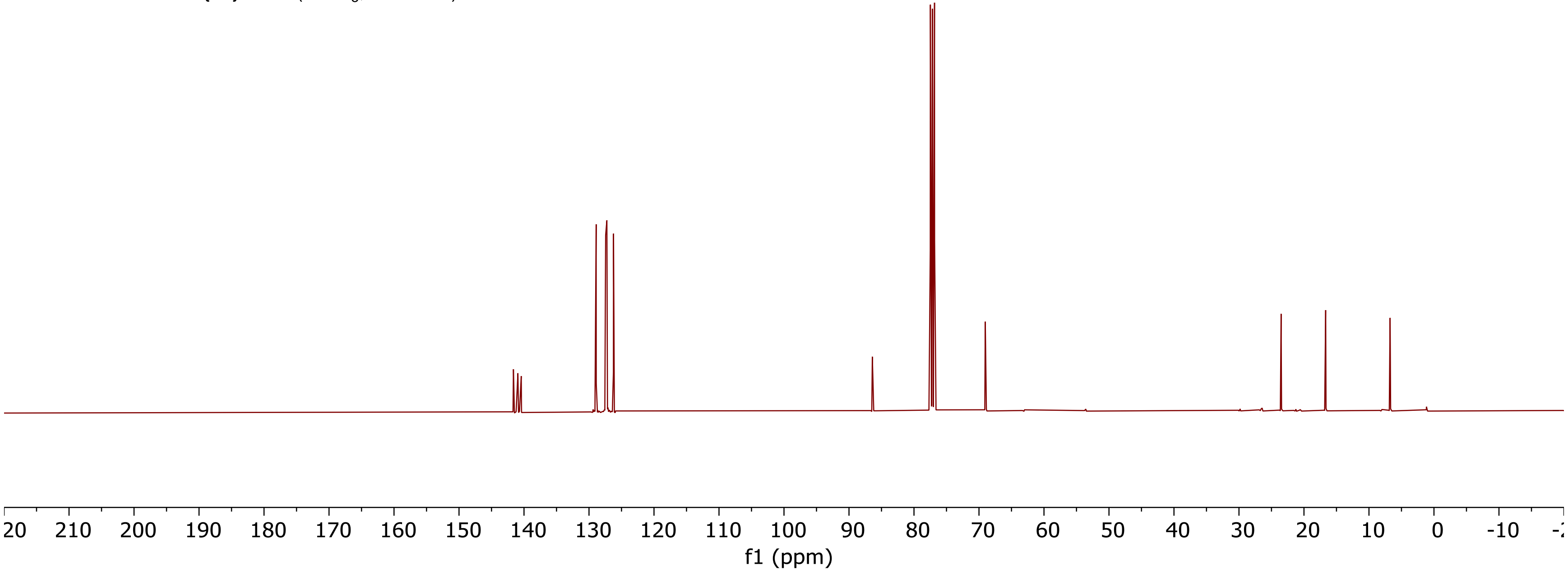

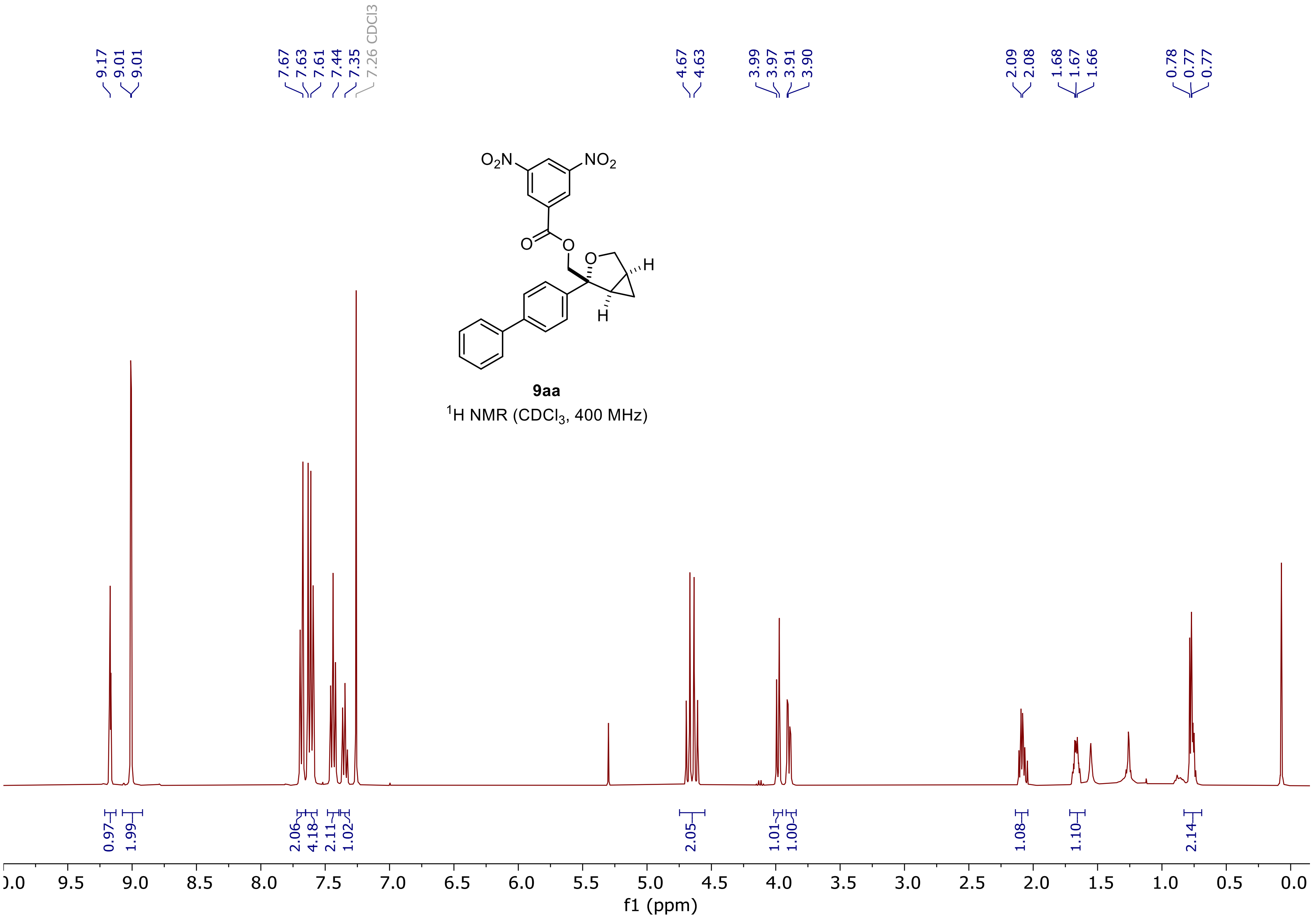
9.17
9.01
9.01
7.67
7.63
7.61
7.44
7.35
7.26 CDCl3
4.67
4.63
3.99
3.97
3.91
3.90
2.09
2.08
1.68
1.67
1.66
0.78
0.77
0.77
O2N
NO2
O
O
O
H
H
9aa
1H NMR (CDCl3, 400 MHz)
0.97
1.99
2.06
4.18
2.11
1.02
2.05
1.01
1.00
1.08
1.10
2.14
f1 (ppm)

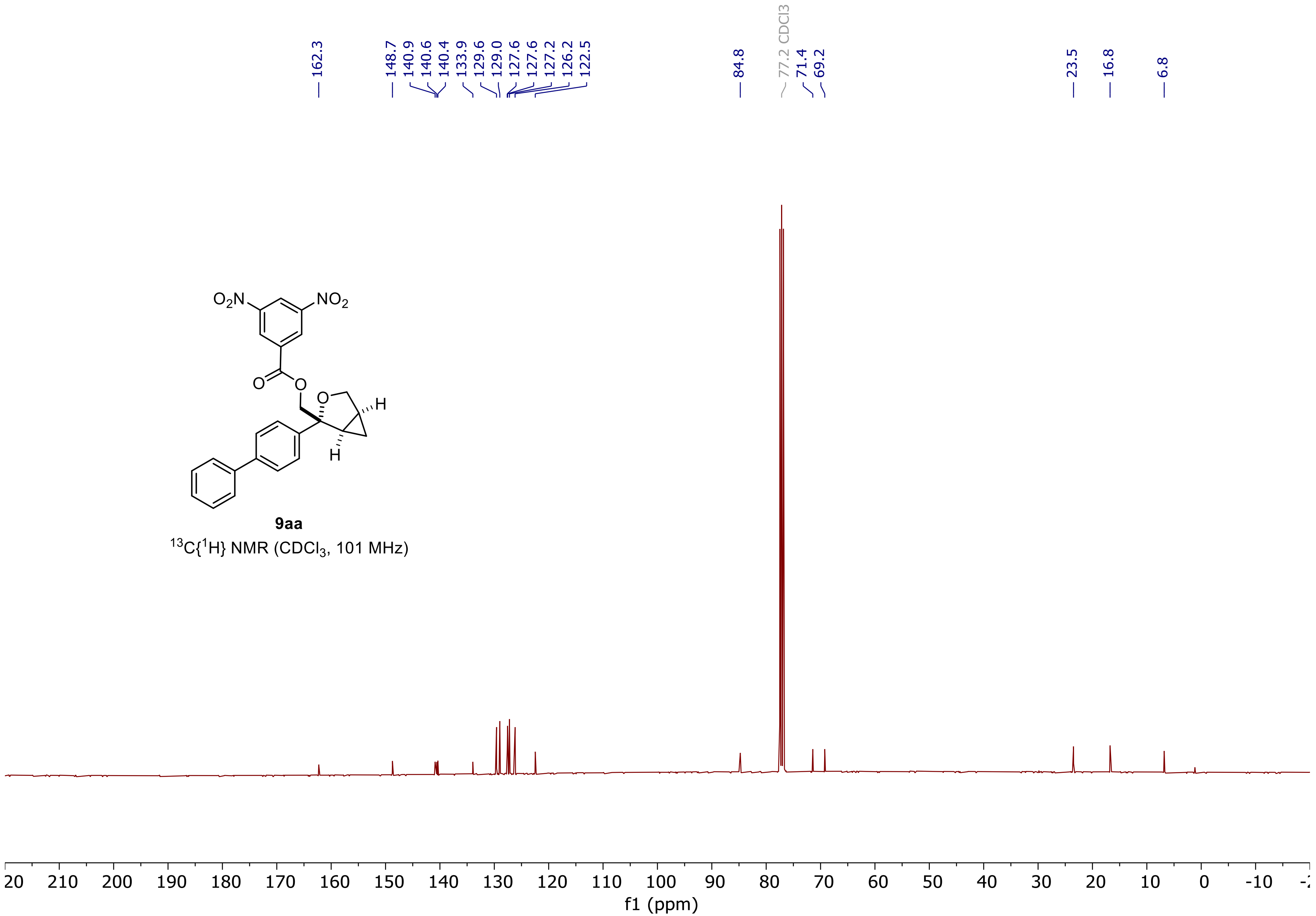

9aa

$^{13}C\{^{1}H\}$ NMR ($CDCl_3$, 101 MHz)

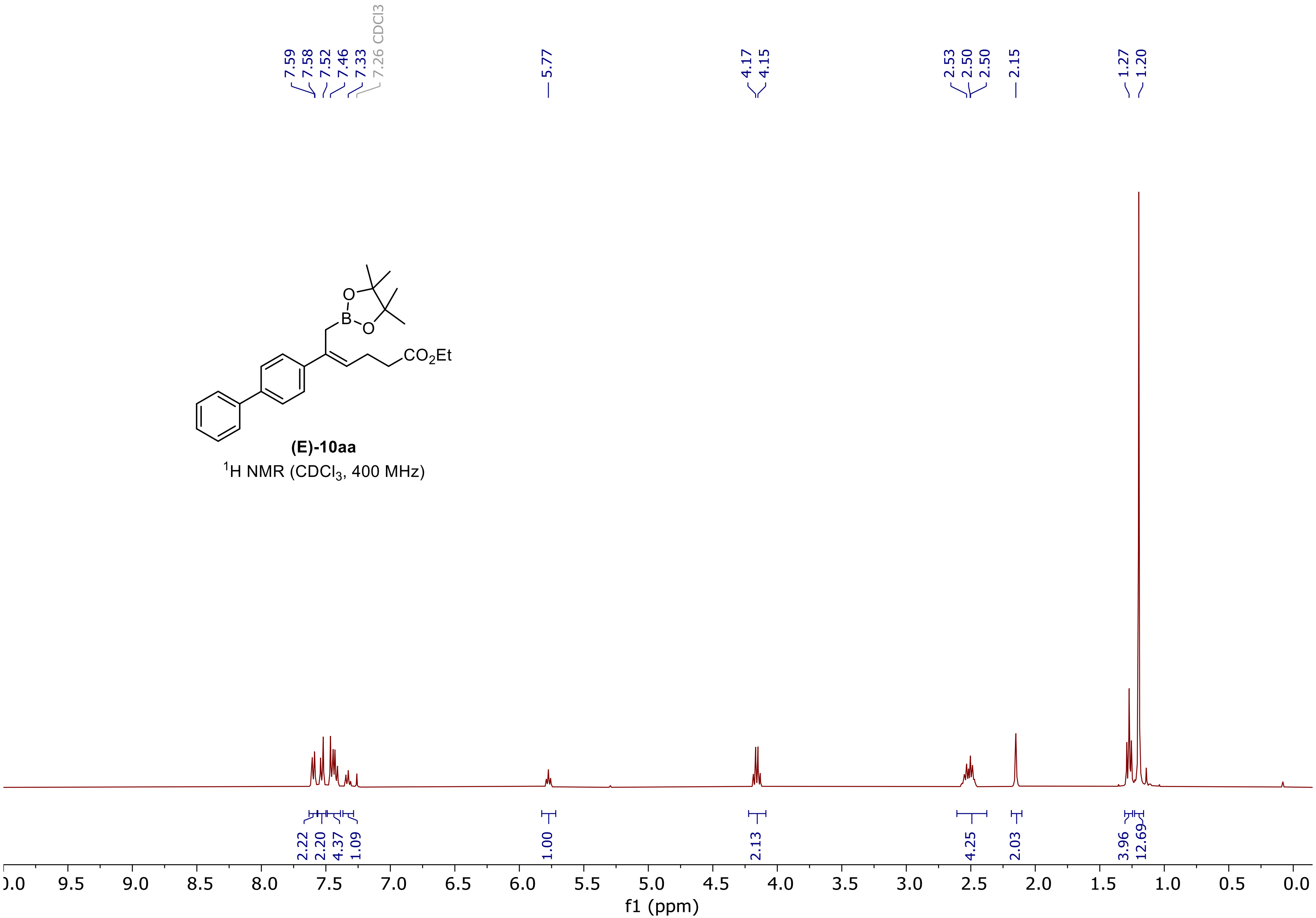

(E)-10aa

^{1}H NMR ($CDCl_3$, 400 MHz)

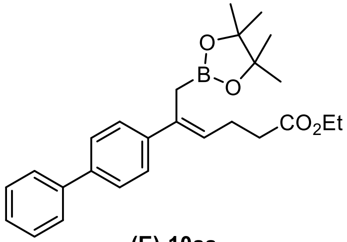

(E)-10aa

^{11}B NMR ($CDCl_3$, 128 MHz)

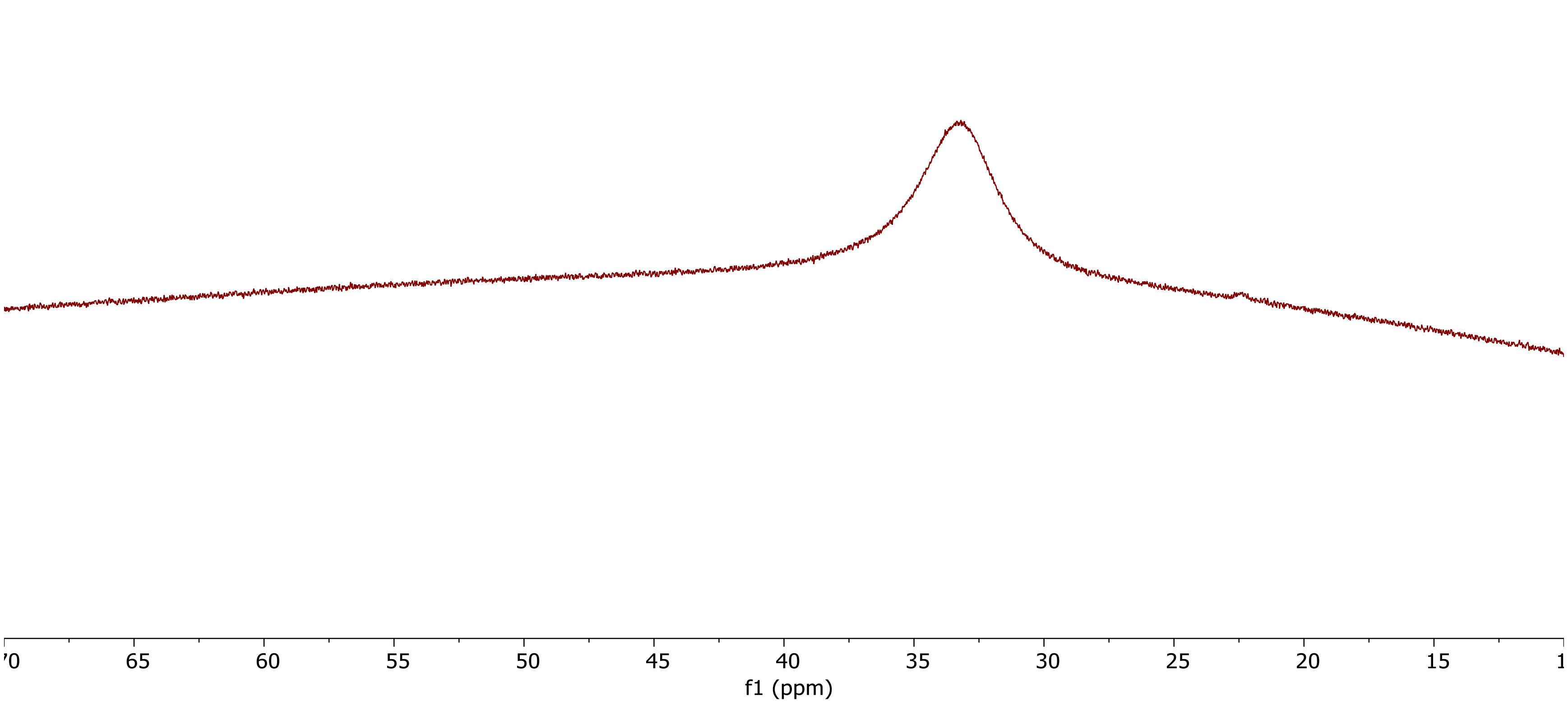

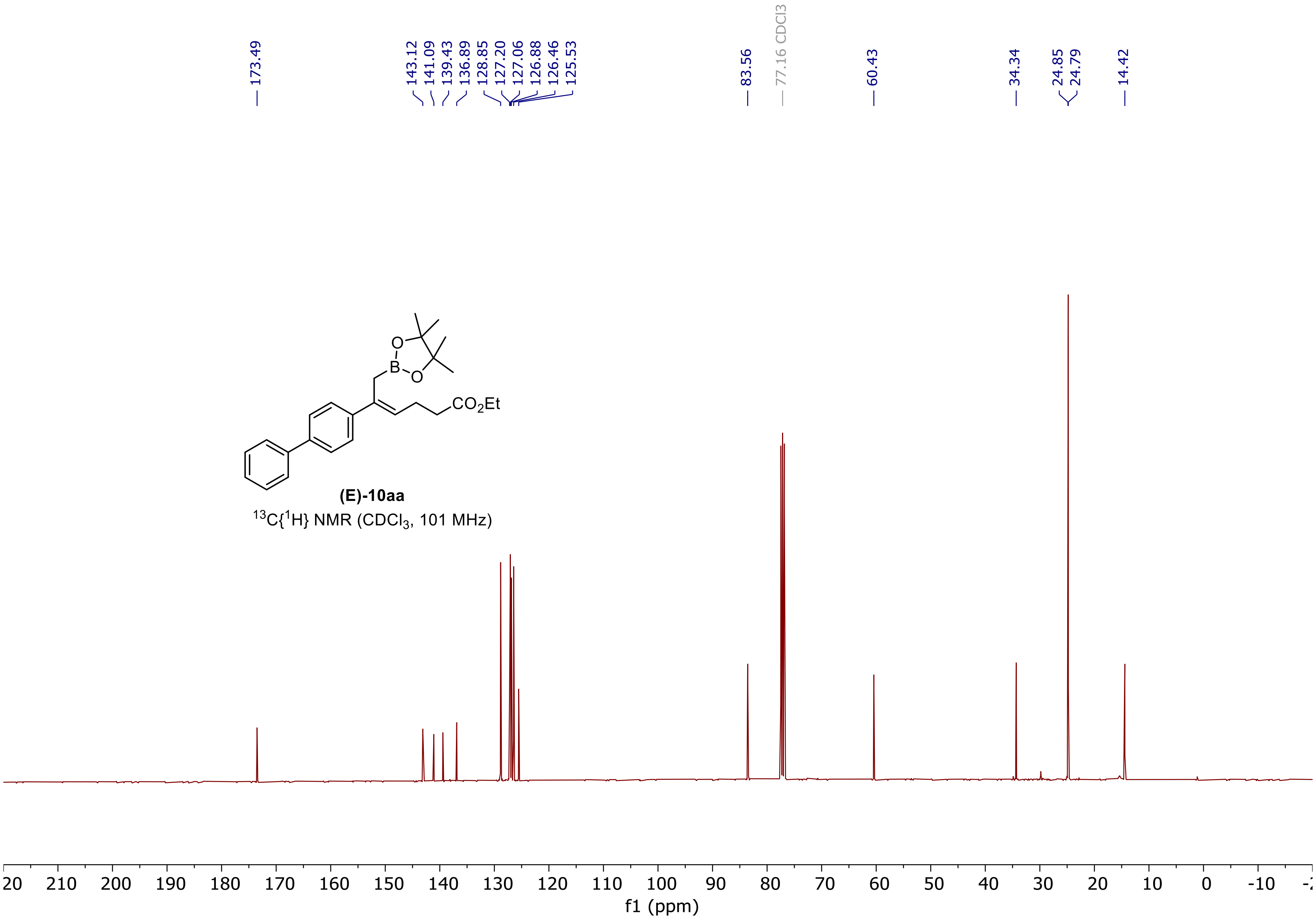

(E)-10aa

$^{13}C\{^1H\}$ NMR ($CDCl_3$, 101 MHz)

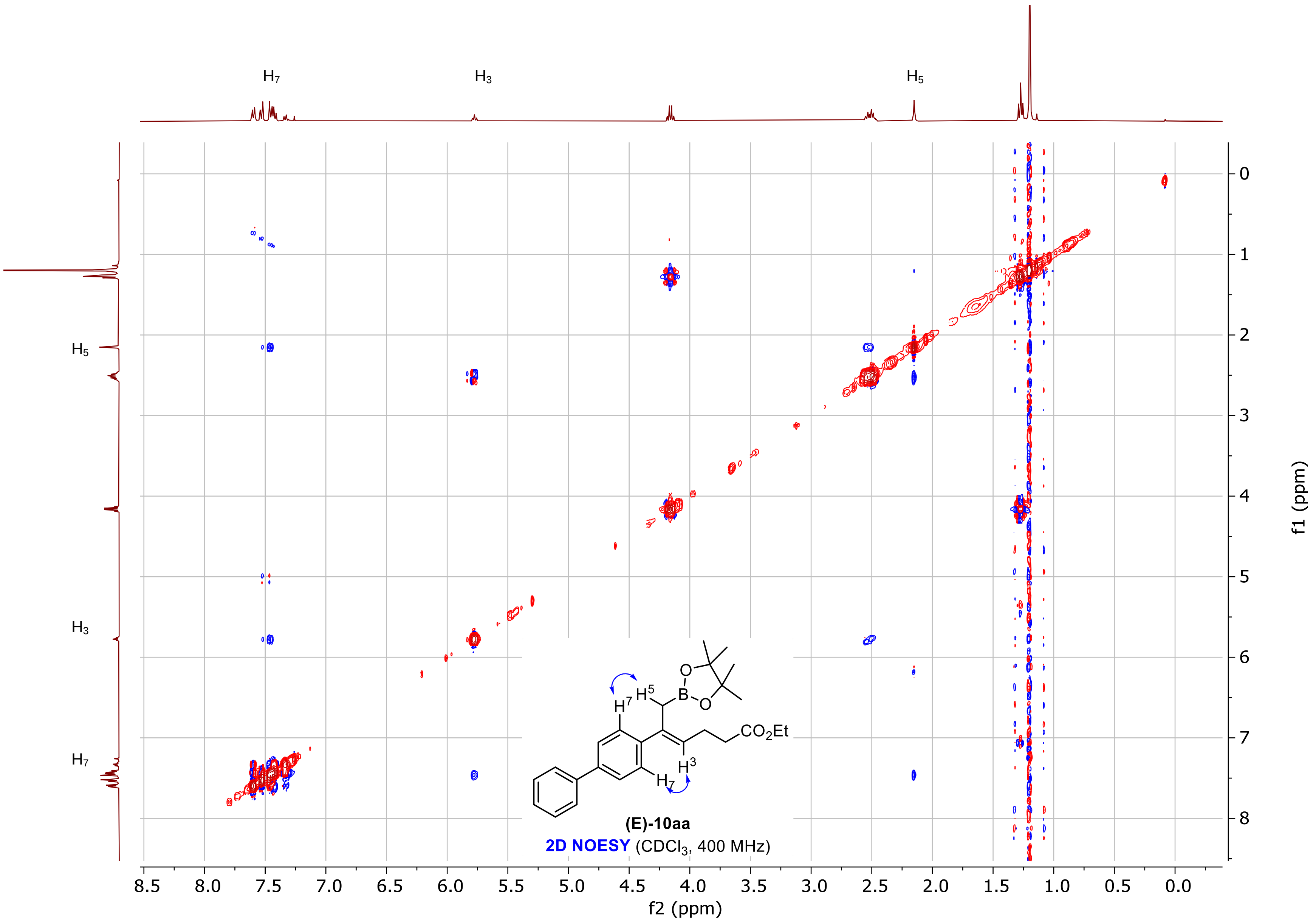
H7
H3
H5
H5
H3
H7
f2 (ppm)
f1 (ppm)
H7 H5
B
O
O
CO2Et
H3
H7
(E)-10aa
2D NOESY (CDCl3, 400 MHz)

10. ^{1}H NMR spectra of known compounds

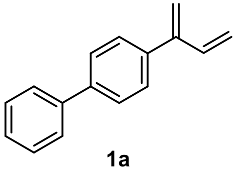

1a

^{1}H NMR ($CDCl_3$, 400 MHz)

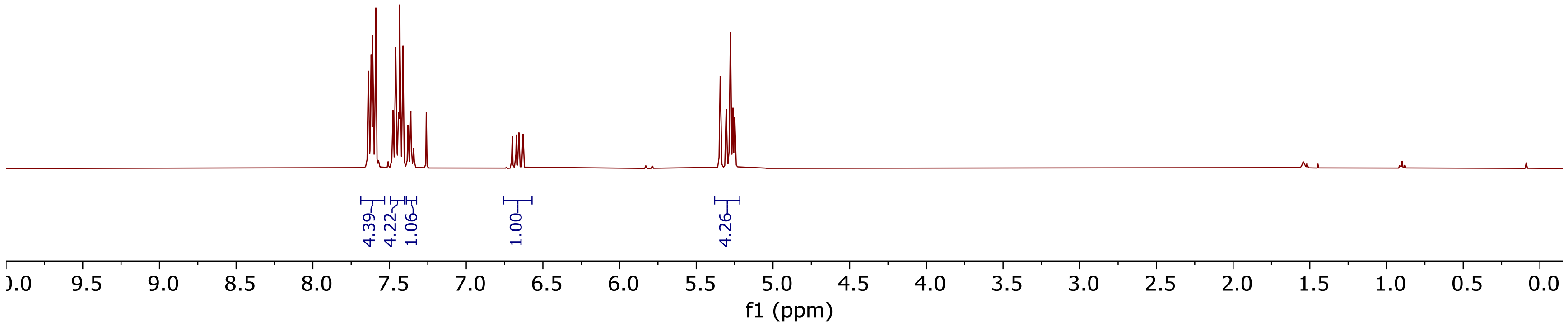

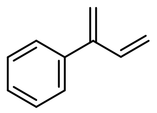

1b

^{1}H NMR ($CDCl_3$, 400 MHz)

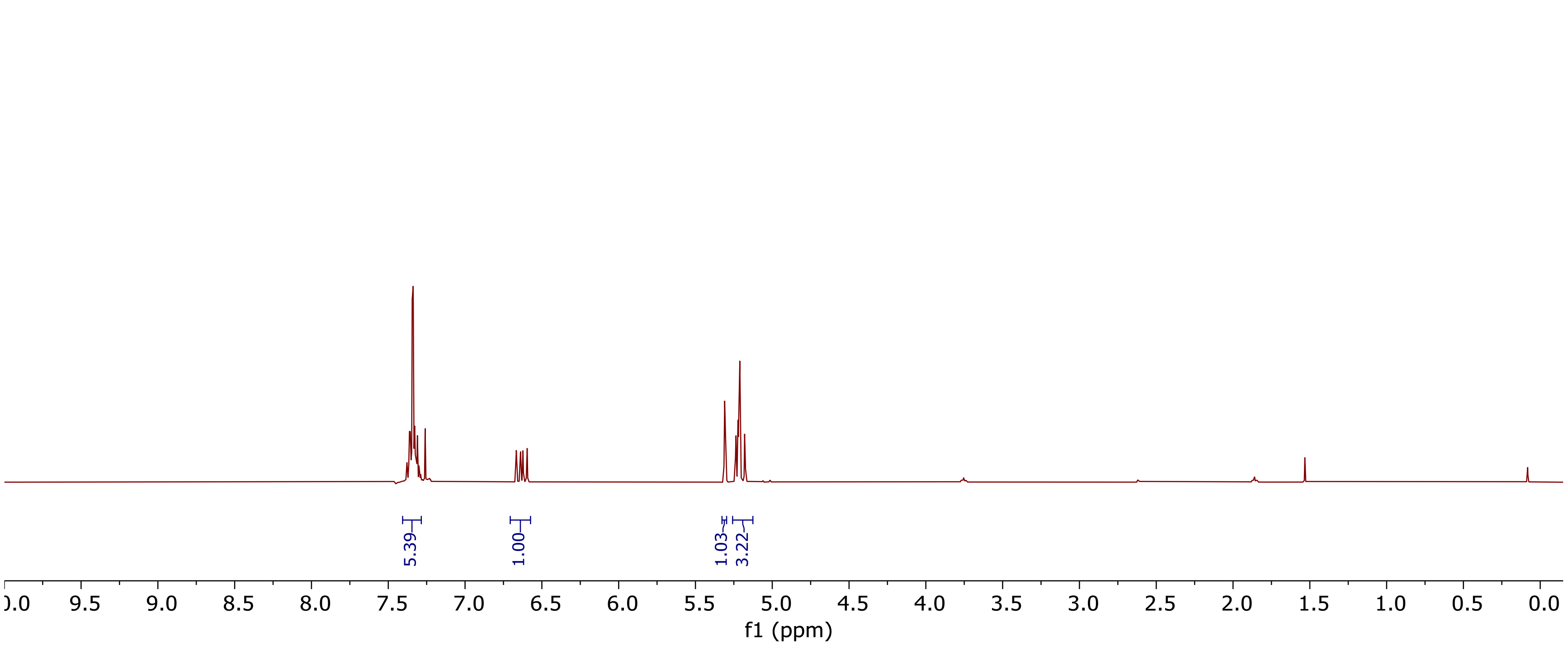

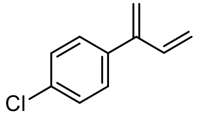

1c

^{1}H NMR ($CDCl_3$, 400 MHz)

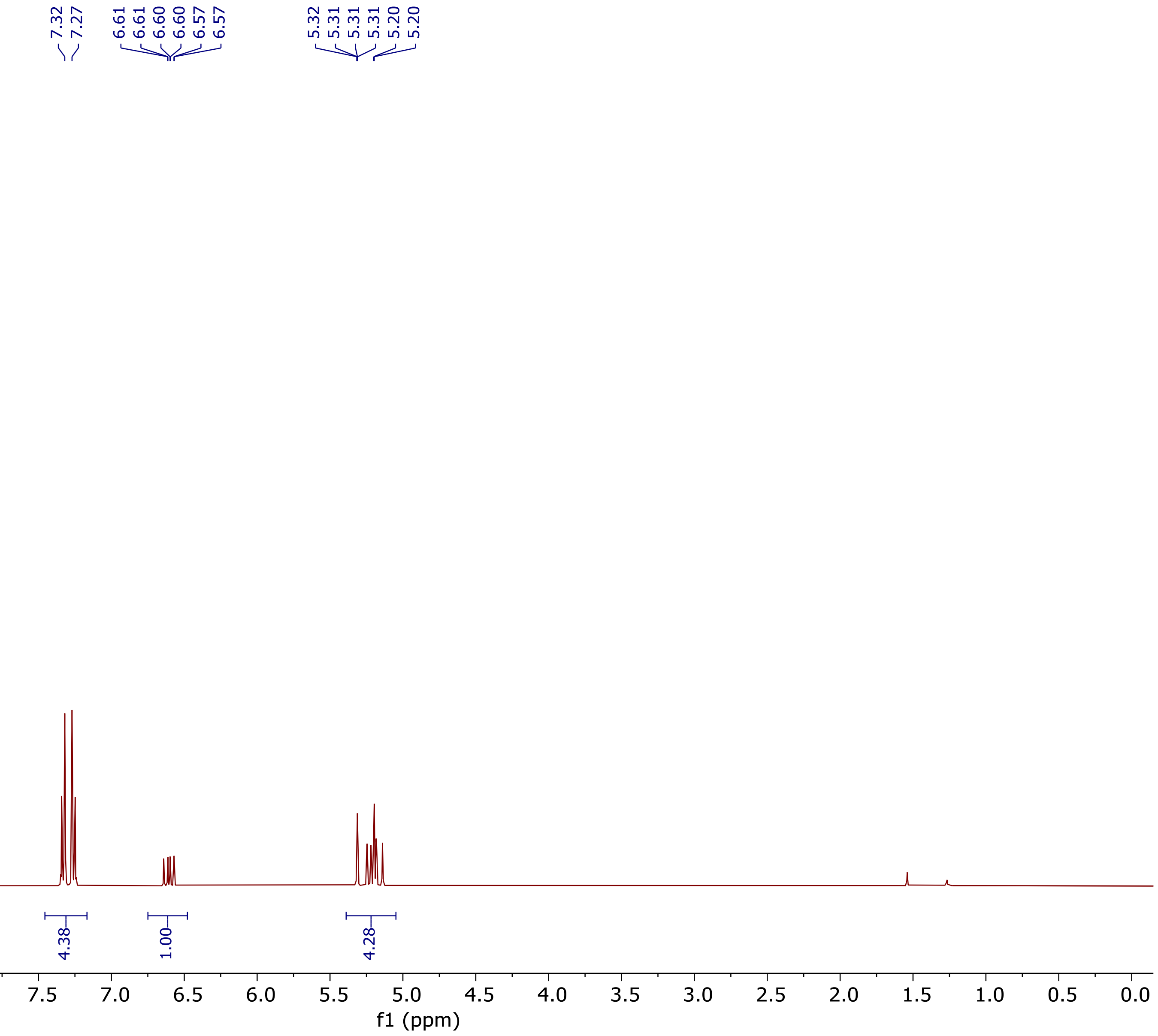

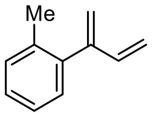

1d

^{1}H NMR ($CDCl_3$, 400 MHz)

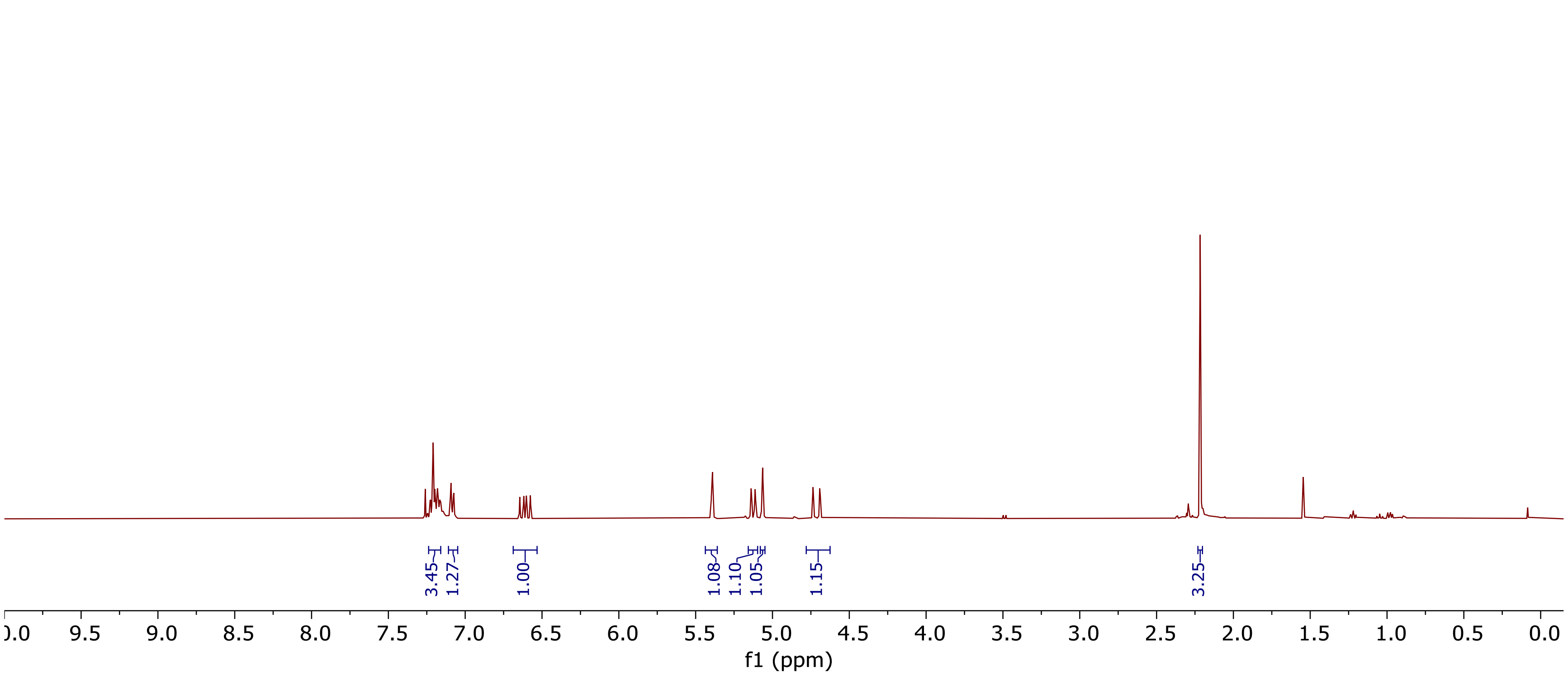

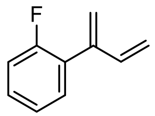

1e

^1H NMR ($CDCl_3$, 300 MHz)

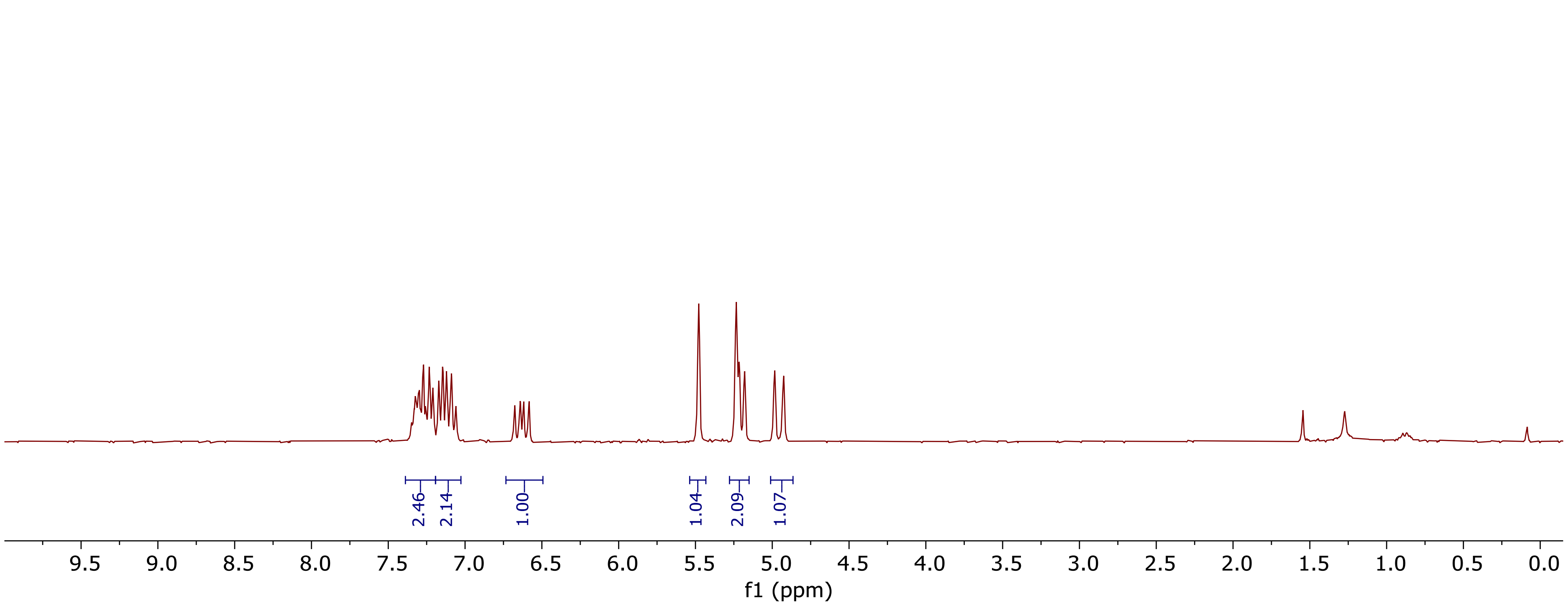

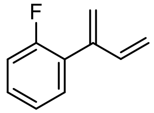

1e
$^{19}F\{^{1}H\}$ NMR ($CDCl_3$, 300 MHz)

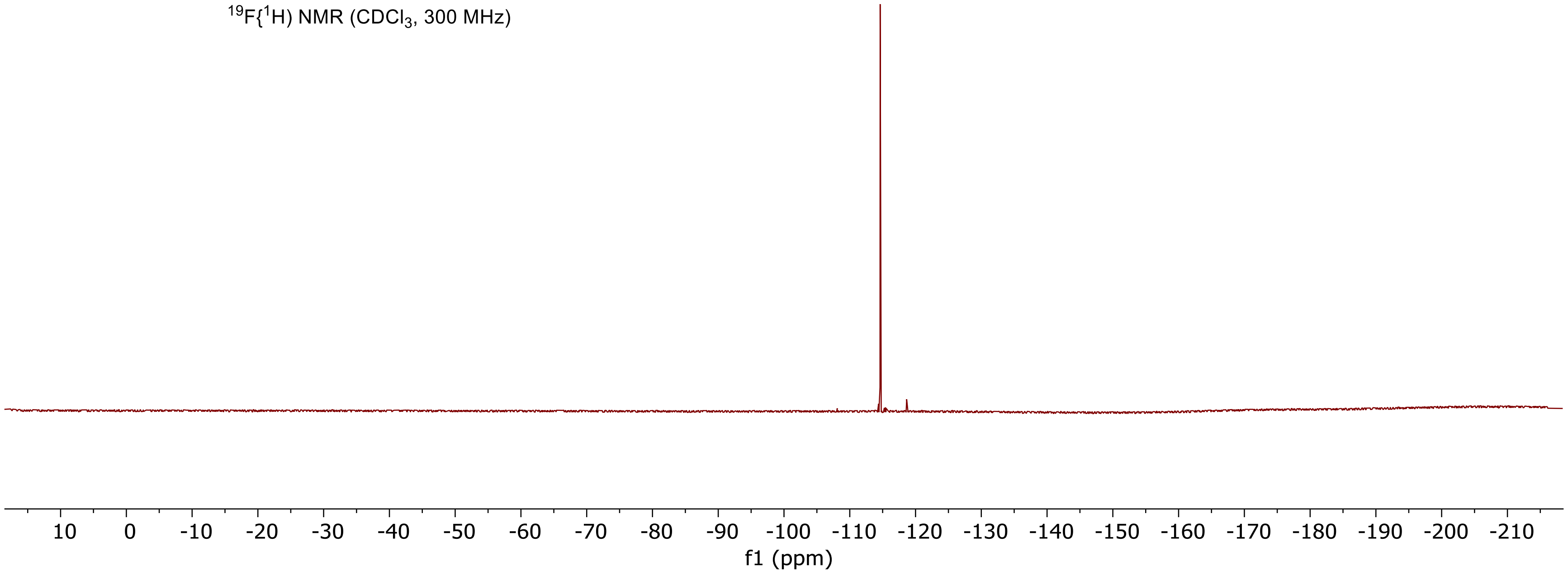

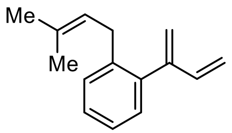

1f

^{1}H NMR ($CDCl_3$, 400 MHz)

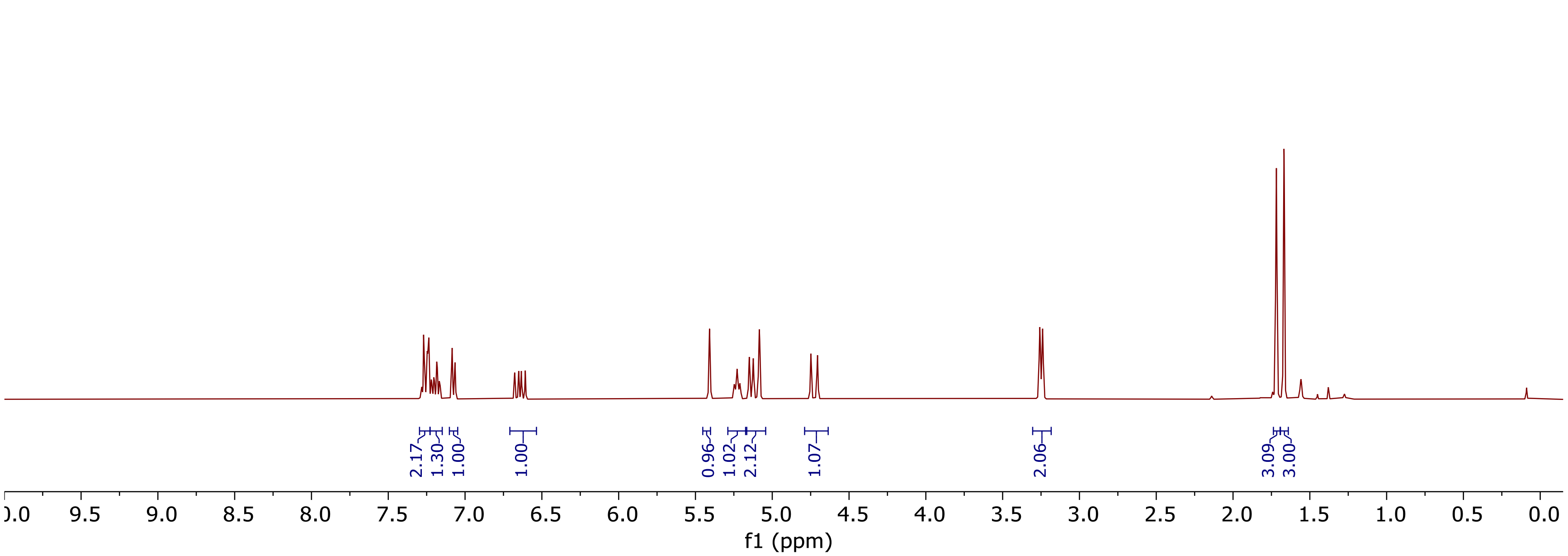

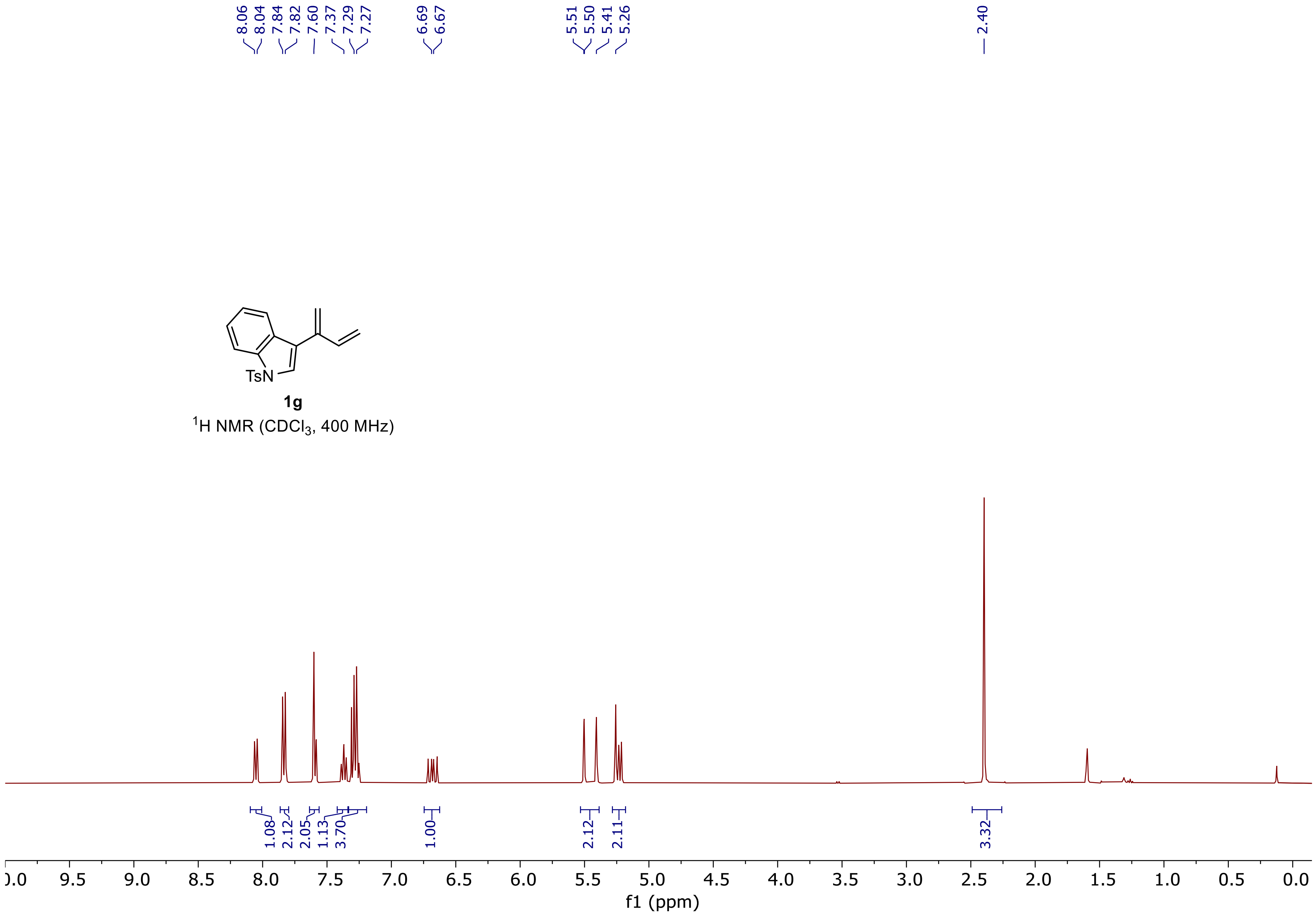

1g

^{1}H NMR ($CDCl_3$, 400 MHz)

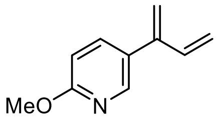

1h

^{1}H NMR ($CDCl_3$, 400 MHz)

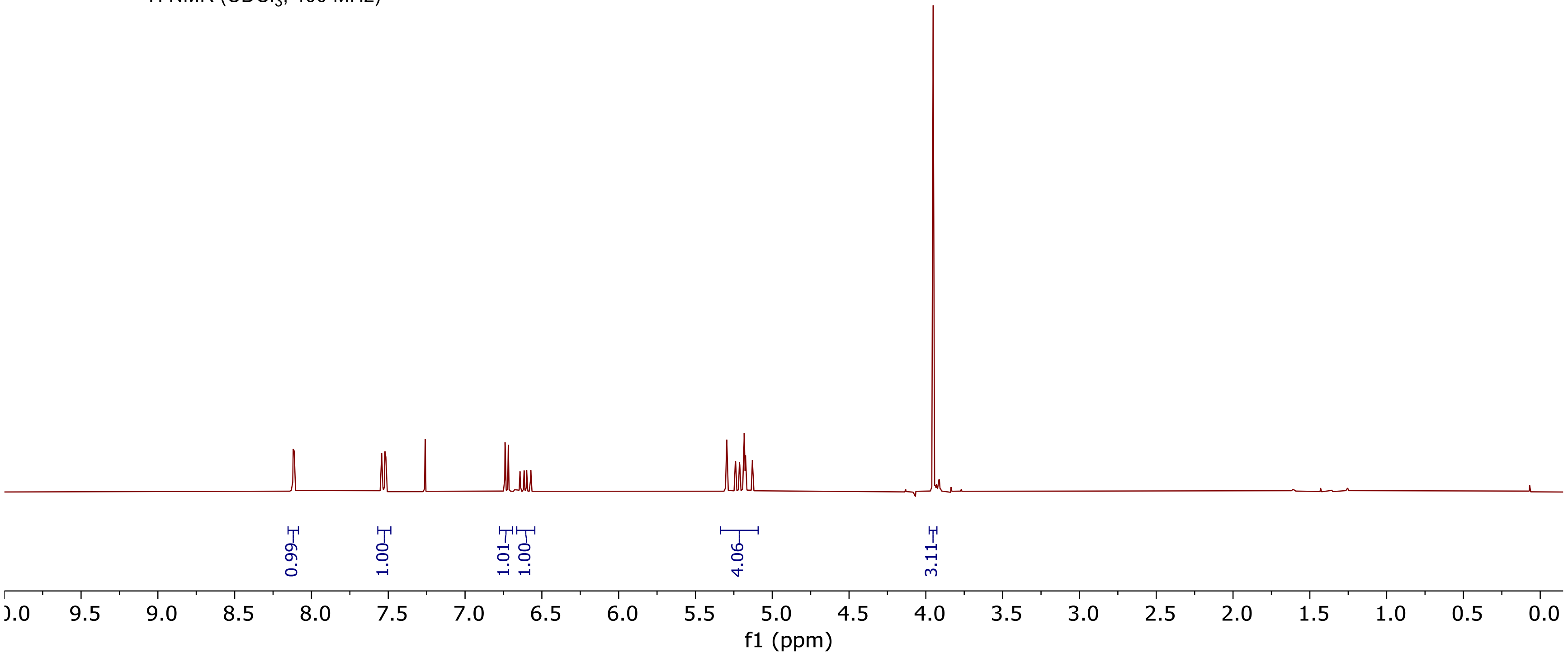

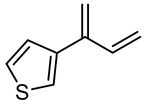

1i

^{1}H NMR ($CDCl_3$, 400 MHz)

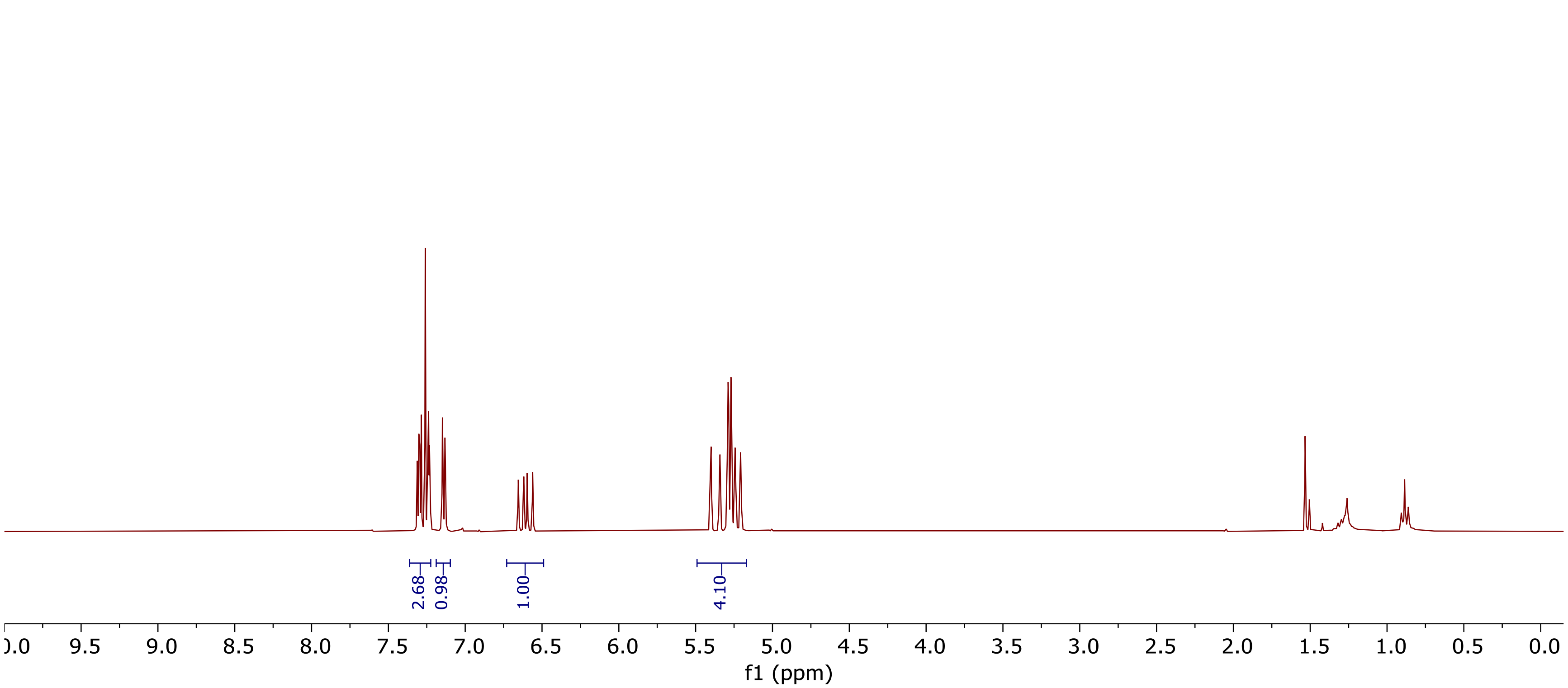

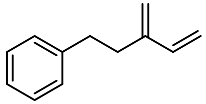

1j

^1H NMR ($CDCl_3$, 400 MHz)

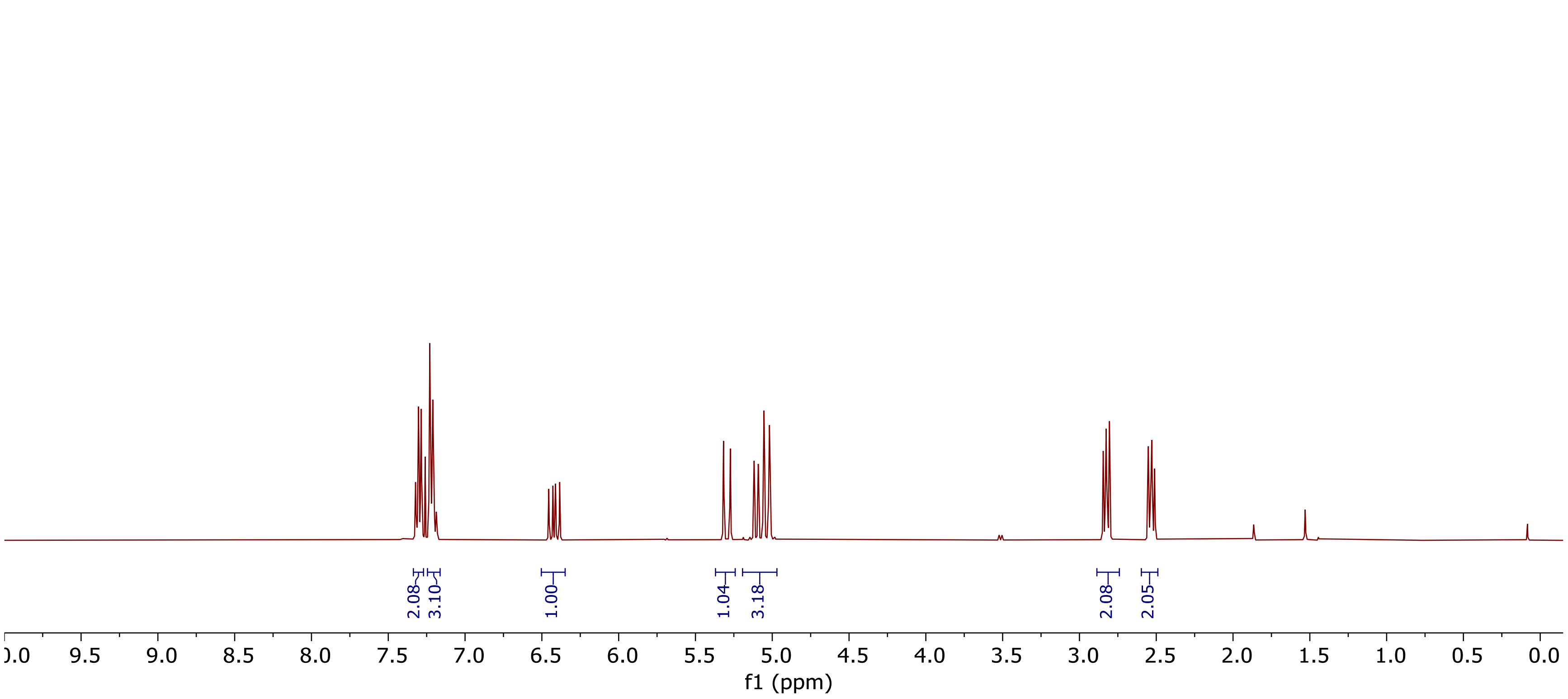

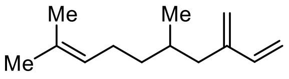

1k

^{1}H NMR ($CDCl_3$, 400 MHz)

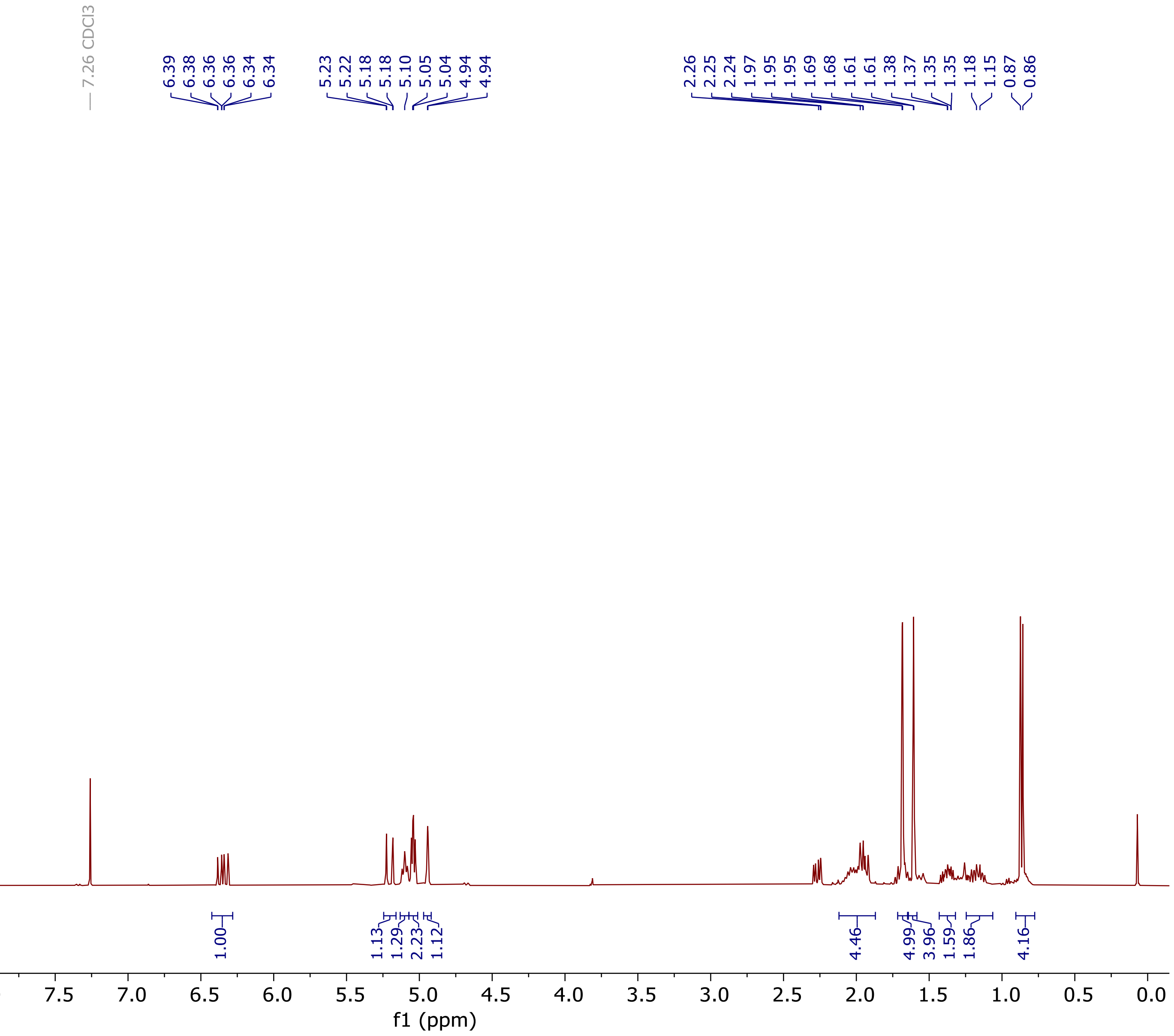

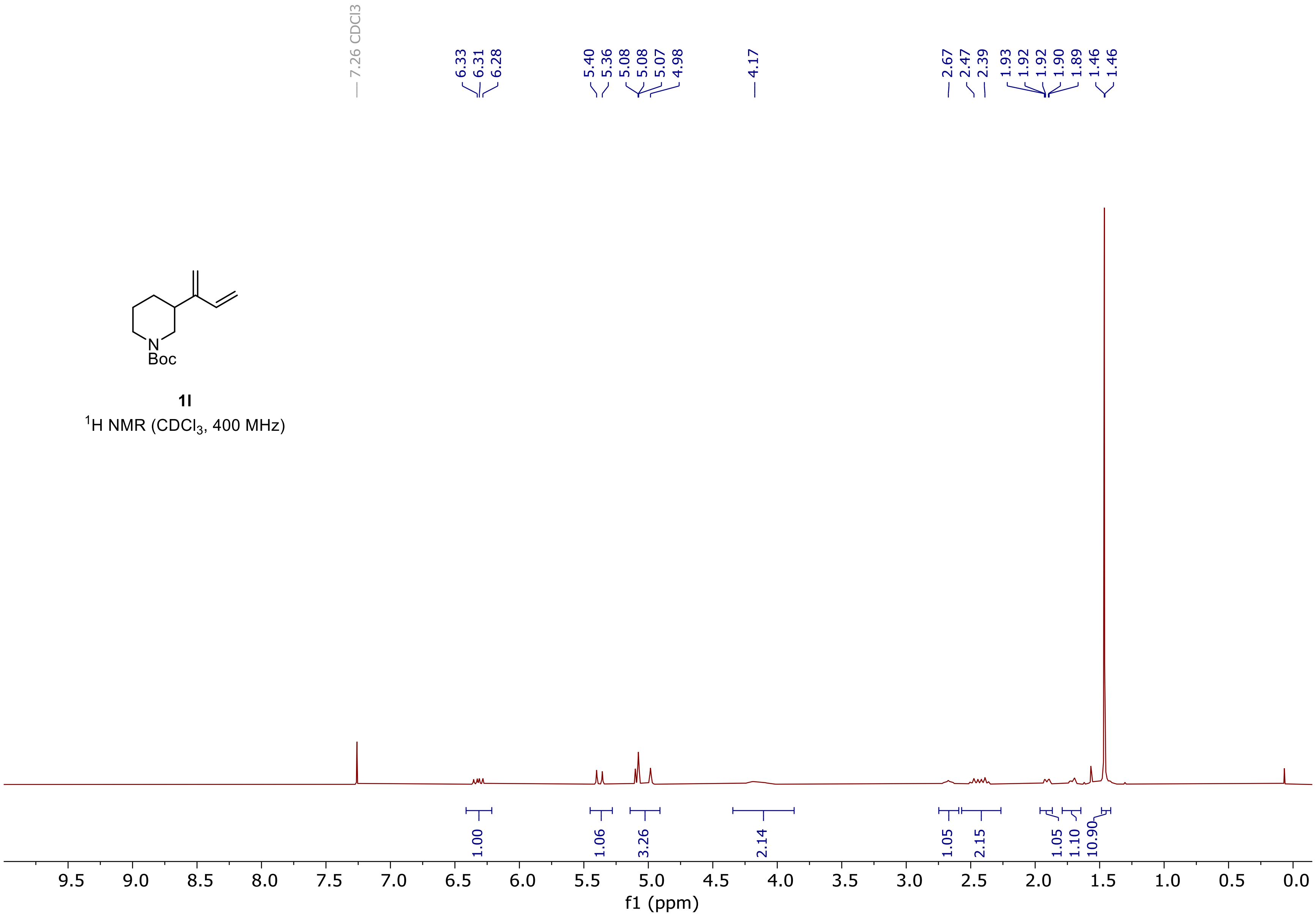

1l

^{1}H NMR ($CDCl_3$, 400 MHz)

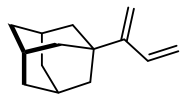

1m

^{1}H NMR ($CDCl_3$, 101 MHz)

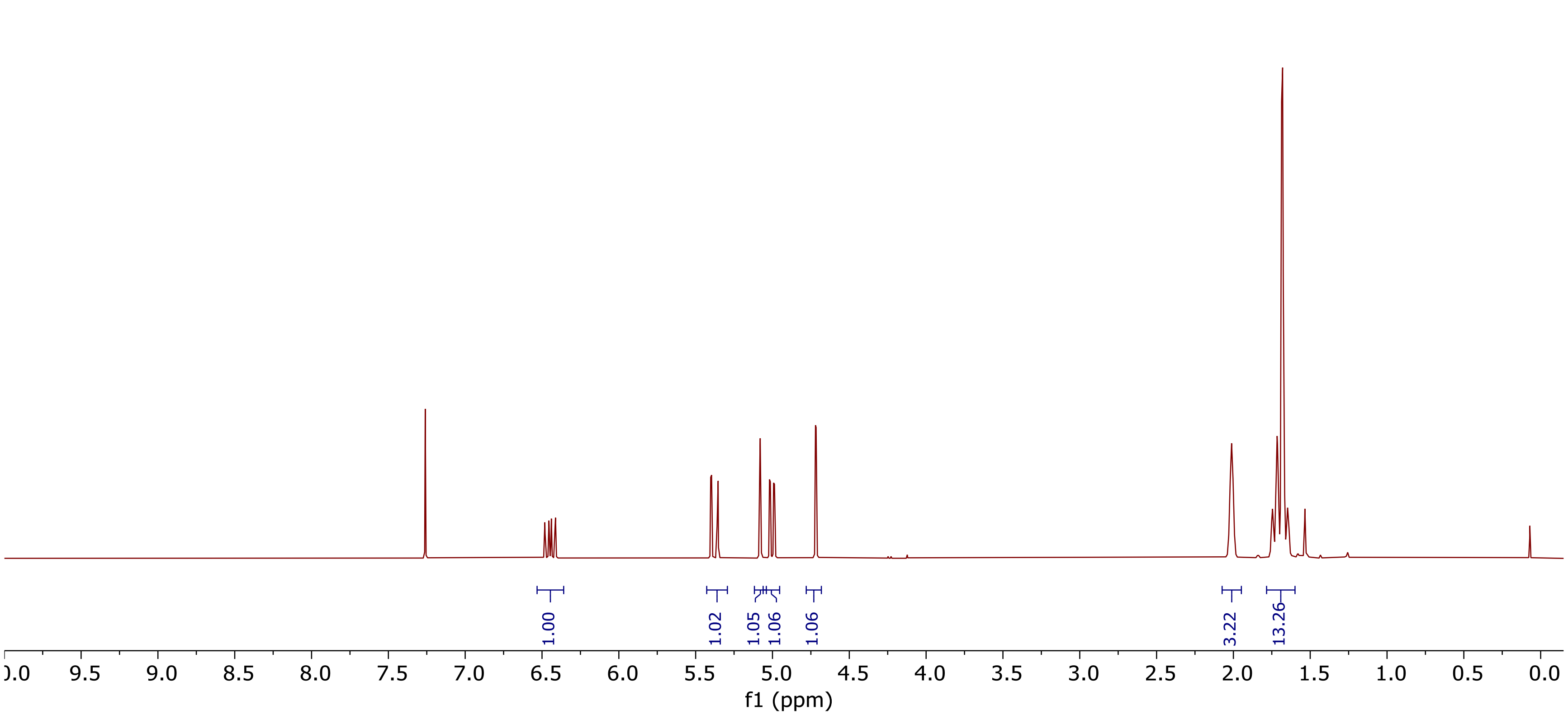

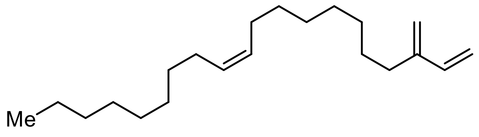

1n

^{1}H NMR ($CDCl_3$, 400 MHz)

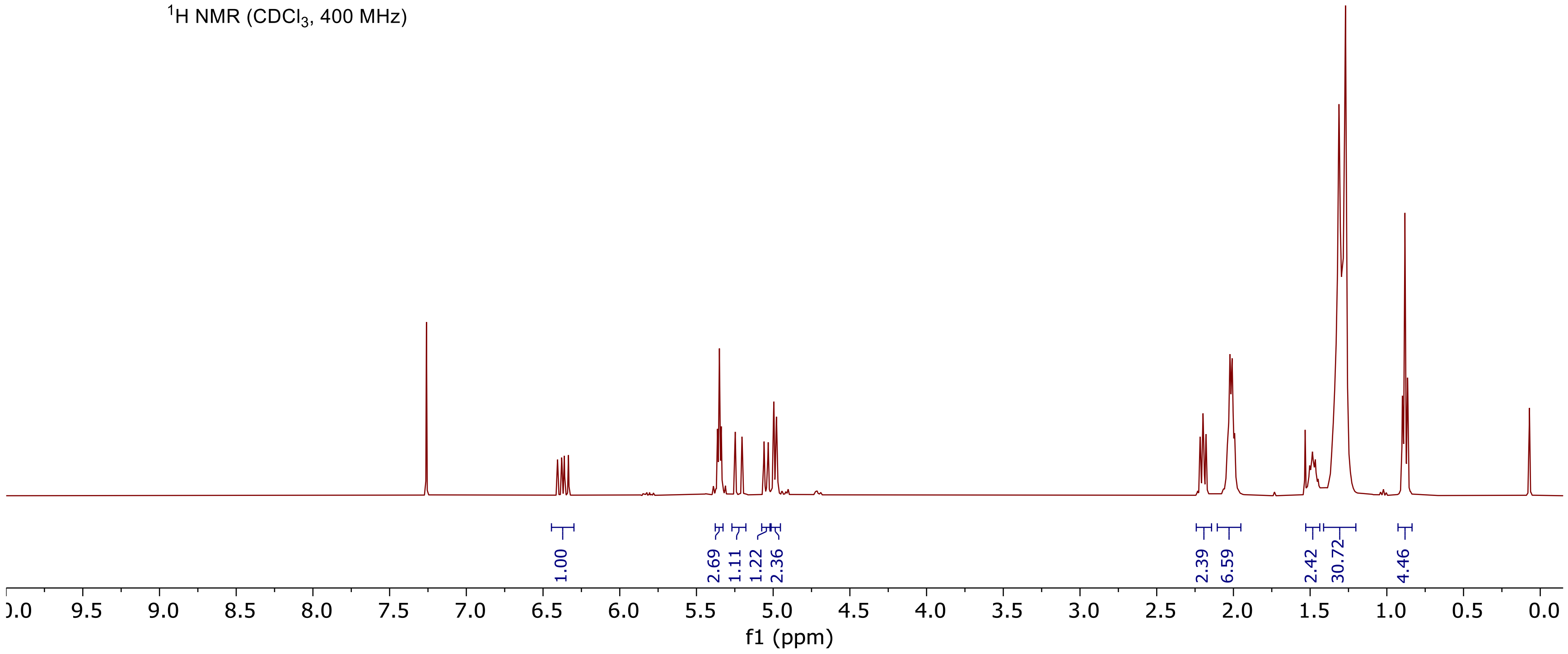

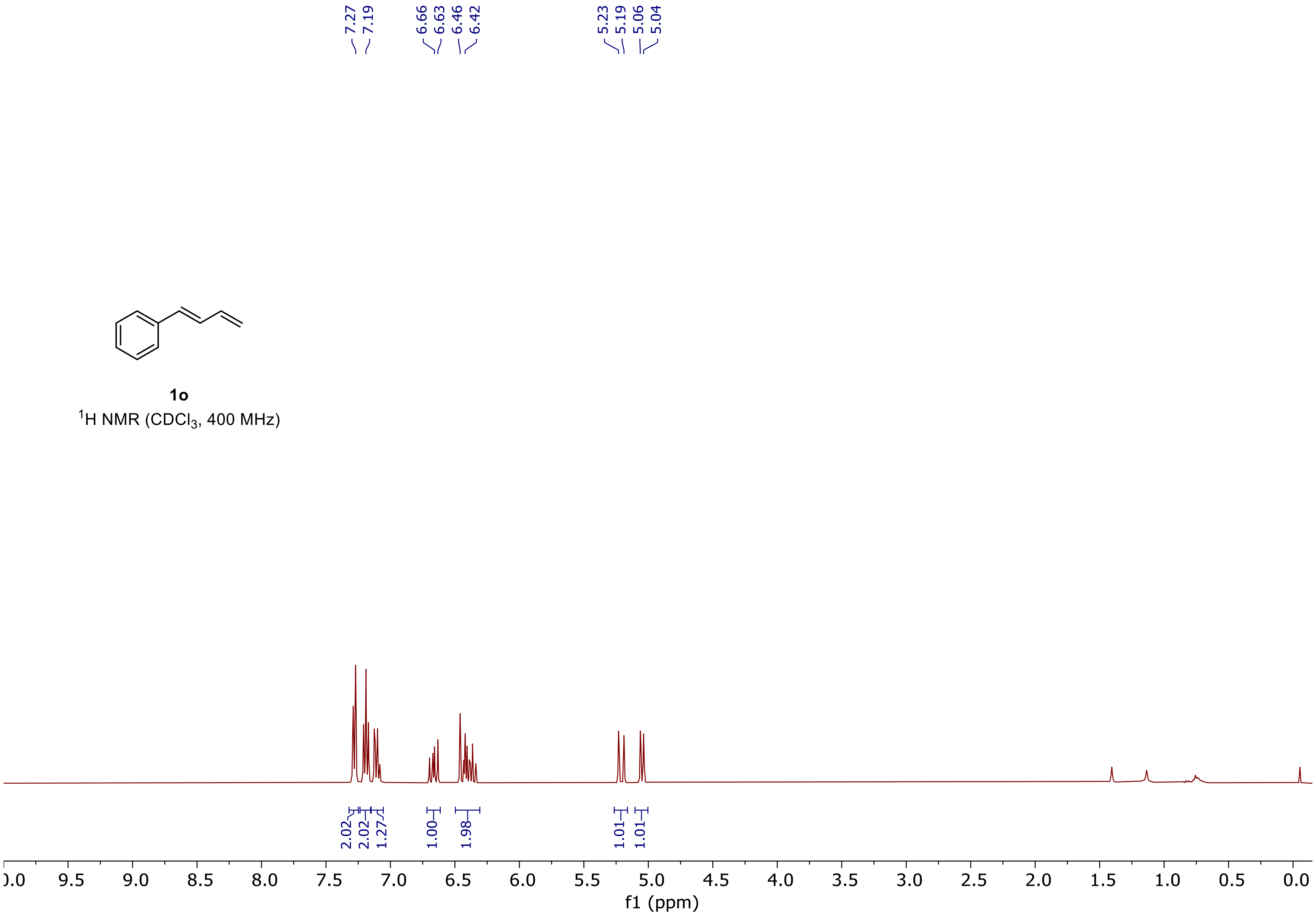

1o

^{1}H NMR ($CDCl_3$, 400 MHz)

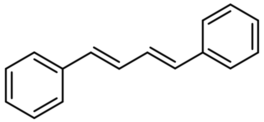

1p

^{1}H NMR ($CDCl_3$, 400 MHz)

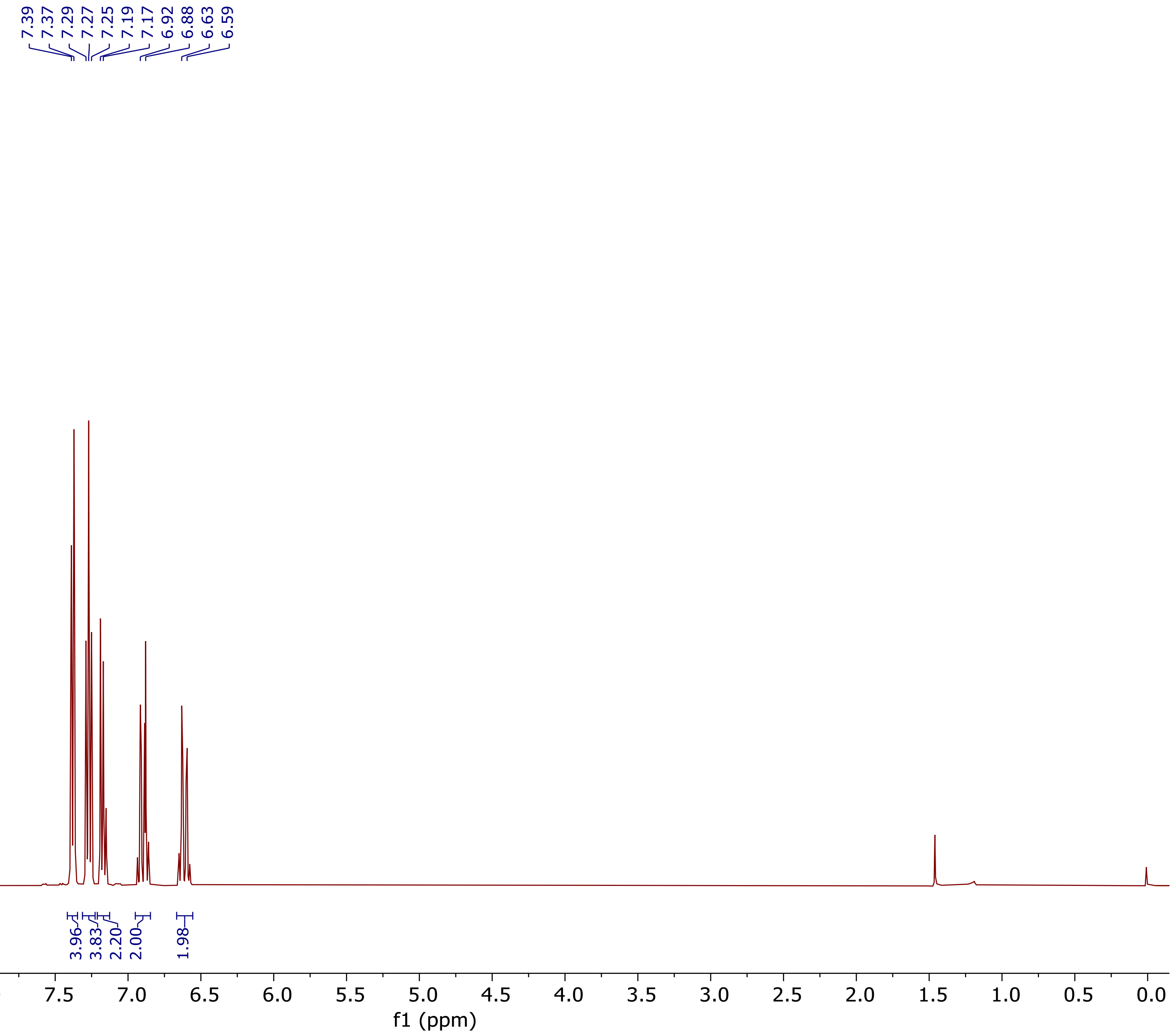

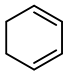

1q

^{1}H NMR ($CDCl_3$, 400 MHz)

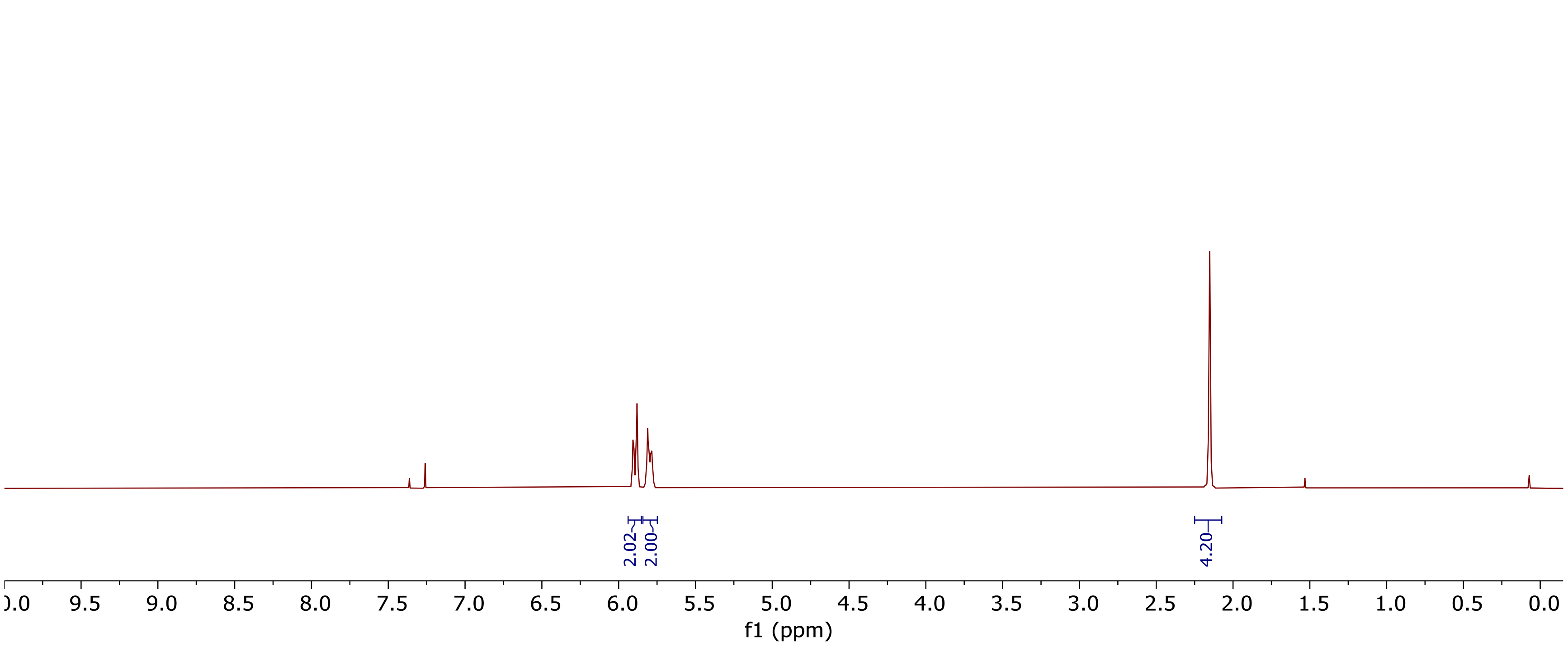

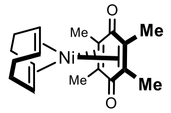

Ni(cod)(DQ)
^{1}H NMR (C_6D_6, 400 MHz)

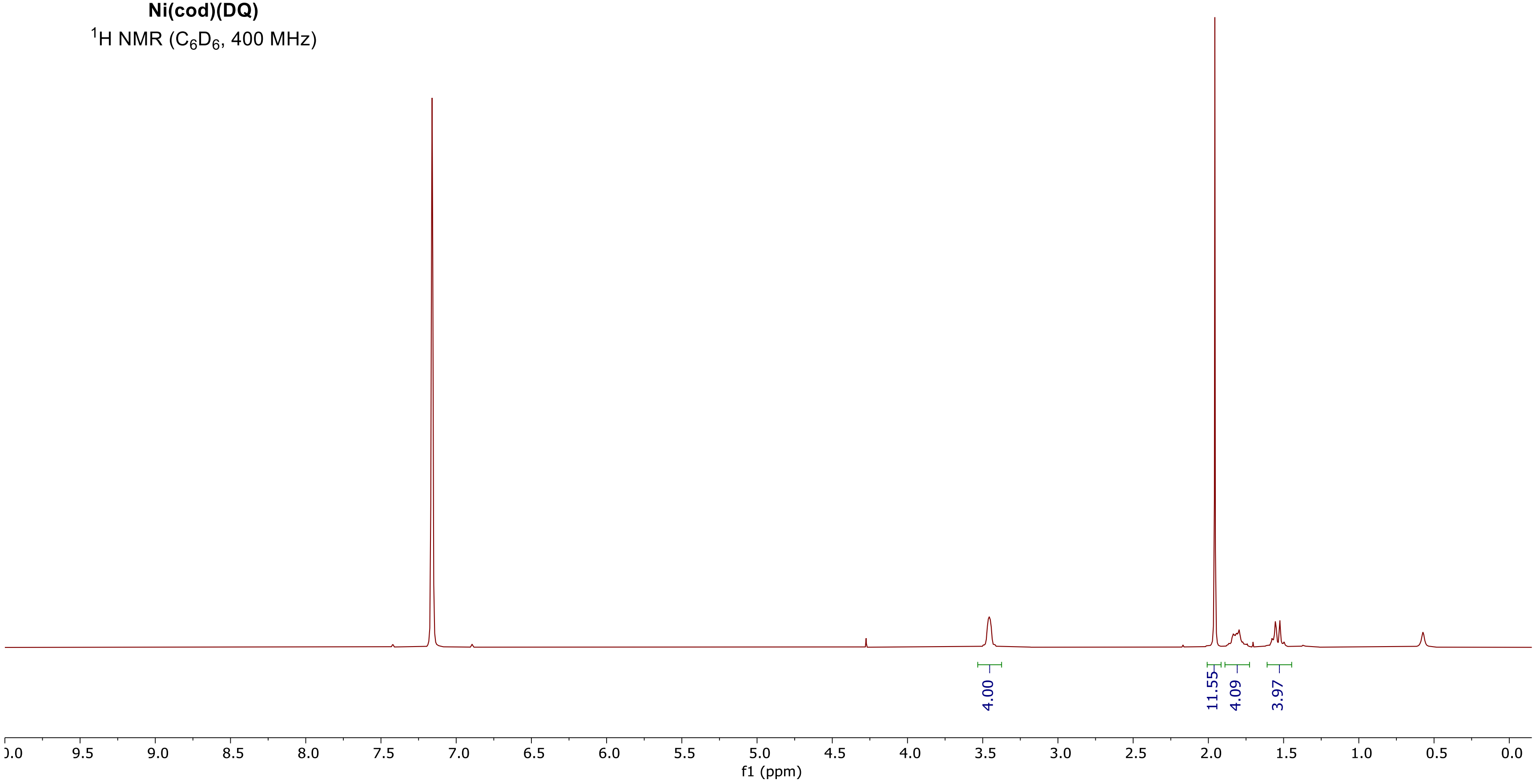

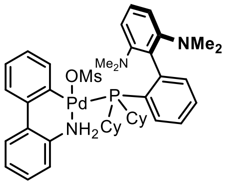

(CPhos)Pd(G3)

^{1}H NMR (THF-d_8, 400 MHz)

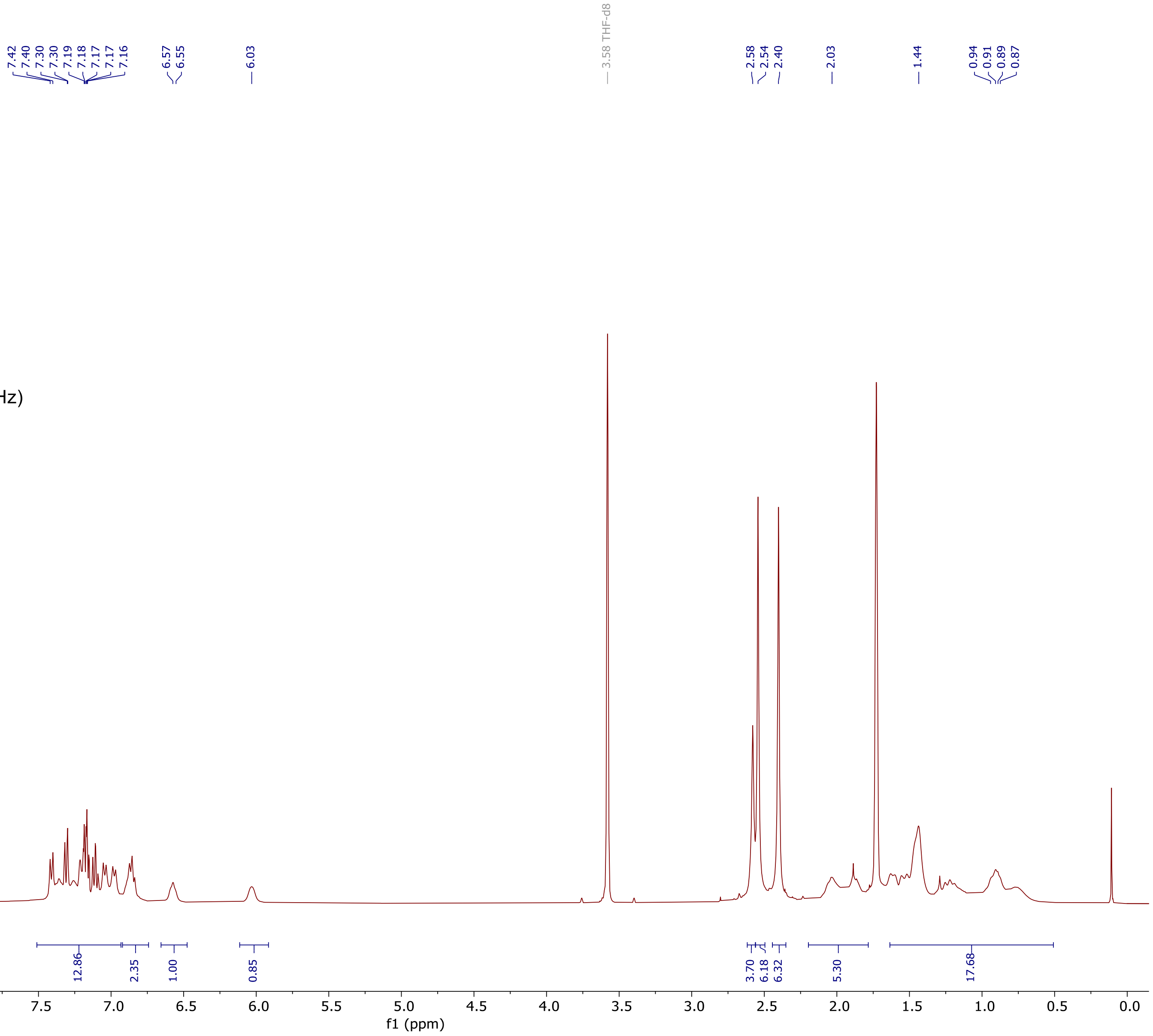

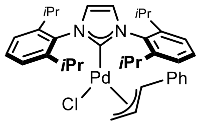

C_1

^{1}H NMR (THF-d_8, 400 MHz)

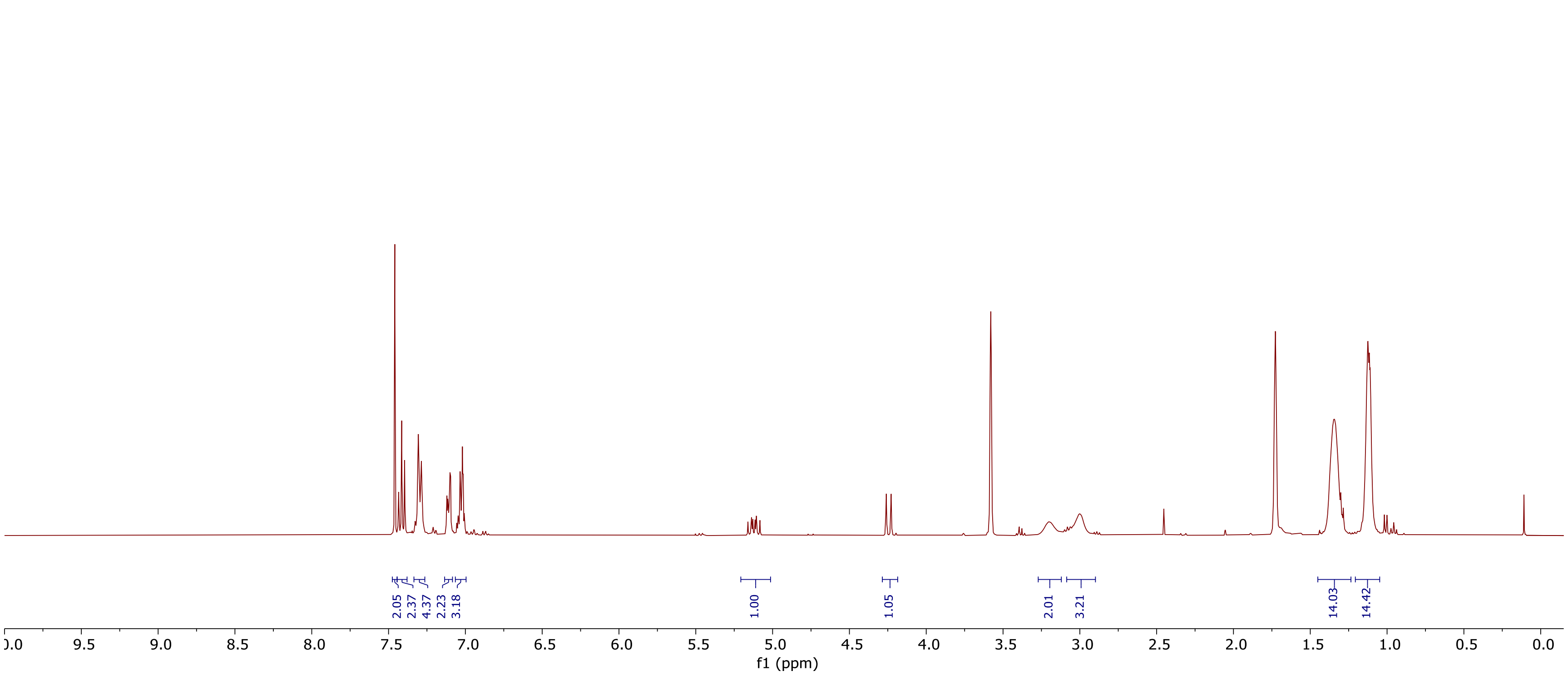

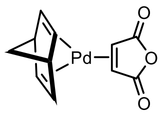

C_4

^{1}H NMR (THF-d_8, 400 MHz)

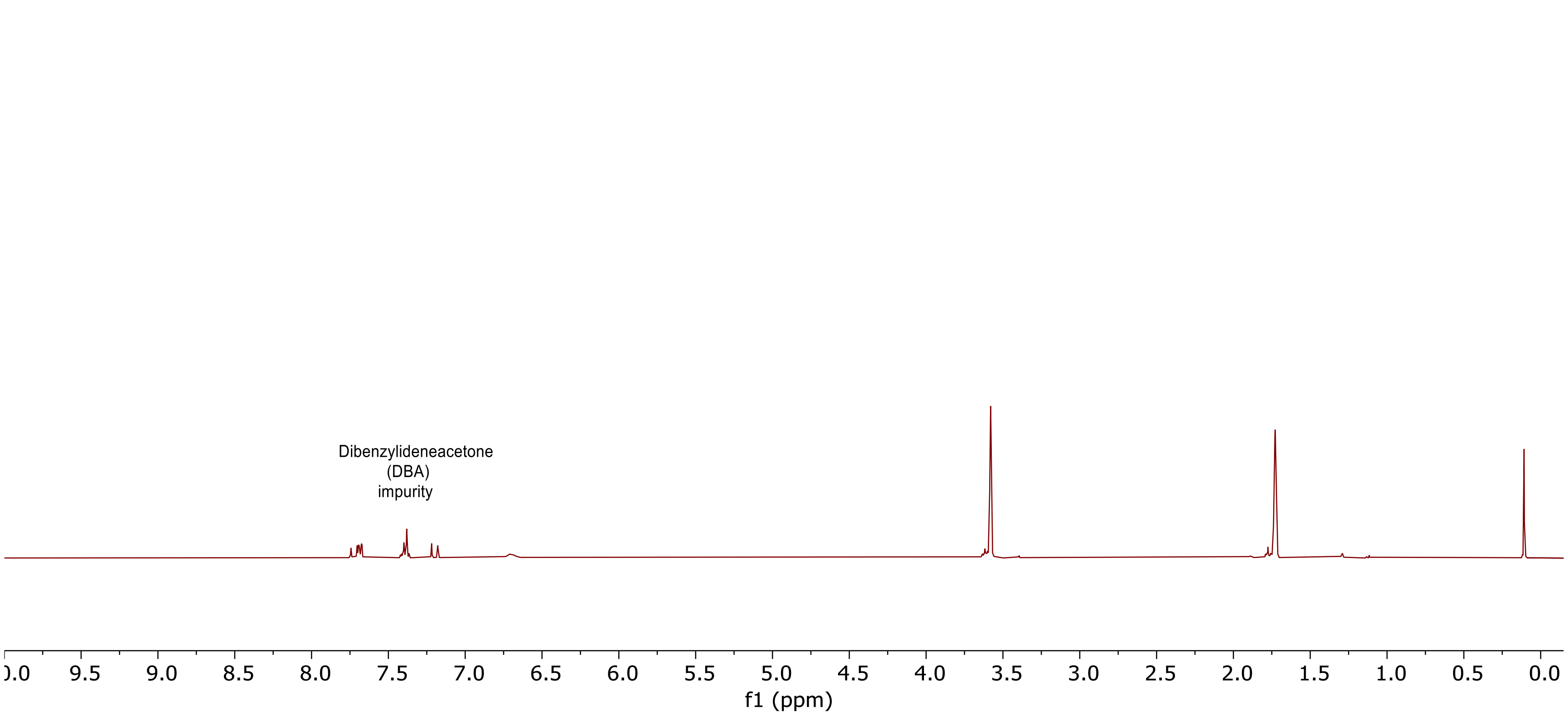

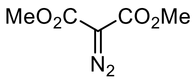

2b

^{1}H NMR ($CDCl_3$, 400 MHz)

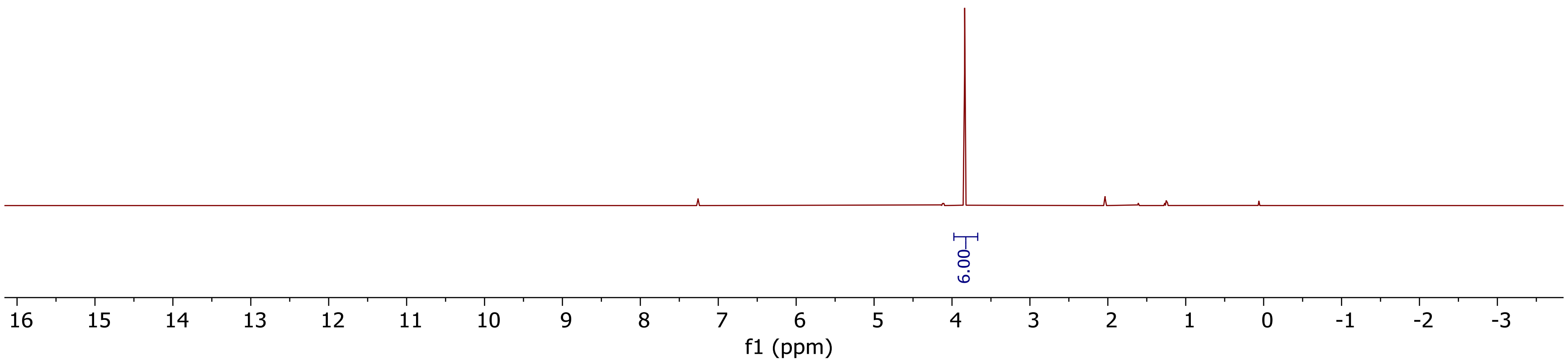

Supplement: Supplementary file 1 — gg3c00024_si_001.pdf [file gg3c00024_si_001.pdf]
